# Supplementary material for: Structural Genomics of SARS-CoV-2 Indicates Evolutionary Conserved Functional Regions of Viral Proteins
Source: Viruses. 2020 Mar 25;12(4):360. doi: 10.3390/v12040360 (PMC7232164; doi:10.3390/v12040360)
Supplement: Supplementary file 1 [file viruses-12-00360-s001.zip › wNsp16_LBS_mapped.pdf]

Supplementary File 7. Ligand binding sites mapped on the sequence of wNsp16 protein aligned with the closest homologs found in UniProt.

Ligand BS:

- S-ADENOSYLMETHIONINE (5YN6-SAM, MERS)
- S-ADENOSYL-L-HOMOCYSTEINE (5YN8-SAH, MERS)
- SINEFUNGIN (5YNB-SFG, MERS)
- P1-7-METHYLGUANOSINE-P3-ADENOSINE-5',5'-TRIPHOSPHATE (5YNF-GTA, MERS)
- 7-METHYL-GUANOSINE-5'-TRIPHOSPHATE-5'-GUANOSINE (5YNI-GTG, MERS)

```
QHN73794                             KVVKVTIDYTEISFMLWCKDGHVETFYPKLQSSQAWQPGVAMPNLYKMQRMLLEKCDLQN 6827
SP|P0C6X7|R1AB_CVHSA                 KVVKVTIDYAEISFMLWCKDGHVETFYPKLQASQAWQPGVAMPNLYKMQRMLLEKCDLQN 6804
TR|Q6UZF5|Q6UZF5_CVHSA               KVVKVTIDYAEISFMLWCKDGHVETFYPKLQASQAWQPGVAMPNLYKMQRMLLEKCDLQN 6804
TR|Q6UZF1|Q6UZF1_CVHSA               KVVKVTIDYAEISFMLWCKDGHVETFYPKLQASQAWQPGVAMPNLYKMQRMLLEKCDLQN 6804
TR|Q6JH48|Q6JH48_CVHSA               KVVKVTIDYAEISFMLWCKDGHVETFYPKLQASQAWQPGVAMPNLYKMQRMLLEKCDLQN 6804
TR|Q692E6|Q692E6_CVHSA               KVVKVTIDYAEISFMLWCKDGHVETFYPKLQASQAWQPGVAMPNLYKMQRMLLEKCDLQN 6804
TR|A0A0K1YZY7|A0A0K1YZY7_CVHSA       KVVKVTIDYVEISFMLWCKDGHVETFYPKLQASQAWQPGVAMPNLYKMQRMLLEKCDLQN 6804
SP|P0C6W2|R1AB_BCHK3                 KVVKVTIDYAEISFMLWCKDGHVETFYPKLQASQAWQPGVAMPNLYKMQRMLLEKCDLQN 6798
SP|P0C6W6|R1AB_BCRP3                 KVVKVTIDYAEISFMLWCKDGHVETFYPKLQASQAWQPGVAMPNLYKMQRMLLEKCDLQN 6802
SP|P0C6V9|R1AB_BC279                 KVVKVTIDYAEISFMLWCKDGHVETFYPKLQASQAWQPGVAMPNLYKMQRMLLEKCDLQN 6810
TR|A0A0U1WHI4|A0A0U1WHI4_CVHSA       KVVKVTIDYAEISFMLWCKDGHVETFYPKLQASQAWQPGVAMPNLYKMQRMLLEKCDLQN 6799
TR|A0A0U1WHG0|A0A0U1WHG0_CVHSA       KVVKVTIDYAEISFMLWCKDGYVETFYPKLQASQAWQPGVAMPNLYKMQRMLLEKCDLQN 6799
TR|A0A166ZL34|A0A166ZL34_9NIDO       KVVKVTIDYAEISFMLWCKDGYVETFYPKLQASQAWQPGVAMPNLYKMQRMLLEKCDLQN 6606
TR|R9QTB2|R9QTB2_CVHSA               ------------------------------------------------------------
TR|R9QTH2|R9QTH2_CVHSA               ------------------------------------------------------------
SP|P0C6U8|R1A_CVHSA                  ------------------------------------------------------------
TR|Q6JH47|Q6JH47_CVHSA               ------------------------------------------------------------
TR|Q692E5|Q692E5_CVHSA               ------------------------------------------------------------
SP|P0C6F8|R1A_BCHK3                  ------------------------------------------------------------
TR|A0A0K1Z0N1|A0A0K1Z0N1_CVHSA       ------------------------------------------------------------
SP|P0C6F5|R1A_BC279                  ------------------------------------------------------------
SP|P0C6T7|R1A_BCRP3                  ------------------------------------------------------------

QHN73794                             YGDSATLPKGIMMNVAKYTQLCQYLNTLTLAVPYNMRVIHFGAGSDKGVAPGTAVLRQWL 6887
SP|P0C6X7|R1AB_CVHSA                 YGENAVIPKGIMMNVAKYTQLCQYLNTLTLAVPYNMRVIHFGAGSDKGVAPGTAVLRQWL 6864
TR|Q6UZF5|Q6UZF5_CVHSA               YGENAVIPKGIMMNVAKYTQLCQYLNTLTLAVPYNMRVIHFGAGSDKGVAPGTAVLRQWL 6864
TR|Q6UZF1|Q6UZF1_CVHSA               YGENAVIPKGIMMNVAKYTQLCQYLNTLTLAVPYNMRVIHFGAGSDKGVAPGTAVLRQWL 6864
TR|Q6JH48|Q6JH48_CVHSA               YGENAVIPKGIMMNVAKYTQLCQYLNTLTLAVPYNMRVIHFGAGSDKGVAPGTAVLRQWL 6864
TR|Q692E6|Q692E6_CVHSA               YGENAVIPKGIMMNVAKYTQLCQYLNTLTLAVPYNMRVIHFGAGSDKGVAPGTAVLRQWL 6864
TR|A0A0K1YZY7|A0A0K1YZY7_CVHSA       YGENAVIPKGIMMNVAKYTQLCQYLNTLTLAVPYNMRVIHFGAGSDKGVAPGTAVLRQWL 6864
SP|P0C6W2|R1AB_BCHK3                 YGENAVIPKGIMMNVAKYTQLCQYLNTLTLAVPYNMRVIHFGAGSDKGVAPGTAVLRQWL 6858
SP|P0C6W6|R1AB_BCRP3                 YGENAVIPKGIMMNVAKYTQLCQYLNTLTLAVPYNMRVIHFGAGSDKGVAPGTAVLRQWL 6862
SP|P0C6V9|R1AB_BC279                 YGENAVIPKGIMMNVAKYTQLCQYLNTLTLAVPYNMRVIHFGAGSDKGVAPGTAVLRQWL 6870
TR|A0A0U1WHI4|A0A0U1WHI4_CVHSA       YGENAVIPKGIMMNVAKYTQLCQYLNTLTLAVPYNMRVIHFGAGSDKGVAPGTAVLRQWL 6859
TR|A0A0U1WHG0|A0A0U1WHG0_CVHSA       YGENAVIPKGIMMNVAKYTQLCQYLNTLTLAVPYNMRVIHFGAGSDKGVAPGTAVLRQWL 6859
TR|A0A166ZL34|A0A166ZL34_9NIDO       YGENAVIPKGIMMNVAKYTQLCQYLNTLTLAVPYNMRVIHFGAGSDKGVAPGTAVLRQWL 6666
TR|R9QTB2|R9QTB2_CVHSA               ------------------------------------------------------------
TR|R9QTH2|R9QTH2_CVHSA               ------------------------------------------------------------
SP|P0C6U8|R1A_CVHSA                  ------------------------------------------------------------
TR|Q6JH47|Q6JH47_CVHSA               ------------------------------------------------------------
TR|Q692E5|Q692E5_CVHSA               ------------------------------------------------------------
SP|P0C6F8|R1A_BCHK3                  ------------------------------------------------------------
TR|A0A0K1Z0N1|A0A0K1Z0N1_CVHSA       ------------------------------------------------------------
SP|P0C6F5|R1A_BC279                  ------------------------------------------------------------
SP|P0C6T7|R1A_BCRP3                  ------------------------------------------------------------
```

```
QHN73794                         PTGTLLVDSDLNDFVSDADSTLIGDCATVHTANKWDLIISDMYDPKTKNVTKENDSKEGF 6947
SP|P0C6X7|R1AB_CVHSA             PTGTLLVDSDLNDFVSDADSTLIGDCATVHTANKWDLIISDMYDPRTKHVTKENDSKEGF 6924
TR|Q6UZF5|Q6UZF5_CVHSA           PTGTLLVDSDLNDFVSDADSTLIGDCATVHTANKWDLIISDMYDPRTKHVTKENDSKEGF 6924
TR|Q6UZF1|Q6UZF1_CVHSA           PTGTLLVDSDLNDFVSDADSTLIGDCATVHTANKWDLIISDMYDPRTKHVTKENDSKEGF 6924
TR|Q6JH48|Q6JH48_CVHSA           PTGTLLVDSDLNDFVSDADSTLIGDCATVHTANKWDLIISDMYDPRTKHVTKENDSKEGF 6924
TR|Q692E6|Q692E6_CVHSA           PTGTLLVDSDLNDFVSDADSTLIGDCATVHTANKWDLIISDMYDPRTKHVTKENDSKEGF 6924
TR|A0A0K1YZY7|A0A0K1YZY7_CVHSA   PTGTLLVDSDLNDFVSDADSTLIGDCATVHTANKWDLIISDMYDPKTKHVTKENDSKEGF 6924
SP|P0C6W2|R1AB_BCHK3             PTGTLLVDSDLNDFVSDADSTLIGDCATVHTANKWDLIISDMYDPKTKHVLKDNDSKEGF 6918
SP|P0C6W6|R1AB_BCRP3             PTGTLLVDSDLNDFVSDADSTLIGDCATVHTANKWDLIVSDMYDPKAKHVTKENDSKEGF 6922
SP|P0C6V9|R1AB_BC279             PTGALLVDSDLNDFVSDADSTLIGDCATVHTANKWDLIISDMYDPKTKHVTKENDSKEGF 6930
TR|A0A0U1WHI4|A0A0U1WHI4_CVHSA   PIGTLLVDSDLNDFVSDADSTLIGDCATVHTANKWDLIISDMYDPKTKHVTEENDSKEGF 6919
TR|A0A0U1WHG0|A0A0U1WHG0_CVHSA   PIGTLLVDSDLNDFVSDADSTLIGECATVHTANKWDLIVSDMYDPKTKHVTKENDSKEGF 6919
TR|A0A166ZL34|A0A166ZL34_9NIDO   PIGTLLVDSDLNDFVSDADSTLIGECATVHTANKWDLIVSDMYDPKTKHVTKENDSKEGF 6726
TR|R9QTB2|R9QTB2_CVHSA           ------------------------------------------------------------
TR|R9QTH2|R9QTH2_CVHSA           ------------------------------------------------------------
SP|P0C6U8|R1A_CVHSA              ------------------------------------------------------------
TR|Q6JH47|Q6JH47_CVHSA           ------------------------------------------------------------
TR|Q692E5|Q692E5_CVHSA           ------------------------------------------------------------
SP|P0C6F8|R1A_BCHK3              ------------------------------------------------------------
TR|A0A0K1Z0N1|A0A0K1Z0N1_CVHSA   ------------------------------------------------------------
SP|P0C6F5|R1A_BC279              ------------------------------------------------------------
SP|P0C6T7|R1A_BCRP3              ------------------------------------------------------------

QHN73794                         FTYICGFIQQKLALGGSVAIKITEHSWNADLYKLMGHFAWWTAFVTNVNASSSEAFLIGC 7007
SP|P0C6X7|R1AB_CVHSA             FTYLCGFIKQKLALGGSIAVKITEHSWNADLYKLMGHFSWWTAFVTNVNASSSEAFLIGA 6984
TR|Q6UZF5|Q6UZF5_CVHSA           FTYLCGFIKQKLALGGSIAVKITEHSWNADLYKLMGHFSWWTAFVTNVNASSSEAFLIGA 6984
TR|Q6UZF1|Q6UZF1_CVHSA           FTYLCGFIKQKLALGGSIAVKITEHSWNADLYKLMGHFSWWTAFVTNVNASSSEAFLIGA 6984
TR|Q6JH48|Q6JH48_CVHSA           FTYLCGFIKQKLALGGSIAVKITEHSWNADLYKLMGHFSWWTAFVTNVNASSSEAFLIGA 6984
TR|Q692E6|Q692E6_CVHSA           FTYLCGFIKQKLALGGSIAVKITEHSWNADLYKLMGHFSWWTAFVTNVNASSSEAFLIGA 6984
TR|A0A0K1YZY7|A0A0K1YZY7_CVHSA   FTYLCGFIKQKLALGGSAAVKITEHSWNADLYKLMGHFSWWTAFVTNVNASSSEAFLIGV 6984
SP|P0C6W2|R1AB_BCHK3             FTYLCGFIKQKLALGGSVAVKITEHSWNADLYKLMGHFSWWTAFVTNVNASSSEAFLIGV 6978
SP|P0C6W6|R1AB_BCRP3             FTYLCGFIKQKLALGGSVAVKITEHSWNADLYKLMGHFSWWTAFVTNVNASSSEAFLIGV 6982
SP|P0C6V9|R1AB_BC279             FTYLCGFIKQKLALGGSVAVKITEHSWNADLYKLMGHFSWWTAFVTNVNASSSEAFLIGV 6990
TR|A0A0U1WHI4|A0A0U1WHI4_CVHSA   FTYLCGFIKQKLALGGSVAVKITEHSWNADLYKLMGYFSWWTAFVTNVNASSSEAFLIGV 6979
TR|A0A0U1WHG0|A0A0U1WHG0_CVHSA   FTYLCGFIKQKLALGGSVAVKITEHSWNADLYKLMGHFSWWTAFVTNVNASSSEAFLIGV 6979
TR|A0A166ZL34|A0A166ZL34_9NIDO   FTYLCGFIKQKLALGGSVAVKITEHSWNADLYKLMGHFSWWTAFVTNVNASSSEAFLIGV 6786
TR|R9QTB2|R9QTB2_CVHSA           ------------------------------------------------------------
TR|R9QTH2|R9QTH2_CVHSA           ------------------------------------------------------------
SP|P0C6U8|R1A_CVHSA              ------------------------------------------------------------
TR|Q6JH47|Q6JH47_CVHSA           ------------------------------------------------------------
TR|Q692E5|Q692E5_CVHSA           ------------------------------------------------------------
SP|P0C6F8|R1A_BCHK3              ------------------------------------------------------------
TR|A0A0K1Z0N1|A0A0K1Z0N1_CVHSA   ------------------------------------------------------------
SP|P0C6F5|R1A_BC279              ------------------------------------------------------------
SP|P0C6T7|R1A_BCRP3              ------------------------------------------------------------

QHN73794                         NYLGKPREQIDGYVMHANYIFWRNTNPIQLSSYSLFDMSKFPLKLRGTAVMSLKEGQIND 7067
SP|P0C6X7|R1AB_CVHSA             NYLGKPKEQIDGYTMHANYIFWRNTNPIQLSSYSLFDMSKFPLKLRGTAVMSLKENQIND 7044
TR|Q6UZF5|Q6UZF5_CVHSA           NYLGKPKEQIDGYTMHANYIFWRNTNPIQLSSYSLFDMSKFPLKLRGTAVMSLKENQIND 7044
TR|Q6UZF1|Q6UZF1_CVHSA           NYLGKPKEQIDGYTMHANYIFWRNTNPIQLSSYSLFDMSKFPLKLRGTAVMSLKENQIND 7044
TR|Q6JH48|Q6JH48_CVHSA           NYLGKPKEQIDGYTMHANYIFWRNTNPIQLSSYSLFDMSKFPLKLRGTAVMSLKENQIND 7044
TR|Q692E6|Q692E6_CVHSA           NYLGKPKEQIDGYTMHANYIFWRNTNPIQLSSYSLFDMSKFPLKLRGTAVMSLKENQIND 7044
TR|A0A0K1YZY7|A0A0K1YZY7_CVHSA   NYLGKPKEQIDGYTMHANYIFWRNTNPIQLSSYSLFDMSKFPLKLRGTAVMFLKENQIND 7044
SP|P0C6W2|R1AB_BCHK3             NYLGKPKEQIDGYTMHANYIFWRNTNPIQLSSYSLFDMSKFPLKLRGTAVMSLKENQIND 7038
SP|P0C6W6|R1AB_BCRP3             NYLGKPKEQIDGYTMHANYIFWRNTNPIQLSSYSLFDMSKFPLKLRGTAVMSLKENQIND 7042
SP|P0C6V9|R1AB_BC279             NYLGKLREQIDGYTMHANYIFWRNTNPIQLSSYSLFDMSKFPLKLRGTAVMSLKENQIND 7050
TR|A0A0U1WHI4|A0A0U1WHI4_CVHSA   NYLGKPKEQIDGYTMHANYVFWRNTNPIQLSSYSLFDMSKFPLKLRGTAVMSLKENQIND 7039
TR|A0A0U1WHG0|A0A0U1WHG0_CVHSA   NYLGKPKEQIDGYTMHANYIFWRNTNPIQLSSYSLFDMSKFPLKLRGTAVMSLKENQIND 7039
TR|A0A166ZL34|A0A166ZL34_9NIDO   NYLGKPKEQIDGYTMHANYIFWRNTNPIQLSSYSLFDMSKFPLKLRGTAVMSLKENQIND 6846
TR|R9QTB2|R9QTB2_CVHSA           ------------------------------------------------------------
TR|R9QTH2|R9QTH2_CVHSA           ------------------------------------------------------------
SP|P0C6U8|R1A_CVHSA              ------------------------------------------------------------
TR|Q6JH47|Q6JH47_CVHSA           ------------------------------------------------------------
TR|Q692E5|Q692E5_CVHSA           ------------------------------------------------------------
SP|P0C6F8|R1A_BCHK3              ------------------------------------------------------------
TR|A0A0K1Z0N1|A0A0K1Z0N1_CVHSA   ------------------------------------------------------------
SP|P0C6F5|R1A_BC279              ------------------------------------------------------------
SP|P0C6T7|R1A_BCRP3              ------------------------------------------------------------
```

```
QHN73794                       MILSLLSKGRLIIRENNRVVISSDVLVNN 7096
SP|P0C6X7|R1AB_CVHSA           MIYSLLEKGRLIIRENNRVVVSSDILVNN 7073
TR|Q6UZF5|Q6UZF5_CVHSA         MIYSLLEKGRLIIRENNRVVVSSDILVNN 7073
TR|Q6UZF1|Q6UZF1_CVHSA         MIYSLLEKGRLIIRENNRVVVSSDILVNN 7073
TR|Q6JH48|Q6JH48_CVHSA         MIYSLLEKGRLIIRENNRVVVSSDILVNN 7073
TR|Q692E6|Q692E6_CVHSA         MIYSLLEKGRLIIRENNRVVVSSDILVNN 7073
TR|A0A0K1YZY7|A0A0K1YZY7_CVHSA MIYSLLEKGRLIIRENNTVVVSSDVLVNH 7073
SP|P0C6W2|R1AB_BCHK3           MIYSLLEKGRLIIRENNRVVVSSDILVNN 7067
SP|P0C6W6|R1AB_BCRP3           MIYSLLEKGRLIIRENNRVVVSSDILVNN 7071
SP|P0C6V9|R1AB_BC279           MIYSLLENGRLIIRENNRVVVSSDILVNN 7079
TR|A0A0U1WHI4|A0A0U1WHI4_CVHSA MIYSLLEKGRLIIRENNTVVVSSDVLVNH 7068
TR|A0A0U1WHG0|A0A0U1WHG0_CVHSA MIYSLLEKGRLIVRENNRVIVSSDVLVNN 7068
TR|A0A166ZL34|A0A166ZL34_9NIDO MIYSLLEKGRLIVRENNRVIVSSDVLVNN 6875
TR|R9QTB2|R9QTB2_CVHSA         -----------------------------
TR|R9QTH2|R9QTH2_CVHSA         -----------------------------
SP|P0C6U8|R1A_CVHSA            -----------------------------
TR|Q6JH47|Q6JH47_CVHSA         -----------------------------
TR|Q692E5|Q692E5_CVHSA         -----------------------------
SP|P0C6F8|R1A_BCHK3            -----------------------------
TR|A0A0K1Z0N1|A0A0K1Z0N1_CVHSA -----------------------------
SP|P0C6F5|R1A_BC279            -----------------------------
SP|P0C6T7|R1A_BCRP3            -----------------------------
```
