# Supplementary material for: Structural Genomics of SARS-CoV-2 Indicates Evolutionary Conserved Functional Regions of Viral Proteins
Source: Viruses. 2020 Mar 25;12(4):360. doi: 10.3390/v12040360 (PMC7232164; doi:10.3390/v12040360)
Supplement: Supplementary file 1 [file viruses-12-00360-s001.zip › wS_human_PBS_mapped.pdf]

Supplementary File 10. Intra-viral protein binding sites and a homo-oligomer binding site mapped on the sequences of wS protein aligned with the closest homologs found in UniProt.

<u>Homo-oligomer Protein Binding Sites:</u>

wS_homotrimer-Conf1-5X58

<u>Host-Virus Interaction Protein Binding Sites</u>:

wS_heterodimer-IGHV3-30-3-2GHW
wS_trimer-ACE2-Conf1-6ACG
wS_trimer-ACE2-Conf2-6ACJ
wS_trimer-ACE2-Conf3-6ACK
wS_trimer-IGHV3-30-3-Conf1-6NB6
wS_trimer-IGHV3-30-3-Conf2-6NB7

```
YP_009724390                   MFV-------FLVLLPLVSSQCVNLTTRTQLPPAYTNSFTRGVYYPDKVFRSSVLHSTQD 53
SP|P59594|SPIKE_CVHSA          MFI--FLL-FLTLTSGSDLDRCTTFDDVQAPNYTQHTSSMRGVYYPDEIFRSDTLYLTQD 57
TR|Q6UZF4|Q6UZF4_CVHSA         MFI--FLL-FLTLTSGSDLDRCTTFDDVQAPNYTQHTSSMRGVYYPDEIFRSDTLYLTQD 57
TR|Q6UZF0|Q6UZF0_CVHSA         MFI--FLL-FLTLTSGSDLDRCTTFDDVQAPNYTQHTSSMRGVYYPDEIFRSDTLYLTQD 57
TR|Q6JH46|Q6JH46_CVHSA         MFI--FLL-FLTLTSGSDLDRCTTFDDVQAPNYTQHTSSMRGVYYPDEIFRSDTLYLTQD 57
TR|Q692E4|Q692E4_CVHSA         MFI--FLL-FLTLTSGSDLDRCTTFDDVQAPNYTQHTSSMRGVYYPDEIFRSDTLYLTQD 57
SP|Q3LZX1|SPIKE_BCHK3          MKILIF---AFLANLAKAQEGCGIISRKPQPKMAQVSSSRRGVYYNDDIFRSDVLHLTQD 57
SP|Q3I5J5|SPIKE_BCRP3          MKILIL---AFLASLAKAQEGCGIISRKPQPKMAQVSSSRRGVYYNDDIFRSNVLHLTQD 57
SP|Q0Q475|SPIKE_BC279          MKVLIF---ALLFSLAKAQEGCGIISRKPQPKMEKVSSSRRGVYYNDDIFRSDVLHLTQD 57
TR|R9QTA0|R9QTA0_CVHSA         --MILL---LLFLSSAKAQEGCGVISNKPQRTFDQYSSTFRGVYYNDDIFRSDVLHLTQD 55
TR|R9QTH3|R9QTH3_CVHSA         MKIFLF---SLLFSAALAQEGCGLLSFKPQPKLAQFSSSKRGVYYNDDIFRSDVLHLTQD 57
TR|A0A0K1Z074|A0A0K1Z074_CVHSA MKILIF---AFLVTLVKAQEGCGVINLRTQPKLSQVSSSRRGVYYNDDIFRSDVLHLTQD 57
TR|A0A0U1WHI6|A0A0U1WHI6_CVHSA MKILIF---AFLVTLVKAQEGCGVINLKTQPTLTQVSSSRRGVYYNDDIFRSDVLHLTQD 57
TR|A0A166ZL64|A0A166ZL64_9NIDO MFIVFLISYTTFLIPYTATTTC--FSGPTIENKLNISSGSRGVYYPDDIFRSNVSVLVTG 58
TR|A0A0U1UYX4|A0A0U1UYX4_CVHSA MFIVFLISYTTFLIPYTATTTC--FKGPTTENKLNISSGSRGVYYPDDIFRSNVSVLVTG 58
                                 :                  *  :           .*  ***** *.:***..   . .

YP_009724390                   LFLPFFSNVTWFHAIHVSGTNGTKRFDNPVLPFNDGVYFASTEKSNIIRGWIFGTTLDSK 113
SP|P59594|SPIKE_CVHSA          LFLPFYSNVTGFHTINH-------TFGNPVIPFKDGIYFAATEKSNVVRGWVFGSTMNNK 110
TR|Q6UZF4|Q6UZF4_CVHSA         LFLPFYSNVTGFHTINH-------TFGNPVIPFKDGIYFAATEKSNVVRGWVFGSTMNNK 110
TR|Q6UZF0|Q6UZF0_CVHSA         LFLPFYSNVTGFHTINH-------TFGNPVIPFKDGIYFAATEKSNVVRGWVFGSTMNNK 110
TR|Q6JH46|Q6JH46_CVHSA         LFLPFYSNVTGFHTINH-------TFGNPVIPFKDGIYFAATEKSNVVRGWVFGSTMNNK 110
TR|Q692E4|Q692E4_CVHSA         LFLPFYSNVTGFHTINH-------TFGNPVIPFKDGIYFAATEKSNVVRGWVFGSTMNNK 110
SP|Q3LZX1|SPIKE_BCHK3          YFLPFDSNLTQYFSLNVDS-DRYTYFDNPILDFGDGVYFAATEKSNVIRGWIFGSSFDNT 116
SP|Q3I5J5|SPIKE_BCRP3          YFLPFDSNLTQYFSLNVDS-DRFTYFDNPILDFGDGVYFAATEKSNVIRGWIFGSTFDNT 116
SP|Q0Q475|SPIKE_BC279          YFLPFDSNLTQYFSLNIDS-NKYTYFDNPILDFGDGVYFAATEKSNVIRGWIFGSSFDNT 116
TR|R9QTA0|R9QTA0_CVHSA         YFLPFNTNVTRYLSLNAAQ-NTIVYFDNHVIPFYDGIYFAATERSNVIRGWIFGSTFDNR 114
TR|R9QTH3|R9QTH3_CVHSA         YFLPFHSNLTQYFSLNVDS-DRQVYFDNPTLNFGDGVYFAATEKSNVIRGWIFGSTMDNS 116
TR|A0A0K1Z074|A0A0K1Z074_CVHSA YFLPFHSNLTQYFSLSIQS-DKIVYFDNPILKFGDGIYFAATEKSNVIRGWVFGSTFDNT 116
TR|A0A0U1WHI6|A0A0U1WHI6_CVHSA YFLPFHSNLTQYFSLNIES-D--IYFDNPILKFGDGVYFAATEKSNVIRGWVFGSTFDNT 114
TR|A0A166ZL64|A0A166ZL64_9NIDO RFLRFNTTLTWYNSWNQ-------AYSSPVLPFGHGVYFSTIDKSNVVRGWIFGTTLDNT 111
TR|A0A0U1UYX4|A0A0U1UYX4_CVHSA RFLRFNTTLTWYNSWNQ-------AYSSPVLPFGHGVYFSTIDKSNVVRGWIFGTTLDNT 111
                                ** * :.:* : :           :..  : * .*:**:: ::**::***:**::::.

YP_009724390                   TQSLLIVNNATNVVIKVCEFQFCNDPFLGVYYHKNNKSWMESEFRVYSSANNCTFEYVSQ 173
SP|P59594|SPIKE_CVHSA          SQSVIIINNSTNVVIRACNFELCDNPFFAVSKPMG----TQTHTMIFDNAFNCTFEYISD 166
TR|Q6UZF4|Q6UZF4_CVHSA         SQSVIIINNSTNVVIRACNFELCDNPFFAVSKPMG----TQTHTMIFDNAFNCTFEYISD 166
TR|Q6UZF0|Q6UZF0_CVHSA         SQSVIIINNSTNVVIRACNFELCDNPFFAVSKPMG----TQTHTMIFDNAFNCTFEYISD 166
TR|Q6JH46|Q6JH46_CVHSA         SQSVIIINNSTNVVIRACNFELCDNPFFAVSKPMG----TQTHTMIFDNAFNCTFEYISD 166
TR|Q692E4|Q692E4_CVHSA         SQSVIIINNSTNVVIRACNFELCDNPFFAVSKPMG----TQTHTMIFDNAFNCTFEYISD 166
SP|Q3LZX1|SPIKE_BCHK3          TQSAVIVNNSTHIIIRVCNFNLCKEPMYTVSR--G----TQQNAWVYQSAFNCTYDRVEK 170
SP|Q3I5J5|SPIKE_BCRP3          TQSAVIVNNSTHIIIRVCNFNLCKEPMYTVSR--G----AQQSSWVYQSAFNCTYDRVEK 170
SP|Q0Q475|SPIKE_BC279          TQSAIIVNNSTHIIIRVCNFNLCKEPMYTVSK--G----TQQSSWVYQSAFNCTYDRVEK 170
TR|R9QTA0|R9QTA0_CVHSA         SQSAIIVNNSTHILVKVCNFVLCTEPMFTVSR--N----QHYKSWVYQHARNCTYDVAYP 168
TR|R9QTH3|R9QTH3_CVHSA         TQSAIIVNNSTHIIIRVCNFNLCKEPMFTVSR--G----VHFSSWVYQSAFNCTYDRVEK 170
TR|A0A0K1Z074|A0A0K1Z074_CVHSA TQSAIIVNNSTHIIIRVCYFNLCKDPMYTVSA--G----TQISSWVYQNAFNCTYDRVEK 170
TR|A0A0U1WHI6|A0A0U1WHI6_CVHSA TQSAIIVNNSTHIIIRVCYFNLCKDPMYTVSA--G----TQVSSWVYQSAFNCTYDRVEK 168
TR|A0A166ZL64|A0A166ZL64_9NIDO TQSALLVNNGSAITIEVCYFQFCDNPAFIISG--G----AQTNTAIYINLRNCTYVDTLR 165
TR|A0A0U1UYX4|A0A0U1UYX4_CVHSA TQSALLVNNGSAITIEVCYFQFCDNPAFIIRD--G----AQINTAIYINLRNCTYVDTLR 165
                               :** :::**.: : :..* * :* :*   :    .     .    ::    ***:
```

```
YP_009724390                      PFLMDLEGK-QGNFKNLREFVFKNIDGYFKIYSKHTPINLVR----DLPQGFSALEPLVD 228
SP|P59594|SPIKE_CVHSA             AFSLDVSEK-SGNFKHLREFVFKNKDGFLYVYKGYQPIDVVR----DLPSGFNTLKPIFK 221
TR|Q6UZF4|Q6UZF4_CVHSA            AFSLDVSEK-SGNFKHLREFVFKNKDGFLYVYKGYQPIDVVR----DLPSGFNTLKPIFK 221
TR|Q6UZF0|Q6UZF0_CVHSA            AFSLDVSEK-SGNFKHLREFVFKNKDGFLYVYKGYQPIDVVR----DLPSGFNTLKPIFK 221
TR|Q6JH46|Q6JH46_CVHSA            AFSLDVSEK-SGNFKHLREFVFKNKDGFLYVYKGYQPIDVVR----DLPSGFNTLKPIFK 221
TR|Q692E4|Q692E4_CVHSA            AFSLDVSEK-SGNFKHLREFVFKNKDGFLYVYKGYQPIDVVR----DLPSGFNTLKPIFK 221
SP|Q3LZX1|SPIKE_BCHK3             SFQLDTTPK-TGNFKDLREYVFKNRDGFLSVYQTYTAVNLPR----GLPTGFSVLKPILK 225
SP|Q3I5J5|SPIKE_BCRP3             SFQLDTAPK-TGNFKDLREYVFKNRDGFLSVYQTYTAVNLPR----GLPIGFSVLRPILK 225
SP|Q0Q475|SPIKE_BC279             SFQLDTAPK-TGNFKDLREYVFKNRDGFLSVYQTYTAVNLPR----GFPAGFSVLRPILK 225
TR|R9QTA0|R9QTA0_CVHSA            SFQLDVSLKNNVNFQHLREFIFKNVDGFLKIYSSYEPINVVS----GIPSGFSVLKPVMS 224
TR|R9QTH3|R9QTH3_CVHSA            SFQLDTAPK-TGNFKDLREYVFKNRDGFLSVYHSYTPVDIIR----GIPVGFSVLKPILK 225
TR|A0A0K1Z074|A0A0K1Z074_CVHSA    SFQLDTAPK-SGNFIALREFVFKNRDGFFTVYQDYTPVNLLR----GLPAGLSVLKPILK 225
TR|A0A0U1WHI6|A0A0U1WHI6_CVHSA    SFQLDTSPK-TGNFTDLREFVFKNRDGFFTVYQTYTPVNLLR----GLPSGLSVLKPILK 223
TR|A0A166ZL64|A0A166ZL64_9NIDO    DLPLSFED-VDGGFKHLREFVFKNSDGFLHIYGAYQPYDLAIGATAALPAQFLPLKPLWK 224
TR|A0A0U1UYX4|A0A0U1UYX4_CVHSA    DLPLSFAE-VDGGFKHLREFVFKNSDGFLHIYGAYQPYDLAIGATAALPAQFLPLKPLWK 224
                                   : :.        .*  ***::*** **:: :*   :    ::        :*  :   *.*: .

YP_009724390                      LPIGINITRFQTLLALHRSYLTPGDSSSGWTAGAAAYYVGYLQPRTFLLKYNENGTITDA 288
SP|P59594|SPIKE_CVHSA             LPLGINITNFRAILTAFS------PAQDIWGTSAAAYFVGYLKPTTFMLKYDENGTITDA 275
TR|Q6UZF4|Q6UZF4_CVHSA            LPLGINITNFRAILTAFS------PAQDIWGTSAAAYFVGYLKPTTFMLKYDENGTITDA 275
TR|Q6UZF0|Q6UZF0_CVHSA            LPLGINITNFRAILTAFS------PAQDIWGTSAAAYFVGYLKPTTFMLKYDENGTITDA 275
TR|Q6JH46|Q6JH46_CVHSA            LPLGINITNFRAILTAFS------PAQDIWGTSAAAYFVGYLKPTTFMLKYDENGTITDA 275
TR|Q692E4|Q692E4_CVHSA            LPLGINITNFRAILTAFS------PAQDTWGTSAAAYFVGYLKPTTFMLKYDENGTITDA 275
SP|Q3LZX1|SPIKE_BCHK3             LPFGINITSYRVVMAMFS------QTTSNFLPESAAYYVGNLKYSTFMLRFNENGTITDA 279
SP|Q3I5J5|SPIKE_BCRP3             LPFGINITSYRVVMAMFS------QTTSNFLPESAAYYVGNLKYTTFMLSFNENGTITNA 279
SP|Q0Q475|SPIKE_BC279             LPFGINITSYRVVMTMFS------QFNSNFLPESAAYYVGNLKYTTFMLSFNENGTITDA 279
TR|R9QTA0|R9QTA0_CVHSA            LPLGINITGMRVVMTMFS------NTQANFLTENAAYYVGYLKPRTFMLQFNTNGTIVNA 278
TR|R9QTH3|R9QTH3_CVHSA            LPIGINITSFKVVMTMYS------QTTSNFLSESAAYYVGNLKYVTFMFQFNENGTIADA 279
TR|A0A0K1Z074|A0A0K1Z074_CVHSA    LPFGINITSFRVVMAMFS------KTTSNYVPESAAYYVGNLKQSTFMLSFNQNGTITDA 279
TR|A0A0U1WHI6|A0A0U1WHI6_CVHSA    LPFGINITSFRVVMAMFS------KTTSNYVPESAAYYVGNLKQSTFMLSFNQNGTIVDA 277
TR|A0A166ZL64|A0A166ZL64_9NIDO    LPLGLNITNYKVVTTLKP------TNQ----AFQAAYIVGNLKHTTMMLSFNENGTMSNA 274
TR|A0A0U1UYX4|A0A0U1UYX4_CVHSA    LPLGLNITNYKVVTTLKP------TNQ----AFQAAYIVGNLKHTTMMLSFNENGTMSNA 274
                                  **:*:***  :.: :                     *** ** *:  *::: :: ***: :*

YP_009724390                      VDCALDPLSETKCTLKSFTVEKGIYQTSNFRVQPTESIVRFPNITNLCPFGEVFNATRFA 348
SP|P59594|SPIKE_CVHSA             VDCSQNPLAELKCSVKSFEIDKGIYQTSNFRVVPSGDVVRFPNITNLCPFGEVFNATKFP 335
TR|Q6UZF4|Q6UZF4_CVHSA            VDCSQNPLAELKCSVKSFEIDKGIYQTSNFRVVPSGDVVRFPNITNLCPFGEVFNATKFP 335
TR|Q6UZF0|Q6UZF0_CVHSA            VDCSQNPLAELKCSVKSFEIDKGIYQTSNFRVVPSGDVVRFPNITNLCPFGEVFNATKFP 335
TR|Q6JH46|Q6JH46_CVHSA            VDCSQNPLAELKCSVKSFEIDKGIYQTSNFRVVPSGDVVRFPNITNLCPFGEVFNATKFP 335
TR|Q692E4|Q692E4_CVHSA            VDCSQNPLAELKCSVKSFEIDKGIYQTSNFRVVPSGDVVRFPNITNLCPFGEVFNATKFP 335
SP|Q3LZX1|SPIKE_BCHK3             VDCSQNPLAELKCTIKNFNVDKGIYQTSNFRVSPTQEVIRFPNITNRCPFDKVFNATRFP 339
SP|Q3I5J5|SPIKE_BCRP3             IDCAQNPLAELKCTIKNFNVSKGIYQTSNFRVSPTQEVIRFPNITNRCPFDKVFNATRFP 339
SP|Q0Q475|SPIKE_BC279             VDCSQNPLAELKCTIKNFNVSKGIYQTSNFRVTPTQEVVRFPNITNRCPFDKVFNASRFP 339
TR|R9QTA0|R9QTA0_CVHSA            VDCSQDPLSELKCTLKNFNITKGIYQTSNFRVSPTQEVVRFPNITNRCPFDKVFNATRFP 338
TR|R9QTH3|R9QTH3_CVHSA            VDCSQNPLAELKCTLKNFNVSKGIYQTSNFRVSPSTEVIRFPNITNRCPFDRVFNASRFP 339
TR|A0A0K1Z074|A0A0K1Z074_CVHSA    VDCSQNPLAELKCTTKSFNVSKGIYQTSNFRVAPVTEVVRFPNITNLCPFDKVFNATRFP 339
TR|A0A0U1WHI6|A0A0U1WHI6_CVHSA    VDCSQDPLAELKCTTKSFNVSKGIYQTSNFRVSPVTEVVRFPNITNLCPFDKVFNATRFP 337
TR|A0A166ZL64|A0A166ZL64_9NIDO    IDCSQDPLAELKCTLKQFDVGKGIYQTSNFRVQPTVDVARFPNITNVCPFDKVFNATRFP 334
TR|A0A0U1UYX4|A0A0U1UYX4_CVHSA    IDCSQDPLAELKCTLKQFDVGKGIYQTSNFRVQPTVDVARFPNITNVCPFDKVFNATRFP 334
                                  :**: :**:* **: *.* : *********** *  .: ******* ***..****::*

YP_009724390                      SVYAWNRKRISNCVADYSVLYNSASFSTFKCYGVSPTKLNDLCFTNVYADSFVIRGDEVR 408
SP|P59594|SPIKE_CVHSA             SVYAWERKKISNCVADYSVLYNSTFFSTFKCYGVSATKLNDLCFSNVYADSFVVKGDDVR 395
TR|Q6UZF4|Q6UZF4_CVHSA            SVYAWERKKISNCVADYSVLYNSTFFSTFKCYGVSATKLNDLCFSNVYADSFVVKGDDVR 395
TR|Q6UZF0|Q6UZF0_CVHSA            SVYAWERKKISNCVADYSVLYNSTFFSTFKCYGVSATKLNDLCFSNVYADSFVVKGDDVR 395
TR|Q6JH46|Q6JH46_CVHSA            SVYAWERKKISNCVADYSVLYNSTFFSTFKCYGVSATKLNDLCFSNVYADSFVVKGDDVR 395
TR|Q692E4|Q692E4_CVHSA            SVYAWERKKISNCVADYSVLYNSTFFSTFKCYGVSATKLNDLCFSNVYADSFVVKGDDVR 395
SP|Q3LZX1|SPIKE_BCHK3             NVYAWERTKISDCVADYTVLYNSTSFSTFKCYGVSPSKLIDLCFTSVYADTFLIRSSEVR 399
SP|Q3I5J5|SPIKE_BCRP3             NVYAWERTKISDCVADYTVLYNSTSFSTFKCYGVSPSKLIDLCFTSVYADTFLIRSSEVR 399
SP|Q0Q475|SPIKE_BC279             NVYAWERTKISDCVADYTVLYNSTSFSTFKCYGVSPSKLIDLCFTSVYADTFLIRSSEVR 399
TR|R9QTA0|R9QTA0_CVHSA            SVYAWERTKISDCVADYTVLYNSTSFSTFKCYGVSPSKLIDLCFTSVYADTFLIRSSEVR 398
TR|R9QTH3|R9QTH3_CVHSA            SVYAWERTKISDCVADYTVLYNSTSFSTFKCYGVSPSKLIDLCFTSVYADTFLIRFSEVR 399
TR|A0A0K1Z074|A0A0K1Z074_CVHSA    SVYAWERTKISDCVADYTVFYNSTSFSTFNCYGVSPSKLIDLCFTSVYADTFLIRFSEVR 399
TR|A0A0U1WHI6|A0A0U1WHI6_CVHSA    SVYAWERTKISDCVADYTVFYNSTSFSTFNCYGVSPSKLIDLCFTSVYADTFLIRFSEVR 397
TR|A0A166ZL64|A0A166ZL64_9NIDO    SVYAWERTKISDCVADYTVFYNSTSFSTFNCYGVSPSKLIDLCFTSVYADTFLIRFSEVR 394
TR|A0A0U1UYX4|A0A0U1UYX4_CVHSA    SVYAWERTKISDCVADYTVFYNSTSFSTFNCYGVSPSKLIDLCFTSVYADTFLIRFSEVR 394
                                  .****:*.:**:*****:*:***: ****:***** :** ****:.****:*::: .:**
```

```
YP_009724390                   QIAPGQTGKIADYNYKLPDDFTGCVIAWNSNNLDSKVGGNYNYLYRLFRKSNLKPFERDI 468
SP|P59594|SPIKE_CVHSA          QIAPGQTGVIADYNYKLPDDFMGCVLAWNTRNIDATSTGNYNYKYRYLRHGKLRPFERDI 455
TR|Q6UZF4|Q6UZF4_CVHSA         QIAPGQTGVIADYNYKLPDDFMGCVLAWNTRNIDATSTGNYNYKYRYLRHGKLRPFERDI 455
TR|Q6UZF0|Q6UZF0_CVHSA         QIAPGQTGVIADYNYKLPDDFMGCVLAWNTRNIDATSTGNYNYKYRYLRHGKLRPFERDI 455
TR|Q6JH46|Q6JH46_CVHSA         QIAPGQTGVIADYNYKLPDDFMGCVLAWNTRNIDATSTGNYNYKYRSLRHGKLRPFERDI 455
TR|Q692E4|Q692E4_CVHSA         QIAPGQTGVIADYNYKLPDDFMGCVLAWNTRNIDATSTGNYNYKYRYLRHGKLRPFERDI 455
SP|Q3LZX1|SPIKE_BCHK3          QVAPGETGVIADYNYKLPDDFTGCVIAWNTAKHDTG-----NYYYRSHRKTKLKPFERDL 454
SP|Q3I5J5|SPIKE_BCRP3          QVAPGETGVIADYNYKLPDDFTGCVIAWNTAKQDQG-----QYYYRSHRKTKLKPFERDL 454
SP|Q0Q475|SPIKE_BC279          QVAPGETGVIADYNYKLPDDFTGCVIAWNTAQQDQG-----QYYYRSYRKEKLKPFERDL 454
TR|R9QTA0|R9QTA0_CVHSA         QVAPGETGVIADYNYKLPDDFTGCVIAWNTANQDQG-----QYYYRSSRKEKLKPFERDL 453
TR|R9QTH3|R9QTH3_CVHSA         QIAPGETGVIADYNYKLPDEFTGCVIAWNTANQDRG-----QYYYRSSRKTKLKPFERDL 454
TR|A0A0K1Z074|A0A0K1Z074_CVHSA QVAPGQTGVIADYNYKLPDDFTGCVIAWNTAKYDVG-----SYFYRSHRSSKLKPFERDL 454
TR|A0A0U1WHI6|A0A0U1WHI6_CVHSA QVAPGQTGVIADYNYKLPDDFTGCVIAWNTAKQDVG-----SYFYRSHRSSKLKPFERDL 452
TR|A0A166ZL64|A0A166ZL64_9NIDO QVAPGQTGVIADYNYKLPDDFIGCVIAWNTAKQDVG-----SYFYRSHRSSKLKPFERDL 449
TR|A0A0U1UYX4|A0A0U1UYX4_CVHSA QVAPGQTGVIADYNYKLPDDFIGCVIAWNTAKQDVG-----SYFYRSHRSSKLKPFERDL 449
                               *:***:** **********:* ***:***: : *       .* **  *  :*:*****:

YP_009724390                   STEIYQAGSTPCNGVEGFNCYFPLQSYGFQPTNGVGYQPYRVVVLSFELLHAPATVCGPK 528
SP|P59594|SPIKE_CVHSA          SNVPFSPDGKPCTPP-ALNCYWPLNDYGFYTTTGIGYQPYRVVVLSFELLNAPATVCGPK 514
TR|Q6UZF4|Q6UZF4_CVHSA         SNVPFSPDGKPCTPP-ALNCYWPLNDYGFYTTTGIGYQPYRVVVLSFELLNAPATVCGPK 514
TR|Q6UZF0|Q6UZF0_CVHSA         SNVPFSPDGKPCTPP-ALNCYWPLNDYGFYTTTGIGYQPYRVVVLSFELLNAPATVCGPK 514
TR|Q6JH46|Q6JH46_CVHSA         SNVPFSPDGKPCTPP-ALNCYWPLNDYGFYTTTGIGYQPYRVVVLSFELLNAPATVCGPK 514
TR|Q692E4|Q692E4_CVHSA         SNVPFSPDGKPCTPP-ALNCYWPLNDYGFYTTTGIGYQPYRVVVLSFELLNAPATVCGPK 514
SP|Q3LZX1|SPIKE_BCHK3          SSDD-------------GNGVYTLSTYDFNPNVPVAYQATRVVVLSFELLNAPATVCGPK 501
SP|Q3I5J5|SPIKE_BCRP3          SSD--------------ENGVRTLSTYDFYPSVPVAYQATRVVVLSFELLNAPATVCGPK 500
SP|Q0Q475|SPIKE_BC279          SSD--------------ENGVYTLSTYDFYPSIPVEYQATRVVVLSFELLNAPATVCGPK 500
TR|R9QTA0|R9QTA0_CVHSA         SSD--------------ENGVYTLSTYDFYPSVPLDYQATRVVVLSFELLNAPATVCGPK 499
TR|R9QTH3|R9QTH3_CVHSA         SSD--------------ENGVRTLSTYDFYPSVPLEYQATRVVVLSFELLNAPATVCGPK 500
TR|A0A0K1Z074|A0A0K1Z074_CVHSA SSE--------------ENGARTLSTYDFNQNVPLEYQATRVVVLSFELLNAPATVCGPK 500
TR|A0A0U1WHI6|A0A0U1WHI6_CVHSA SSE--------------ENGVRTLSTYDFNQYVPLEYQATRVVVLSFELLNAPATVCGPK 498
TR|A0A166ZL64|A0A166ZL64_9NIDO SSE--------------ENGVLTLSTYDFNQNVPLEYQATRVVVLSFELLNAPATVCGPK 495
TR|A0A0U1UYX4|A0A0U1UYX4_CVHSA SSE--------------ENGVLTLSTYDFNQNVPLEYQATRVVVLSFELLNAPATVCGPK 495
                               *.                *    *. *.*     : **  **********:*********

YP_009724390                   KSTNLVKNKCVNFNFNGLTGTGVLTESNKKFLPFQQFGRDIADTTDAVRDPQTLEILDIT 588
SP|P59594|SPIKE_CVHSA          LSTDLIKNQCVNFNFNGLTGTGVLTPSSKRFQPFQQFGRDVSDFTDSVRDPKTSEILDIS 574
TR|Q6UZF4|Q6UZF4_CVHSA         LSTDLIKNQCVNFNFNGLTGTGVLTPSSKRFQPFQQFGRDVSDFTDSVRDPKTSEILDIS 574
TR|Q6UZF0|Q6UZF0_CVHSA         LSTDLIKNQCVNFNFNGLTGTGVLTPSSKRFQPFQQFGRDVSDFTDSVRDPKTSEILDIS 574
TR|Q6JH46|Q6JH46_CVHSA         LSTDLIKNQCVNFNFNGLTGTGVLTPSSKRFQPFQQFGRDVSDFTDSVRDPKTSEILDIS 574
TR|Q692E4|Q692E4_CVHSA         LSTDLIKNQCVNFNFNGLTGTGVLTPSSKRFQPFQQFGRDVSDFTDSVRDPKTSEILDIS 574
SP|Q3LZX1|SPIKE_BCHK3          LSTELVKNQCVNFNFNGLKGTGVLTSSSKRFQSFQQFGRDTSDFTDSVRDPQTLEILDIS 561
SP|Q3I5J5|SPIKE_BCRP3          LSTQLVKNQCVNFNFNGLKGTGVLTESSKRFQSFQQFGRDTSDFTDSVRDPQTLEILDIS 560
SP|Q0Q475|SPIKE_BC279          LSTQLVKNQCVNFNFNGLRGTGVLTTSSKRFQSFQQFGRDTSDFTDSVRDPQTLEILDIS 560
TR|R9QTA0|R9QTA0_CVHSA         LSTTLVKNQCVNFNFNGLKGTGVLTASSKKFQSFQQFGRDASDFTDSVRDPQTLEILDIS 559
TR|R9QTH3|R9QTH3_CVHSA         LSTSLIKNQCVNFNFNGLKGTGVLTDSSKKFQSFQQFGRDASDFTDSVRDPQTLQILDIS 560
TR|A0A0K1Z074|A0A0K1Z074_CVHSA LSTSLVKNQCVNFNFNGFKGTGVLTDSSKTFQSFQQFGRDASDFTDSVRDPKTLQILDIS 560
TR|A0A0U1WHI6|A0A0U1WHI6_CVHSA LSTSLVKNQCVNFNFNGFKGTGVLTDSSKTFQSFQQFGRDASDFTDSVRDPQTLRILDIS 558
TR|A0A166ZL64|A0A166ZL64_9NIDO LSTPLVKNQCVNFNFNGLKGTGVLTDSSKTFQSFQQFGRDASDFTDSVRDPQTLQILDIS 555
TR|A0A0U1UYX4|A0A0U1UYX4_CVHSA LSTPLVKNQCVNFNFNGLKGTGVLTDSSKTFQSFQQFGRDASDFTDSVRDPQTLQILDIS 555
                                ** *:**:********: ****** *.* *  ******* :* **:****:* .****:
```

```
YP_009724390                       PCSFGGVSVITPGTNTSNQVAVLYQDVNCTEVPVAIHADQLTPTWRVYSTGSNVFQTRAG 648
SP|P59594|SPIKE_CVHSA              PCSFGGVSVITPGTNASSEVAVLYQDVNCTDVSTAIHADQLTPAWRIYSTGNNVFQTQAG 634
TR|Q6UZF4|Q6UZF4_CVHSA             PCSFGGVSVITPGTNASSEVAVLYQDVNCTDVSTAIHADQLTPAWRIYSTGNNVFQTQAG 634
TR|Q6UZF0|Q6UZF0_CVHSA             PCSFGGVSVITPGTNASSEVAVLYQDVNCTDVSTAIHADQLTPAWRIYSTGNNVFQTQAG 634
TR|Q6JH46|Q6JH46_CVHSA             PCSFGGVSVITPGTNASSEVAVLYQDVNCTDVSTAIHADQLTPAWRIYSTGNNVFQTQAG 634
TR|Q692E4|Q692E4_CVHSA             PCSFGGVSVITPGTNASSEVAVLYQDVNCTDVSTAIHADQLTPAWRIYSTGNNVFQTQAG 634
SP|Q3LZX1|SPIKE_BCHK3              PCSFGGVSVITPGTNASSEVAVLYQDVNCTDVPTAIRADQLTPAWRVYSTGVNVFQTQAG 621
SP|Q3I5J5|SPIKE_BCRP3              PCSFGGVSVITPGTNASSEVAVLYQDVNCTDVPAAIHADQLTPAWRVYSTGTNVFQTQAG 620
SP|Q0Q475|SPIKE_BC279              PCSFGGVSVITPGTNASSEVAVLYQDVNCTDVPTSIHADQLTPAWRVYSTGVNVFQTQAG 620
TR|R9QTA0|R9QTA0_CVHSA             PCSFGGVSVITPGTNASTEVAVLYQDVNCTDVPTAINADQLTPAWRVYSTGINVFQTQAG 619
TR|R9QTH3|R9QTH3_CVHSA             PCSFGGVSVITPGTNASSEVAVLYQDVNCTDVPTAIRADQLTPAWRVYSAGVNVFQTQAG 620
TR|A0A0K1Z074|A0A0K1Z074_CVHSA     PCSFGGVSVITPGTNTSSAVAVLYQDVNCTDVPKTIHADQLAPSWRVYTSGPFVFQTQAG 620
TR|A0A0U1WHI6|A0A0U1WHI6_CVHSA     PCSFGGVSVITPGTNTSSAVAVLYQDVNCTDVPTTLHADQLAPSWRVYTTGPYVFQTQAG 618
TR|A0A166ZL64|A0A166ZL64_9NIDO     PCSFGGVSVITPGTNTSSAVAVLYQDVNCTDVPTTIHADHLTHSWRVYTTGPYVFQTQAG 615
TR|A0A0U1UYX4|A0A0U1UYX4_CVHSA     PCSFGGVSVITPGTNTSSAVAVLYQDVNCTDVPTTIHADHLTHSWRVYTTGPYVFQTQAG 615
                                   ***************:*. ***********:*  ::.**:*: :**:*::*  ****:**

YP_009724390                       CLIGAEHVNNSYECDIPIGAGICASYQTQTNSPRRARSVASQSIIAYTMSLGAENSVAYS 708
SP|P59594|SPIKE_CVHSA              CLIGAEHVDTSYECDIPIGAGICASYHTVS----LLRSTSQKSIVAYTMSLGADSSIAYS 690
TR|Q6UZF4|Q6UZF4_CVHSA             CLIGAEHVDTSYECDIPIGAGICASYHTVS----LLRSTSQKSIVAYTMSLGADSSIAYS 690
TR|Q6UZF0|Q6UZF0_CVHSA             CLIGAEHVDTSYECDIPIGAGICASYHTVS----LLRSTSQKSIVAYTMSLGADSSIAYS 690
TR|Q6JH46|Q6JH46_CVHSA             CLIGAEHVDTSYECDIPIGAGICASYHTVS----LLRSTSQKSIVAYTMSLGADSSIAYS 690
TR|Q692E4|Q692E4_CVHSA             CLIGAEHVDTSYECDIPIGAGICASYHTVS----LLRNTSQKSIVAYTMSLGADSSIAYS 690
SP|Q3LZX1|SPIKE_BCHK3              CLIGAEHVNASYECDIPIGAGICASYHTAS----VLRSTGQKSIVAYTMSLGAENSIAYA 677
SP|Q3I5J5|SPIKE_BCRP3              CLIGAEHVNASYECDIPIGAGICASYHTAS----TLRSVGQKSIVAYTMSLGAENSIAYA 676
SP|Q0Q475|SPIKE_BC279              CLIGAEHVNASYECDIPIGAGICASYHTAS----VLRSTGQKSIVAYTMSLGAENSIAYA 676
TR|R9QTA0|R9QTA0_CVHSA             CLIGAEHVNASYECDIPIGAGICASYHTAS----VLRSTGQKSIVAYTMSLGAENSIAYA 675
TR|R9QTH3|R9QTH3_CVHSA             CLIGAEHVNASYECDIPIGAGICASYHTAS----LLRNTGQKSIVAYTMSLGAENSIAYA 676
TR|A0A0K1Z074|A0A0K1Z074_CVHSA     CLIGAEHVNSSYECDIPIGAGICASYHTAS----VLRSTGQKSIVAYTMSLGAENSVAYA 676
TR|A0A0U1WHI6|A0A0U1WHI6_CVHSA     CLIGAEHVNASYQCDIPIGAGICASYHTAS----LLRSTGQKSIVAYTMSLGAENSVAYA 674
TR|A0A166ZL64|A0A166ZL64_9NIDO     CLIGAEHVNASYQCDIPIGAGICASYHTAS----LLRSTGQKSIVAYTMSLGAENSVAYA 671
TR|A0A0U1UYX4|A0A0U1UYX4_CVHSA     CLIGAEHVNASYQCDIPIGAGICASYHTAS----LLRSTGQKSIVAYTMSLGAENSVAYA 671
                                   ********: **:**************:* :      *....:**:********:.*:**:

YP_009724390                       NNSIAIPTNFTISVTTEILPVSMTKTSVDCTMYICGDSTECSNLLLQYGSFCTQLNRALT 768
SP|P59594|SPIKE_CVHSA              NNTIAIPTNFSISITTEVMPVSMAKTSVDCNMYICGDSTECANLLLQYGSFCTQLNRALS 750
TR|Q6UZF4|Q6UZF4_CVHSA             NNTIAIPTNFSISITTEVMPVSMAKTSVDCNMYICGDSTECANLLLQYGSFCTQLNRALS 750
TR|Q6UZF0|Q6UZF0_CVHSA             NNTIAIPTNFSISITTEVMPVSMAKTSVDCNMYICGDSTECANLLLQYGSFCTQLNRALS 750
TR|Q6JH46|Q6JH46_CVHSA             NNTIAIPTNFSISITTEVMPVSMAKTSVDCNMYICGDSTECANLLLQYGSFCTQLNRALS 750
TR|Q692E4|Q692E4_CVHSA             NNTIAIPTNFSISITTEVMPVSMAKTSVDCNMYICGDSTECANLLLQYGSFCTQLNRALS 750
SP|Q3LZX1|SPIKE_BCHK3              NNSIAIPTNFSISVTTEVMPVSMAKTAVDCTMYICGDSLECSNLLLQYGSFCTQLNRALT 737
SP|Q3I5J5|SPIKE_BCRP3              NNSIAIPTNFSISVTTEVMPVSMAKTSVDCTMYICGDSLECSNLLLQYGSFCTQLNRALS 736
SP|Q0Q475|SPIKE_BC279              NNSIAIPTNFSISVTTEVMPVSIAKTSVDCTMYICGDSLECSNLLLQYGSFCTQLNRALT 736
TR|R9QTA0|R9QTA0_CVHSA             NNSIAIPTNFSISVTTEVMPVSMAKTSVDCTMYICGDSLECSNLLLQYGSFCTQLNRALT 735
TR|R9QTH3|R9QTH3_CVHSA             NNSIAIPTNFSISVTTEVMPVSMAKTSVDCTMYICGDSQECSNLLLQYGSFCTQLNRALS 736
TR|A0A0K1Z074|A0A0K1Z074_CVHSA     NNSIAIPTNFSISVTTEVMPVSMAKTSVDCTMYICGDSAECSNLLLQYGSFCTQLNRALS 736
TR|A0A0U1WHI6|A0A0U1WHI6_CVHSA     NNSIAIPTNFSISVTTEVMPVSMAKTSVDCTMYICGDSLECSNLLLQYGSFCTQLNRALS 734
TR|A0A166ZL64|A0A166ZL64_9NIDO     NNSIAIPTNFSISVTTEVMPVSMAKTSVDCTMYICGDSLECSNLLLQYGSFCTQLNRALS 731
TR|A0A0U1UYX4|A0A0U1UYX4_CVHSA     NNSIAIPTNFSISVTTEVMPVSMAKTSVDCTMYICGDSLECSNLLLQYGSFCTQLNRALS 731
                                   **:*******:**:***::***::**:**:***.******* **:*****************:

YP_009724390                       GIAVEQDKNTQEVFAQVKQIYKTPPIKDFGGFNFSQILPDPSKPSKRSFIEDLLFNKVTL 828
SP|P59594|SPIKE_CVHSA              GIAAEQDRNTREVFAQVKQMYKTPTLKYFGGFNFSQILPDPLKPTKRSFIEDLLFNKVTL 810
TR|Q6UZF4|Q6UZF4_CVHSA             GIAAEQDRNTREVFAQVKQMYKTPTLKYFGGFNFSQILPDPLKPTKRSFIEDLLFNKVTL 810
TR|Q6UZF0|Q6UZF0_CVHSA             GIAAEQDRNTREVFAQVKQMYKTPTLKYFGGFNFSQILPDPLKPTKRSFIEDLLFNKVTL 810
TR|Q6JH46|Q6JH46_CVHSA             GIAAEQDRNTREVFAQVKQMYKTPTLKYFGGFNFSQILPDPLKPTKRSFIEDLLFNKVTL 810
TR|Q692E4|Q692E4_CVHSA             GIAAEQDRNTREVFAQVKQMYKTPTLKYFGGFNFSQILPDPLKPTKRSFIEDLLFNKVTL 810
SP|Q3LZX1|SPIKE_BCHK3              GIAIEQDKNTQEVFAQVKQMYKTPAIKDFGGFNFSQILPDPSKPTKRSFIEDLLFNKVTL 797
SP|Q3I5J5|SPIKE_BCRP3              GIAIEQDKNTQEVFAQVKQMYKTPAIKDFGGFNFSQILPDPSKPTKRSFIEDLLFNKVTL 796
SP|Q0Q475|SPIKE_BC279              GIAIEQDKNTQEVFAQVKQMYKTPAIKDFGGFNFSQILPDPSKPTKRSFIEDLLFNKVTL 796
TR|R9QTA0|R9QTA0_CVHSA             GIAIEQDKNTQEVFAQVKQMYKTPAIKDFGGFNFSQILPDPSKPTKRSFIEDLLFNKVTL 795
TR|R9QTH3|R9QTH3_CVHSA             GIAVEQDKNTQEVFAQVKQMYKTPAIKDFGGFNFSQILPDPSKPTKRSFIEDLLFNKVTL 796
TR|A0A0K1Z074|A0A0K1Z074_CVHSA     GIAVEQDKNTQEVFAQVKQMYKTPTLRDFGGFNFSQILPDPLKPTKRSFIEDLLYNKVTL 796
TR|A0A0U1WHI6|A0A0U1WHI6_CVHSA     GIAVEQDKNTQEVFAQVKQMYKTPTIRDFGGFNFSQILPDPLKPTKRSFIEDLLYNKVTL 794
TR|A0A166ZL64|A0A166ZL64_9NIDO     GIAVEQDKNTQEVFAQVKQMYKTPTIRDFGGFNFSQILPDPLKPTKRSFIEDLLYNKVTL 791
TR|A0A0U1UYX4|A0A0U1UYX4_CVHSA     GIAVEQDKNTQEVFAQVKQMYKTPTIRDFGGFNFSQILPDPLKPTKRSFIEDLLYNKVTL 791
                                   *** ***:**:********:**** :: ************* **:*********:*****
```

```
YP_009724390                       ADAGFIKQYGDCLGDIAARDLICAQKFNGLTVLPPLLTDEMIAQYTSALLAGTITSGWTF 888
SP|P59594|SPIKE_CVHSA              ADAGFMKQYGECLGDINARDLICAQKFNGLTVLPPLLTDDMIAAYTAALVSGTATAGWTF 870
TR|Q6UZF4|Q6UZF4_CVHSA             ADAGFMKQYGECLGDINARDLICAQKFNGLTVLPPLLTDDMIAAYTAALVSGTATAGWTF 870
TR|Q6UZF0|Q6UZF0_CVHSA             ADAGFMKQYGECLGDINARDLICAQKFNGLTVLPPLLTDDMIAAYTAALVSGTATAGWTF 870
TR|Q6JH46|Q6JH46_CVHSA             ADAGFMKQYGECLGDINARDLICAQKFNGLTVLPPLLTDDMIAAYTAALVSGTATAGWTF 870
TR|Q692E4|Q692E4_CVHSA             ADAGFMKQYGECLGDINARDLICAQKFNGLTVLPPLLTDDMIAAYTAALVSGTATAGWTF 870
SP|Q3LZX1|SPIKE_BCHK3              ADAGFMKQYGDCLGDVSARDLICAQKFNGLTVLPPLLTDEMVAAYTAALVSGTATAGWTF 857
SP|Q3I5J5|SPIKE_BCRP3              ADAGFMKQYGECLGDISARDLICAQKFNGLTVLPPLLTDEMIAAYTAALVSGTATAGWTF 856
SP|Q0Q475|SPIKE_BC279              ADAGFMKQYGECLGDISARDLICAQKFNGLTVLPPLLTDEMIAAYTAALVSGTATAGWTF 856
TR|R9QTA0|R9QTA0_CVHSA             ADAGFMKQYGECLGDISARDLICAQKFNGLTVLPPLLTDEMIAAYTAALVSGTATAGWTF 855
TR|R9QTH3|R9QTH3_CVHSA             ADAGFMKQYGECLGDISARDLICAQKFNGLTVLPPLLTDEMIAAYTAALVSGTATAGWTF 856
TR|A0A0K1Z074|A0A0K1Z074_CVHSA     ADAGFMKQYADCLGGINARDLICAQKFNGLTVLPPLLTDDMIAAYTAALISGTATAGWTF 856
TR|A0A0U1WHI6|A0A0U1WHI6_CVHSA     ADAGFMKQYADCLGGINARDLICAQKFNGLTVLPPLLTDDMIAAYTAALISGTATAGWTF 854
TR|A0A166ZL64|A0A166ZL64_9NIDO     ADAGFMKQYADCLGGINARDLICAQKFNGLTVLPPLLTDDMIAAYTAALISGTATAGWTF 851
TR|A0A0U1UYX4|A0A0U1UYX4_CVHSA     ADAGFMKQYADCLGGINARDLICAQKFNGLTVLPPLLTDDMIAAYTAALISGTATAGWTF 851
                                   *****:***.:***.: **********************:*:* **:**::** *:****

YP_009724390                       GAGAALQIPFAMQMAYRFNGIGVTQNVLYENQKLIANQFNSAIGKIQDSLSSTASALGKL 948
SP|P59594|SPIKE_CVHSA              GAGAALQIPFAMQMAYRFNGIGVTQNVLYENQKQIANQFNKAISQIQESLTTTSTALGKL 930
TR|Q6UZF4|Q6UZF4_CVHSA             GAGAALQIPFAMQMAYRFNGIGVTQNVLYENQKQIANQFNKAISQIQESLTTTSTALGKL 930
TR|Q6UZF0|Q6UZF0_CVHSA             GAGAALQIPFAMQMAYRFNGIGVTQNVLYENQKQIANQFNKAISQIQESLTTTSTALGKL 930
TR|Q6JH46|Q6JH46_CVHSA             GAGAALQIPFAMQMAYRFNGIGVTQNVLYENQKQIANQFNKAISQIQESLTTTSTALGKL 930
TR|Q692E4|Q692E4_CVHSA             GAGAALQIPFAMQMAYRFNGIGVTQNVLYENQKQIANQFNKAISQIQESLTTTSTALGKL 930
SP|Q3LZX1|SPIKE_BCHK3              GAGAALQIPFAMQMAYRFNGIGVTQNVLYENQKLIANQFNSAIGKIQESLSSTASALGKL 917
SP|Q3I5J5|SPIKE_BCRP3              GAGSALQIPFAMQMAYRFNGIGVTQNVLYENQKQIANQFNKAISQIQESLTTTSTALGKL 916
SP|Q0Q475|SPIKE_BC279              GAGSALQIPFAMQMAYRFNGIGVTQNVLYENQKQIANQFNKAISQIQESLTTTSTALGKL 916
TR|R9QTA0|R9QTA0_CVHSA             GAGAALQIPFAMQMAYRFNGIGVTQNVLYENQKQIANQFNKAISQIQESLTTTSTALGKL 915
TR|R9QTH3|R9QTH3_CVHSA             GAGAALQIPFAMQMAYRFNGIGVTQNVLYENQKQIANQFNKAISQIQESLTTTSTALGKL 916
TR|A0A0K1Z074|A0A0K1Z074_CVHSA     GAGAALQIPFAMQMAYRFNGIGVTQNVLYENQKQIANQFNKAITQIQESLTTTSTALGKL 916
TR|A0A0U1WHI6|A0A0U1WHI6_CVHSA     GAGAALQIPFAMQMAYRFNGIGVTQNVLYENQKQIANQFNKAITQIQESLTTTSTALGKL 914
TR|A0A166ZL64|A0A166ZL64_9NIDO     GAGAALQIPFAMQMAYRFNGIGVTQNVLYENQKQIANQFNKAITQIQESLTTTATALGKL 911
TR|A0A0U1UYX4|A0A0U1UYX4_CVHSA     GAGAALQIPFAMQMAYRFNGIGVTQNVLYENQKQIANQFNKAITQIQESLTTTATALGKL 911
                                   ***:***************************** ******.** :**:**::*::*****

YP_009724390                       QDVVNQNAQALNTLVKQLSSNFGAISSVLNDILSRLDKVEAEVQIDRLITGRLQSLQTYV 1008
SP|P59594|SPIKE_CVHSA              QDVVNQNAQALNTLVKQLSSNFGAISSVLNDILSRLDKVEAEVQIDRLITGRLQSLQTYV 990
TR|Q6UZF4|Q6UZF4_CVHSA             QDVVNQNAQALNTLVKQLSSNFGAISSVLNDILSRLDKVEAEVQIDRLITGRLQSLQTYV 990
TR|Q6UZF0|Q6UZF0_CVHSA             QDVVNQNAQALNTLVKQLSSNFGAISSVLNDILSRLDKVEAEVQIDRLITGRLQSLQTYV 990
TR|Q6JH46|Q6JH46_CVHSA             QDVVNQNAQALNTLVKQLSSNFGAISSVLNDILSRLDKVEAEVQIDRLITGRLQSLQTYV 990
TR|Q692E4|Q692E4_CVHSA             QDVVNQNAQALNTLVKQLSSNFGAISSVLNDILSRLDKVEAEVQIDRLITGRLQSLQTYV 990
SP|Q3LZX1|SPIKE_BCHK3              QDVVNQNAQALNTLVKQLSSNFGAISSVLNDILSRLDKVEAEVQIDRLITGRLQSLQTYV 977
SP|Q3I5J5|SPIKE_BCRP3              QDVVNQNAQALNTLVKQLSSNFGAISSVLNDILSRLDKVEAEVQIDRLITGRLQSLQTYV 976
SP|Q0Q475|SPIKE_BC279              QDVVNDNAQALNTLVKQLSSNFGAISSVLNDILSRLDKVEAEVQIDRLITGRLQSLQTYV 976
TR|R9QTA0|R9QTA0_CVHSA             QDVVNQNAQALNTLVKQLSSNFGAISSVLNDILSRLDKVEAEVQIDRLITGRLQSLQTYV 975
TR|R9QTH3|R9QTH3_CVHSA             QDVVNQNAQALNTLVKQLSSNFGAISSVLNDILSRLDKVEAEVQIDRLITGRLQSLQTYV 976
TR|A0A0K1Z074|A0A0K1Z074_CVHSA     QDVVNQNAQALNTLVKQLSSNFGAISSVLNDILSRLDKVEAEVQIDRLITGRLQSLQTYV 976
TR|A0A0U1WHI6|A0A0U1WHI6_CVHSA     QDVVNQNAQALNTLVKQLSSNFGAISSVLNDILSRLDKVEAEVQIDRLITGRLQSLQTYV 974
TR|A0A166ZL64|A0A166ZL64_9NIDO     QDVVNQNAQALNTLVKQLSSNFGAISSALNDILSRLDKVEAEVQIDRLITGRLQSLQTYV 971
TR|A0A0U1UYX4|A0A0U1UYX4_CVHSA     QDVVNQNAQALNTLVKQLSSNFGAISSALNDILSRLDKVEAEVQIDRLITGRLQSLQTYV 971
                                   *****:*********************.********************************

YP_009724390                       TQQLIRAAEIRASANLAATKMSECVLGQSKRVDFCGKGYHLMSFPQSAPHGVVFLHVTYV 1068
SP|P59594|SPIKE_CVHSA              TQQLIRAAEIRASANLAATKMSECVLGQSKRVDFCGKGYHLMSFPQAAPHGVVFLHVTYV 1050
TR|Q6UZF4|Q6UZF4_CVHSA             TQQLIRAAEIRASANLAATKMSECVLGQSKRVDFCGKGYHLMSFPQAAPHGVVFLHVTYV 1050
TR|Q6UZF0|Q6UZF0_CVHSA             TQQLIRAAEIRASANLAATKMSECVLGQSKRVDFCGKGYHLMSFPQAAPHGVVFLHVTYV 1050
TR|Q6JH46|Q6JH46_CVHSA             TQQLIRAAEIRASANLAATKMSECVLGQSKRVDFCGKGYHLMSFPQAAPHGVVFLHVTYV 1050
TR|Q692E4|Q692E4_CVHSA             TQQLIRAAEIRASANLAATKMSECVLGQSKRVDFCGKGYHLMSFPQAAPHGVVFLHVTYV 1050
SP|Q3LZX1|SPIKE_BCHK3              TQQLIRAAEIRASANLAATKMSECVLGQSKRVDFCGKGYHLMSFPQSAPHGVVFLHVTYV 1037
SP|Q3I5J5|SPIKE_BCRP3              TQQLIRAAEIRASANLAATKMSECVLGQSKRVDFCGKGYHLMSFPQAAPHGVVFLHVTYV 1036
SP|Q0Q475|SPIKE_BC279              TQQLIRAAEIRASANLAATKMSECVLGQSKRVDFCGKGYHLMSFPQAAPHGVVFLHVTYV 1036
TR|R9QTA0|R9QTA0_CVHSA             TQQLIRAAEIRASANLAATKMSECVLGQSKRVDFCGKGYHLMSFPQAAPHGVVFLHVTYV 1035
TR|R9QTH3|R9QTH3_CVHSA             TQQLIRAAEIRASANLAATKMSECVLGQSKRVDFCGKGYHLMSFPQAAPHGVVFLHVTYV 1036
TR|A0A0K1Z074|A0A0K1Z074_CVHSA     TQQLIRAAEIRASANLAATKMSECVLGQSKRVDFCGKGYHLMSFPQAAPHGVVFLHVTYV 1036
TR|A0A0U1WHI6|A0A0U1WHI6_CVHSA     TQQLIRAAEIRTSANLAATKMSECVLGQSKRVDFCGKGYHLMSFPQSAPHGVVFLHVTYV 1034
TR|A0A166ZL64|A0A166ZL64_9NIDO     TQQLIRAAEIRASANLAATKMSECVLGQSKRVDFCGKGYHLMSFPQSAPHGVVFLHVTYV 1031
TR|A0A0U1UYX4|A0A0U1UYX4_CVHSA     TQQLIRAAEIRASANLAATKMSECVLGQSKRVDFCGKGYHLMSFPQSAPHGVVFLHVTYV 1031
                                   ***********:**********************************:*************
```

```
YP_009724390                      PAQEKNFTTAPAICHDGKAHFPREGVFVSNGTHWFVTQRNFYEPQIITTDNTFVSGNCDV 1128
SP|P59594|SPIKE_CVHSA             PSQERNFTTAPAICHEGKAYFPREGVFVFNGTSWFITQRNFFSPQIITTDNTFVSGNCDV 1110
TR|Q6UZF4|Q6UZF4_CVHSA            PSQERNFTTAPAICHEGKAYFPREGVFVFNGTSWFITQRNFFSPQIITTDNTFVSGNCDV 1110
TR|Q6UZF0|Q6UZF0_CVHSA            PSQERNFTTAPAICHEGKAYFPREGVFVFNGTSWFITQRNFFSPQIITTDNTFVSGNCDV 1110
TR|Q6JH46|Q6JH46_CVHSA            PSQERNFTTAPAICHEGKAYFPREGVFVFNGTSWFITQRNFFSPQIITTDNTFVSGNCDV 1110
TR|Q692E4|Q692E4_CVHSA            PSQERNFTTAPAICHEGKAYFPREGVFVFNGTSWFITQRNFFSPQIITTDNTFVSGKCDV 1110
SP|Q3LZX1|SPIKE_BCHK3             PSQEKNFTTAPAICHEGKAYFPREGVFVSNGTSWFITQRNFYSPQLITTDNTFVSGNCDV 1097
SP|Q3I5J5|SPIKE_BCRP3             PSQERNFTTAPAICHEGKAYFPREGVFVSNGTSWFITQRNFYSPQIITTDNTFVAGSCDV 1096
SP|Q0Q475|SPIKE_BC279             PSQERNFTTAPAICHEGKAYFPREGVFVSNGTSWFITQRNFYSPQIITTDNTFVAGNCDV 1096
TR|R9QTA0|R9QTA0_CVHSA            PSQERNFTTAPAICHEGKAYFPREGVFVSNGTSWFITQRNFYSPQIITTDNTFVAGSCNV 1095
TR|R9QTH3|R9QTH3_CVHSA            PSQERNFTTAPAICHQGKAYFPREGVFVSNGTSWFITQRNFFSPQIITTDNTFVSGNCDV 1096
TR|A0A0K1Z074|A0A0K1Z074_CVHSA PSQEKNFTTAPAICHEGKAYFPREGVFVFNGTSWFITQRNFYSPQTITTDNTFVAGNCDV 1096
TR|A0A0U1WHI6|A0A0U1WHI6_CVHSA PSQERNFTTAPAICHEGKAYFPREGVFVSNGSSWFITQRNFYSPQIITTDNTFVAGSCDV 1094
TR|A0A166ZL64|A0A166ZL64_9NIDO PAQEKNFTTAPAICHEGKAYFPREGVFVSNGSSWFITQRNFYSPQIITTDNTFVAGSCDV 1091
TR|A0A0U1UYX4|A0A0U1UYX4_CVHSA PAQEKNFTTAPAICHEGKAYFPREGVFVSNGSSWFITQRNFYSPQIITTDNTFVAGSCDV 1091
                                  *:**:**********:***:******** **: **:*****:.** ********:*.*:*

YP_009724390                      VIGIVNNTVYDPLQPELDSFKEELDKYFKNHTSPDVDLGDISGINASVVNIQKEIDRLNE 1188
SP|P59594|SPIKE_CVHSA             VIGIINNTVYDPLQPELDSFKEELDKYFKNHTSPDVDLGDISGINASVVNIQKEIDRLNE 1170
TR|Q6UZF4|Q6UZF4_CVHSA            VIGIINNTVYDPLQPELDSFKEELDKYFKNHTSPDVDLGDISGINASVVNIQKEIDRLNE 1170
TR|Q6UZF0|Q6UZF0_CVHSA            VIGIINNTVYDPLQPELDSFKEELDKYFKNHTSPDVDLGDISGINASVVNIQKEIDRLNE 1170
TR|Q6JH46|Q6JH46_CVHSA            VIGIINNTVYDPLQPELDSFKEELDKYFKNHTSPDVDLGDISGINASVVNIQKEIDRLNE 1170
TR|Q692E4|Q692E4_CVHSA            VIGIINNTVYDPLQPELDSFKEELDKYFKNHTSPDVDLGDISGINASVVNIQKEIDRLNE 1170
SP|Q3LZX1|SPIKE_BCHK3             VIGIINNTVYDPLQPELDSFKEELDKYFKNHTSPDVDLGDISGINASVVNIQKEIDRLNE 1157
SP|Q3I5J5|SPIKE_BCRP3             VIGIINNTVYDPLQPELDSFKEELDKYFKNHTSPDVDLGDISGINASVVNIQKEIDRLNE 1156
SP|Q0Q475|SPIKE_BC279             VIGIINNTVYDPLQPELDSFKEELDKYFKNHTSPDVDLGDISGINASVVNIQKEIDRLNE 1156
TR|R9QTA0|R9QTA0_CVHSA            VIGIINNTVYDPLQPELDSFKEELDKYFKNHTSPDVDLGDISGINASVVNIQKEIDRLNE 1155
TR|R9QTH3|R9QTH3_CVHSA            VIGIINNTVYDPLQPELDSFKEELDKYFKNHTSPDVDLGDISGINASVVNIQKEIDRLNE 1156
TR|A0A0K1Z074|A0A0K1Z074_CVHSA VIGIINNTVYDPLQPELDSFKEELDKYFKNHTSPDVDLGDISGINASVVNIQKEIDRLNE 1156
TR|A0A0U1WHI6|A0A0U1WHI6_CVHSA VIGIINNTVYDPLQPELDSFKQELDKYFKNHTSPDVDFGDISGINASVVDIQKEIDRLNE 1154
TR|A0A166ZL64|A0A166ZL64_9NIDO VIGIINNTVYDPLQPELDSFKQELDKYFKNHTSPDVDLGDISGINASVVDIQKEIDRLNE 1151
TR|A0A0U1UYX4|A0A0U1UYX4_CVHSA VIGIINNTVYDPLQPELDSFKQELDKYFKNHTSPDVDLGDISGINASVVDIQKEIDRLNE 1151
                                  ****:****************:***************:***********:**********

YP_009724390                      VAKNLNESLIDLQELGKYEQYIKWPWYIWLGFIAGLIAIVMVTIMLCCMTSCCSCLKGCC 1248
SP|P59594|SPIKE_CVHSA             VAKNLNESLIDLQELGKYEQYIKWPWYVWLGFIAGLIAIVMVTILLCCMTSCCSCLKGAC 1230
TR|Q6UZF4|Q6UZF4_CVHSA            VAKNLNESLIDLQELGKYEQYIKWPWYVWLGFIAGLIAIVMVTILLCCMTSCCSCLKGAC 1230
TR|Q6UZF0|Q6UZF0_CVHSA            VAKNLNESLIDLQELGKYEQYIKWPWYVWLGFIAGLIAIVMVTILLCCMTSCCSCLKGAC 1230
TR|Q6JH46|Q6JH46_CVHSA            VAKNLNESLIDLQELGKYEQYIKWPWYVWLGFIAGLIAIVMVTILLCCMTSCCSCLKGAC 1230
TR|Q692E4|Q692E4_CVHSA            VAKNLNESLIDLQELGKYEQYIKWPWYVWLGFIAGLIAIVMVTILLCCMTSCCSCLKGAC 1230
SP|Q3LZX1|SPIKE_BCHK3             VAKNLNESLIDLQELGKYEQYIKWPWYVWLGFIAGLIAIVMVTILLCCMTSCCSCLKGAC 1217
SP|Q3I5J5|SPIKE_BCRP3             VAKNLNESLIDLQELGKYEQYIKWPWYVWLGFIAGLIAIVMVTILLCCMTSCCSCLKGAC 1216
SP|Q0Q475|SPIKE_BC279             VAKNLNESLIDLQELGKYEQYIKWPWYVWLGFIAGLIAIVMVTILLCCMTSCCSCLKGAC 1216
TR|R9QTA0|R9QTA0_CVHSA            VAKNLNESLIDLQELGKYEQYIKWPWYVWLGFIAGLIAIVMVTILLCCMTSCCSCLKGAC 1215
TR|R9QTH3|R9QTH3_CVHSA            VAKNLNESLIDLQELGKYEQYIKWPWYVWLGFIAGLIAIVMATILLCCMTSCCSCLKGAC 1216
TR|A0A0K1Z074|A0A0K1Z074_CVHSA VAKNLNDSLIDLQELGKYEQYIKWPWYVWLGFIAGLVGLFMAIILLCYFTSCCSCCKGMC 1216
TR|A0A0U1WHI6|A0A0U1WHI6_CVHSA VAKNLNESLIDLQELGKYEQYIKWPWYVWLGFIAGLVGLFMAIILLCYFTSCCSCCKGMC 1214
TR|A0A166ZL64|A0A166ZL64_9NIDO VAKNLNESLIDLQELGKYEQYIKWPWYVWLGFIAGLVGLFMAIILLCYFTSCCSCCKGMC 1211
TR|A0A0U1UYX4|A0A0U1UYX4_CVHSA VAKNLNESLIDLQELGKYEQYIKWPWYVWLGFIAGLVGLFMAIILLCYFTSCCSCCKGMC 1211
                                  ******:********************:********:.:.*. *:** :****** ** *

YP_009724390                      SCGSCCKFDEDDSEPVLKGVKLHYT 1273
SP|P59594|SPIKE_CVHSA             SCGSCCKFDEDDSEPVLKGVKLHYT 1255
TR|Q6UZF4|Q6UZF4_CVHSA            SCGSCCKFDEDDSEPVLKGVKLHYT 1255
TR|Q6UZF0|Q6UZF0_CVHSA            SCGSCCKFDEDDSEPVLKGVKLHYT 1255
TR|Q6JH46|Q6JH46_CVHSA            SCGSCCKFDEDDSEPVLKGVKLHYT 1255
TR|Q692E4|Q692E4_CVHSA            SCGSCCKFDEDDSEPVLKGVKLHYT 1255
SP|Q3LZX1|SPIKE_BCHK3             SCGSCCKFDEDDSEPVLKGVKLHYT 1242
SP|Q3I5J5|SPIKE_BCRP3             SCGSCCKFDEDDSEPVLKGVKLHYT 1241
SP|Q0Q475|SPIKE_BC279             SCGSCCKFDEDDSEPVLKGVKLHYT 1241
TR|R9QTA0|R9QTA0_CVHSA            SCGSCCKFDEDDSEPVLKGVKLHYT 1240
TR|R9QTH3|R9QTH3_CVHSA            SCGSCCKFDEDDSEPVLKGVKLHYT 1241
TR|A0A0K1Z074|A0A0K1Z074_CVHSA SCGSCCRFDEDDSEPVLKGVKLHYT 1241
TR|A0A0U1WHI6|A0A0U1WHI6_CVHSA SCGSCCRFDEDDSEPVLKGVKLHYT 1239
TR|A0A166ZL64|A0A166ZL64_9NIDO SCGSCCRFDEDDSEPVLKGVKLHYT 1236
TR|A0A0U1UYX4|A0A0U1UYX4_CVHSA SCGSCCRFDEDDSEPVLKGVKLHYT 1236
                                  ******:******************
```
