# Supplementary material for: Structural Genomics of SARS-CoV-2 Indicates Evolutionary Conserved Functional Regions of Viral Proteins
Source: Viruses. 2020 Mar 25;12(4):360. doi: 10.3390/v12040360 (PMC7232164; doi:10.3390/v12040360)
Supplement: Supplementary file 1 [file viruses-12-00360-s001.zip › wORF1AB_Nsp7_Nsp8_Nsp10_Nsp12_Nsp14_Nsp16_PBS_mapped.pdf]

Supplementary File 8. Intra-viral protein binding sites mapped on the sequences of wNsp7, wNsp8, wNsp10, wNsp12, wNsp14, wNsp16 proteins aligned with the closest homologs found in UniProt.

Intra-Viral Heteromeric Interaction Protein Binding Sites:

- wNsp7 (yellow)
- wNsp8 (green)
- wNsp12 (cyan)
- wNsp10 (magenta)
- wNsp14 (blue)
- wNsp16 (red)

```
QHN73794                           LLPPKNSIDAFKLNIKLLGVGGKPCIKVATVQSKMSDVKCTSVVLLSVLQQLRVESSSKL 3887
SP|P0C6X7|R1AB_CVHSA               LLPPKSSIDAFKLNIKLLGIGGKPCIKVATVQSKMSDVKCTSVVLLSVLQQLRVESSSKL 3864
TR|Q6UZF5|Q6UZF5_CVHSA             LLPPKSSIDAFKLNIKLLGIGGKPCIKVATVQSKMSDVKCTSVVLLSVLQQLRVESSSKL 3864
TR|Q6UZF1|Q6UZF1_CVHSA             LLPPKSSIDAFKLNIKLLGIGGKPCIKVATVQSKMSDVKCTSVVLLSVLQQLRVESSSKL 3864
TR|Q6JH48|Q6JH48_CVHSA             LLPPKSSIDAFKLNIKLLGIGGKPCIKVATVQSKMSDVKCTSVVLLSVLQQLRVESSSKL 3864
TR|Q692E6|Q692E6_CVHSA             LLPPKSSIDAFKLNIKLLGIGGKPCIKVATVQSKMSDVKCTSVVLLSVLQQLRVESSSKL 3864
TR|A0A0K1YZY7|A0A0K1YZY7_CVHSA LLPPKSSIDAFRLNIKLLGIGGKPCIKVATVQSKMSDVKCTSVVLLSVLQQLRVESSSKL 3864
SP|P0C6W2|R1AB_BCHK3               LLPPKSSIDAFKLNIKLLGIGGKPCIKVATVQSKMSDVKCTSVVLLSVLQQLRVESSSKL 3858
SP|P0C6W6|R1AB_BCRP3               LLPPKSSIDAFKLNIKLLGIGGKPCIKVATVQSKMSDVKCTSVVLLSVLQQLRVESSSKL 3862
SP|P0C6V9|R1AB_BC279               LLPPKSSIDAFKLNIKLLGIGGKPCIKVATVQSKMSDVKCTSVVLLSVLQQLRVESSSKL 3870
TR|A0A0U1WHI4|A0A0U1WHI4_CVHSA LLPPKSSIDAFKLNIKLLGIGGKPCIKVATVQSKMSDVKCTSVVLLSVLQQLRVESSSKL 3859
TR|A0A0U1WHG0|A0A0U1WHG0_CVHSA LLPPKSSIDAFKLNIKLLGIGGKPCIKVATVQSKMSDVKCTSVVLLSVLQQLRVESSSKL 3859
TR|A0A166ZL34|A0A166ZL34_9NIDO LLPPKSSIDAFKLNIKLLGIGGKPCIKVATVQSKMSDVKCTSVVLLSVLQQLRVESSSKL 3666
TR|R9QTB2|R9QTB2_CVHSA             LLPPKSSIDAFKLNIKLLGIGGKPCIKVATVQSKMSDVKCTSVVLLSVLQQLRVESSSKL 3856
TR|R9QTH2|R9QTH2_CVHSA             LLPPKSSIDAFKLNIKLLGIGGKPCIKVATVQSKMSDVKCTSVVLLSVLQQLRVESSSKL 3865
SP|P0C6U8|R1A_CVHSA                LLPPKSSIDAFKLNIKLLGIGGKPCIKVATVQSKMSDVKCTSVVLLSVLQQLRVESSSKL 3864
TR|Q6JH47|Q6JH47_CVHSA             LLPPKSSIDAFKLNIKLLGIGGKPCIKVATVQSKMSDVKCTSVVLLSVLQQLRVESSSKL 3864
TR|Q692E5|Q692E5_CVHSA             LLPPKSSIDAFKLNIKLLGIGGKPCIKVATVQSKMSDVKCTSVVLLSVLQQLRVESSSKL 3864
SP|P0C6F8|R1A_BCHK3                LLPPKSSIDAFKLNIKLLGIGGKPCIKVATVQSKMSDVKCTSVVLLSVLQQLRVESSSKL 3858
TR|A0A0K1Z0N1|A0A0K1Z0N1_CVHSA LLPPKSSIDAFRLNIKLLGIGGKPCIKVATVQSKMSDVKCTSVVLLSVLQQLRVESSSKL 3864
SP|P0C6F5|R1A_BC279                LLPPKSSIDAFKLNIKLLGIGGKPCIKVATVQSKMSDVKCTSVVLLSVLQQLRVESSSKL 3870
SP|P0C6T7|R1A_BCRP3                LLPPKSSIDAFKLNIKLLGIGGKPCIKVATVQSKMSDVKCTSVVLLSVLQQLRVESSSKL 3862
                                   *****.*****:*******:****************************************

QHN73794                           WAQCVQLHNDILLAKDTTEAFEKMVSLLSVLLSMQGAVDINKLCEEMLDNRATLQAIASE 3947
SP|P0C6X7|R1AB_CVHSA               WAQCVQLHNDILLAKDTTEAFEKMVSLLSVLLSMQGAVDINRLCEEMLDNRATLQAIASE 3924
TR|Q6UZF5|Q6UZF5_CVHSA             WAQCVQLHNDILLAKDTTEAFEKMVSLLSVLLSMQGAVDINRLCEEMLDNRATLQAIASE 3924
TR|Q6UZF1|Q6UZF1_CVHSA             WAQCVQLHNDILLAKDTTEAFEKMVSLLSVLLSMQGAVDINRLCEEMLDNRATLQAIASE 3924
TR|Q6JH48|Q6JH48_CVHSA             WAQCVQLHNDILLAKDTTEAFEKMVSLLSVLLSMQGAVDINRLCEEMLDNRATLQAIASE 3924
TR|Q692E6|Q692E6_CVHSA             WAQCVQLHNDILLAKDTTEAFEKMVSLLSVLLSMQGAVDINRLCEEMLDNRATLQAIASE 3924
TR|A0A0K1YZY7|A0A0K1YZY7_CVHSA WAQCVQLHNDILLAKDTTAAFEKMVSLLSVLLSMQGAVDISKLCEEMLDNRATLQAIASE 3924
SP|P0C6W2|R1AB_BCHK3               WAQCVQLHNDILLAKDTTEAFEKMVSLLSVLLSMQGAVDINKLCEEMLDNRATLQAIASE 3918
SP|P0C6W6|R1AB_BCRP3               WAQCVQLHNDILLAKDTTEAFEKMVSLLSVLLSMQGAVDINKLCEEMLDNRATLQAIASE 3922
SP|P0C6V9|R1AB_BC279               WAQCVQLHNDILLAKDTTEAFEKMVSLLSVLLSMQGAVDINKLCEEMLDNRATLQAIASE 3930
TR|A0A0U1WHI4|A0A0U1WHI4_CVHSA WAQCVQLHNDILLAKDTTEAFEKMVSLLSVLLSMQGAVDINKLCEEMLDNRATLQAIASE 3919
TR|A0A0U1WHG0|A0A0U1WHG0_CVHSA WARCVQLHNDILLAKDTTEAFEKMVSLLSVLLSMQGAVDINKLCEEMLDNRATLQAIASE 3919
TR|A0A166ZL34|A0A166ZL34_9NIDO WARCVQLHNDILLAKDTTEAFEKMVSLLSVLLSMQGAVDINKLCEEMLDNRATLQAIASE 3726
TR|R9QTB2|R9QTB2_CVHSA             WAQCVQLHNDILLAKDTTEAFEKMVSLLSVLLSMQGAVDINKLCEEMLDNRATLQAIASE 3916
TR|R9QTH2|R9QTH2_CVHSA             WAQCVQLHNDILLAKDTTEAFEKMVSLLSVLLSMQGAVDINKLCEEMLDNRATLQAIASE 3925
SP|P0C6U8|R1A_CVHSA                WAQCVQLHNDILLAKDTTEAFEKMVSLLSVLLSMQGAVDINRLCEEMLDNRATLQAIASE 3924
TR|Q6JH47|Q6JH47_CVHSA             WAQCVQLHNDILLAKDTTEAFEKMVSLLSVLLSMQGAVDINRLCEEMLDNRATLQAIASE 3924
TR|Q692E5|Q692E5_CVHSA             WAQCVQLHNDILLAKDTTEAFEKMVSLLSVLLSMQGAVDINRLCEEMLDNRATLQAIASE 3924
SP|P0C6F8|R1A_BCHK3                WAQCVQLHNDILLAKDTTEAFEKMVSLLSVLLSMQGAVDINKLCEEMLDNRATLQAIASE 3918
TR|A0A0K1Z0N1|A0A0K1Z0N1_CVHSA WAQCVQLHNDILLAKDTTAAFEKMVSLLSVLLSMQGAVDISKLCEEMLDNRATLQAIASE 3924
SP|P0C6F5|R1A_BC279                WAQCVQLHNDILLAKDTTEAFEKMVSLLSVLLSMQGAVDINKLCEEMLDNRATLQAIASE 3930
SP|P0C6T7|R1A_BCRP3                WAQCVQLHNDILLAKDTTEAFEKMVSLLSVLLSMQGAVDINKLCEEMLDNRATLQAIASE 3922
                                   **:*************** *********************.:******************

QHN73794                           FSSLPSYAAFATAQEAYEQAVANGDSEVVLKKLKKSLNVAKSEFDRDAAMQRKLEKMADQ 4007
SP|P0C6X7|R1AB_CVHSA               FSSLPSYAAYATAQEAYEQAVANGDSEVVLKKLKKSLNVAKSEFDRDAAMQRKLEKMADQ 3984
TR|Q6UZF5|Q6UZF5_CVHSA             FSSLPSYAAYATAQEAYEQAVANGDSEVVLKKLKKSLNVAKSEFDRDAAMQRKLEKMADQ 3984
TR|Q6UZF1|Q6UZF1_CVHSA             FSSLPSYAAYATAQEAYEQAVANGDSEVVLKKLKKSLNVAKSEFDRDAAMQRKLEKMADQ 3984
TR|Q6JH48|Q6JH48_CVHSA             FSSLPSYAAYATAQEAYEQAVANGDSEVVLKKLKKSLNVAKSEFDRDAAMQRKLEKMADQ 3984
TR|Q692E6|Q692E6_CVHSA             FSSLPSYAAYATAQEAYEQAVANGDSEVVLKKLKKSLNVAKSEFDRDAAMQRKLEKMADQ 3984
TR|A0A0K1YZY7|A0A0K1YZY7_CVHSA FSSLPSYAAYATAQEAYEQAVANGDSEVVLKKLKKSLNVAKSEFDRDAAMQRKLEKMADQ 3984
SP|P0C6W2|R1AB_BCHK3               FSSLPSYAAYATAQEAYEQAVSNGDSEVVLKKLKKSLNVAKSEFDHDAAMQRKLEKMADQ 3978
SP|P0C6W6|R1AB_BCRP3               FSSLPSYAAYATAQEAYEQAVANGDSEVVLKKLKKSLNVAKSEFDRNAAMQRKLEKMADQ 3982
SP|P0C6V9|R1AB_BC279               FSSLPSYAAYATAQEAYEQAVANGDSEVVLKKLKKSLNVAKSEFDRDAAMQRKLEKMADQ 3990
TR|A0A0U1WHI4|A0A0U1WHI4_CVHSA FSSLPSYAAYATAQEAYGQAVANGDSEVVLKKLKKSLNVAKSEFDRDAAMQRKLEKMADQ 3979
TR|A0A0U1WHG0|A0A0U1WHG0_CVHSA FSSLPSYAAYATAQEAYEQAVANGDSEILKKLKKSLNVAKSEFDRDAAMQRKLEKMADQ 3979
TR|A0A166ZL34|A0A166ZL34_9NIDO FSSLPSYAAYATAQEAYEQAVANGDSEILKKLKKSLNVAKSEFDRDAAMQRKLEKMADQ 3786
TR|R9QTB2|R9QTB2_CVHSA             FSSLPSYAAYATAQEAYEQAVANGDSEVVLKKLKKSLNVAKSEFDRDAAMQRKLEKMADQ 3976
TR|R9QTH2|R9QTH2_CVHSA             FSSLPSYAAYATAQEAYEQAVANGDSEVVLKKLKKCLNVAKSEFDRDAAMQRKLEKMADQ 3985
SP|P0C6U8|R1A_CVHSA                FSSLPSYAAYATAQEAYEQAVANGDSEVVLKKLKKSLNVAKSEFDRDAAMQRKLEKMADQ 3984
TR|Q6JH47|Q6JH47_CVHSA             FSSLPSYAAYATAQEAYEQAVANGDSEVVLKKLKKSLNVAKSEFDRDAAMQRKLEKMADQ 3984
TR|Q692E5|Q692E5_CVHSA             FSSLPSYAAYATAQEAYEQAVANGDSEVVLKKLKKSLNVAKSEFDRDAAMQRKLEKMADQ 3984
SP|P0C6F8|R1A_BCHK3                FSSLPSYAAYATAQEAYEQAVSNGDSEVVLKKLKKSLNVAKSEFDHDAAMQRKLEKMADQ 3978
TR|A0A0K1Z0N1|A0A0K1Z0N1_CVHSA FSSLPSYAAYATAQEAYEQAVANGDSEVVLKKLKKSLNVAKSEFDRDAAMQRKLEKMADQ 3984
SP|P0C6F5|R1A_BC279                FSSLPSYAAYATAQEAYEQAVANGDSEVVLKKLKKSLNVAKSEFDRDAAMQRKLEKMADQ 3990
SP|P0C6T7|R1A_BCRP3                FSSLPSYAAYATAQEAYEQAVANGDSEVVLKKLKKSLNVAKSEFDRNAAMQRKLEKMADQ 3982
                                   *********:******* ***:******:******.*********::*************
```

```
QHN73794                           AMTQMYKQARSEDKRAKVTSAMQTMLFTMLRKLDNDALNNIINNARDGCVPLNIIPLTTA 4067
SP|P0C6X7|R1AB_CVHSA               AMTQMYKQARSEDKRAKVTSAMQTMLFTMLRKLDNDALNNIINNARDGCVPLNIIPLTTA 4044
TR|Q6UZF5|Q6UZF5_CVHSA             AMTQMYKQARSEDKRAKVTSAMQTMLFTMLRKLDNDALNNIINNARDGCVPLNIIPLTTA 4044
TR|Q6UZF1|Q6UZF1_CVHSA             AMTQMYKQARSEDKRAKVTSAMQTMLFTMLRKLDNDALNNIINNARDGCVPLNIIPLTTA 4044
TR|Q6JH48|Q6JH48_CVHSA             AMTQMYKQARSEDKRAKVTSAMQTMLFTMLRKLDNDALNNIINNARDGCVPLNIIPLTTA 4044
TR|Q692E6|Q692E6_CVHSA             AMTQMYKQARSEDKRAKVTSAMQTMLFTMLRKLDNDALNNIINNARDGCVPLNIIPLTTA 4044
TR|A0A0K1YZY7|A0A0K1YZY7_CVHSA     AMTQMYKQARSEDKRAKVTSAMQTMLFTMLRKLDNDALNNIINNARDGCVPLNIIPLTTA 4044
SP|P0C6W2|R1AB_BCHK3               AMTQMYKQARSEDKRAKVTSAMQTMLFTMLRKLDNDALNNIINNARDGCVPLNIIPLTTA 4038
SP|P0C6W6|R1AB_BCRP3               AMTQMYKQARSEDKRAKVTSAMQTMLFTMLRKLDNDALNNIINNARDGCVPLNIIPLTTA 4042
SP|P0C6V9|R1AB_BC279               AMTQMYKQARSEDKRAKVTSAMQTMLFTMLRKLDNDALNNIINNARDGCVPLNIIPLTTA 4050
TR|A0A0U1WHI4|A0A0U1WHI4_CVHSA     AMTQMYKQARSEDKRAKVTSAMQTMLFTMLRKLDNDALNNIINNARDGCVPLNIIPLTTA 4039
TR|A0A0U1WHG0|A0A0U1WHG0_CVHSA     AMTQMYKQARSEDKRAKVTSAMQTMLFTMLRKLDNDALNNIINNARDGCVPLNIIPLTTA 4039
TR|A0A166ZL34|A0A166ZL34_9NIDO     AMTQMYKQARSEDKRAKVTSAMQTMLFTMLRKLDNDALNNIINNARDGCVPLNIIPLTTA 3846
TR|R9QTB2|R9QTB2_CVHSA             AMTQMYKQARSEDKRAKVTSAMQTMLFTMLRKLDNDALNNIINNARDGCVPLNIIPLTTA 4036
TR|R9QTH2|R9QTH2_CVHSA             AMTQMYKQARSEDKRAKVTSAMQTMLFTMLRKLDNDALNNIINNARDGCVPLNIIPLTTA 4045
SP|P0C6U8|R1A_CVHSA                AMTQMYKQARSEDKRAKVTSAMQTMLFTMLRKLDNDALNNIINNARDGCVPLNIIPLTTA 4044
TR|Q6JH47|Q6JH47_CVHSA             AMTQMYKQARSEDKRAKVTSAMQTMLFTMLRKLDNDALNNIINNARDGCVPLNIIPLTTA 4044
TR|Q692E5|Q692E5_CVHSA             AMTQMYKQARSEDKRAKVTSAMQTMLFTMLRKLDNDALNNIINNARDGCVPLNIIPLTTA 4044
SP|P0C6F8|R1A_BCHK3                AMTQMYKQARSEDKRAKVTSAMQTMLFTMLRKLDNDALNNIINNARDGCVPLNIIPLTTA 4038
TR|A0A0K1Z0N1|A0A0K1Z0N1_CVHSA     AMTQMYKQARSEDKRAKVTSAMQTMLFTMLRKLDNDALNNIINNARDGCVPLNIIPLTTA 4044
SP|P0C6F5|R1A_BC279                AMTQMYKQARSEDKRAKVTSAMQTMLFTMLRKLDNDALNNIINNARDGCVPLNIIPLTTA 4050
SP|P0C6T7|R1A_BCRP3                AMTQMYKQARSEDKRAKVTSAMQTMLFTMLRKLDNDALNNIINNARDGCVPLNIIPLTTA 4042
                                   ************************************************************

QHN73794                           AKLMVVIPDYNTYKNTCDGTTFTYASALWEIQQVVDADSKIVQLSEISMDNSPNLAWPLI 4127
SP|P0C6X7|R1AB_CVHSA               AKLMVVVPDYGTYKNTCDGNTFTYASALWEIQQVVDADSKIVQLSEINMDNSPNLAWPLI 4104
TR|Q6UZF5|Q6UZF5_CVHSA             AKLMVVVPDYGTYKNTCDGNTFTYASALWEIQQVVDADSKIVQLSEINMDNSPNLAWPLI 4104
TR|Q6UZF1|Q6UZF1_CVHSA             AKLMVVVPDYGTYKNTCDGNTFTYASALWEIQQVVDADSKIVQLSEINMDNSPNLAWPLI 4104
TR|Q6JH48|Q6JH48_CVHSA             AKLMVVVPDYGTYKNTCDGNTFTYASALWEIQQVVDADSKIVQLSEINMDNSPNLAWPLI 4104
TR|Q692E6|Q692E6_CVHSA             AKLMVVVPDYGTYKNTCDGNTFTYASALWEIQQVVDADSKIVQLSEINMDNSPNLAWPLI 4104
TR|A0A0K1YZY7|A0A0K1YZY7_CVHSA     AKLMVVVPDYGTYKNTCDGNTFTYASALWEIQQVVDADSKIIQLSEINMDNSPNLAWPLI 4104
SP|P0C6W2|R1AB_BCHK3               AKLMVVVPDYGTYKNTCDGNTFTYASALWEIQQVVDADSKIVQLSEINMDNSPNLAWPLI 4098
SP|P0C6W6|R1AB_BCRP3               AKLMVVVPDYGTYKNTCDGNTFTYASALWEIQQVVDADSKIVQLSEINMENSSNLAWPLI 4102
SP|P0C6V9|R1AB_BC279               AKLMVVVPDYGTYKNTCDGNTFTYASALWEIQQVVDADSKIVQLSEINMDNSQNLAWPLI 4110
TR|A0A0U1WHI4|A0A0U1WHI4_CVHSA     AKLMVVVPDYGTYKNTCDGNTFTYASALWEIQQVVDADSKIVPLSEINMDNSPNLAWPLI 4099
TR|A0A0U1WHG0|A0A0U1WHG0_CVHSA     AKLMVVVPDYGTYKNTCDGNTFTYASALWEIQQVVDADSKIVPLSEINMDNSPNLAWPLI 4099
TR|A0A166ZL34|A0A166ZL34_9NIDO     AKLMVVVPDYGTYKNTCDGNTFTYASALWEIQQVVDADSKIVPLSEINMDNSPNLAWPLI 3906
TR|R9QTB2|R9QTB2_CVHSA             AKLMVVVPDYGTYKNTCDGNTFTYASALWEIQQVVDADSKIVQLSEINMDNSPNLAWPLI 4096
TR|R9QTH2|R9QTH2_CVHSA             AKLMVVVPDYGTYKNTCDGNTFTYASALWEIQQVVDADSKIVQLSEINMDNSPNLAWPLI 4105
SP|P0C6U8|R1A_CVHSA                AKLMVVVPDYGTYKNTCDGNTFTYASALWEIQQVVDADSKIVQLSEINMDNSPNLAWPLI 4104
TR|Q6JH47|Q6JH47_CVHSA             AKLMVVVPDYGTYKNTCDGNTFTYASALWEIQQVVDADSKIVQLSEINMDNSPNLAWPLI 4104
TR|Q692E5|Q692E5_CVHSA             AKLMVVVPDYGTYKNTCDGNTFTYASALWEIQQVVDADSKIVQLSEINMDNSPNLAWPLI 4104
SP|P0C6F8|R1A_BCHK3                AKLMVVVPDYGTYKNTCDGNTFTYASALWEIQQVVDADSKIVQLSEINMDNSPNLAWPLI 4098
TR|A0A0K1Z0N1|A0A0K1Z0N1_CVHSA     AKLMVVVPDYGTYKNTCDGNTFTYASALWEIQQVVDADSKIIQLSEINMDNSPNLAWPLI 4104
SP|P0C6F5|R1A_BC279                AKLMVVVPDYGTYKNTCDGNTFTYASALWEIQQVVDADSKIVQLSEINMDNSQNLAWPLI 4110
SP|P0C6T7|R1A_BCRP3                AKLMVVVPDYGTYKNTCDGNTFTYASALWEIQQVVDADSKIVQLSEINMENSSNLAWPLI 4102
                                   ******:***.********.*********************: ****.*:** *******

QHN73794                           VTALRANSAVKLQNNELSPVALRQMSCAAGTTQTACTDDNALAYYNTTKGGRFVLALLSD 4187
SP|P0C6X7|R1AB_CVHSA               VTALRANSAVKLQNNELSPVALRQMSCAAGTTQTACTDDNALAYYNNSKGGRFVLALLSD 4164
TR|Q6UZF5|Q6UZF5_CVHSA             VTALRANSAVKLQNNELSPVALRQMSCAAGTTQTACTDDNALAYYNNSKGGRFVLALLSD 4164
TR|Q6UZF1|Q6UZF1_CVHSA             VTALRANSAVKLQNNELSPVALRQMSCAAGTTQTACTDDNALAYYNNSKGGRFVLALLSD 4164
TR|Q6JH48|Q6JH48_CVHSA             VTALRANSAVKLQNNELSPVALRQMSCAAGTTQTACTDDNALAYYNNSKGGRFVLALLSD 4164
TR|Q692E6|Q692E6_CVHSA             VTALRANSAVKLQNNELSPVALRQMSCAAGTTQTACTDDNALAYYNNSKGGRFVLALLSD 4164
TR|A0A0K1YZY7|A0A0K1YZY7_CVHSA     VTALRANSAVKLQNNELSPVALRQMSCAAGTTQTACTDDNALAYYNNSKGGRFVLALLSD 4164
SP|P0C6W2|R1AB_BCHK3               VTALRANSAVKLQNNELSPVALRQMSCAAGTTQTACTDDNALAYYNNAKGGRFVLALLSD 4158
SP|P0C6W6|R1AB_BCRP3               VTALRANSAVKLQNNELSPVALRQMSCAAGTTQTACTDDNALAYYNNSKGGRFVLALLSD 4162
SP|P0C6V9|R1AB_BC279               VTALRANSAVKLQNNELSPVALRQMSCAAGTTQTACTDDNALAYYNNSKGGRFVLALLSD 4170
TR|A0A0U1WHI4|A0A0U1WHI4_CVHSA     VTALRANSAVKLQNNELSPVALRQMSCAAGTTQTACTDDNALAYYNNSKGGRFVLALLSD 4159
TR|A0A0U1WHG0|A0A0U1WHG0_CVHSA     VTALRANSAVKLQNNELSPVALRQMSCAAGTTQTACTDDNALAYYNNSKGGRFVLALLSD 4159
TR|A0A166ZL34|A0A166ZL34_9NIDO     VTALRANSAVKLQNNELSPVALRQMSCAAGTTQTACTDDNALAYYNNSKGGRFVLALLSD 3966
TR|R9QTB2|R9QTB2_CVHSA             VTALRANSAVKLQNNELSPVALRQMSCAAGTTQTACTDDNALAYYNNSKGGRFVLALLSD 4156
TR|R9QTH2|R9QTH2_CVHSA             VTALRANSAVKLQNNELSPVALRQMSCAAGTTQTACTDDNALAYYNNSKGGRFVLALLSD 4165
SP|P0C6U8|R1A_CVHSA                VTALRANSAVKLQNNELSPVALRQMSCAAGTTQTACTDDNALAYYNNSKGGRFVLALLSD 4164
TR|Q6JH47|Q6JH47_CVHSA             VTALRANSAVKLQNNELSPVALRQMSCAAGTTQTACTDDNALAYYNNSKGGRFVLALLSD 4164
TR|Q692E5|Q692E5_CVHSA             VTALRANSAVKLQNNELSPVALRQMSCAAGTTQTACTDDNALAYYNNSKGGRFVLALLSD 4164
SP|P0C6F8|R1A_BCHK3                VTALRANSAVKLQNNELSPVALRQMSCAAGTTQTACTDDNALAYYNNAKGGRFVLALLSD 4158
TR|A0A0K1Z0N1|A0A0K1Z0N1_CVHSA     VTALRANSAVKLQNNELSPVALRQMSCAAGTTQTACTDDNALAYYNNSKGGRFVLALLSD 4164
SP|P0C6F5|R1A_BC279                VTALRANSAVKLQNNELSPVALRQMSCAAGTTQTACTDDNALAYYNNSKGGRFVLALLSD 4170
SP|P0C6T7|R1A_BCRP3                VTALRANSAVKLQNNELSPVALRQMSCAAGTTQTACTDDNALAYYNNSKGGRFVLALLSD 4162
                                   **********************************************.:************
```

```
QHN73794                          LQDLKWARFPKSDGTGTIYTELEPPCRFVTDTPKGPKVKYLYFIKGLNNLNRGMVLGSLA 4247
SP|P0C6X7|R1AB_CVHSA              HQDLKWARFPKSDGTGTIYTELEPPCRFVTDTPKGPKVKYLYFIKGLNNLNRGMVLGSLA 4224
TR|Q6UZF5|Q6UZF5_CVHSA            HQDLKWARFPKSDGTGTIYTELEPPCRFVTDTPKGPKVKYLYFIKGLNNLNRGMVLGSLA 4224
TR|Q6UZF1|Q6UZF1_CVHSA            HQDLKWARFPKSDGTGTIYTELEPPCRFVTDTPKGPKVKYLYFIKGLNNLNRGMVLGSLA 4224
TR|Q6JH48|Q6JH48_CVHSA            HQDLKWARFPKSDGTGTIYTELEPPCRFVTDTPKGPKVKYLYFIKGLNNLNRGMVLGSLA 4224
TR|Q692E6|Q692E6_CVHSA            HQDLKWARFPKSDGTGTIYTELEPPCRFVTDTPKGPKVKYLYFIKGLNNLNRGMVLGSLA 4224
TR|A0A0K1YZY7|A0A0K1YZY7_CVHSA    HQDLKWARFPKSDGTGTIYTELEPPCRFVTDTPKGPKVKYLYFIKGLNNLNRGMVLGSLA 4224
SP|P0C6W2|R1AB_BCHK3              HQDLKWARFPKSDGTGTIYTELEPPCRFVTDTPKGPKVKYLYFIKGLNNLNRGMVLGSLA 4218
SP|P0C6W6|R1AB_BCRP3              HQDLKWARFPKSDGTGTIYTELEPPCRFVTDTPKGPKVKYLHFIKGLNNLNRGMVLGSLA 4222
SP|P0C6V9|R1AB_BC279              HQDLKWARFPKSDGTGTIYTELEPPCRFVTDTPRGPKVKYLYFIKGLNNLNRGMVLGSLA 4230
TR|A0A0U1WHI4|A0A0U1WHI4_CVHSA    HQDLKWARFPKSDGTGTIYTELEPPCRFVTDTPKGPKVKYLYFIKGLNNLNRGMVLGSLA 4219
TR|A0A0U1WHG0|A0A0U1WHG0_CVHSA    HQDLKWARFPKSDGTGTIYTELEPPCRFVTDTPKGPKVKYLYFIKGLNNLNRGMVLGSLA 4219
TR|A0A166ZL34|A0A166ZL34_9NIDO    HQDLKWARFPKSDGTGTIYTELEPPCRFVTDTPKGPKVKYLYFIKGLNNLNRGMVLGSLA 4026
TR|R9QTB2|R9QTB2_CVHSA            HQDLKWARFPKSDGTGTIYTELEPPCRFVTDTPKGPKVKYLYFIKGLNNLNRGMVLGSLA 4216
TR|R9QTH2|R9QTH2_CVHSA            HQDLKWARFPKSDGSGTIYTELEPPCRFVTDTPKGPKVKYLYFIKGLNNLNRGMVLGSLA 4225
SP|P0C6U8|R1A_CVHSA               HQDLKWARFPKSDGTGTIYTELEPPCRFVTDTPKGPKVKYLYFIKGLNNLNRGMVLGSLA 4224
TR|Q6JH47|Q6JH47_CVHSA            HQDLKWARFPKSDGTGTIYTELEPPCRFVTDTPKGPKVKYLYFIKGLNNLNRGMVLGSLA 4224
TR|Q692E5|Q692E5_CVHSA            HQDLKWARFPKSDGTGTIYTELEPPCRFVTDTPKGPKVKYLYFIKGLNNLNRGMVLGSLA 4224
SP|P0C6F8|R1A_BCHK3               HQDLKWARFPKSDGTGTIYTELEPPCRFVTDTPKGPKVKYLYFIKGLNNLNRGMVLGSLA 4218
TR|A0A0K1Z0N1|A0A0K1Z0N1_CVHSA    HQDLKWARFPKSDGTGTIYTELEPPCRFVTDTPKGPKVKYLYFIKGLNNLNRGMVLGSLA 4224
SP|P0C6F5|R1A_BC279               HQDLKWARFPKSDGTGTIYTELEPPCRFVTDTPRGPKVKYLYFIKGLNNLNRGMVLGSLA 4230
SP|P0C6T7|R1A_BCRP3               HQDLKWARFPKSDGTGTIYTELEPPCRFVTDTPKGPKVKYLHFIKGLNNLNRGMVLGSLA 4222
                                   *************:******************:*******:******************

QHN73794                          ATVRLQAGNATEVPANSTVLSFCAFAVDAAKAYKDYLASGGQPITNCVKMLCTHTGTGQA 4307
SP|P0C6X7|R1AB_CVHSA              ATVRLQAGNATEVPANSTVLSFCAFAVDPAKAYKDYLASGGQPITNCVKMLCTHTGTGQA 4284
TR|Q6UZF5|Q6UZF5_CVHSA            ATVRLQAGNATEVPANSTVLSFCAFAVDPAKAYKDYLASGGQPITNCVKMLCTHTGTGQA 4284
TR|Q6UZF1|Q6UZF1_CVHSA            ATVRLQAGNATEVPANSTVLSFCAFAVDPAKAYKDYLASGGQPITNCVKMLCTHTGTGQA 4284
TR|Q6JH48|Q6JH48_CVHSA            ATVRLQAGNATEVPANSTVLSFCAFAVDPAKAYKDYLASGGQPITNCVKMLCTHTGTGQA 4284
TR|Q692E6|Q692E6_CVHSA            ATVRLQAGNATEVPANSTVLSFCAFAVDPAKAYKDYLASGGQPITNCVKMLCTHTGTGQA 4284
TR|A0A0K1YZY7|A0A0K1YZY7_CVHSA    ATVRLQAGNATEVPANSTVLSFCAFAVDPAKAYKDYLASGGQPITNCVKMLCTHTGTGQA 4284
SP|P0C6W2|R1AB_BCHK3              ATVRLQAGNATEVPANSTVLSFCAFAVDPAKAYKDYLASGGQPITNCVKMLCTHTGTGQA 4278
SP|P0C6W6|R1AB_BCRP3              ATVRLQAGNATEVPANSTVLSFCAFAVDPAKAYKDYLASGGQPITNCVKMLCTHTGTGQA 4282
SP|P0C6V9|R1AB_BC279              ATVRLQAGNATEVPANSAVLSFCAFAVDPAKAYKDYLASGGQPITNCVKMLCTHTGTGQA 4290
TR|A0A0U1WHI4|A0A0U1WHI4_CVHSA    ATVRLQAGNATEVPANSTVLSFCAFAVDPAKAYKDYLSSGGQPITNCVKMLCTHTGTGQA 4279
TR|A0A0U1WHG0|A0A0U1WHG0_CVHSA    ATVRLQAGNATEVPANSTVLSFCAFAVDPAKAYKDYLSSGGQPITNCVKMLCTHTGTGQA 4279
TR|A0A166ZL34|A0A166ZL34_9NIDO    ATVRLQAGNATEVPANSTVLSFCAFAVDPAKAYKDYLSSGGQPITNCVKMLCTHTGTGQA 4086
TR|R9QTB2|R9QTB2_CVHSA            ATVRLQAGNATEVPANSTVLSFCAFAVDPAKAYKDYLASGGQPITNCVKMLCTHTGTGQA 4276
TR|R9QTH2|R9QTH2_CVHSA            ATVRLQAGNATEVPANSTVLSFCAFAVDPAKAYKDYLASGGQPITNCVKMLCTHTGTGQA 4285
SP|P0C6U8|R1A_CVHSA               ATVRLQAGNATEVPANSTVLSFCAFAVDPAKAYKDYLASGGQPITNCVKMLCTHTGTGQA 4284
TR|Q6JH47|Q6JH47_CVHSA            ATVRLQAGNATEVPANSTVLSFCAFAVDPAKAYKDYLASGGQPITNCVKMLCTHTGTGQA 4284
TR|Q692E5|Q692E5_CVHSA            ATVRLQAGNATEVPANSTVLSFCAFAVDPAKAYKDYLASGGQPITNCVKMLCTHTGTGQA 4284
SP|P0C6F8|R1A_BCHK3               ATVRLQAGNATEVPANSTVLSFCAFAVDPAKAYKDYLASGGQPITNCVKMLCTHTGTGQA 4278
TR|A0A0K1Z0N1|A0A0K1Z0N1_CVHSA    ATVRLQAGNATEVPANSTVLSFCAFAVDPAKAYKDYLASGGQPITNCVKMLCTHTGTGQA 4284
SP|P0C6F5|R1A_BC279               ATVRLQAGNATEVPANSAVLSFCAFAVDPAKAYKDYLASGGQPITNCVKMLCTHTGTGQA 4290
SP|P0C6T7|R1A_BCRP3               ATVRLQAGNATEVPANSTVLSFCAFAVDPAKAYKDYLASGGQPITNCVKMLCTHTGTGQA 4282
                                  *****************:********** ********:**********************

QHN73794                          ITVTPEANMDQESFGGASCCLYCRCHIDHPNPKGFCDLKGKYVQIPTTCANDPVGFTLKN 4367
SP|P0C6X7|R1AB_CVHSA              ITVTPEANMDQESFGGASCCLYCRCHIDHPNPKGFCDLKGKYVQIPTTCANDPVGFTLRN 4344
TR|Q6UZF5|Q6UZF5_CVHSA            ITVTPEANMDQESFGGASCCLYCRCHIDHPNPKGFCDLKGKYVQIPTTCANDPVGFTLRN 4344
TR|Q6UZF1|Q6UZF1_CVHSA            ITVTPEANMDQESFGGASCCLYCRCHIDHPNPKGFCDLKGKYVQIPTTCANDPVGFTLRN 4344
TR|Q6JH48|Q6JH48_CVHSA            ITVTPEANMDQESFGGASCCLYCRCHIDHPNPKGFCDLKGKYVQIPTTCANDPVGFTLRN 4344
TR|Q692E6|Q692E6_CVHSA            ITVTPEANMDQESFGGASCCLYCRCHIDHPNPKGFCDLKGKYVQIPTTCANDPVGFTLRN 4344
TR|A0A0K1YZY7|A0A0K1YZY7_CVHSA    ITVTPEANMDQESFGGASCCLYCRCHIDHPNPKGFCDLKGKYIQIPTTCANDPVGFTLRN 4344
SP|P0C6W2|R1AB_BCHK3              ITVTPEANMDQESFGGASCCLYCRCHIDHPNPKGFCDLKGKYVQIPTTCANDPVGFTLRN 4338
SP|P0C6W6|R1AB_BCRP3              ITVTPEANMDQESFGGASCCLYCRCHIDHPNPKGFCDLKGKYVQIPTTCANDPVGFTLRN 4342
SP|P0C6V9|R1AB_BC279              ITVTPEANMDQESFGGASCCLYCRCHIDHPNPKGFCDLKGKYVQIPATCANDPVGFTLKN 4350
TR|A0A0U1WHI4|A0A0U1WHI4_CVHSA    ITVTPEANMDQESFGGASCCLYCRCHIDHPNPKGFCDLKGKYVQIPTTCANDPVGFTLRN 4339
TR|A0A0U1WHG0|A0A0U1WHG0_CVHSA    ITVTPEANMDQESFGGASCCLYCRCHIDHPNPKGFCDLKGKYVQIPTTCANDPVGFTLRN 4339
TR|A0A166ZL34|A0A166ZL34_9NIDO    ITVTPEANMDQESFGGASCCLYCRCHIDHPNPKGFCDLKGKYVQIPTTCANDPVGFTLRN 4146
TR|R9QTB2|R9QTB2_CVHSA            ITVTPEANMDQESFGGASCCLYCRCHIDHPNPKGFCDLKGKYVQIPTTCANDPVGFTLRN 4336
TR|R9QTH2|R9QTH2_CVHSA            ITVTPEANMDQESFGGASCCLYCRCHIDHPNPKGFCDLKGKYVQIPTTCANDPVGFTLRN 4345
SP|P0C6U8|R1A_CVHSA               ITVTPEANMDQESFGGASCCLYCRCHIDHPNPKGFCDLKGKYVQIPTTCANDPVGFTLRN 4344
TR|Q6JH47|Q6JH47_CVHSA            ITVTPEANMDQESFGGASCCLYCRCHIDHPNPKGFCDLKGKYVQIPTTCANDPVGFTLRN 4344
TR|Q692E5|Q692E5_CVHSA            ITVTPEANMDQESFGGASCCLYCRCHIDHPNPKGFCDLKGKYVQIPTTCANDPVGFTLRN 4344
SP|P0C6F8|R1A_BCHK3               ITVTPEANMDQESFGGASCCLYCRCHIDHPNPKGFCDLKGKYVQIPTTCANDPVGFTLRN 4338
TR|A0A0K1Z0N1|A0A0K1Z0N1_CVHSA    ITVTPEANMDQESFGGASCCLYCRCHIDHPNPKGFCDLKGKYIQIPTTCANDPVGFTLRN 4344
SP|P0C6F5|R1A_BC279               ITVTPEANMDQESFGGASCCLYCRCHIDHPNPKGFCDLKGKYVQIPATCANDPVGFTLKN 4350
SP|P0C6T7|R1A_BCRP3               ITVTPEANMDQESFGGASCCLYCRCHIDHPNPKGFCDLKGKYVQIPTTCANDPVGFTLRN 4342
                                  ******************************************:***:***********:*
```

```
QHN73794                            TVCTVCGMWKGYGCSCDQLREPMLQSADAQSFLNRVCGVSAARLTPCGTGTSTDVVYRAF 4427
SP|P0C6X7|R1AB_CVHSA                TVCTVCGMWKGYGCSCDQLREPLMQSADASTFLNRVCGVSAARLTPCGTGTSTDVVYRAF 4404
TR|Q6UZF5|Q6UZF5_CVHSA              TVCTVCGMWKGYGCSCDQLREPLMQSADASTFLNRVCGVSAARLTPCGTGTSTDVVYRAF 4404
TR|Q6UZF1|Q6UZF1_CVHSA              TVCTVCGMWKGYGCSCDQLREPLMQSADASTFLNRVCGVSAARLTPCGTGTSTDVVYRAF 4404
TR|Q6JH48|Q6JH48_CVHSA              TVCTVCGMWKGYGCSCDQLREPLMQSADASTFLNRVCGVSAARLTPCGTGTSTDVVYRAF 4404
TR|Q692E6|Q692E6_CVHSA              TVCTVCGMWKGYGCSCDQLREPLMQSADASTFFKRVCGVSAARLTPCGTGTSTDVVYRAF 4404
TR|A0A0K1YZY7|A0A0K1YZY7_CVHSA      TVCTVCGMWKGYGCSCDQLREPMMQSADASTFLNRVCGVSAARLTPCGTGISTDVVYRAF 4404
SP|P0C6W2|R1AB_BCHK3                TVCTVCGMWKGYGCSCDQLREPMMQSADASTFLNRVCGVSAARLTPCGTGTSTDVVYRAF 4398
SP|P0C6W6|R1AB_BCRP3                TVCTVCGMWKGYGCSCDQLREPMMQSADASTFLNRVCGVSAARLTPCGTGISTDVVYRAF 4402
SP|P0C6V9|R1AB_BC279                TVCTVCGTWKGYGCSCDQLREPMMQSADASTFLNRVCGVSAARLTPCGTGTSTDVVYRAF 4410
TR|A0A0U1WHI4|A0A0U1WHI4_CVHSA      TVCTVCGMWKGYGCSCDQLREPMMQSADASAFLNRVCGVSAARLTPCGTGTSTDVVYRAF 4399
TR|A0A0U1WHG0|A0A0U1WHG0_CVHSA      TVCTVCGMWKGYGCSCDQLREPMMQSADASPFLNRVCGVSAARLTPCGTGTSTDVVYRAF 4399
TR|A0A166ZL34|A0A166ZL34_9NIDO      TVCTVCGMWKGYGCSCDQLREPMMQSADASPFLNRVCGVSAARLTPCGTGTSTDVVYRAF 4206
TR|R9QTB2|R9QTB2_CVHSA              TVCTVCGMWKGYGCSCDQLREPMMQSADASTFLNGFAV---------------------- 4374
TR|R9QTH2|R9QTH2_CVHSA              TVCTVCGMWKGYGCSCDQLREPMMQSADASTFLNGFAV---------------------- 4383
SP|P0C6U8|R1A_CVHSA                 TVCTVCGMWKGYGCSCDQLREPLMQSADASTFLNGFAV---------------------- 4382
TR|Q6JH47|Q6JH47_CVHSA              TVCTVCGMWKGYGCSCDQLREPLMQSADASTFLNGFAV---------------------- 4382
TR|Q692E5|Q692E5_CVHSA              TVCTVCGMWKGYGCSCDQLREPLMQSADASTFLNGFAV---------------------- 4382
SP|P0C6F8|R1A_BCHK3                 TVCTVCGMWKGYGCSCDQLREPMMQSADASTFLNGFAV---------------------- 4376
TR|A0A0K1Z0N1|A0A0K1Z0N1_CVHSA      TVCTVCGMWKGYGCSCDQLREPMMQSADASTFLNGFAV---------------------- 4382
SP|P0C6F5|R1A_BC279                 TVCTVCGTWKGYGCSCDQLREPMMQSADASTFLNGFAV---------------------- 4388
SP|P0C6T7|R1A_BCRP3                 TVCTVCGMWKGYGCSCDQLREPMMQSADASTFLNGFAV---------------------- 4380
                                    ******* **************::*****. *:: ..

QHN73794                            DIYNDKVAGFAKFLKTNCCRFQEKDEDDNLIDSYFVVKRHTFSNYQHEETIYNLLKDCPA 4487
SP|P0C6X7|R1AB_CVHSA                DIYNEKVAGFAKFLKTNCCRFQEKDEEGNLLDSYFVVKRHTMSNYQHEETIYNLVKDCPA 4464
TR|Q6UZF5|Q6UZF5_CVHSA              DIYNEKVAGFAKFLKTNCCRFQEKDEEGNLLDSYFVVKRHTMSNYQHEETIYNLVKDCPA 4464
TR|Q6UZF1|Q6UZF1_CVHSA              DIYNEKVAGFAKFLKTNCCRFQEKDEEGNLLDSYFVVKRHTMSNYQHEETIYNLVKDCPA 4464
TR|Q6JH48|Q6JH48_CVHSA              DIYNEKVAGFAKFLKTNCCRFQEKDEEGNLLDSYFVVKRHTMSNYQHEETIYNLVKDCPA 4464
TR|Q692E6|Q692E6_CVHSA              DIYNEKVAGFAKFLKTNCCRFQEKDEEGNLLDSYFVVKRHTMSNYQHEETIYNLVKDCPA 4464
TR|A0A0K1YZY7|A0A0K1YZY7_CVHSA      DIYNEKVAGFAKFLKTNCCRFQEKDEEGNLLDSYFVVKRHTMSNYQHEETIYNLVKDCPA 4464
SP|P0C6W2|R1AB_BCHK3                DIYNEKVAGFAKFLKTNCCRFQEKDEEGNLLDSYFVVKRHTMSNYQHEETIYNLIKECPA 4458
SP|P0C6W6|R1AB_BCRP3                DIYNEKVAGFAKFLKTNCCRFQEKDEEGNLLDSYFVVKRHTMSNYQHEETIYNLVKDCPA 4462
SP|P0C6V9|R1AB_BC279                DIYNERVAGFAKFLKTNCCRFQEKDEEGNLLDSYFVVKRHTMSNYQHEETIYNLVKECPA 4470
TR|A0A0U1WHI4|A0A0U1WHI4_CVHSA      DIYNEKVAGFAKFLKTNCCRFQEKDEEGNLIDSYFVVKRHTMSNYQHEEAIYNLLKECPA 4459
TR|A0A0U1WHG0|A0A0U1WHG0_CVHSA      DIYNEKVAGFAKFLKTNCCRFQEMDEDGNLIDSYFVVKRHTMSNYQHEEAIYNLLKECPA 4459
TR|A0A166ZL34|A0A166ZL34_9NIDO      DIYNEKVAGFAKFLKTNCCRFQEMDEDGNLIDSYFVVKRHTMSNYQHEEAIYNLLKECPA 4266
TR|R9QTB2|R9QTB2_CVHSA              ------------------------------------------------------------
TR|R9QTH2|R9QTH2_CVHSA              ------------------------------------------------------------
SP|P0C6U8|R1A_CVHSA                 ------------------------------------------------------------
TR|Q6JH47|Q6JH47_CVHSA              ------------------------------------------------------------
TR|Q692E5|Q692E5_CVHSA              ------------------------------------------------------------
SP|P0C6F8|R1A_BCHK3                 ------------------------------------------------------------
TR|A0A0K1Z0N1|A0A0K1Z0N1_CVHSA      ------------------------------------------------------------
SP|P0C6F5|R1A_BC279                 ------------------------------------------------------------
SP|P0C6T7|R1A_BCRP3                 ------------------------------------------------------------


QHN73794                            VAKHDFFKFRIDGDMVPHISRQRLTKYTMADLVYALRHFDEGNCDTLKEILVTYNCCDDD 4547
SP|P0C6X7|R1AB_CVHSA                VAVHDFFKFRVDGDMVPHISRQRLTKYTMADLVYALRHFDEGNCDTLKEILVTYNCCDDD 4524
TR|Q6UZF5|Q6UZF5_CVHSA              VAVHDFFKFRVDGDMVPHISRQRLTKYTMADLVYALRHFDEGNCDTLKEILVTYNCCDDD 4524
TR|Q6UZF1|Q6UZF1_CVHSA              VAVHDFFKFRVDGDMVPHISRQRLTKYTMADLVYALRHFDEGNCDTLKEILVTYNCCDDD 4524
TR|Q6JH48|Q6JH48_CVHSA              VAVHDFFKFRVDGDMVPHISRQRLTKYTMADLVYALRHFDEGNCDTLKEILVTYNCCDDD 4524
TR|Q692E6|Q692E6_CVHSA              VAVHDFFKFRVDGDMVPHISRQRLTKYTMADLVYALRHFDEGNCDTLKEILVTYNCCDDD 4524
TR|A0A0K1YZY7|A0A0K1YZY7_CVHSA      VAVHDFFKFRVDGDMVPHISRQRLTKYTMADLVYALRHFDEGNCDTLKEILVTYNCCDDD 4524
SP|P0C6W2|R1AB_BCHK3                VAVHDFFKFRVDGDMVPHISRQRLTKYTMADLVYALRHFDEGNCDTLKEILVTYNCCDDN 4518
SP|P0C6W6|R1AB_BCRP3                VAVHDFFKFRVDGDMVPHISRQRLTKYTMADLVYALRHFDEGNCDTLKEILVTYNCCDDD 4522
SP|P0C6V9|R1AB_BC279                VAVHDFFKFRVDGDMVPHISRQRLTKYTMADLVYALRHFDEGNCDTLKEILVTYNCCDDD 4530
TR|A0A0U1WHI4|A0A0U1WHI4_CVHSA      VAVHDFFKFRVDGDMVPHISRQRLTKYTMADLVYALRHFDEGNCDTLKEILVTYNCCDDD 4519
TR|A0A0U1WHG0|A0A0U1WHG0_CVHSA      VAVHDFFKFRVDGDMVPHISRQRLTKYTMADLVYALRHFDEGNCDTLKEILVTYNCCDDD 4519
TR|A0A166ZL34|A0A166ZL34_9NIDO      VAVHDFFKFRVDGDMVPHISRQRLTKYTMADLVYALRHFDEGNCDTLKEILVTYNCCDDD 4326
TR|R9QTB2|R9QTB2_CVHSA              ------------------------------------------------------------
TR|R9QTH2|R9QTH2_CVHSA              ------------------------------------------------------------
SP|P0C6U8|R1A_CVHSA                 ------------------------------------------------------------
TR|Q6JH47|Q6JH47_CVHSA              ------------------------------------------------------------
TR|Q692E5|Q692E5_CVHSA              ------------------------------------------------------------
SP|P0C6F8|R1A_BCHK3                 ------------------------------------------------------------
TR|A0A0K1Z0N1|A0A0K1Z0N1_CVHSA      ------------------------------------------------------------
SP|P0C6F5|R1A_BC279                 ------------------------------------------------------------
SP|P0C6T7|R1A_BCRP3                 ------------------------------------------------------------
```

```
QHN73794                          YFNKKDWYDFVENPDILRVYANLGERVRQALLKTVQFCDAMRNAGIVGVLTLDNQDLNGN 4607
SP|P0C6X7|R1AB_CVHSA              YFNKKDWYDFVENPDILRVYANLGERVRQSLLKTVQFCDAMRDAGIVGVLTLDNQDLNGN 4584
TR|Q6UZF5|Q6UZF5_CVHSA            YFNKKDWYDFVENPDILRVYANLGERVRQSLLKTVQFCDAMRDAGIVGVLTLDNQDLNGN 4584
TR|Q6UZF1|Q6UZF1_CVHSA            YFNKKDWYDFVENPDILRVYANLGERVRQSLLKTVQFCDAMRDAGIVGVLTLDNQDLNGN 4584
TR|Q6JH48|Q6JH48_CVHSA            YFNKKDWYDFVENPDILRVYANLGERVRQSLLKTVQFCDAMRDAGIVGVLTLDNQDLNGN 4584
TR|Q692E6|Q692E6_CVHSA            YFNKKDWYDFVENPDILRVYANLGERVRQSLLKTVQFCDAMRDAGIVGVLTLDNQDLNGN 4584
TR|A0A0K1YZY7|A0A0K1YZY7_CVHSA    YFNKKDWYDFVENPDILRVYANLGERVRQALLKTVQFCDAMRDAGIVGVLTLDNQDLNGN 4584
SP|P0C6W2|R1AB_BCHK3              YFNKKDWYDFVENPDVLRVYANLGERVRRALLKTVQFCDAMRDAGIVGVLTLDNQDLNGN 4578
SP|P0C6W6|R1AB_BCRP3              YFNKKDWYDFVENPDILRVYANLGERVRQALLKTVQFCDAMRDAGIVGVLTLDNQDLNGN 4582
SP|P0C6V9|R1AB_BC279              YFNKKDWYDFVENPDILRVYANLGERVRQALLKTVQFCDAMRDAGIVGVLTLDNQDLNGN 4590
TR|A0A0U1WHI4|A0A0U1WHI4_CVHSA    YFNKKDWYDFVENPDILRVYANLGERVRQALLKTVQFCDAMRDAGIVGVLTLDNQDLNGN 4579
TR|A0A0U1WHG0|A0A0U1WHG0_CVHSA    YFNKKDWYDFVENPDILRVYANLGERVRQALLKTVQFCDAMRDAGIVGVLTLDNQDLNGN 4579
TR|A0A166ZL34|A0A166ZL34_9NIDO    YFNKKDWYDFVENPDILRVYANLGERVRQALLKTVQFCDAMRDAGIVGVLTLDNQDLNGN 4386
TR|R9QTB2|R9QTB2_CVHSA            ------------------------------------------------------------
TR|R9QTH2|R9QTH2_CVHSA            ------------------------------------------------------------
SP|P0C6U8|R1A_CVHSA               ------------------------------------------------------------
TR|Q6JH47|Q6JH47_CVHSA            ------------------------------------------------------------
TR|Q692E5|Q692E5_CVHSA            ------------------------------------------------------------
SP|P0C6F8|R1A_BCHK3               ------------------------------------------------------------
TR|A0A0K1Z0N1|A0A0K1Z0N1_CVHSA    ------------------------------------------------------------
SP|P0C6F5|R1A_BC279               ------------------------------------------------------------
SP|P0C6T7|R1A_BCRP3               ------------------------------------------------------------

QHN73794                          WYDFGDFIQTTPGSGVPVVDSYYSLLMPILTLTRALTAESHVDTDLTKPYIKWDLLKYDF 4667
SP|P0C6X7|R1AB_CVHSA              WYDFGDFVQVAPGCGVPIVDSYYSLLMPILTLTRALAAESHMDADLAKPLIKWDLLKYDF 4644
TR|Q6UZF5|Q6UZF5_CVHSA            WYDFGDFVQVAPGCGVPIVDSYYSLLMPILTLTRALAAESHMDADLAKPLIKWDLLKYDF 4644
TR|Q6UZF1|Q6UZF1_CVHSA            WYDFGDFVQVAPGCGVPIVDSYYSLLMPILTLTRALAAESHMDADLAKPLIKWDLLKYDF 4644
TR|Q6JH48|Q6JH48_CVHSA            WYDFGDFVQVAPGCGVPIVDSYYSLLMPILTLTRALAAESHMDADLAKPLIKWDLLKYDF 4644
TR|Q692E6|Q692E6_CVHSA            WYDFGDFVQVAPGCGVPIVDSYYSLLMPILTLTRALAAESHMDADLAKPLIKWDLLKYDF 4644
TR|A0A0K1YZY7|A0A0K1YZY7_CVHSA    WYDFGDFVQVAPGCGVPIVDSYYSLLMPILTMTRALAAESHMDADLAKPLIKWDLLKYDF 4644
SP|P0C6W2|R1AB_BCHK3              WYDFGDFVQVAPGCGVPIVDSYYSLLMPILTLTKALAAESHMDADLAKPLVKWDLLKYDF 4638
SP|P0C6W6|R1AB_BCRP3              WYDFGDFVQVAPGCGVPIVDSYYSLLMPILTLTRALAAESHMDADLAKPLIKWDLLKYDF 4642
SP|P0C6V9|R1AB_BC279              WYDFGDFVQVAPGCGVPIVDSYYSLLMPILTLTKALAAESHMDADLAKPLIKWDLLKYDF 4650
TR|A0A0U1WHI4|A0A0U1WHI4_CVHSA    WYDFGDFVQVAPGCGVPIVDSYYSLLMPILTLTRALAAESHMDADLTKPLIKWDLLKYDF 4639
TR|A0A0U1WHG0|A0A0U1WHG0_CVHSA    WYDFGDFVQVTPGCGVPIVDSYYSLLMPILTLTRALAAESHMDTDLTKPLIKWDLLKYDF 4639
TR|A0A166ZL34|A0A166ZL34_9NIDO    WYDFGDFVQVTPGCGVPIVDSYYSLLMPILTLTRALAAESHMDTDLTKPLIKWDLLKYDF 4446
TR|R9QTB2|R9QTB2_CVHSA            ------------------------------------------------------------
TR|R9QTH2|R9QTH2_CVHSA            ------------------------------------------------------------
SP|P0C6U8|R1A_CVHSA               ------------------------------------------------------------
TR|Q6JH47|Q6JH47_CVHSA            ------------------------------------------------------------
TR|Q692E5|Q692E5_CVHSA            ------------------------------------------------------------
SP|P0C6F8|R1A_BCHK3               ------------------------------------------------------------
TR|A0A0K1Z0N1|A0A0K1Z0N1_CVHSA    ------------------------------------------------------------
SP|P0C6F5|R1A_BC279               ------------------------------------------------------------
SP|P0C6T7|R1A_BCRP3               ------------------------------------------------------------

QHN73794                          TEERLKLFDRYFKYWDQTYHPNCVNCLDDRCILHCANFNVLFSTVFPPTSFGPLVRKIFV 4727
SP|P0C6X7|R1AB_CVHSA              TEERLCLFDRYFKYWDQTYHPNCINCLDDRCILHCANFNVLFSTVFPPTSFGPLVRKIFV 4704
TR|Q6UZF5|Q6UZF5_CVHSA            TEERLCLFDRYFKYWDQTYHPNCINCLDDRCILHCANFNVLFSTVFPPTSFGPLVRKIFV 4704
TR|Q6UZF1|Q6UZF1_CVHSA            TEERLCLFDRYFKYWDQTYHPNCINCLDDRCILHCANFNVLFSTVFPPTSFGPLVRKIFV 4704
TR|Q6JH48|Q6JH48_CVHSA            TEERLCLFDRYFKYWDQTYHPNCINCLDDRCILHCANFNVLFSTVFPPTSFGPLVRKIFV 4704
TR|Q692E6|Q692E6_CVHSA            TEERLCLFDRYFKYWDQTYHPNCINCLDDRCILHCANFNVLFSTVFPPTSFGPLVRKIFV 4704
TR|A0A0K1YZY7|A0A0K1YZY7_CVHSA    TEERLCLFDRYFKYWDQTYHPNCINCLDDRCILHCANFNVLFSTVFPPTSFGPLVRKIFV 4704
SP|P0C6W2|R1AB_BCHK3              TEERLCLFDRYFKYWDQTYHPNCINCLDDRCILHCANFNVLFSTVFPPTSFGPLVRKIFV 4698
SP|P0C6W6|R1AB_BCRP3              TAERLCLFDRYFKYWDQTYHPNCINCLDDRCILHCANFNVLFSTVFPPTSFGPLVRKIFV 4702
SP|P0C6V9|R1AB_BC279              TEERLCLFDRYFKYWDQTYHPNCINCLDDRCILHCANFNVLFSTVFPPTSFGPLVRKIFV 4710
TR|A0A0U1WHI4|A0A0U1WHI4_CVHSA    TEERLCLFDRYFKYWDQTYHPNCVNCLDDRCILHCANFNVLFSTVFPPTSFGPLVRKIFV 4699
TR|A0A0U1WHG0|A0A0U1WHG0_CVHSA    TEERLCLFDRYFKYWDQTYHPNCINCLDDRCILHCANFNVLFSTVFPPTSFGPLVRKMFV 4699
TR|A0A166ZL34|A0A166ZL34_9NIDO    TEERLCLFDRYFKYWDQTYHPNCINCLDDRCILHCANFNVLFSTVFPPTSFGPLVRKMFV 4506
TR|R9QTB2|R9QTB2_CVHSA            ------------------------------------------------------------
TR|R9QTH2|R9QTH2_CVHSA            ------------------------------------------------------------
SP|P0C6U8|R1A_CVHSA               ------------------------------------------------------------
TR|Q6JH47|Q6JH47_CVHSA            ------------------------------------------------------------
TR|Q692E5|Q692E5_CVHSA            ------------------------------------------------------------
SP|P0C6F8|R1A_BCHK3               ------------------------------------------------------------
TR|A0A0K1Z0N1|A0A0K1Z0N1_CVHSA    ------------------------------------------------------------
SP|P0C6F5|R1A_BC279               ------------------------------------------------------------
SP|P0C6T7|R1A_BCRP3               ------------------------------------------------------------
```

```
QHN73794                       DGVPFVVSTGYHFRELGVVHNQDVNLHSSRLSFKELLVYAADPAMHAASGNLLLDKRTTC 4787
SP|P0C6X7|R1AB_CVHSA           DGVPFVVSTGYHFRELGVVHNQDVNLHSSRLSFKELLVYAADPAMHAASGNLLLDKRTTC 4764
TR|Q6UZF5|Q6UZF5_CVHSA         DGVPFVVSTGYHFRELGVVHNQDVNLHSSRLSFKELLVYAADPAMHAASGNLLLDKRTTC 4764
TR|Q6UZF1|Q6UZF1_CVHSA         DGVPFVVSTGYHFRELGVVHNQDVNLHSSRLSFKELLVYAADPAMHAASGNLLLDKRTTC 4764
TR|Q6JH48|Q6JH48_CVHSA         DGVPFVVSTGYHFRELGVVHNQDVNLHSSRLSFKELLVYAADPAMHAASGNLLLDKRTTC 4764
TR|Q692E6|Q692E6_CVHSA         DGVPFVVSTGYHFRELGVVHNQDVNLHSSRLSFKELLVYAADPAMHAASGNLLLDKRTTC 4764
TR|A0A0K1YZY7|A0A0K1YZY7_CVHSA DGVPFVVSTGYHFRELGVVHNQDVNLHSSRLSFKELLVYAADPAMHAASGNLLLDKRTTC 4764
SP|P0C6W2|R1AB_BCHK3           DGVPFVVSTGYHFRELGVVHNQDVNLHSSRLSFKELLVYAADPAMHAASGNLLLDKRTTC 4758
SP|P0C6W6|R1AB_BCRP3           DGVPFVVSTGYHFRELGVVHNQDVNLHSSRLSFKELLVYAADPAMHAASGNLLLDKRTTC 4762
SP|P0C6V9|R1AB_BC279           DGVPFVVSTGYHFRELGVVHNQDVNLHSSRLSFKELLVYAADPAMHAASGNLLLDKRTTC 4770
TR|A0A0U1WHI4|A0A0U1WHI4_CVHSA DGVPFVVSTGYHFRELGVVHNQDVNLHSSRLSFKELLVYAADPAMHAASGNLLLDKRTTC 4759
TR|A0A0U1WHG0|A0A0U1WHG0_CVHSA DGVPFVVSTGYHFRELGVVHNQDVNLHSSRLSFKELLVYAADPAMHAASGNLLLDKRTTC 4759
TR|A0A166ZL34|A0A166ZL34_9NIDO DGVPFVVSTGYHFRELGVVHNQDVNLHSSRLSFKELLVYAADPAMHAASGNLLLDKRTTC 4566
TR|R9QTB2|R9QTB2_CVHSA         ------------------------------------------------------------
TR|R9QTH2|R9QTH2_CVHSA         ------------------------------------------------------------
SP|P0C6U8|R1A_CVHSA            ------------------------------------------------------------
TR|Q6JH47|Q6JH47_CVHSA         ------------------------------------------------------------
TR|Q692E5|Q692E5_CVHSA         ------------------------------------------------------------
SP|P0C6F8|R1A_BCHK3            ------------------------------------------------------------
TR|A0A0K1Z0N1|A0A0K1Z0N1_CVHSA ------------------------------------------------------------
SP|P0C6F5|R1A_BC279            ------------------------------------------------------------
SP|P0C6T7|R1A_BCRP3            ------------------------------------------------------------

QHN73794                       FSVAALTNNVAFQTVKPGNFNKDFYDFAVSKGFFKEGSSVELKHFFFAQDGNAAISDYDY 4847
SP|P0C6X7|R1AB_CVHSA           FSVAALTNNVAFQTVKPGNFNKDFYDFAVSKGFFKEGSSVELKHFFFAQDGNAAISDYDY 4824
TR|Q6UZF5|Q6UZF5_CVHSA         FSVAALTNNVAFQTVKPGNFNKDFYDFAVSKGFFKEGSSVELKHFFFAQDGNAAISDYDY 4824
TR|Q6UZF1|Q6UZF1_CVHSA         FSVAALTNNVAFQTVKPGNFNKDFYDFAVSKGFFKEGSSVELKHFFFAQDGNAAISDYDY 4824
TR|Q6JH48|Q6JH48_CVHSA         FSVAALTNNVAFQTVKPGNFNKDFYDFAVSKGFFKEGSSVELKHFFFAQDGNAAISDYDY 4824
TR|Q692E6|Q692E6_CVHSA         FSVAALTNNVAFQTVKPGNFNKDFYDFAVSKGFFKEGSSVELKHFFFAQDGNAAISDYDY 4824
TR|A0A0K1YZY7|A0A0K1YZY7_CVHSA FSVAALTNNVAFQTVKPGNFNKDFYDFAVSKGFFKEGSSVELKHFFFAQDGNAAISDYDY 4824
SP|P0C6W2|R1AB_BCHK3           FSVAALTNNVAFQTVKPGNFNKDFYDFAVSKGFFKEGSSVELKHFFFAQDGNAAISDYDY 4818
SP|P0C6W6|R1AB_BCRP3           FSVAALTNNVAFQTVKPGNFNKDFYDFAVSKGFFKEGSSVELKHFFFAQDGNAAISDYDY 4822
SP|P0C6V9|R1AB_BC279           FSVAALTNNVAFQTVKPGNFNKDFYDFAVSKGFFKEGSSVELKHFFFAQDGNAAISDYDY 4830
TR|A0A0U1WHI4|A0A0U1WHI4_CVHSA FSVAALTNNVSFQTVKPGNFNKDFYDFAVSKGFFKEGSSVELKHFFFAQDGNAAISDYDY 4819
TR|A0A0U1WHG0|A0A0U1WHG0_CVHSA FSVAALTNNVSFQTVKPGNFNKDFYDFAVSKGFFKEGSSVELKHFFFAQDGNAAISDYDY 4819
TR|A0A166ZL34|A0A166ZL34_9NIDO FSVAALTNNVSFQTVKPGNFNKDFYDFAVSKGFFKEGSSVELKHFFFAQDGNAAISDYDY 4626
TR|R9QTB2|R9QTB2_CVHSA         ------------------------------------------------------------
TR|R9QTH2|R9QTH2_CVHSA         ------------------------------------------------------------
SP|P0C6U8|R1A_CVHSA            ------------------------------------------------------------
TR|Q6JH47|Q6JH47_CVHSA         ------------------------------------------------------------
TR|Q692E5|Q692E5_CVHSA         ------------------------------------------------------------
SP|P0C6F8|R1A_BCHK3            ------------------------------------------------------------
TR|A0A0K1Z0N1|A0A0K1Z0N1_CVHSA ------------------------------------------------------------
SP|P0C6F5|R1A_BC279            ------------------------------------------------------------
SP|P0C6T7|R1A_BCRP3            ------------------------------------------------------------

QHN73794                       YRYNLPTMCDIRQLLFVVEVVDKYFDCYDGGCINANQVIVNNLDKSAGFPFNKWGKARLY 4907
SP|P0C6X7|R1AB_CVHSA           YRYNLPTMCDIRQLLFVVEVVDKYFDCYDGGCINANQVIVNNLDKSAGFPFNKWGKARLY 4884
TR|Q6UZF5|Q6UZF5_CVHSA         YRYNLPTMCDIRQLLFVVEVVDKYFDCYDGGCINANQVIVNNLDKSAGFPFNKWGKARLY 4884
TR|Q6UZF1|Q6UZF1_CVHSA         YRYNLPTMCDIRQLLFVVEVVDKYFDCYDGGCINANQVIVNNLDKSAGFPFNKWGKARLY 4884
TR|Q6JH48|Q6JH48_CVHSA         YRYNLPTMCDIRQLLFVVEVVDKYFDCYDGGCINANQVIVNNLDKSAGFPFNKWGKARLY 4884
TR|Q692E6|Q692E6_CVHSA         YRYNLPTMCDIRQLLFVVEVVDKYFDCYDGGCINANQVIVNNLDKSAGFPFNKWGKARLY 4884
TR|A0A0K1YZY7|A0A0K1YZY7_CVHSA YRYNLPTMCDIRQLLFVVEVVDKYFDCYDGGCINANQVIVNNLDKSAGFPFNKWGKARLY 4884
SP|P0C6W2|R1AB_BCHK3           YRYNLPTMCDIRQLLFVVEVVDKYFDCYDGGCINANQVIVNNLDKSAGFPFNKWGKARLY 4878
SP|P0C6W6|R1AB_BCRP3           YRYNLPTMCDIRQLLFVVEVVDKYFDCYDGGCINANQVIVNNLDKSAGFPFNKWGKARLY 4882
SP|P0C6V9|R1AB_BC279           YRYNLPTMCDIRQLLFVVEVVDKYFDCYDGGCINANQVIVNNLDKSAGFPFNKWGKARLY 4890
TR|A0A0U1WHI4|A0A0U1WHI4_CVHSA YRYNLPTMCDIRQLLFVVEVVDKYFDCYDGGCINANQVIVNNLDKSAGFPFNKWGKARLY 4879
TR|A0A0U1WHG0|A0A0U1WHG0_CVHSA YRYNLPTMCDIRQLLFVVEVVDKYFDCYDGGCINANQVIVNNLDKSAGFPFNKWGKARLY 4879
TR|A0A166ZL34|A0A166ZL34_9NIDO YRYNLPTMCDIRQLLFVVEVVDKYFDCYDGGCINANQVIVNNLDKSAGFPFNKWGKARLY 4686
TR|R9QTB2|R9QTB2_CVHSA         ------------------------------------------------------------
TR|R9QTH2|R9QTH2_CVHSA         ------------------------------------------------------------
SP|P0C6U8|R1A_CVHSA            ------------------------------------------------------------
TR|Q6JH47|Q6JH47_CVHSA         ------------------------------------------------------------
TR|Q692E5|Q692E5_CVHSA         ------------------------------------------------------------
SP|P0C6F8|R1A_BCHK3            ------------------------------------------------------------
TR|A0A0K1Z0N1|A0A0K1Z0N1_CVHSA ------------------------------------------------------------
SP|P0C6F5|R1A_BC279            ------------------------------------------------------------
SP|P0C6T7|R1A_BCRP3            ------------------------------------------------------------
```

```
QHN73794                           YDSMSYEDQDALFAYTKRNVIPTITQMNLKYAISAKNRARTVAGVSICSTMTNRQFHQKL 4967
SP|P0C6X7|R1AB_CVHSA               YDSMSYEDQDALFAYTKRNVIPTITQMNLKYAISAKNRARTVAGVSICSTMTNRQFHQKL 4944
TR|Q6UZF5|Q6UZF5_CVHSA             YDSMSYEDQDALFAYTKRNVIPTITQMNLKYAISAKNRARTVAGVSICSTMTNRQFHQKL 4944
TR|Q6UZF1|Q6UZF1_CVHSA             YDSMSYEDQDALFAYTKRNVIPTITQMNLKYAISAKNRARTVAGVSICSTMTNRQFHQKL 4944
TR|Q6JH48|Q6JH48_CVHSA             YDSMSYEDQDALFAYTKRNVIPTITQMNLKYAISAKNRARTVAGVSICSTMTNRQFHQKL 4944
TR|Q692E6|Q692E6_CVHSA             YDSMSYEDQDALFAYTKRNVIPTITQMNLKYAISAKNRARTVAGVSICSTMTNRQFHQKL 4944
TR|A0A0K1YZY7|A0A0K1YZY7_CVHSA     YDSMSYEDQDALFAYTKRNVIPTITQMNLKYAISAKNRARTVAGVSICSTMTNRQFHQKL 4944
SP|P0C6W2|R1AB_BCHK3               YDSMSYEDQDALFAYTKRNVIPTITQMNLKYAISAKNRARTVAGVSICSTMTNRQFHQKL 4938
SP|P0C6W6|R1AB_BCRP3               YDSMSYEDQDALFAYTKRNVIPTITQMNLKYAISAKNRARTVAGVSICSTMTNRQFHQKL 4942
SP|P0C6V9|R1AB_BC279               YDSMSYEDQDVLFAYTKRNVIPTITQMNLKYAISAKNRARTVAGVSICSTMTNRQFHQKL 4950
TR|A0A0U1WHI4|A0A0U1WHI4_CVHSA     YDSMSYEDQDALFAYTKRNVLPTITQMNLKYAISAKNRARTVAGVSICSTMTNRQFHQKL 4939
TR|A0A0U1WHG0|A0A0U1WHG0_CVHSA     YDSMSYEDQDALFAYTKRNVLPTITQMNLKYAISAKNRARTVAGVSICSTMTNRQFHQKL 4939
TR|A0A166ZL34|A0A166ZL34_9NIDO     YDSMSYEDQDALFAYTKRNVLPTITQMNLKYAISAKNRARTVAGVSICSTMTNRQFHQKL 4746
TR|R9QTB2|R9QTB2_CVHSA             ------------------------------------------------------------
TR|R9QTH2|R9QTH2_CVHSA             ------------------------------------------------------------
SP|P0C6U8|R1A_CVHSA                ------------------------------------------------------------
TR|Q6JH47|Q6JH47_CVHSA             ------------------------------------------------------------
TR|Q692E5|Q692E5_CVHSA             ------------------------------------------------------------
SP|P0C6F8|R1A_BCHK3                ------------------------------------------------------------
TR|A0A0K1Z0N1|A0A0K1Z0N1_CVHSA     ------------------------------------------------------------
SP|P0C6F5|R1A_BC279                ------------------------------------------------------------
SP|P0C6T7|R1A_BCRP3                ------------------------------------------------------------

QHN73794                           LKSIAATRGATVVIGTSKFYGGWHNMLKTVYSDVENPHLMGWDYPKCDRAMPNMLRIMAS 5027
SP|P0C6X7|R1AB_CVHSA               LKSIAATRGATVVIGTSKFYGGWHNMLKTVYSDVETPHLMGWDYPKCDRAMPNMLRIMAS 5004
TR|Q6UZF5|Q6UZF5_CVHSA             LKSIAATRGATVVIGTSKFYGGWHNMLKTVYSDVETPHLMGWDYPKCDRAMPNMLRIMAS 5004
TR|Q6UZF1|Q6UZF1_CVHSA             LKSIAATRGATVVIGTSKFYGGWHNMLKTVYSDVETPHLMGWDYPKCDRAMPNMLRIMAS 5004
TR|Q6JH48|Q6JH48_CVHSA             LKSIAATRGATVVIGTSKFYGGWHNMLKTVYSDVETPHLMGWDYPKCDRAMPNMLRIMAS 5004
TR|Q692E6|Q692E6_CVHSA             LKSIAATRGATVVIGTSKFYGGWHNMLKTVYSDVETPHLMGWDYPKCDRAMPNMLRIMAS 5004
TR|A0A0K1YZY7|A0A0K1YZY7_CVHSA     LKSIAATRGATVVIGTSKFYGGWHNMLKTVYSDVETPHLMGWDYPKCDRAMPNMLRIMAS 5004
SP|P0C6W2|R1AB_BCHK3               LKSIAATRGATVVIGTSKFYGGWHNMLKTVYSDVESPHLMGWDYPKCDRAMPNMLRIMAS 4998
SP|P0C6W6|R1AB_BCRP3               LKSIAATRGATVVIGTSKFYGGWHNMLKTVYSDVETPHLMGWDYPKCDRAMPNMLRIMAS 5002
SP|P0C6V9|R1AB_BC279               LKSIAATRGATVVIGTSKFYGGWHNMLKTVYSDVETPHLMGWDYPKCDRAMPNMLRIMAS 5010
TR|A0A0U1WHI4|A0A0U1WHI4_CVHSA     LKSIAATRGATVVIGTSKFYGGWHNMLKTVYSDVETPYLMGWDYPKCDRAMPNMLRIMAF 4999
TR|A0A0U1WHG0|A0A0U1WHG0_CVHSA     LKSIAATRGATVVIGTSKFYGGWHNMLKTVYSDVETPHLMGWDYPKCDRAMPNMLRIMAS 4999
TR|A0A166ZL34|A0A166ZL34_9NIDO     LKSIAATRGATVVIGTSKFYGGWHNMLKTVYSDVETPHLMGWDYPKCDRAMPNMLRIMAS 4806
TR|R9QTB2|R9QTB2_CVHSA             ------------------------------------------------------------
TR|R9QTH2|R9QTH2_CVHSA             ------------------------------------------------------------
SP|P0C6U8|R1A_CVHSA                ------------------------------------------------------------
TR|Q6JH47|Q6JH47_CVHSA             ------------------------------------------------------------
TR|Q692E5|Q692E5_CVHSA             ------------------------------------------------------------
SP|P0C6F8|R1A_BCHK3                ------------------------------------------------------------
TR|A0A0K1Z0N1|A0A0K1Z0N1_CVHSA     ------------------------------------------------------------
SP|P0C6F5|R1A_BC279                ------------------------------------------------------------
SP|P0C6T7|R1A_BCRP3                ------------------------------------------------------------

QHN73794                           LVLARKHTTCCSLSHRFYRLANECAQVLSEMVMCGGSLYVKPGGTSSGDATTAYANSVFN 5087
SP|P0C6X7|R1AB_CVHSA               LVLARKHNTCCNLSHRFYRLANECAQVLSEMVMCGGSLYVKPGGTSSGDATTAYANSVFN 5064
TR|Q6UZF5|Q6UZF5_CVHSA             LVLARKHNTCCNLSHRFYRLANECAQVLSEMVMCGGSLYVKPGGTSSGDATTAYANSVFN 5064
TR|Q6UZF1|Q6UZF1_CVHSA             LVLARKHNTCCNLSHRFYRLANECAQVLSEMVMCGGSLYVKPGGTSSGDATTAYANSVFN 5064
TR|Q6JH48|Q6JH48_CVHSA             LVLARKHNTCCNLSHRFYRLANECAQVLSEMVMCGGSLYVKPGGTSSGDATTAYANSVFN 5064
TR|Q692E6|Q692E6_CVHSA             LVLARKHNTCCNLSHRFYRLANECAQVLSEMVMCGGSLYVKPGGTSSGDATTAYANSVFN 5064
TR|A0A0K1YZY7|A0A0K1YZY7_CVHSA     LVLARKHSTCCNLSHRFYRLANECAQVLSEMVMCGGSLYVKPGGTSSGDATTAYANSVFN 5064
SP|P0C6W2|R1AB_BCHK3               LILARKHSTCCNLSHRFYRLANECAQVLSEMVMCGGSLYVKPGGTSSGDATTAYANSVFN 5058
SP|P0C6W6|R1AB_BCRP3               LVLARKHSTCCNLSHRFYRLANECAQVLSEMVMCGGSLYVKPGGTSSGDATTAYANSVFN 5062
SP|P0C6V9|R1AB_BC279               LVLARKHSTCCNLSHRFYRLANECAQVLSEMVMCGGSLYVKPGGTSSGDATTAYANSVFN 5070
TR|A0A0U1WHI4|A0A0U1WHI4_CVHSA     LVFSRKHSTCCNLSHRFYRLANECAQVLSEMVMCGGSLYVKPGGTSSGDATTAYANSVFN 5059
TR|A0A0U1WHG0|A0A0U1WHG0_CVHSA     LVLARKHSTCCNLSHRFYRLANECAQVLSEMVMCGGSLYVKPGGTSSGDATTAYANSVFN 5059
TR|A0A166ZL34|A0A166ZL34_9NIDO     LVLARKHSTCCNLSHRFYRLANECAQVLSEMVMCGGSLYVKPGGTSSGDATTAYANSVFN 4866
TR|R9QTB2|R9QTB2_CVHSA             ------------------------------------------------------------
TR|R9QTH2|R9QTH2_CVHSA             ------------------------------------------------------------
SP|P0C6U8|R1A_CVHSA                ------------------------------------------------------------
TR|Q6JH47|Q6JH47_CVHSA             ------------------------------------------------------------
TR|Q692E5|Q692E5_CVHSA             ------------------------------------------------------------
SP|P0C6F8|R1A_BCHK3                ------------------------------------------------------------
TR|A0A0K1Z0N1|A0A0K1Z0N1_CVHSA     ------------------------------------------------------------
SP|P0C6F5|R1A_BC279                ------------------------------------------------------------
SP|P0C6T7|R1A_BCRP3                ------------------------------------------------------------
```

```
QHN73794                          ICQAVTANVNALLSTDGNKIADKYVRNLQHRLYECLYRNRDVDTDFVNEFYAYLRKHFSM 5147
SP|P0C6X7|R1AB_CVHSA              ICQAVTANVNALLSTDGNKIADKYVRNLQHRLYECLYRNRDVDHEFVDEFYAYLRKHFSM 5124
TR|Q6UZF5|Q6UZF5_CVHSA            ICQAVTANVNALLSTDGNKIADKYVRNLQHRLYECLYRNRDVDHEFVDEFYAYLRKHFSM 5124
TR|Q6UZF1|Q6UZF1_CVHSA            ICQAVTANVNALLSTDGNKIADKYVRNLQHRLYECLYRNRDVDHEFVDEFYAYLRKHFSM 5124
TR|Q6JH48|Q6JH48_CVHSA            ICQAVTANVNALLSTDGNKIADKYVRNLQHRLYECLYRNRDVDHEFVDEFYAYLRKHFSM 5124
TR|Q692E6|Q692E6_CVHSA            ICQAVTANVNALLSTDGNKIADKYVRNLQHRLYECLYRNRDVDHEFVDEFYAYLRKHFSM 5124
TR|A0A0K1YZY7|A0A0K1YZY7_CVHSA    ICQAVTANVNALLSTDGNKIADKYVRNLQHRLYECLYRNRDVDHEFVDEFYAYLRKHFSM 5124
SP|P0C6W2|R1AB_BCHK3              ICQAVTANVNALLSTDGNKIADKYVRNLQHRLYECLYRNRDVDHEFVDEFYAYLRKHFSM 5118
SP|P0C6W6|R1AB_BCRP3              ICQAVTANVNALLSTDGNKIADKYVRNLQHRLYECLYRNRDVDHEFVGEFYAYLRKHFSM 5122
SP|P0C6V9|R1AB_BC279              ICQAVTANVNALLSTDGNKIADKYVRNLQHRLYECLYRNRDVDHEFVDEFYAYLRKHFSM 5130
TR|A0A0U1WHI4|A0A0U1WHI4_CVHSA    ICQAVTANVNALLSTDGNKIADKYVRNLQHRLYECLYRNRDVDHEFVDEFYAYLRKHFSM 5119
TR|A0A0U1WHG0|A0A0U1WHG0_CVHSA    ICQAVTANVNALLSTDGNKIADKYVRNIQHRLYECLYRNRDVDHEFVDEFYAYLRKHFSM 5119
TR|A0A166ZL34|A0A166ZL34_9NIDO    ICQAVTANVNALLSTDGNKIADKYVRNIQHRLYECLYRNRDVDHEFVDEFYAYLRKHFSM 4926
TR|R9QTB2|R9QTB2_CVHSA            ------------------------------------------------------------
TR|R9QTH2|R9QTH2_CVHSA            ------------------------------------------------------------
SP|P0C6U8|R1A_CVHSA               ------------------------------------------------------------
TR|Q6JH47|Q6JH47_CVHSA            ------------------------------------------------------------
TR|Q692E5|Q692E5_CVHSA            ------------------------------------------------------------
SP|P0C6F8|R1A_BCHK3               ------------------------------------------------------------
TR|A0A0K1Z0N1|A0A0K1Z0N1_CVHSA    ------------------------------------------------------------
SP|P0C6F5|R1A_BC279               ------------------------------------------------------------
SP|P0C6T7|R1A_BCRP3               ------------------------------------------------------------

QHN73794                          MILSDDAVVCFNSTYASQGLVASIKNFKSVLYYQNNVFMSEAKCWTETDLTKGPHEFCSQ 5207
SP|P0C6X7|R1AB_CVHSA              MILSDDAVVCYNSNYAAQGLVASIKNFKAVLYYQNNVFMSEAKCWTETDLTKGPHEFCSQ 5184
TR|Q6UZF5|Q6UZF5_CVHSA            MILSDDAVVCYNSNYAAQGLVASIKNFKAVLYYQNNVFMSEAKCWTETDLTKGPHEFCSQ 5184
TR|Q6UZF1|Q6UZF1_CVHSA            MILSDDAVVCYNSNYAAQGLVASIKNFKAVLYYQNNVFMSEAKCWTETDLTKGPHEFCSQ 5184
TR|Q6JH48|Q6JH48_CVHSA            MILSDDAVVCYNSNYAAQGLVASIKNFKAVLYYQNNVFMSEAKCWTETDLTKGPHEFCSQ 5184
TR|Q692E6|Q692E6_CVHSA            MILSDDAVVCYNSNYAAQGLVASIKNFKAVLYYQNNVFMSEAKCWTETDLTKGPHEFCSQ 5184
TR|A0A0K1YZY7|A0A0K1YZY7_CVHSA    MILSDDAVVCYNSNYAAQGLVASIKNFKAVLYYQNNVFMSEAKCWTETDLTKGPHEFCSQ 5184
SP|P0C6W2|R1AB_BCHK3              MILSDDAVVCYNSNYAAQGLVASIKNFKAVLYYQNNVFMSEAKCWTETDLTRGPHEFCSQ 5178
SP|P0C6W6|R1AB_BCRP3              MILSDDAVVCYNSNYAAQGLVASIKNFKAVLYYQNNVFMSEAKCWTETDLTKGPHEFCSQ 5182
SP|P0C6V9|R1AB_BC279              MILSDDAVVCYNSNYAAQGLVASIKNFKAVHYYQNNVFMSEAKCWTETDLTKGPHEFCSQ 5190
TR|A0A0U1WHI4|A0A0U1WHI4_CVHSA    MILSDDAVVCYNSNYAAQGLVASIKNFKAVLYYQNNVFMSEAKCWTETDLTKGPHEFCSQ 5179
TR|A0A0U1WHG0|A0A0U1WHG0_CVHSA    MILSDDAVVCYNSNYAAQGLVASIKNFKAVLYYQNNVFMSEAKCWTETDLTKGPHEFCSQ 5179
TR|A0A166ZL34|A0A166ZL34_9NIDO    MILSDDAVVCYNSNYAAQGLVASIKNFKAVLYYQNNVFMSEAKCWTETDLTKGPHEFCSQ 4986
TR|R9QTB2|R9QTB2_CVHSA            ------------------------------------------------------------
TR|R9QTH2|R9QTH2_CVHSA            ------------------------------------------------------------
SP|P0C6U8|R1A_CVHSA               ------------------------------------------------------------
TR|Q6JH47|Q6JH47_CVHSA            ------------------------------------------------------------
TR|Q692E5|Q692E5_CVHSA            ------------------------------------------------------------
SP|P0C6F8|R1A_BCHK3               ------------------------------------------------------------
TR|A0A0K1Z0N1|A0A0K1Z0N1_CVHSA    ------------------------------------------------------------
SP|P0C6F5|R1A_BC279               ------------------------------------------------------------
SP|P0C6T7|R1A_BCRP3               ------------------------------------------------------------

                                                             _
QHN73794                          HTMLVKQGDDYVYLPYPDPSRILGAGCFVDDIVKTDGTLMIERFVSLAIDAYPLTKHPNQ 5267
SP|P0C6X7|R1AB_CVHSA              HTMLVKQGDDYVYLPYPDPSRILGAGCFVDDIVKTDGTLMIERFVSLAIDAYPLTKHPNQ 5244
TR|Q6UZF5|Q6UZF5_CVHSA            HTMLVKQGDDYVYLPYPDPSRILGAGCFVDDIVKTDGTLMIERFVSLAIDAYPLTKHPNQ 5244
TR|Q6UZF1|Q6UZF1_CVHSA            HTMLVKQGDDYVYLPYPDPSRILGAGCFVDDIVKTDGTLMIERFVSLAIDAYPLTKHPNQ 5244
TR|Q6JH48|Q6JH48_CVHSA            HTMLVKQGDDYVYLPYPDPSRILGAGCFVDDIVKTDGTLMIERFVSLAIDAYPLTKHPNQ 5244
TR|Q692E6|Q692E6_CVHSA            HTMLVKQGDDYVYLPYPDPSRILGAGCFVDDIVKTDGTLMIERFVSLAIDAYPLTKHPNQ 5244
TR|A0A0K1YZY7|A0A0K1YZY7_CVHSA    HTMLVKQGDDYVYLPYPDPSRILGAGCFVDDIVKTDGTLMIERFVSLAIDAYPLTKHPNQ 5244
SP|P0C6W2|R1AB_BCHK3              HTMLVKQGDDYVYLPYPDPSRILGAGCFVDDIVKTDGTLMIERFVSLAIDAYPLTKHPNQ 5238
SP|P0C6W6|R1AB_BCRP3              HTMLVKQGDDYVYLPYPDPSRILGAGCFVDDIVKTDGTLMIERFVSLAIDAYPLTKHPNQ 5242
SP|P0C6V9|R1AB_BC279              HTMLVKQGDDYVYLPYPDPSRILGAGCFVDDIVKTDGTLMIERFVSLAIDAYPLTKHPNQ 5250
TR|A0A0U1WHI4|A0A0U1WHI4_CVHSA    HTMLVKQGDDYVYLPYPDPSRILGAGCFVDDIVKTDGTLMIERFVSLAIDAYPLTKHPNQ 5239
TR|A0A0U1WHG0|A0A0U1WHG0_CVHSA    HTMLVKQGDDYVYLPYPDPSRILGAGCFVDDIVKTDGTLMIERFVSLAIDAYPLTKHPNQ 5239
TR|A0A166ZL34|A0A166ZL34_9NIDO    HTMLVKQGDDYVYLPYPDPSRILGAGCFVDDIVKTDGTLMIERFVSLAIDAYPLTKHPNQ 5046
TR|R9QTB2|R9QTB2_CVHSA            ------------------------------------------------------------
TR|R9QTH2|R9QTH2_CVHSA            ------------------------------------------------------------
SP|P0C6U8|R1A_CVHSA               ------------------------------------------------------------
TR|Q6JH47|Q6JH47_CVHSA            ------------------------------------------------------------
TR|Q692E5|Q692E5_CVHSA            ------------------------------------------------------------
SP|P0C6F8|R1A_BCHK3               ------------------------------------------------------------
TR|A0A0K1Z0N1|A0A0K1Z0N1_CVHSA    ------------------------------------------------------------
SP|P0C6F5|R1A_BC279               ------------------------------------------------------------
SP|P0C6T7|R1A_BCRP3               ------------------------------------------------------------
```

```
QHN73794                         EYADVFHLYLQYIRKLHDELTGHMLDMYSVMLTNDNTSRYWEPEFYEAMYTPHTVLQAVG 5327
SP|P0C6X7|R1AB_CVHSA             EYADVFHLYLQYIRKLHDELTGHMLDMYSVMLTNDNTSRYWEPEFYEAMYTPHTVLQAVG 5304
TR|Q6UZF5|Q6UZF5_CVHSA           EYADVFHLYLQYIRKLHDELTGHMLDMYSVMLTNDNTSRYWEPEFYEAMYTPHTVLQAVG 5304
TR|Q6UZF1|Q6UZF1_CVHSA           EYADVFHLYLQYIRKLHDELTGHMLDMYSVMLTNDNTSRYWEPEFYEAMYTPHTVLQAVG 5304
TR|Q6JH48|Q6JH48_CVHSA           EYAAVFHLYLQYIRKLHDELTGHMLDMYSVMLTNDNTSRYWEPEFYEAMYTPHTVLQAVG 5304
TR|Q692E6|Q692E6_CVHSA           EYADVFHLYLQYIRKLHDELTGHMLDMYSVMLTNDNTSRYWEPEFYEAMYTPHTVLQAVG 5304
TR|A0A0K1YZY7|A0A0K1YZY7_CVHSA EYADVFHLYLQYIRKLHDELTGHMLDMYSVMLTNDNTSRYWEPEFYEAMYTPHTVLQAVG 5304
SP|P0C6W2|R1AB_BCHK3             EYADVFHLYLQYIRKLHDELTGHMLDMYSVMLTNDNTSRYWEPEFYEAMYTPHTVLQAVG 5298
SP|P0C6W6|R1AB_BCRP3             EYADVFHLYLQYIRKLHDELTGHMLDMYSVMLTNDNTSRYWEPEFYEAMYTPHTVLQAVG 5302
SP|P0C6V9|R1AB_BC279             EYADVFHLYLQYIRKLHDELTGHMLDMYSVMLTNDNTSRYWEPEFYEAMYTPHTVLQAVG 5310
TR|A0A0U1WHI4|A0A0U1WHI4_CVHSA EYADVFHLYLQYIRKLHDELTGHMLDMYSVMLTNDNTSRYWEPEFYEAMYTPHTILQAVG 5299
TR|A0A0U1WHG0|A0A0U1WHG0_CVHSA EYADVFHLYLQYIRKLHDELTGHMLDMYSVMLTNDNTSRYWEPEFYEAMYTPHTILQAVG 5299
TR|A0A166ZL34|A0A166ZL34_9NIDO EYADVFHLYLQYIRKLHDELTGHMLDMYSVMLTNDNTSRYWEPEFYEAMYTPHTILQAVG 5106
TR|R9QTB2|R9QTB2_CVHSA           ------------------------------------------------------------
TR|R9QTH2|R9QTH2_CVHSA           ------------------------------------------------------------
SP|P0C6U8|R1A_CVHSA              ------------------------------------------------------------
TR|Q6JH47|Q6JH47_CVHSA           ------------------------------------------------------------
TR|Q692E5|Q692E5_CVHSA           ------------------------------------------------------------
SP|P0C6F8|R1A_BCHK3              ------------------------------------------------------------
TR|A0A0K1Z0N1|A0A0K1Z0N1_CVHSA ------------------------------------------------------------
SP|P0C6F5|R1A_BC279              ------------------------------------------------------------
SP|P0C6T7|R1A_BCRP3              ------------------------------------------------------------

QHN73794                         ACVLCNSQTSLRCGACIRRPFLCCKCCYDHVISTSHKLVLSVNPYVCNAPGCDVTDVTQL 5387
SP|P0C6X7|R1AB_CVHSA             ACVLCNSQTSLRCGACIRRPFLCCKCCYDHVISTSHKLVLSVNPYVCNAPGCDVTDVTQL 5364
TR|Q6UZF5|Q6UZF5_CVHSA           ACVLCNSQTSLRCGACIRRPFLCCKCCYDHVISTSHKLVLSVNPYVCNAPGCDVTDVTQL 5364
TR|Q6UZF1|Q6UZF1_CVHSA           ACVLCNSQTSLRCGACIRRPFLCCKCCYDHVISTSHKLVLSVNPYVCNAPGCDVTDVTQL 5364
TR|Q6JH48|Q6JH48_CVHSA           ACVLCNSQTSLRCGACIRRPFLCCKCCYDHVISTSHKLVLSVNPYVCNAPGCDVTDVTQL 5364
TR|Q692E6|Q692E6_CVHSA           ACVLCNSQTSLRCGACIRRPFLCCKCCYDHVISTSHKLVLSVNPYVCNAPGCDVTDVTQL 5364
TR|A0A0K1YZY7|A0A0K1YZY7_CVHSA ACVLCNSQTSLRCGACIRRPFLCCKCCYDHVISTSHKLVLSVNPYVCNAPGCDVTDVTQL 5364
SP|P0C6W2|R1AB_BCHK3             ACVLCNSQTSLRCGACIRRPFLCCKCCYDHVISTSHKLVLSVNPYVCNAPGCDVTDVTQL 5358
SP|P0C6W6|R1AB_BCRP3             ACVLCNSQTSLRCGACIRRPFLCCKCCYDHVISTSHKLVLSVNPYVCNAPGCDVTDVTQL 5362
SP|P0C6V9|R1AB_BC279             ACVLCNSQTSLRCGACIRRPFLCCKCCYDHVISTSHKLVLSVNPYVCNAPGCDVTDVTQL 5370
TR|A0A0U1WHI4|A0A0U1WHI4_CVHSA ACVLCNSQTSLRCGACIRRPFLCCKCCYDHVISTSHKLVLSVNPYVCNAPGCDVTDVTQL 5359
TR|A0A0U1WHG0|A0A0U1WHG0_CVHSA ACVLCNSQTSLRCGACIRRPFLCCKCCYDHVISTSHKLVLSVNPYVCNAPGCDVTDVTQL 5359
TR|A0A166ZL34|A0A166ZL34_9NIDO ACVLCNSQTSLRCGACIRRPFLCCKCCYDHVISTSHKLVLSVNPYVCNAPGCDVTDVTQL 5166
TR|R9QTB2|R9QTB2_CVHSA           ------------------------------------------------------------
TR|R9QTH2|R9QTH2_CVHSA           ------------------------------------------------------------
SP|P0C6U8|R1A_CVHSA              ------------------------------------------------------------
TR|Q6JH47|Q6JH47_CVHSA           ------------------------------------------------------------
TR|Q692E5|Q692E5_CVHSA           ------------------------------------------------------------
SP|P0C6F8|R1A_BCHK3              ------------------------------------------------------------
TR|A0A0K1Z0N1|A0A0K1Z0N1_CVHSA ------------------------------------------------------------
SP|P0C6F5|R1A_BC279              ------------------------------------------------------------
SP|P0C6T7|R1A_BCRP3              ------------------------------------------------------------

QHN73794                         YLGGMSYYCKSHKPPISFPLCANGQVFGLYKNTCVGSDNVTDFNAIATCDWTNAGDYILA 5447
SP|P0C6X7|R1AB_CVHSA             YLGGMSYYCKSHKPPISFPLCANGQVFGLYKNTCVGSDNVTDFNAIATCDWTNAGDYILA 5424
TR|Q6UZF5|Q6UZF5_CVHSA           YLGGMSYYCKSHKPPISFPLCANGQVFGLYKNTCVGSDNVTDFNAIATCDWTNAGDYILA 5424
TR|Q6UZF1|Q6UZF1_CVHSA           YLGGMSYYCKSHKPPISFPLCANGQVFGLYKNTCVGSDNVTDFNAIATCDWTNAGDYILA 5424
TR|Q6JH48|Q6JH48_CVHSA           YLGGMSYYCKSHKPPISFPLCANGQVFGLYKNTCVGSDNVTDFNAIATCDWTNAGDYILA 5424
TR|Q692E6|Q692E6_CVHSA           YLGGMSYYCKSHKPPISFPLCANGQVFGLYKNTCVGSDNVTDFNAIATCDWTNAGDYILA 5424
TR|A0A0K1YZY7|A0A0K1YZY7_CVHSA YLGGMSYYCKSHKPPISFPLCANGQVFGLYKNTCVGSDNVTDFNAIATCDWTNAGDYILA 5424
SP|P0C6W2|R1AB_BCHK3             YLGGMSYYCKSHKPPISFPLCANGQVFGLYKNTCVGSDNVTDFNAIATCDWTNAGDYILA 5418
SP|P0C6W6|R1AB_BCRP3             YLGGMSYYCKSHKPPISFPLCANGQVFGLYKNTCVGSDNVTDFNAIATCDWTNAGDYILA 5422
SP|P0C6V9|R1AB_BC279             YLGGMSYYCKLHKPPISFPLCANGQVFGLYKNTCVGSDNVTDFNAIATCDWTNAGDYILA 5430
TR|A0A0U1WHI4|A0A0U1WHI4_CVHSA YLGGMSYYCKSHKPPISFPLCANGQVFGLYKNTCVGSDNVTDFNAIATCDWTNAGDYILA 5419
TR|A0A0U1WHG0|A0A0U1WHG0_CVHSA YLGGMSYYCKSHKPSISFPLCANGQVFGLYKNTCVGSDNVTDFNAIATCDWTNAGDYILA 5419
TR|A0A166ZL34|A0A166ZL34_9NIDO YLGGMSYYCKSHKPSISFPLCANGQVFGLYKNTCVGSDNVTDFNAIATCDWTNAGDYILA 5226
TR|R9QTB2|R9QTB2_CVHSA           ------------------------------------------------------------
TR|R9QTH2|R9QTH2_CVHSA           ------------------------------------------------------------
SP|P0C6U8|R1A_CVHSA              ------------------------------------------------------------
TR|Q6JH47|Q6JH47_CVHSA           ------------------------------------------------------------
TR|Q692E5|Q692E5_CVHSA           ------------------------------------------------------------
SP|P0C6F8|R1A_BCHK3              ------------------------------------------------------------
TR|A0A0K1Z0N1|A0A0K1Z0N1_CVHSA ------------------------------------------------------------
SP|P0C6F5|R1A_BC279              ------------------------------------------------------------
SP|P0C6T7|R1A_BCRP3              ------------------------------------------------------------
```

```
QHN73794                            NTCTERLKLFAAETLKATEETFKLSYGIATVREVLSDRELHLSWEVGKPRPPLNRNYVFT 5507
SP|P0C6X7|R1AB_CVHSA                NTCTERLKLFAAETLKATEETFKLSYGIATVREVLSDRELHLSWEVGKPRPPLNRNYVFT 5484
TR|Q6UZF5|Q6UZF5_CVHSA              NTCTERLKLFAAETLKATEETFKLSYGIATVREVLSDRELHLSWEVGKPRPPLNRNYVFT 5484
TR|Q6UZF1|Q6UZF1_CVHSA              NTCTERLKLFAAETLKATEETFKLSYGIATVREVLSDRELHLSWEVGKPRPPLNRNYVFT 5484
TR|Q6JH48|Q6JH48_CVHSA              NTCTERLKLFAAETLKATEETFKLSYGIATVREVLSDRELHLSWEVGKPRPPLNRNYVFT 5484
TR|Q692E6|Q692E6_CVHSA              NTCTERLKLFAAETLKATEETFKLSYGIATVREVLSDRELHLSWEVGKPRPPLNRNYVFT 5484
TR|A0A0K1YZY7|A0A0K1YZY7_CVHSA NTCTERLKLFAAETLKATEETFKLSYGIATVREVLSDRELHLSWEVGKPRPPLNRNYVFT 5484
SP|P0C6W2|R1AB_BCHK3                NTCTERLKLFAAETLKATEETFKLSYGIATVREVLSDRELYLSWEVGKPRPPLNRNYVFT 5478
SP|P0C6W6|R1AB_BCRP3                NTCTERLKLFAAETLKATEETFKLSYGIATVREVLSDRELHLSWEVGKPRPPLNRNYVFT 5482
SP|P0C6V9|R1AB_BC279                NTCTERLKLFAAETLKATEETFKLSYGIATVREVLSDRELHLSWEVGKPRPPLNRNYVFT 5490
TR|A0A0U1WHI4|A0A0U1WHI4_CVHSA NTCTERLKLFAAETLKATEETFKLSYGIATVREVLSDKELHLSWEVGKPRPPLNRNYVFT 5479
TR|A0A0U1WHG0|A0A0U1WHG0_CVHSA NTCTERLKLFAAETLKATEETFKLSYGIATVREVLSDKELYLSWEVGKPRPPLNRNYVFT 5479
TR|A0A166ZL34|A0A166ZL34_9NIDO NTCTERLKLFAAETLKATEETFKLSYGIATVREVLSDKELYLSWEVGKPRPPLNRNYVFT 5286
TR|R9QTB2|R9QTB2_CVHSA              ------------------------------------------------------------
TR|R9QTH2|R9QTH2_CVHSA              ------------------------------------------------------------
SP|P0C6U8|R1A_CVHSA                 ------------------------------------------------------------
TR|Q6JH47|Q6JH47_CVHSA              ------------------------------------------------------------
TR|Q692E5|Q692E5_CVHSA              ------------------------------------------------------------
SP|P0C6F8|R1A_BCHK3                 ------------------------------------------------------------
TR|A0A0K1Z0N1|A0A0K1Z0N1_CVHSA ------------------------------------------------------------
SP|P0C6F5|R1A_BC279                 ------------------------------------------------------------
SP|P0C6T7|R1A_BCRP3                 ------------------------------------------------------------

QHN73794                            GYRVTKNSKVQIGEYTFEKGDYGDAVVYRGTTTYKLNVGDYFVLTSHTVMPLSAPTLVPQ 5567
SP|P0C6X7|R1AB_CVHSA                GYRVTKNSKVQIGEYTFEKGDYGDAVVYRGTTTYKLNVGDYFVLTSHTVMPLSAPTLVPQ 5544
TR|Q6UZF5|Q6UZF5_CVHSA              GYRVTKNSKVQIGEYTFEKGDYGDAVVYRGTTTYKLNVGDYFVLTSHTVMPLSAPTLVPQ 5544
TR|Q6UZF1|Q6UZF1_CVHSA              GYRVTKNSKVQIGEYTFEKGDYGDAVVYRGTTTYKLNVGDYFVLTSHTVMPLSAPTLVSQ 5544
TR|Q6JH48|Q6JH48_CVHSA              GYRVTKNSKVQIGEYTFEKGDYGDAVVYRGTTTYKLNVGDYFVLTSHTVMPLSAPTLVSQ 5544
TR|Q692E6|Q692E6_CVHSA              GYRVTKNSKVQIGEYTFEKGDYGDAVVYRGTTTYKLNVGDYFVLTSHTVMPLSAPTLVPQ 5544
TR|A0A0K1YZY7|A0A0K1YZY7_CVHSA GYRVTKNSKVQIGEYTFEKGDYGDAVVYRGTTTYKLNVGDYFVLTSHTVMPLSAPTLVPQ 5544
SP|P0C6W2|R1AB_BCHK3                GYRVTKNSKVQIGEYTFEKGDYGDAVVYRGTTTYKLNVGDYFVLTSHTVMPLSAPTLVPQ 5538
SP|P0C6W6|R1AB_BCRP3                GYRVTKNSKVQIGEYTFEKGDYGDAVVYRGTTTYKLNIGDYFVLTSHTVMPLSAPTLVPQ 5542
SP|P0C6V9|R1AB_BC279                GYRVTKNSKVQIGEYTFEKGDYGDAVVYRGTTTYKLNVGDYFVLTSHTVMPLSAPTLVPQ 5550
TR|A0A0U1WHI4|A0A0U1WHI4_CVHSA GYRVTKNSKVQIGEYTFEKGDYGDAVVYRGTTTYKLNVGDYFVLTSHTVMPLSAPTLVPQ 5539
TR|A0A0U1WHG0|A0A0U1WHG0_CVHSA GYRVTKNSKVQIGEYTFEKGDYGDAVVYRGTTTYKLNVGDYFVLTSHTVMPLSAPTLVPQ 5539
TR|A0A166ZL34|A0A166ZL34_9NIDO GYRVTKNSKVQIGEYTFEKGDYGDAVVYRGTTTYKLNVGDYFVLTSHTVMPLSAPTLVPQ 5346
TR|R9QTB2|R9QTB2_CVHSA              ------------------------------------------------------------
TR|R9QTH2|R9QTH2_CVHSA              ------------------------------------------------------------
SP|P0C6U8|R1A_CVHSA                 ------------------------------------------------------------
TR|Q6JH47|Q6JH47_CVHSA              ------------------------------------------------------------
TR|Q692E5|Q692E5_CVHSA              ------------------------------------------------------------
SP|P0C6F8|R1A_BCHK3                 ------------------------------------------------------------
TR|A0A0K1Z0N1|A0A0K1Z0N1_CVHSA ------------------------------------------------------------
SP|P0C6F5|R1A_BC279                 ------------------------------------------------------------
SP|P0C6T7|R1A_BCRP3                 ------------------------------------------------------------

QHN73794                            EHYVRITGLYPTLNISDEFSSNVANYQKVGMQKYSTLQGPPGTGKSHFAIGLALYYPSAR 5627
SP|P0C6X7|R1AB_CVHSA                EHYVRITGLYPTLNISDEFSSNVANYQKVGMQKYSTLQGPPGTGKSHFAIGLALYYPSAR 5604
TR|Q6UZF5|Q6UZF5_CVHSA              EHYVRITGLYPTLNISDEFSSNVANYQKVGMQKYSTLQGPPGTGKSHFAIGLALYYPSAR 5604
TR|Q6UZF1|Q6UZF1_CVHSA              EHYVRITGLYPTLNISDEFSSNVANYQKVGMQKYSTLQGPPGTGKSHFAIGLALYYPSAR 5604
TR|Q6JH48|Q6JH48_CVHSA              EHYVRITGLYPTLNISDEFSSNVANYQKVGMQKYSTLQGPPGTGKSHFAIGLALYYPSAR 5604
TR|Q692E6|Q692E6_CVHSA              EHYVRITGLYPTLNISDEFSSNVANYQKVGMQKYSTLQGPPGTGKSHFAIGLALYYPSAR 5604
TR|A0A0K1YZY7|A0A0K1YZY7_CVHSA EYYVRITGLYPTLNISDEFSSNVANYQKVGMQKYSTLQGPPGTGKSHFAIGLALYYPSAR 5604
SP|P0C6W2|R1AB_BCHK3                EHYVRITGLYPTLNISNEFSSNVANYQKIGMQKYSTLQGPPGTGKSHFAIGLALYYPSAR 5598
SP|P0C6W6|R1AB_BCRP3                EHYVRITGLYPTLNISDEFSSNVANYQKVGMQKYSTLQGPPGTGKSHFAIGLALYYPSAR 5602
SP|P0C6V9|R1AB_BC279                EHYVRITGLYPTLNISDEFSSNVANYQKVGMQKYSTLQGPPGTGKSHFAIGLALYYPSAR 5610
TR|A0A0U1WHI4|A0A0U1WHI4_CVHSA EHYVRITGLYPTLNISEEFSSNVANYQKVGMQKYSTLQGPPGTGKSHFAIGLALYYPSAR 5599
TR|A0A0U1WHG0|A0A0U1WHG0_CVHSA EHYVRITGLYPTLNISEEFSSNVANYQKVGMQKYSTLQGPPGTGKSHFAIGLALYYPSAR 5599
TR|A0A166ZL34|A0A166ZL34_9NIDO EHYVRITGLYPTLNISEEFSSNVANYQKVGMQKYSTLQGPPGTGKSHFAIGLALYYPSAR 5406
TR|R9QTB2|R9QTB2_CVHSA              ------------------------------------------------------------
TR|R9QTH2|R9QTH2_CVHSA              ------------------------------------------------------------
SP|P0C6U8|R1A_CVHSA                 ------------------------------------------------------------
TR|Q6JH47|Q6JH47_CVHSA              ------------------------------------------------------------
TR|Q692E5|Q692E5_CVHSA              ------------------------------------------------------------
SP|P0C6F8|R1A_BCHK3                 ------------------------------------------------------------
TR|A0A0K1Z0N1|A0A0K1Z0N1_CVHSA ------------------------------------------------------------
SP|P0C6F5|R1A_BC279                 ------------------------------------------------------------
SP|P0C6T7|R1A_BCRP3                 ------------------------------------------------------------
```

```
QHN73794                        IVYTACSHAAVDALCEKALKYLPIDKCSRIIPARARVECFDKFKVNSTLEQYVFCTVNAL 5687
SP|P0C6X7|R1AB_CVHSA            IVYTACSHAAVDALCEKALKYLPIDKCSRIIPARARVECFDKFKVNSTLEQYVFCTVNAL 5664
TR|Q6UZF5|Q6UZF5_CVHSA          IVYTACSHAAVDALCEKALKYLPIDKCSRIIPARARVECFDKFKVNSTLEQYVFCTVNAL 5664
TR|Q6UZF1|Q6UZF1_CVHSA          IVYTACSHAAVDALCEKALKYLPIDKCSRIIPARARVECFDKFKVNSTLEQYVFCTVNAL 5664
TR|Q6JH48|Q6JH48_CVHSA          IVYTACSHAAVDALCEKALKYLPIDKCSRIIPARARVECFDKFKVNSTLEQYVFCTVNAL 5664
TR|Q692E6|Q692E6_CVHSA          IVYTACSHAAVDALCEKALKYLPIDKCSRIIPARARVECFDKFKVNSTLEQYVFCTVNAL 5664
TR|A0A0K1YZY7|A0A0K1YZY7_CVHSA IVYTACSHAAVDALCEKALKYLPIDKCSRIIPARARVECFDKFKVNSTLEQYVFCTVNAL 5664
SP|P0C6W2|R1AB_BCHK3            IVYTACSHAAVDALCEKALKYLPIDKCSRIIPARARVECFDKFKVNSTLEQYVFCTVNAL 5658
SP|P0C6W6|R1AB_BCRP3            IVYTACSHAAVDALCEKALKYLPIDKCSRIIPARARVECFDKFKVNSTLEQYVFCTVNAL 5662
SP|P0C6V9|R1AB_BC279            IVYTACSHAAVDALCEKALKYLPIDKCSRIIPARARVECFDKFKVNSTLEQYVFCTVNAL 5670
TR|A0A0U1WHI4|A0A0U1WHI4_CVHSA IVYTACSHAAVDALCEKALKYLPIDKCSRIIPARARVECFDKFKVNSTLEQYVFCTVNAL 5659
TR|A0A0U1WHG0|A0A0U1WHG0_CVHSA IVYTACSHAAVDALCEKALKYLPIDKCSRIIPARARVECFAKFKVNSTLEQYVFCTVNAL 5659
TR|A0A166ZL34|A0A166ZL34_9NIDO IVYTACSHAAVDALCEKALKYLPIDKCSRIIPARARVECFAKFKVNSTLEQYVFCTVNAL 5466
TR|R9QTB2|R9QTB2_CVHSA          ------------------------------------------------------------
TR|R9QTH2|R9QTH2_CVHSA          ------------------------------------------------------------
SP|P0C6U8|R1A_CVHSA             ------------------------------------------------------------
TR|Q6JH47|Q6JH47_CVHSA          ------------------------------------------------------------
TR|Q692E5|Q692E5_CVHSA          ------------------------------------------------------------
SP|P0C6F8|R1A_BCHK3             ------------------------------------------------------------
TR|A0A0K1Z0N1|A0A0K1Z0N1_CVHSA ------------------------------------------------------------
SP|P0C6F5|R1A_BC279             ------------------------------------------------------------
SP|P0C6T7|R1A_BCRP3             ------------------------------------------------------------

QHN73794                        PETTADIVVFDEISMATNYDLSVVNARLRAKHYVYIGDPAQLPAPRTLLTKGTLEPEYFN 5747
SP|P0C6X7|R1AB_CVHSA            PETTADIVVFDEISMATNYDLSVVNARLRAKHYVYIGDPAQLPAPRTLLTKGTLEPEYFN 5724
TR|Q6UZF5|Q6UZF5_CVHSA          PETTADIVVFDEISMATNYDLSVVNARLRAKHYVYIGDPAQLPAPRTLLTKGTLEPEYFN 5724
TR|Q6UZF1|Q6UZF1_CVHSA          PETTADIVVFDEISMATNYDLSVVNARLRAKHYVYIGDPAQLPAPRTLLTKGTLEPEYFN 5724
TR|Q6JH48|Q6JH48_CVHSA          PETTADIVVFDEISMATNYDLSVVNARLRAKHYVYIGDPAQLPAPRTLLTKGTLEPEYFN 5724
TR|Q692E6|Q692E6_CVHSA          PETTADIVVFDEISMATNYDLSVVNARLRAKHYVYIGDPAQLPAPRTLLTKGTLEPEYFN 5724
TR|A0A0K1YZY7|A0A0K1YZY7_CVHSA PETTADIVVFDEISMATNYDLSVVNARLRAKHYVYIGDPAQLPAPRTLLTKGTLEPEYFN 5724
SP|P0C6W2|R1AB_BCHK3            PETTADIVVFDEISMATNYDLSVVNARLRAKHYVYIGDPAQLPAPRTLLTKGTLEPEYFN 5718
SP|P0C6W6|R1AB_BCRP3            PETTADIVVFDEISMATNYDLSVVNARLRAKHYVYIGDPAQLPAPRTLLTKGTLEPEYFN 5722
SP|P0C6V9|R1AB_BC279            PETTADIVVFDEISMATNYDLSVVNARLRAKHYVYIGDPAQLPAPRTLLTKGTLEPEYFN 5730
TR|A0A0U1WHI4|A0A0U1WHI4_CVHSA PETTADIVVFDEISMATNYDLSVVNARLRAKHYVYIGDPAQLPAPRTLLTKGTLEPEYFN 5719
TR|A0A0U1WHG0|A0A0U1WHG0_CVHSA PETTADIVVFDEISMATNYDLSVVNARLRAKHYVYIGDPAQLPAPRTLLTKGTLEPEYFN 5719
TR|A0A166ZL34|A0A166ZL34_9NIDO PETTADIVVFDEISMATNYDLSVVNARLRAKHYVYIGDPAQLPAPRTLLTKGTLEPEYFN 5526
TR|R9QTB2|R9QTB2_CVHSA          ------------------------------------------------------------
TR|R9QTH2|R9QTH2_CVHSA          ------------------------------------------------------------
SP|P0C6U8|R1A_CVHSA             ------------------------------------------------------------
TR|Q6JH47|Q6JH47_CVHSA          ------------------------------------------------------------
TR|Q692E5|Q692E5_CVHSA          ------------------------------------------------------------
SP|P0C6F8|R1A_BCHK3             ------------------------------------------------------------
TR|A0A0K1Z0N1|A0A0K1Z0N1_CVHSA ------------------------------------------------------------
SP|P0C6F5|R1A_BC279             ------------------------------------------------------------
SP|P0C6T7|R1A_BCRP3             ------------------------------------------------------------

QHN73794                        SVCRLMKTIGPDMFLGTCRRCPAEIVDTVSALVYDNKLKAHKDKSAQCFKMFYKGVITHD 5807
SP|P0C6X7|R1AB_CVHSA            SVCRLMKTIGPDMFLGTCRRCPAEIVDTVSALVYDNKLKAHKDKSAQCFKMFYKGVITHD 5784
TR|Q6UZF5|Q6UZF5_CVHSA          SVCRLMKTIGPDMFLGTCRRCPAEIVDTVSALVYDNKLKAHKDKSAQCFKMFYKGVITHD 5784
TR|Q6UZF1|Q6UZF1_CVHSA          SVCRLMKTIGPDMFLGTCRRCPAEIVDTVSALVYDNKLKAHKDKSAQCFKMFYKGVITHD 5784
TR|Q6JH48|Q6JH48_CVHSA          SVCRLMKTIGPDMFLGTCRRCPAEIVDTVSALVYDNKLKAHKDKSAQCFKMFYKGVITHD 5784
TR|Q692E6|Q692E6_CVHSA          SVCRLMKTIGPDMFLGTCRRCPAEIVDTVSALVYDNKLKAHKEKSAQCFKMFYKGVITHD 5784
TR|A0A0K1YZY7|A0A0K1YZY7_CVHSA SVCRLMKTIGPDMFLGTCRRCPAEIVDTVSALVYDNKLKAHKEKSAQCFKMFYKGVITHD 5784
SP|P0C6W2|R1AB_BCHK3            SVCRLMKTIGPDMFLGTCRRCPAEIVDTVSALVYDNKLKAHKEKSAQCFKMYYKGVITHD 5778
SP|P0C6W6|R1AB_BCRP3            SVCRLMKTIGPDMFLGTCRRCPAEIVDTVSALVYDNKLKAHKEKSAQCFKMFYKGVITHD 5782
SP|P0C6V9|R1AB_BC279            SVCRLMKTIGPDMFLGTCRRCPAEIVDTVSALVYDNKLKAHKEKSAQCFKMFYKGVITHD 5790
TR|A0A0U1WHI4|A0A0U1WHI4_CVHSA SVCRLMKTIGPDMFLGTCRRCPAEIVDTVSALVYDNKLKAHKEKSAQCFKMFYKGVITHD 5779
TR|A0A0U1WHG0|A0A0U1WHG0_CVHSA SVCRLMKTIGPDMFLGTCRRCPAEIVDTVSALVYDNKLKAHKEKSAQCFKMFYKGVITHD 5779
TR|A0A166ZL34|A0A166ZL34_9NIDO SVCRLMKTIGPDMFLGTCRRCPAEIVDTVSALVYDNKLKAHKEKSAQCFKMFYKGVITHD 5586
TR|R9QTB2|R9QTB2_CVHSA          ------------------------------------------------------------
TR|R9QTH2|R9QTH2_CVHSA          ------------------------------------------------------------
SP|P0C6U8|R1A_CVHSA             ------------------------------------------------------------
TR|Q6JH47|Q6JH47_CVHSA          ------------------------------------------------------------
TR|Q692E5|Q692E5_CVHSA          ------------------------------------------------------------
SP|P0C6F8|R1A_BCHK3             ------------------------------------------------------------
TR|A0A0K1Z0N1|A0A0K1Z0N1_CVHSA ------------------------------------------------------------
SP|P0C6F5|R1A_BC279             ------------------------------------------------------------
SP|P0C6T7|R1A_BCRP3             ------------------------------------------------------------
```

```
QHN73794                          VSSAINRPQIGVVREFLTRNPAWRKAVFISPYNSQNAVASKILGLPTQTVDSSQGSEYDY 5867
SP|P0C6X7|R1AB_CVHSA              VSSAINRPQIGVVREFLTRNPAWRKAVFISPYNSQNAVASKILGLPTQTVDSSQGSEYDY 5844
TR|Q6UZF5|Q6UZF5_CVHSA            VSSAINRPQIGVVREFLTRNPAWRKAVFISPYNSQNAVASKILGLPTQTVDSSQGSEYDY 5844
TR|Q6UZF1|Q6UZF1_CVHSA            VSSAINRPQIGVVREFLTRNPAWRKAVFISPYNSQNAVASKILGLPTQTVDSSQGSEYDY 5844
TR|Q6JH48|Q6JH48_CVHSA            VSSAINRPQIGVVREFLTRNPAWRKAVFISPYNSQNAVASKILGLPTQTVDSSQGSEYDY 5844
TR|Q692E6|Q692E6_CVHSA            VSSAINRPQIGVVREFLTRNPAWRKAVFISPYNSQNAVASKILGLPTQTVDSSQGSEYDY 5844
TR|A0A0K1YZY7|A0A0K1YZY7_CVHSA    VSSAINRPQIGVVREFLTRNPAWRKAVFISPYNSQNAVASKILGLPTQTVDSSQGSEYDY 5844
SP|P0C6W2|R1AB_BCHK3              VSSAINRPQIGVVREFLTRNPAWRKAVFISPYNSQNAVASKILGLPTQTVDSSQGSEYDY 5838
SP|P0C6W6|R1AB_BCRP3              VSSAINRPQIGVVREFLTRNPAWRKAVFISPYNSQNAVASKILGLPTQTVDSSQGSEYDY 5842
SP|P0C6V9|R1AB_BC279              VSSAINRPQIGVVREFLTRNPAWRKAVFISPYNSQNAVASKILGLPTQTVDSSQGSEYDY 5850
TR|A0A0U1WHI4|A0A0U1WHI4_CVHSA    VSSAINRPQIGVVREFLTRNPAWRKAVFISPYNSQNAVASKILGLPTQTVDSSQGSEYDY 5839
TR|A0A0U1WHG0|A0A0U1WHG0_CVHSA    VSSAINRPQIGVVREFLTRNPVWRKAVFISPYNSQNAVASKILGLPTQTVDSSQGSEYDY 5839
TR|A0A166ZL34|A0A166ZL34_9NIDO    VSSAINRPQIGVVREFLTRNPVWRKAVFISPYNSQNAVASKILGLPTQTVDSSQGSEYDY 5646
TR|R9QTB2|R9QTB2_CVHSA            ------------------------------------------------------------
TR|R9QTH2|R9QTH2_CVHSA            ------------------------------------------------------------
SP|P0C6U8|R1A_CVHSA               ------------------------------------------------------------
TR|Q6JH47|Q6JH47_CVHSA            ------------------------------------------------------------
TR|Q692E5|Q692E5_CVHSA            ------------------------------------------------------------
SP|P0C6F8|R1A_BCHK3               ------------------------------------------------------------
TR|A0A0K1Z0N1|A0A0K1Z0N1_CVHSA    ------------------------------------------------------------
SP|P0C6F5|R1A_BC279               ------------------------------------------------------------
SP|P0C6T7|R1A_BCRP3               ------------------------------------------------------------

QHN73794                          VIFTQTTETAHSCNVNRFNVAITRAKVGILCIMSDRDLYDKLQFTSLEIPRRNVATLQAE 5927
SP|P0C6X7|R1AB_CVHSA              VIFTQTTETAHSCNVNRFNVAITRAKIGILCIMSDRDLYDKLQFTSLEIPRRNVATLQAE 5904
TR|Q6UZF5|Q6UZF5_CVHSA            VIFTQTTETAHSCNVNRFNVAITRAKIGILCIMSDRDLYDKLQFTSLEIPRRNVATLQAE 5904
TR|Q6UZF1|Q6UZF1_CVHSA            VIFTQTTETAHSCNVNRFNVAITRAKIGILCIMSDRDLYDKLQFTSLEIPRRNVATLQAE 5904
TR|Q6JH48|Q6JH48_CVHSA            VIFTQTTETAHSCNVNRFNVAITRAKIGILCIMSDRDLYDKLQFTSLEIPRRNVATLQAE 5904
TR|Q692E6|Q692E6_CVHSA            VIFTQTTETAHSCNVNRFNVAITRAKIGILCIMSDRDLYDKLQFTSLEIPRRNVATLQAE 5904
TR|A0A0K1YZY7|A0A0K1YZY7_CVHSA    VIFTQTTETAHSCNVNRFNVAITRAKIGILCIMSDRDLYDKLQFTSLEVPRRNVATLQAE 5904
SP|P0C6W2|R1AB_BCHK3              VIFTQTTETAHSCNVNRFNVAITRAKIGILCIMSDRDLYDKLQFTSLEVPRRNVATLQAE 5898
SP|P0C6W6|R1AB_BCRP3              VIFTQTTETAHSCNVNRFNVAITRAKIGILCIMSDRDLYDKLQFTSLEVPRRNVATLQAE 5902
SP|P0C6V9|R1AB_BC279              VIFTQTTETAHSCNVNRFNVAITRAKIGILCIMSDRDLYDKLQFTSLEVPRRNVATLQAE 5910
TR|A0A0U1WHI4|A0A0U1WHI4_CVHSA    VIFTQTTETAHSCNVNRFNVAITRAKIGILCIMSDRDLYDKLQFTSLEVPRRNVATLQAE 5899
TR|A0A0U1WHG0|A0A0U1WHG0_CVHSA    VIFTQTTETAHSCNVNRFNVAITRAKIGILCIMSDRDLYDKLQFTSLEVPRRNVATLQAE 5899
TR|A0A166ZL34|A0A166ZL34_9NIDO    VIFTQTTETAHSCNVNRFNVAITRAKIGILCIMSDRDLYDKLQFTSLEVPRRNVATLQAE 5706
TR|R9QTB2|R9QTB2_CVHSA            ------------------------------------------------------------
TR|R9QTH2|R9QTH2_CVHSA            ------------------------------------------------------------
SP|P0C6U8|R1A_CVHSA               ------------------------------------------------------------
TR|Q6JH47|Q6JH47_CVHSA            ------------------------------------------------------------
TR|Q692E5|Q692E5_CVHSA            ------------------------------------------------------------
SP|P0C6F8|R1A_BCHK3               ------------------------------------------------------------
TR|A0A0K1Z0N1|A0A0K1Z0N1_CVHSA    ------------------------------------------------------------
SP|P0C6F5|R1A_BC279               ------------------------------------------------------------
SP|P0C6T7|R1A_BCRP3               ------------------------------------------------------------

QHN73794                          NVTGLFKDCSKVITGLHPTQAPTHLSVDTKFKTEGLCVDIPGIPKDMTYRRLISMMGFKM 5987
SP|P0C6X7|R1AB_CVHSA              NVTGLFKDCSKIITGLHPTQAPTHLSVDIKFKTEGLCVDIPGIPKDMTYRRLISMMGFKM 5964
TR|Q6UZF5|Q6UZF5_CVHSA            NVTGLFKDCSKIITGLHPTQAPTHLSVDIKFKTEGLCVDIPGIPKDMTYRRLISMMGFKM 5964
TR|Q6UZF1|Q6UZF1_CVHSA            NVTGLFKDCSKIITGLHPTQAPTHLSVDIKFKTEGLCVDIPGIPKDMTYRRLISMMGFKM 5964
TR|Q6JH48|Q6JH48_CVHSA            NVTGLFKDCSKIITGLHPTQAPTHLSVDIKFKTEGLCVDIPGIPKDMTYRRLISMMGFKM 5964
TR|Q692E6|Q692E6_CVHSA            NVTGLFKDCSKIITGLHPTQAPTHLSVDIKFKTEGLCVDIPGIPKDMTYRRLISMMGFKM 5964
TR|A0A0K1YZY7|A0A0K1YZY7_CVHSA    NVTGLFKDCSKIITGLHPTQAPTHLSVDTKFKTEGLCVDIPGIPKDMTYRRLISMMGFKM 5964
SP|P0C6W2|R1AB_BCHK3              NVTGLFKDCSKIITGLHPTQAPTHLSVDTKFKTEGLCVDIPGIPKDMTYRRLISMMGFKM 5958
SP|P0C6W6|R1AB_BCRP3              NVTGLFKDCSKIITGLHPTQAPTHLSVDTKFKTEGLCVDIPGIPKDMTYRRLISMMGFKM 5962
SP|P0C6V9|R1AB_BC279              NVTGLFKDCSKIITGLHPTQAPTHLSVDTKFKTEGLCVDIPGIPKDMTYRRLISMMGFKM 5970
TR|A0A0U1WHI4|A0A0U1WHI4_CVHSA    NVTGLFKDCSKIITGLHPTQAPTHLSVDTKFKTEGLCVDIPGIPKDMTYRRLISMMGFKM 5959
TR|A0A0U1WHG0|A0A0U1WHG0_CVHSA    NVTGLFKDCSKIITGLHPTQAPTHLSVDTKFKTEGLCVDIPGIPKDMTYRRLISMMGFKM 5959
TR|A0A166ZL34|A0A166ZL34_9NIDO    NVTGLFKDCSKIITGLHPTQAPTHLSVDTKFKTEGLCVDIPGIPKDMTYRRLISMMGFKM 5766
TR|R9QTB2|R9QTB2_CVHSA            ------------------------------------------------------------
TR|R9QTH2|R9QTH2_CVHSA            ------------------------------------------------------------
SP|P0C6U8|R1A_CVHSA               ------------------------------------------------------------
TR|Q6JH47|Q6JH47_CVHSA            ------------------------------------------------------------
TR|Q692E5|Q692E5_CVHSA            ------------------------------------------------------------
SP|P0C6F8|R1A_BCHK3               ------------------------------------------------------------
TR|A0A0K1Z0N1|A0A0K1Z0N1_CVHSA    ------------------------------------------------------------
SP|P0C6F5|R1A_BC279               ------------------------------------------------------------
SP|P0C6T7|R1A_BCRP3               ------------------------------------------------------------
```

```
QHN73794                              NYQVNGYPNMFITREEAIRHVRAWIGFDVEGCHATREAVGTNLPLQLGFSTGVNLVAVPT 6047
SP|P0C6X7|R1AB_CVHSA                  NYQVNGYPNMFITREEAIRHVRAWIGFDVEGCHATRDAVGTNLPLQLGFSTGVNLVAVPT 6024
TR|Q6UZF5|Q6UZF5_CVHSA                NYQVNGYPNMFITREEAIRHVRAWIGFDVEGCHATRDAVGTNLPLQLGFSTGVNLVAVPT 6024
TR|Q6UZF1|Q6UZF1_CVHSA                NYQVNGYPNMFITREEAIRHVRAWIGFDVEGCHATRDAVGTNLPLQLGFSTGVNLVAVPT 6024
TR|Q6JH48|Q6JH48_CVHSA                NYQVNGYPNMFITREEAIRHVRAWIGFDVEGCHATRDAVGTNLPLQLGFSTGVNLVAVPT 6024
TR|Q692E6|Q692E6_CVHSA                NYQVNGYPNMFITREEAIRHVRAWIGFDVEGCHATRDAVGTNLPLQLGFSTGVNLVAVPT 6024
TR|A0A0K1YZY7|A0A0K1YZY7_CVHSA        NYQVNGYPNMFITREEAIRHVRAWIGFDVEGCHATRDAVGTNLPLQLGFSTGVNLVAVPT 6024
SP|P0C6W2|R1AB_BCHK3                  NYQVNGYPNMFITREEAIRHVRAWIGFDVEGCHATRDAVGTNLPLQLGFSTGVNLVAVPT 6018
SP|P0C6W6|R1AB_BCRP3                  NYQVNGYPNMFITREEAIRHVRAWIGFDVEGCHATRDAVGTNLPLQLGFSTGVNLVAIPT 6022
SP|P0C6V9|R1AB_BC279                  NYQVNGYPNMFITREEAIRHVRAWIGFDVEGCHATRDAVGTNLPLQLGFSTGVNLVAVPT 6030
TR|A0A0U1WHI4|A0A0U1WHI4_CVHSA        NYQVNGYPNMFITREEAIRHVRAWIGFDVEGCHATRDAVGTNLPLQLGFSTGVNLVAVPT 6019
TR|A0A0U1WHG0|A0A0U1WHG0_CVHSA        NYQVNGYPNMFITREEAIRHVRAWIGFDVEGCHATRDAVGTNLPLQLGFSTGVNLVAVPT 6019
TR|A0A166ZL34|A0A166ZL34_9NIDO        NYQVNGYPNMFITREEAIRHVRAWIGFDVEGCHATRDAVGTNLPLQLGFSTGVNLVAVPT 5826
TR|R9QTB2|R9QTB2_CVHSA                ------------------------------------------------------------
TR|R9QTH2|R9QTH2_CVHSA                ------------------------------------------------------------
SP|P0C6U8|R1A_CVHSA                   ------------------------------------------------------------
TR|Q6JH47|Q6JH47_CVHSA                ------------------------------------------------------------
TR|Q692E5|Q692E5_CVHSA                ------------------------------------------------------------
SP|P0C6F8|R1A_BCHK3                   ------------------------------------------------------------
TR|A0A0K1Z0N1|A0A0K1Z0N1_CVHSA        ------------------------------------------------------------
SP|P0C6F5|R1A_BC279                   ------------------------------------------------------------
SP|P0C6T7|R1A_BCRP3                   ------------------------------------------------------------

QHN73794                              GYVDTPNNTDFSRVSAKPPPGDQFKHLIPLMYKGLPWNVVRIKIVQMLSDTLKNLSDRVV 6107
SP|P0C6X7|R1AB_CVHSA                  GYVDTENNTEFTRVNAKPPPGDQFKHLIPLMYKGLPWNVVRIKIVQMLSDTLKGLSDRVV 6084
TR|Q6UZF5|Q6UZF5_CVHSA                GYVDTENNTEFTRVNAKPPPGDQFKHLIPLMYKGLPWNVVRIKIVQMLSDTLKGLSDRVV 6084
TR|Q6UZF1|Q6UZF1_CVHSA                GYVDTENNTEFTRVNAKPPPGDQFKHLIPLMYKGLPWNVVRIKIVQMLSDTLKGLSDRVV 6084
TR|Q6JH48|Q6JH48_CVHSA                GYVDTENNTEFTRVNAKPPPGDQFKHLIPLMYKGLPWNVVRIKIVQMLSDTLKGLSDRVV 6084
TR|Q692E6|Q692E6_CVHSA                GYVDTENNTEFTRVNAKPPPGDQFKHLIPLMYKGLPWNVVRIKIVQMLSDTLKGLSDRVV 6084
TR|A0A0K1YZY7|A0A0K1YZY7_CVHSA        GYVDTENNTEFTRVNAKPPPGDQFKHLIPLMYKGLPWNVVRIKIVQMLSDTLKGLSDRVV 6084
SP|P0C6W2|R1AB_BCHK3                  GYVDTENSTEFTRVNAKPPPGDQFKHLIPLMYKGLPWNVVRIKIVQMLSDTLKGLSDRVV 6078
SP|P0C6W6|R1AB_BCRP3                  GYVDTENNTEFTRVNAKPPPGDQFKHLIPLMYKGLPWNVVRIKIVQMLSDTLKGLSDRVV 6082
SP|P0C6V9|R1AB_BC279                  GYVDTENNTEFTRVNAKPPPGDQFKHLIPLMYKGLPWNVVRIKIVQMLSDTLKGLSDRVV 6090
TR|A0A0U1WHI4|A0A0U1WHI4_CVHSA        GYVDTENNTEFTRVNAKPPPGDQFKHLIPLMYKGLPWNVVRIKIVQMLSDTLKGLSDRVV 6079
TR|A0A0U1WHG0|A0A0U1WHG0_CVHSA        GYVDTENNTEFTRVNAKPPPGDQFKHLIPLMYKGLPWSVVRIKIVQMLSDTLKGLSDRVV 6079
TR|A0A166ZL34|A0A166ZL34_9NIDO        GYVDTENNTEFTRVNAKPPPGDQFKHLIPLMYKGLPWSVVRIKIVQMLSDTLKGLSDRVV 5886
TR|R9QTB2|R9QTB2_CVHSA                ------------------------------------------------------------
TR|R9QTH2|R9QTH2_CVHSA                ------------------------------------------------------------
SP|P0C6U8|R1A_CVHSA                   ------------------------------------------------------------
TR|Q6JH47|Q6JH47_CVHSA                ------------------------------------------------------------
TR|Q692E5|Q692E5_CVHSA                ------------------------------------------------------------
SP|P0C6F8|R1A_BCHK3                   ------------------------------------------------------------
TR|A0A0K1Z0N1|A0A0K1Z0N1_CVHSA        ------------------------------------------------------------
SP|P0C6F5|R1A_BC279                   ------------------------------------------------------------
SP|P0C6T7|R1A_BCRP3                   ------------------------------------------------------------

QHN73794                              FVLWAHGFELTSMKYFVKIGPERTCCLCDRRATCFSTASDTYACWHHSIGFDYVYNPFMI 6167
SP|P0C6X7|R1AB_CVHSA                  FVLWAHGFELTSMKYFVKIGPERTCCLCDKRATCFSTSSDTYACWNHSVGFDYVYNPFMI 6144
TR|Q6UZF5|Q6UZF5_CVHSA                FVLWAHGFELTSMKYFVKIGPERTCCLCDKRATCFSTSSDTYACWNHSVGFDYVYNPFMI 6144
TR|Q6UZF1|Q6UZF1_CVHSA                FVLWAHGFELTSMKYFVKIGPERTCCLCDKRATCFSTSSDTYACWNHSVGFDYVYNPFMI 6144
TR|Q6JH48|Q6JH48_CVHSA                FVLWAHGFELTSMKYFVKIGPERTCCLCDKRATCFSTSSDTYACWNHSVGFDYVYNPFMI 6144
TR|Q692E6|Q692E6_CVHSA                FVLWAHGFELTSMKYFVKIGPERTCCLCDKRATCFSTSSDTYACWNHSVGFDYVYNPFMI 6144
TR|A0A0K1YZY7|A0A0K1YZY7_CVHSA        FVLWAHGFELTSMKYFVKIGPERTCCLCDKRATCFSTSSDTYACWNHSVGFDYVYNPFMI 6144
SP|P0C6W2|R1AB_BCHK3                  FVLWAHGFELTSMKYFVKIGPERTCCLCDKRATCFSTSSDTYACWNHSVGFDYVYNPFMI 6138
SP|P0C6W6|R1AB_BCRP3                  FVLWAHGFELTSMKYFVKIGPERTCCLCDKRATCFSTSSDTYACWNHSVGFDYVYNPFMI 6142
SP|P0C6V9|R1AB_BC279                  FVLWAHGFELTSMKYFVKIGPERTCCLCDRRATCFSTSSDTYACWNHSVGFDYVYNPFMI 6150
TR|A0A0U1WHI4|A0A0U1WHI4_CVHSA        FVLWAHGFELTSMKYFVKIGPERTCCLCDKRATCFSTSSDTYACWNHSVGFDYVYNPFMI 6139
TR|A0A0U1WHG0|A0A0U1WHG0_CVHSA        FVLWAHGFELTSMKYFVKIGSERTCCLCDKRATCFSTSSDTYACWNHSVGFDYVYNPFMI 6139
TR|A0A166ZL34|A0A166ZL34_9NIDO        FVLWAHGFELTSMKYFVKIGSERTCCLCDKRATCFSTSSDTYACWNHSVGFDYVYNPFMI 5946
TR|R9QTB2|R9QTB2_CVHSA                ------------------------------------------------------------
TR|R9QTH2|R9QTH2_CVHSA                ------------------------------------------------------------
SP|P0C6U8|R1A_CVHSA                   ------------------------------------------------------------
TR|Q6JH47|Q6JH47_CVHSA                ------------------------------------------------------------
TR|Q692E5|Q692E5_CVHSA                ------------------------------------------------------------
SP|P0C6F8|R1A_BCHK3                   ------------------------------------------------------------
TR|A0A0K1Z0N1|A0A0K1Z0N1_CVHSA        ------------------------------------------------------------
SP|P0C6F5|R1A_BC279                   ------------------------------------------------------------
SP|P0C6T7|R1A_BCRP3                   ------------------------------------------------------------
```

```
QHN73794                       DVQQWGFTGNLQSNHDLYCQVHGNAHVASCDAIMTRCLAVHECFVKRVDWTIEYPIIGDE 6227
SP|P0C6X7|R1AB_CVHSA           DVQQWGFTGNLQSNHDQHCQVHGNAHVASCDAIMTRCLAVHECFVKRVDWSVEYPIIGDE 6204
TR|Q6UZF5|Q6UZF5_CVHSA         DVQQWGFTGNLQSNHDQHCQVHGNAHVASCDAIMTRCLAVHECFVKRVDWSVEYPIIGDE 6204
TR|Q6UZF1|Q6UZF1_CVHSA         DVQQWGFTGNLQSNHDQHCQVHGNAHVASCDAIMTRCLAVHECFVKRVDWSVEYPIIGDE 6204
TR|Q6JH48|Q6JH48_CVHSA         DVQQWGFTGNLQSNHDQHCQVHGNAHVASCDAIMTRCLAVHECFVKRVDWSVEYPIIGDE 6204
TR|Q692E6|Q692E6_CVHSA         DVQQWGFTGNLQSNHDQHCQVHGNAHVASCDAIMTRCLAVHECFVKRVDWSVEYPIIGDE 6204
TR|A0A0K1YZY7|A0A0K1YZY7_CVHSA DVQQWGFTGNLQSNHDQHCQVHGNAHVASCDAIMTRCLAVHECFVKRVDWSVEYPIIGDE 6204
SP|P0C6W2|R1AB_BCHK3           DVQQWGFTGNLQSNHDQHCQVHGNAHVASCDAIMTRCLAVHECFVKRVDWSVEYPIIGDE 6198
SP|P0C6W6|R1AB_BCRP3           DVQQWGFTGNLQSNHDQHCQVHGNAHVASCDAIMTRCLAVHECFVKRVDWSVEYPIIGDE 6202
SP|P0C6V9|R1AB_BC279           DVQQWGFTGNLQSNHDQHCQVHGNAHVASCDAIMTRCLAVHECFVKRVDWSVEYPIIGDE 6210
TR|A0A0U1WHI4|A0A0U1WHI4_CVHSA DVQQWGLTGNLQSNHDQHCQVHGNAHVASCDAIMTRCLAVHECFVKRVDWSVEYPIIGDE 6199
TR|A0A0U1WHG0|A0A0U1WHG0_CVHSA DVQQWGFTGNLQSNHDQHCQVHGNAHVASCDAIMTRCLAVHECFVKRVDWSVEYPVIGDE 6199
TR|A0A166ZL34|A0A166ZL34_9NIDO DVQQWGFTGNLQSNHDQHCQVHGNAHVASCDAIMTRCLAVHECFVKRVDWSVEYPVIGDE 6006
TR|R9QTB2|R9QTB2_CVHSA         ------------------------------------------------------------
TR|R9QTH2|R9QTH2_CVHSA         ------------------------------------------------------------
SP|P0C6U8|R1A_CVHSA            ------------------------------------------------------------
TR|Q6JH47|Q6JH47_CVHSA         ------------------------------------------------------------
TR|Q692E5|Q692E5_CVHSA         ------------------------------------------------------------
SP|P0C6F8|R1A_BCHK3            ------------------------------------------------------------
TR|A0A0K1Z0N1|A0A0K1Z0N1_CVHSA ------------------------------------------------------------
SP|P0C6F5|R1A_BC279            ------------------------------------------------------------
SP|P0C6T7|R1A_BCRP3            ------------------------------------------------------------

QHN73794                       LKINAACRKVQHMVVKAALLADKFPVLHDIGNPKAIKCVPQADVEWKFYDAQPCSDKAYK 6287
SP|P0C6X7|R1AB_CVHSA           LRVNSACRKVQHMVVKSALLADKFPVLHDIGNPKAIKCVPQAEVEWKFYDAQPCSDKAYK 6264
TR|Q6UZF5|Q6UZF5_CVHSA         LRVNSACRKVQHMVVKSALLADKFPVLHDIGNPKAIKCVPQAEVEWKFYDAQPCSDKAYK 6264
TR|Q6UZF1|Q6UZF1_CVHSA         LRVNSACRKVQHMVVKSALLADKFPVLHDIGNPKAIKCVPQAEVEWKFYDAQPCSDKAYK 6264
TR|Q6JH48|Q6JH48_CVHSA         LRVNSACRKVQHMVVKSALLADKFPVLHDIGNPKAIKCVPQAEVEWKFYDAQPCSDKAYK 6264
TR|Q692E6|Q692E6_CVHSA         LRVNSACRKVQHMVVKSALLADKFPVLHDIGNPKAIKCVPQAEVEWKFYDAQPCSDKAYK 6264
TR|A0A0K1YZY7|A0A0K1YZY7_CVHSA LKINSACRKVQHMVVKSALLADKFPVLHDIGNPKAIKCVPQAEVEWKFYDAQPCSDKAYK 6264
SP|P0C6W2|R1AB_BCHK3           LKINAACRKVQHMVVKSALLADKFTVLHDIGNPKAIRCVPQAEVDWKFYDAQPCSDKAYK 6258
SP|P0C6W6|R1AB_BCRP3           LKINSACRKVQHMVVKSALLADKFPVLHDIGNPKAIKCVPQAEVEWKFYDAQPCSDKAYK 6262
SP|P0C6V9|R1AB_BC279           LKINAACRKVQHMVVKSALLADKFSVLHDIGNPKAIKCVPQAEVDWKFYDAQPCSDKAYK 6270
TR|A0A0U1WHI4|A0A0U1WHI4_CVHSA LKINAACRKVQHMVVKSALLADKFPVLHDIGNPKAIKCVPQADVEWKFYDAQPCSDKAYK 6259
TR|A0A0U1WHG0|A0A0U1WHG0_CVHSA LKINAACRKVQHMVVKSALLADKFPVLHDIGNPKAIKCVPQADVEWKFYDVQPCSDKAYK 6259
TR|A0A166ZL34|A0A166ZL34_9NIDO LKINAACRKVQHMVVKSALLADKFPVLHDIGNPKAIKCVPQADVEWKFYDVQPCSDKAYK 6066
TR|R9QTB2|R9QTB2_CVHSA         ------------------------------------------------------------
TR|R9QTH2|R9QTH2_CVHSA         ------------------------------------------------------------
SP|P0C6U8|R1A_CVHSA            ------------------------------------------------------------
TR|Q6JH47|Q6JH47_CVHSA         ------------------------------------------------------------
TR|Q692E5|Q692E5_CVHSA         ------------------------------------------------------------
SP|P0C6F8|R1A_BCHK3            ------------------------------------------------------------
TR|A0A0K1Z0N1|A0A0K1Z0N1_CVHSA ------------------------------------------------------------
SP|P0C6F5|R1A_BC279            ------------------------------------------------------------
SP|P0C6T7|R1A_BCRP3            ------------------------------------------------------------

QHN73794                       IEELFYSYATHSDKFTDGVCLFWNCNVDRYPANSIVCRFDTRVLSNLNLPGCDGGSLYVN 6347
SP|P0C6X7|R1AB_CVHSA           IEELFYSYATHHDKFTDGVCLFWNCNVDRYPANAIVCRFDTRVLSNLNLPGCDGGSLYVN 6324
TR|Q6UZF5|Q6UZF5_CVHSA         IEELFYSYATHHDKFTDGVCLFWNCNVDRYPANAIVCRFDTRVLSNLNLPGCDGGSLYVN 6324
TR|Q6UZF1|Q6UZF1_CVHSA         IEELFYSYATHHDKFTDGVCLFWNCNVDRYPANAIVCRFDTRVLSNLNLPGCDGGSLYVN 6324
TR|Q6JH48|Q6JH48_CVHSA         IEELFYSYATHHDKFTDGVCLFWNCNVDRYPANAIVCRFDTRVLSNLNLPGCDGGSLYVN 6324
TR|Q692E6|Q692E6_CVHSA         IEELFYSYATHHDKFTDGVCLFWNCNVDRYPANAIVCRFDTRVLSNLNLPGCDGGSLYVN 6324
TR|A0A0K1YZY7|A0A0K1YZY7_CVHSA IEELFYSYATHHDKFTDGVCLFWNCNVDRYPANAIVCRFDTRVLSNLNLPGCDGGSLYVN 6324
SP|P0C6W2|R1AB_BCHK3           IEELFYSYATHHDKFTDGVCLFWNCNVDRYPANAIVCRFDTRVLSNLNLPGCDGGSLYVN 6318
SP|P0C6W6|R1AB_BCRP3           IEELFYSYATHHDKFTDGVCLFWNCNVDRYPANAIVCRFDTRVLSNLNLPGCDGGSLYVN 6322
SP|P0C6V9|R1AB_BC279           IEELFYSYATHHDKFTDGVCLFWNCNVDRYPANAIVCRFDTRVLSNLNLPGCDGGSLYVN 6330
TR|A0A0U1WHI4|A0A0U1WHI4_CVHSA IEELFYSYATHHDKFTDGVCLFWNCNVDRYPANAIVCRFDTRVLSNLNLPGCDGGSLYVN 6319
TR|A0A0U1WHG0|A0A0U1WHG0_CVHSA IEELFYSYATHHDKFTDGVCLFWNCNVDRYPANAIVCRFDTRVLSNLNLPGCDGGSLYVN 6319
TR|A0A166ZL34|A0A166ZL34_9NIDO IEELFYSYATHHDKFTDGVCLFWNCNVDRYPANAIVCRFDTRVLSNLNLPGCDGGSLYVN 6126
TR|R9QTB2|R9QTB2_CVHSA         ------------------------------------------------------------
TR|R9QTH2|R9QTH2_CVHSA         ------------------------------------------------------------
SP|P0C6U8|R1A_CVHSA            ------------------------------------------------------------
TR|Q6JH47|Q6JH47_CVHSA         ------------------------------------------------------------
TR|Q692E5|Q692E5_CVHSA         ------------------------------------------------------------
SP|P0C6F8|R1A_BCHK3            ------------------------------------------------------------
TR|A0A0K1Z0N1|A0A0K1Z0N1_CVHSA ------------------------------------------------------------
SP|P0C6F5|R1A_BC279            ------------------------------------------------------------
SP|P0C6T7|R1A_BCRP3            ------------------------------------------------------------
```

```
QHN73794                       KHAFHTPAFDKSAFVNLKQLPFFYYSDSPCESHGKQVVSDIDYVPLKSATCITRCNLGGA 6407
SP|P0C6X7|R1AB_CVHSA           KHAFHTPAFDKSAFTNLKQLPFFYYSDSPCESHGKQVVSDIDYVPLKSATCITRCNLGGA 6384
TR|Q6UZF5|Q6UZF5_CVHSA         KHAFHTPAFDKSAFTNLKQLPFFYYSDSPCESHGKQVVSDIDYVPLKSATCITRCNLGGA 6384
TR|Q6UZF1|Q6UZF1_CVHSA         KHAFHTPAFDKSAFTNLKQLPFFYYSDSPCESHGKQVVSDIDYVPLKSATCITRCNLGGA 6384
TR|Q6JH48|Q6JH48_CVHSA         KHAFHTPAFDKSAFTNLKQLPFFYYSDSPCESHGKQVVSDIDYVPLKSATCITRCNLGGA 6384
TR|Q692E6|Q692E6_CVHSA         KHAFHTPAFDKSAFTNLKQLPFFYYSDSPCESHGKQVVSDIDYVPLKSATCITRCNLGGA 6384
TR|A0A0K1YZY7|A0A0K1YZY7_CVHSA KHAFHTPAFDKSAFTNLKQLPFFYYSDSPCESHGKQVVSDIDYVPLKSATCITRCNLGGA 6384
SP|P0C6W2|R1AB_BCHK3           KHAFHTPAFDKSAFTHLKQLPFFYYSDSPCESHGKQVVSDIDYVPLKSATCITRCNLGGA 6378
SP|P0C6W6|R1AB_BCRP3           KHAFHTPAFDKSAFTNLKQLPFFYYSDSPCESHGKQVVSDIDYVPLKSATCITRCNLGGA 6382
SP|P0C6V9|R1AB_BC279           KHAFHTPAFDKSAFTYLKQLPFFYYSDSPCESHGKQVVSDIDYVPLKSATCITRCNLGGA 6390
TR|A0A0U1WHI4|A0A0U1WHI4_CVHSA KHAFHTPAFDKSAFSNLKQLPFFYYSDSPCESHGKQVVSDIDYVPLKSATCITRCNLGGA 6379
TR|A0A0U1WHG0|A0A0U1WHG0_CVHSA KHAFHTPAFDKSAFTHLKQLPFFYYSDSPCESHGKQVVSDIDYVPLKSATCITRCNLGGA 6379
TR|A0A166ZL34|A0A166ZL34_9NIDO KHAFYTPAFDKSAFTHLKQLPFFYYSDSPCESHGKQVVSDIDYVPLKSATCITRCNLGGA 6186
TR|R9QTB2|R9QTB2_CVHSA         ------------------------------------------------------------
TR|R9QTH2|R9QTH2_CVHSA         ------------------------------------------------------------
SP|P0C6U8|R1A_CVHSA            ------------------------------------------------------------
TR|Q6JH47|Q6JH47_CVHSA         ------------------------------------------------------------
TR|Q692E5|Q692E5_CVHSA         ------------------------------------------------------------
SP|P0C6F8|R1A_BCHK3            ------------------------------------------------------------
TR|A0A0K1Z0N1|A0A0K1Z0N1_CVHSA ------------------------------------------------------------
SP|P0C6F5|R1A_BC279            ------------------------------------------------------------
SP|P0C6T7|R1A_BCRP3            ------------------------------------------------------------

QHN73794                       VCRHHANEYRLYLDAYNMMISAGFSLWVYKQFDTYNLWNTFTRLQSLENVAFNVVNKGHF 6467
SP|P0C6X7|R1AB_CVHSA           VCRHHANEYRQYLDAYNMMISAGFSLWIYKQFDTYNLWNTFTRLQSLENVAYNVVNKGHF 6444
TR|Q6UZF5|Q6UZF5_CVHSA         VCRHHANEYRQYLDAYNMMISAGFSLWIYKQFDTYNLWNTFTRLQSLENVAYNVVNKGHF 6444
TR|Q6UZF1|Q6UZF1_CVHSA         VCRHHANEYRQYLDAYNMMISAGFSLWIYKQFDTYNLWNTFTRLQSLENVAYNVVNKGHF 6444
TR|Q6JH48|Q6JH48_CVHSA         VCRHHANEYRQYLDAYNMMISAGFSLWIYKQFDTYNLWNTFTRLQSLENVAYNVVNKGHF 6444
TR|Q692E6|Q692E6_CVHSA         VCRHHANEYRQYLDAYNMMISAGFSLWIYKQFDTYNLWNTFTRLQSLENVAYNVVNKGHF 6444
TR|A0A0K1YZY7|A0A0K1YZY7_CVHSA VCRHHANEYRQYLDAYNMMISAGFSLWIYKQFDTYNLWNTFTRLQSLENVAYNVVNKGHF 6444
SP|P0C6W2|R1AB_BCHK3           VCRHHANEYRQYLDAYNMMISAGFSLWIYKQFDTYNLWNTFTKLQSLENVAYNVVNKGHF 6438
SP|P0C6W6|R1AB_BCRP3           VCRHHANEYRQYLDAYNMMISAGFSLWIYKQFDTYNLWNTFTRLQSLENVAYNVVNKGHF 6442
SP|P0C6V9|R1AB_BC279           VCRRHANEYRQYLDAYNMMISAGFSLWIYKQFDTYNLWNTFTRLQSLENVAYNVVNKGHF 6450
TR|A0A0U1WHI4|A0A0U1WHI4_CVHSA VCRHHANEYRQYLDAYNMMISAGFSLWIYKQFDTYNLWNTFTRLQSLENVAYNVINKGHF 6439
TR|A0A0U1WHG0|A0A0U1WHG0_CVHSA VCRHHANEYRQYLDAYNMMISAGFSLWIYKQFDTYNLWNTFTRLQSLENVAYNVINKGHF 6439
TR|A0A166ZL34|A0A166ZL34_9NIDO VCRHHANEYRQYLDAYNMMISAGFSLWIYKQFDTYNLWNTFTRLQSLENVAYNVINKGHF 6246
TR|R9QTB2|R9QTB2_CVHSA         ------------------------------------------------------------
TR|R9QTH2|R9QTH2_CVHSA         ------------------------------------------------------------
SP|P0C6U8|R1A_CVHSA            ------------------------------------------------------------
TR|Q6JH47|Q6JH47_CVHSA         ------------------------------------------------------------
TR|Q692E5|Q692E5_CVHSA         ------------------------------------------------------------
SP|P0C6F8|R1A_BCHK3            ------------------------------------------------------------
TR|A0A0K1Z0N1|A0A0K1Z0N1_CVHSA ------------------------------------------------------------
SP|P0C6F5|R1A_BC279            ------------------------------------------------------------
SP|P0C6T7|R1A_BCRP3            ------------------------------------------------------------

QHN73794                       DGQQGEVPVSIINNTVYTKVDGVDVELFENKTTLPVNVAFELWAKRNIKPVPEVKILNNL 6527
SP|P0C6X7|R1AB_CVHSA           DGHAGEAPVSIINNAVYTKVDGIDVEIFENKTTLPVNVAFELWAKRNIKPVPEIKILNNL 6504
TR|Q6UZF5|Q6UZF5_CVHSA         DGHAGEAPVSIINNAVYTKVDGIDVEIFENKTTLPVNVAFELWAKRNIKPVPEIKILNNL 6504
TR|Q6UZF1|Q6UZF1_CVHSA         DGHAGEAPVSIINNAVYTKVDGIDVEIFENKTTLPVNVAFELWAKRNIKPVPEIKILNNL 6504
TR|Q6JH48|Q6JH48_CVHSA         DGHAGEAPVSIINNAVYTKVDGIDVEIFENKTTLPVNVAFELWAKRNIKPVPEIKILNNL 6504
TR|Q692E6|Q692E6_CVHSA         DGHAGEAPVSIINNAVYTKVDGIDVEIFENKTTLPVNVAFELWAKRNIKPVPEIKILNNL 6504
TR|A0A0K1YZY7|A0A0K1YZY7_CVHSA DGQAGEAPVSIINNAVYTKVDGVDVEIFENKTTLPVNVAFELWAKRNIKPVPEIKILNNL 6504
SP|P0C6W2|R1AB_BCHK3           DGQSGEAPVSIINNAVYTKVDGIDVEIFENKTTLPVNVAFELWAKRNIKPVPEIKILNNL 6498
SP|P0C6W6|R1AB_BCRP3           DGQAGETPVSIINNAVYTKVDGFDVEIFENKTTLPVNVAFELWAKRNIKSVPEIKILNNL 6502
SP|P0C6V9|R1AB_BC279           DGQIGEAPVSIINNAVYTKVDGNDVEIFENKTTLPVNVAFELWAKRNIKPVPEIKILNNL 6510
TR|A0A0U1WHI4|A0A0U1WHI4_CVHSA DGQIGEVPVSIINNAVYTKVDGVDVEIFENKTILPVNVAFELWAKRNIKPVPEIKILNNL 6499
TR|A0A0U1WHG0|A0A0U1WHG0_CVHSA DGQIGEAPVSIINNAVYTKVDGVDVEIFENKTTLPVNVAFELWAKRNIKPVPEIKILNNL 6499
TR|A0A166ZL34|A0A166ZL34_9NIDO DGQIGEAPVSIINNAVYTKVDGVDVEIFENKTTLPVNVAFELWAKRNIKPVPEIKILNNL 6306
TR|R9QTB2|R9QTB2_CVHSA         ------------------------------------------------------------
TR|R9QTH2|R9QTH2_CVHSA         ------------------------------------------------------------
SP|P0C6U8|R1A_CVHSA            ------------------------------------------------------------
TR|Q6JH47|Q6JH47_CVHSA         ------------------------------------------------------------
TR|Q692E5|Q692E5_CVHSA         ------------------------------------------------------------
SP|P0C6F8|R1A_BCHK3            ------------------------------------------------------------
TR|A0A0K1Z0N1|A0A0K1Z0N1_CVHSA ------------------------------------------------------------
SP|P0C6F5|R1A_BC279            ------------------------------------------------------------
SP|P0C6T7|R1A_BCRP3            ------------------------------------------------------------
```

```
QHN73794                           GVDIAANTVIWDYKRDAPAHISTIGVCSMTDIAKKPTETICAPLTVFFDGRVDGQVDLFR 6587
SP|P0C6X7|R1AB_CVHSA               GVDIAANTVIWDYKREAPAHVSTIGVCTMTDIAKKPTESACSSLTVLFDGRVEGQVDLFR 6564
TR|Q6UZF5|Q6UZF5_CVHSA             GVDIAANTVIWDYKREAPAHVSTIGVCTMTDIAKKPTESACSSLTVLFDGRVEGQVDLFR 6564
TR|Q6UZF1|Q6UZF1_CVHSA             GVDIAANTVIWDYKREAPAHVSTIGVCTMTDIAKKPTESACSSLTVLFDGRVEGQVDLFR 6564
TR|Q6JH48|Q6JH48_CVHSA             GVDIAANTVIWDYKREAPAHVSTIGVCTMTDIAKKPTESACSSLTVLFDGRVEGQVDLFR 6564
TR|Q692E6|Q692E6_CVHSA             GVDIAANTVIWDYKREAPAHVSTIGVCTMTDIAKKPTESACSSLTVLFDGRVEGQVDLFR 6564
TR|A0A0K1YZY7|A0A0K1YZY7_CVHSA GVDIAANTVIWDYKREAPAHMSTIGVSTMTDIAKKPTESACSSLTVLFDGRVEGQVDLFR 6564
SP|P0C6W2|R1AB_BCHK3               GVDIAANNVIWDYKREAPAHVSTIGVCTMTDIAKKPTESACSSLIVLFDGRVEGQVDFFR 6558
SP|P0C6W6|R1AB_BCRP3               GVDIAANTVIWDHKREAPVHMSTIGVCTMTDIAKKPTESACSSLTVLFDGRVEGQVDLFR 6562
SP|P0C6V9|R1AB_BC279               GVDIAANTVIWDYKREAPAHVSTIGVCTMTDIAKKPTESACSSLTVLFDGRVEGQVDLFR 6570
TR|A0A0U1WHI4|A0A0U1WHI4_CVHSA GVDIAANTVIWDYKREAPAHVSTIGICTMTDIAKKPTESACSSLTVLFDGRVEGQVDLFR 6559
TR|A0A0U1WHG0|A0A0U1WHG0_CVHSA GVDIAANTVIWDYKREAPAHVSTIGICTMTDIAKKPTESTCSSLTVLFDGRVEGQVDLFR 6559
TR|A0A166ZL34|A0A166ZL34_9NIDO GVDIAANTVIWDYKREAPAHVSTIGICTMTDIAKKPTESTCSSLTVLFDGRVEGQVDLFR 6366
TR|R9QTB2|R9QTB2_CVHSA             ------------------------------------------------------------
TR|R9QTH2|R9QTH2_CVHSA             ------------------------------------------------------------
SP|P0C6U8|R1A_CVHSA                ------------------------------------------------------------
TR|Q6JH47|Q6JH47_CVHSA             ------------------------------------------------------------
TR|Q692E5|Q692E5_CVHSA             ------------------------------------------------------------
SP|P0C6F8|R1A_BCHK3                ------------------------------------------------------------
TR|A0A0K1Z0N1|A0A0K1Z0N1_CVHSA ------------------------------------------------------------
SP|P0C6F5|R1A_BC279                ------------------------------------------------------------
SP|P0C6T7|R1A_BCRP3                ------------------------------------------------------------

QHN73794                           NARNGVLITEGSVKGLQPSVGPKQASLNGVTLIGEAVKTQFNYYKKVDGVVQQLPETYFT 6647
SP|P0C6X7|R1AB_CVHSA               NARNGVLITEGSVKGLTPSKGPAQASVNGVTLIGESVKTQFNYFKKVDGIIQQLPETYFT 6624
TR|Q6UZF5|Q6UZF5_CVHSA             NARNGVLITEGSVKGLTPSKGPAQASVNGVTLIGESVKTQFNYFKKVDGIIQQLPETYFT 6624
TR|Q6UZF1|Q6UZF1_CVHSA             NARNGVLITEGSVKGLTPSKGPAQASVNGVTLIGESVKTQFNYFKKVDGIIQQLPETYFT 6624
TR|Q6JH48|Q6JH48_CVHSA             NARNGVLITEGSVKGLTPSKGPAQASVNGVTLIGESVKTQFNYFKKVDGIIQQLPETYFT 6624
TR|Q692E6|Q692E6_CVHSA             NARNGVLITEGSVKGLTPSKGPAQASVNGVTLIGESVKTQFNYFKKVDGIIQQLPETYFT 6624
TR|A0A0K1YZY7|A0A0K1YZY7_CVHSA NARNGVLITEGSVKGLTPSKGPAQASVNGVTLIGESVKTQFNYFKKVDGIIQQLPETYFT 6624
SP|P0C6W2|R1AB_BCHK3               NARNGVLITEGSVKGLTPSKGPAQASVNGVTLIGESVKTQFNYFKKVDGIIQQLPETYFT 6618
SP|P0C6W6|R1AB_BCRP3               NARNGVLITEGSVKGLTPSKGPAQASVNGVTLIGESVKTQFNYFKKVDGIIQQLPETYFT 6622
SP|P0C6V9|R1AB_BC279               NARNGVLITEGSVKGLTPSKGPAQASVNGVTLIGESVKTQFNYFKKVDGIIQQLPETYFT 6630
TR|A0A0U1WHI4|A0A0U1WHI4_CVHSA NARNGVLITEGSVKGLTPSKGPVQASVNGVTLIGESVKTQFNYFKKVDGIIQQLPETYFT 6619
TR|A0A0U1WHG0|A0A0U1WHG0_CVHSA NARNGVLITEGSVKGLTPSKGPVQASVNGVTLIGESVKTQFNYFKKVDGIIQQLPETYFT 6619
TR|A0A166ZL34|A0A166ZL34_9NIDO NARNGVLITEGSVKGLTPSKGPVQASVNGVTLIGESVKTQFNYFKKVDGIIQQLPETYFT 6426
TR|R9QTB2|R9QTB2_CVHSA             ------------------------------------------------------------
TR|R9QTH2|R9QTH2_CVHSA             ------------------------------------------------------------
SP|P0C6U8|R1A_CVHSA                ------------------------------------------------------------
TR|Q6JH47|Q6JH47_CVHSA             ------------------------------------------------------------
TR|Q692E5|Q692E5_CVHSA             ------------------------------------------------------------
SP|P0C6F8|R1A_BCHK3                ------------------------------------------------------------
TR|A0A0K1Z0N1|A0A0K1Z0N1_CVHSA ------------------------------------------------------------
SP|P0C6F5|R1A_BC279                ------------------------------------------------------------
SP|P0C6T7|R1A_BCRP3                ------------------------------------------------------------

QHN73794                           QSRNLQEFKPRSQMEIDFLELAMDEFIERYKLEGYAFEHIVYGDFSHSQLGGLHLLIGLA 6707
SP|P0C6X7|R1AB_CVHSA               QSRDLEDFKPRSQMETDFLELAMDEFIQRYKLEGYAFEHIVYGDFSHGQLGGLHLMIGLA 6684
TR|Q6UZF5|Q6UZF5_CVHSA             QSRDLEDFKPRSQMETDFLELAMDEFIQRYKLEGYAFEHIVYGDFSHGQLGGLHLMIGLA 6684
TR|Q6UZF1|Q6UZF1_CVHSA             QSRDLEDFKPRSQMETDFLELAMDEFIQRYKLEGYAFEHIVYGDFSHGQLGGLHLMIGLA 6684
TR|Q6JH48|Q6JH48_CVHSA             QSRDLEDFKPRSQMETDFLELAMDEFIQRYKLEGYAFEHIVYGDFSHGQLGGLHLMIGLA 6684
TR|Q692E6|Q692E6_CVHSA             QSRDLEDFKPRSQMETDFLELAMDEFIQRYKLEGYAFEHIVYGDFSHGQLGGLHLMIGLA 6684
TR|A0A0K1YZY7|A0A0K1YZY7_CVHSA QSRDLEDFKPRSQMETDFLELVMDEFIQRYKLEGYAFEHIVYGDFSHGQLGGLHLMIGLA 6684
SP|P0C6W2|R1AB_BCHK3               QSRDLEDFKPRSQMETDFLELAMDEFIQRYKLEGYAFEHIVYGDFSHGQLGGLHLMIGLA 6678
SP|P0C6W6|R1AB_BCRP3               QSRDLEDFKPRSQMETDFLELAMDEFIQRYKLEGYAFEHIVYGDFSHGQLGGLHLMIGLA 6682
SP|P0C6V9|R1AB_BC279               QSRDLEDFKPRSKMETDFLELAMDEFIQRYKLEGYAFEHIVYGDFSHGQLGGLHLMIGLA 6690
TR|A0A0U1WHI4|A0A0U1WHI4_CVHSA QSRDLEDFKPRSQMETDFLELAMDEFIQRYKLEGYAFEHIVYGDFSHGQLGGLHLMIGLA 6679
TR|A0A0U1WHG0|A0A0U1WHG0_CVHSA QSRDLEDFKPRSQMETDFLELAMDEFIQRYKLEGYAFDHIVYGDFSHGQLGGLHLMIGLA 6679
TR|A0A166ZL34|A0A166ZL34_9NIDO QSRDLEDFKPRSQMETDFLELAMDEFIQRYKLEGYAFDHIVYGDFSHGQLGGLHLMIGLA 6486
TR|R9QTB2|R9QTB2_CVHSA             ------------------------------------------------------------
TR|R9QTH2|R9QTH2_CVHSA             ------------------------------------------------------------
SP|P0C6U8|R1A_CVHSA                ------------------------------------------------------------
TR|Q6JH47|Q6JH47_CVHSA             ------------------------------------------------------------
TR|Q692E5|Q692E5_CVHSA             ------------------------------------------------------------
SP|P0C6F8|R1A_BCHK3                ------------------------------------------------------------
TR|A0A0K1Z0N1|A0A0K1Z0N1_CVHSA ------------------------------------------------------------
SP|P0C6F5|R1A_BC279                ------------------------------------------------------------
SP|P0C6T7|R1A_BCRP3                ------------------------------------------------------------
```

```
QHN73794                          KRFKESPFELEDFIPMDSTVKNYFITDAQTGSSKCVCSVIDLLLDDFVEIIKSQDLSVVS 6767
SP|P0C6X7|R1AB_CVHSA              KRSQDSPLKLEDFIPMDSTVKNYFITDAQTGSSKCVCSVIDLLLDDFVEIIKSQDLSVIS 6744
TR|Q6UZF5|Q6UZF5_CVHSA            KRSQDSPLKLEDFIPMDSTVKNYFITDAQTGSSKCVCSVIDLLLDDFVEIIKSQDLSVIS 6744
TR|Q6UZF1|Q6UZF1_CVHSA            KRSQDSPLKLEDFIPMDSTVKNYFITDAQTGSSKCVCSVIDLLLDDFVEIIKSQDLSVIS 6744
TR|Q6JH48|Q6JH48_CVHSA            KRSQDSPLKLEDFIPMDSTVKNYFITDAQTGSSKCVCSVIDLLLDDFVEIIKSQDLSVIS 6744
TR|Q692E6|Q692E6_CVHSA            KRSQDSPLKLEDFIPMDSTVKNYFITDAQTGSSKCVCSVIDLLLDDFVEIIKSQDLSVIS 6744
TR|A0A0K1YZY7|A0A0K1YZY7_CVHSA    KRSQDSPLKLEDFIPMDSTVKNYFITDAQTGSSKCVCSVIDLLLDDFVEIIKSQDLSVIS 6744
SP|P0C6W2|R1AB_BCHK3              KRSQDSLLKLEDFIPMDSTVKNYFITDAQTGSSKCVCSVIDLLLDDFVEIIKSQDLSVVS 6738
SP|P0C6W6|R1AB_BCRP3              KRSRDSPLKLEDFIPMDSTVKNYFITDAQTGSSKCVCSVIDLLLDDFVEIIKSQDLSVVS 6742
SP|P0C6V9|R1AB_BC279              KRSQDSPLKLEDFIPTDSTVKNYFITDAQTGSSKCVCSVIDLLLDDFVEIIKSQDLSVIS 6750
TR|A0A0U1WHI4|A0A0U1WHI4_CVHSA    KRSQDSPLKLEDFIPMDSTVKNYFITDAQTGSSKCVCSVIDLLLDDFVEIIKSQDLSVVS 6739
TR|A0A0U1WHG0|A0A0U1WHG0_CVHSA    KRSQDSPLKLEDFIPMDSTVKNYFITDAKTGSSKCVCSVIDLLLDDFVEIIKSQDLSVIS 6739
TR|A0A166ZL34|A0A166ZL34_9NIDO    KRSQDSPLKLEDFIPMDSTVKNYFITDAQTGSSKCVCSVIDLLLDDFVEIIKSQDLSVIS 6546
TR|R9QTB2|R9QTB2_CVHSA            ------------------------------------------------------------
TR|R9QTH2|R9QTH2_CVHSA            ------------------------------------------------------------
SP|P0C6U8|R1A_CVHSA               ------------------------------------------------------------
TR|Q6JH47|Q6JH47_CVHSA            ------------------------------------------------------------
TR|Q692E5|Q692E5_CVHSA            ------------------------------------------------------------
SP|P0C6F8|R1A_BCHK3               ------------------------------------------------------------
TR|A0A0K1Z0N1|A0A0K1Z0N1_CVHSA    ------------------------------------------------------------
SP|P0C6F5|R1A_BC279               ------------------------------------------------------------
SP|P0C6T7|R1A_BCRP3               ------------------------------------------------------------

QHN73794                          KVVKVTIDYTEISFMLWCKDGHVETFYPKLQSSQAWQPGVAMPNLYKMQRMLLEKCDLQN 6827
SP|P0C6X7|R1AB_CVHSA              KVVKVTIDYAEISFMLWCKDGHVETFYPKLQASQAWQPGVAMPNLYKMQRMLLEKCDLQN 6804
TR|Q6UZF5|Q6UZF5_CVHSA            KVVKVTIDYAEISFMLWCKDGHVETFYPKLQASQAWQPGVAMPNLYKMQRMLLEKCDLQN 6804
TR|Q6UZF1|Q6UZF1_CVHSA            KVVKVTIDYAEISFMLWCKDGHVETFYPKLQASQAWQPGVAMPNLYKMQRMLLEKCDLQN 6804
TR|Q6JH48|Q6JH48_CVHSA            KVVKVTIDYAEISFMLWCKDGHVETFYPKLQASQAWQPGVAMPNLYKMQRMLLEKCDLQN 6804
TR|Q692E6|Q692E6_CVHSA            KVVKVTIDYAEISFMLWCKDGHVETFYPKLQASQAWQPGVAMPNLYKMQRMLLEKCDLQN 6804
TR|A0A0K1YZY7|A0A0K1YZY7_CVHSA    KVVKVTIDYVEISFMLWCKDGHVETFYPKLQASQAWQPGVAMPNLYKMQRMLLEKCDLQN 6804
SP|P0C6W2|R1AB_BCHK3              KVVKVTIDYAEISFMLWCKDGHVETFYPKLQASQAWQPGVAMPNLYKMQRMLLEKCDLQN 6798
SP|P0C6W6|R1AB_BCRP3              KVVKVTIDYAEISFMLWCKDGHVETFYPKLQASQAWQPGVAMPNLYKMQRMLLEKCDLQN 6802
SP|P0C6V9|R1AB_BC279              KVVKVTIDYAEISFMLWCKDGHVETFYPKLQASQAWQPGVAMPNLYKMQRMLLEKCDLQN 6810
TR|A0A0U1WHI4|A0A0U1WHI4_CVHSA    KVVKVTIDYAEISFMLWCKDGHVETFYPKLQASQAWQPGVAMPNLYKMQRMLLEKCDLQN 6799
TR|A0A0U1WHG0|A0A0U1WHG0_CVHSA    KVVKVTIDYAEISFMLWCKDGYVETFYPKLQASQAWQPGVAMPNLYKMQRMLLEKCDLQN 6799
TR|A0A166ZL34|A0A166ZL34_9NIDO    KVVKVTIDYAEISFMLWCKDGYVETFYPKLQASQAWQPGVAMPNLYKMQRMLLEKCDLQN 6606
TR|R9QTB2|R9QTB2_CVHSA            ------------------------------------------------------------
TR|R9QTH2|R9QTH2_CVHSA            ------------------------------------------------------------
SP|P0C6U8|R1A_CVHSA               ------------------------------------------------------------
TR|Q6JH47|Q6JH47_CVHSA            ------------------------------------------------------------
TR|Q692E5|Q692E5_CVHSA            ------------------------------------------------------------
SP|P0C6F8|R1A_BCHK3               ------------------------------------------------------------
TR|A0A0K1Z0N1|A0A0K1Z0N1_CVHSA    ------------------------------------------------------------
SP|P0C6F5|R1A_BC279               ------------------------------------------------------------
SP|P0C6T7|R1A_BCRP3               ------------------------------------------------------------

QHN73794                          YGDSATLPKGIMMNVAKYTQLCQYLNTLTLAVPYNMRVIHFGAGSDKGVAPGTAVLRQWL 6887
SP|P0C6X7|R1AB_CVHSA              YGENAVIPKGIMMNVAKYTQLCQYLNTLTLAVPYNMRVIHFGAGSDKGVAPGTAVLRQWL 6864
TR|Q6UZF5|Q6UZF5_CVHSA            YGENAVIPKGIMMNVAKYTQLCQYLNTLTLAVPYNMRVIHFGAGSDKGVAPGTAVLRQWL 6864
TR|Q6UZF1|Q6UZF1_CVHSA            YGENAVIPKGIMMNVAKYTQLCQYLNTLTLAVPYNMRVIHFGAGSDKGVAPGTAVLRQWL 6864
TR|Q6JH48|Q6JH48_CVHSA            YGENAVIPKGIMMNVAKYTQLCQYLNTLTLAVPYNMRVIHFGAGSDKGVAPGTAVLRQWL 6864
TR|Q692E6|Q692E6_CVHSA            YGENAVIPKGIMMNVAKYTQLCQYLNTLTLAVPYNMRVIHFGAGSDKGVAPGTAVLRQWL 6864
TR|A0A0K1YZY7|A0A0K1YZY7_CVHSA    YGENAVIPKGIMMNVAKYTQLCQYLNTLTLAVPYNMRVIHFGAGSDKGVAPGTAVLRQWL 6864
SP|P0C6W2|R1AB_BCHK3              YGENAVIPKGIMMNVAKYTQLCQYLNTLTLAVPYNMRVIHFGAGSDKGVAPGTAVLRQWL 6858
SP|P0C6W6|R1AB_BCRP3              YGENAVIPKGIMMNVAKYTQLCQYLNTLTLAVPYNMRVIHFGAGSDKGVAPGTAVLRQWL 6862
SP|P0C6V9|R1AB_BC279              YGENAVIPKGIMMNVAKYTQLCQYLNTLTLAVPYNMRVIHFGAGSDKGVAPGTAVLRQWL 6870
TR|A0A0U1WHI4|A0A0U1WHI4_CVHSA    YGENAVIPKGIMMNVAKYTQLCQYLNTLTLAVPYNMRVIHFGAGSDKGVAPGTAVLRQWL 6859
TR|A0A0U1WHG0|A0A0U1WHG0_CVHSA    YGENAVIPKGIMMNVAKYTQLCQYLNTLTLAVPYNMRVIHFGAGSDKGVAPGTAVLRQWL 6859
TR|A0A166ZL34|A0A166ZL34_9NIDO    YGENAVIPKGIMMNVAKYTQLCQYLNTLTLAVPYNMRVIHFGAGSDKGVAPGTAVLRQWL 6666
TR|R9QTB2|R9QTB2_CVHSA            ------------------------------------------------------------
TR|R9QTH2|R9QTH2_CVHSA            ------------------------------------------------------------
SP|P0C6U8|R1A_CVHSA               ------------------------------------------------------------
TR|Q6JH47|Q6JH47_CVHSA            ------------------------------------------------------------
TR|Q692E5|Q692E5_CVHSA            ------------------------------------------------------------
SP|P0C6F8|R1A_BCHK3               ------------------------------------------------------------
TR|A0A0K1Z0N1|A0A0K1Z0N1_CVHSA    ------------------------------------------------------------
SP|P0C6F5|R1A_BC279               ------------------------------------------------------------
SP|P0C6T7|R1A_BCRP3               ------------------------------------------------------------
```

```
QHN73794                          PTGTLLVDSDLNDFVSDADSTLIGDCATVHTANKWDLIISDMYDPKTKNVTKENDSKEGF 6947
SP|P0C6X7|R1AB_CVHSA              PTGTLLVDSDLNDFVSDADSTLIGDCATVHTANKWDLIISDMYDPRTKHVTKENDSKEGF 6924
TR|Q6UZF5|Q6UZF5_CVHSA            PTGTLLVDSDLNDFVSDADSTLIGDCATVHTANKWDLIISDMYDPRTKHVTKENDSKEGF 6924
TR|Q6UZF1|Q6UZF1_CVHSA            PTGTLLVDSDLNDFVSDADSTLIGDCATVHTANKWDLIISDMYDPRTKHVTKENDSKEGF 6924
TR|Q6JH48|Q6JH48_CVHSA            PTGTLLVDSDLNDFVSDADSTLIGDCATVHTANKWDLIISDMYDPRTKHVTKENDSKEGF 6924
TR|Q692E6|Q692E6_CVHSA            PTGTLLVDSDLNDFVSDADSTLIGDCATVHTANKWDLIISDMYDPRTKHVTKENDSKEGF 6924
TR|A0A0K1YZY7|A0A0K1YZY7_CVHSA    PTGTLLVDSDLNDFVSDADSTLIGDCATVHTANKWDLIISDMYDPKTKHVTKENDSKEGF 6924
SP|P0C6W2|R1AB_BCHK3              PTGTLLVDSDLNDFVSDADSTLIGDCATVHTANKWDLIISDMYDPKTKHVLKDNDSKEGF 6918
SP|P0C6W6|R1AB_BCRP3              PTGTLLVDSDLNDFVSDADSTLIGDCATVHTANKWDLIVSDMYDPKAKHVTKENDSKEGF 6922
SP|P0C6V9|R1AB_BC279              PTGALLVDSDLNDFVSDADSTLIGDCATVHTANKWDLIISDMYDPKTKHVTKENDSKEGF 6930
TR|A0A0U1WHI4|A0A0U1WHI4_CVHSA    PIGTLLVDSDLNDFVSDADSTLIGDCATVHTANKWDLIVSDMYDPKTKHVTEENDSKEGF 6919
TR|A0A0U1WHG0|A0A0U1WHG0_CVHSA    PIGTLLVDSDLNDFVSDADSTLIGECATVHTANKWDLIVSDMYDPKTKHVTKENDSKEGF 6919
TR|A0A166ZL34|A0A166ZL34_9NIDO    PIGTLLVDSDLNDFVSDADSTLIGECATVHTANKWDLIVSDMYDPKTKHVTKENDSKEGF 6726
TR|R9QTB2|R9QTB2_CVHSA            ------------------------------------------------------------
TR|R9QTH2|R9QTH2_CVHSA            ------------------------------------------------------------
SP|P0C6U8|R1A_CVHSA               ------------------------------------------------------------
TR|Q6JH47|Q6JH47_CVHSA            ------------------------------------------------------------
TR|Q692E5|Q692E5_CVHSA            ------------------------------------------------------------
SP|P0C6F8|R1A_BCHK3               ------------------------------------------------------------
TR|A0A0K1Z0N1|A0A0K1Z0N1_CVHSA    ------------------------------------------------------------
SP|P0C6F5|R1A_BC279               ------------------------------------------------------------
SP|P0C6T7|R1A_BCRP3               ------------------------------------------------------------

QHN73794                          FTYICGFIQQKLALGGSVAIKITEHSWNADLYKLMGHFAWWTAFVTNVNASSSEAFLIGC 7007
SP|P0C6X7|R1AB_CVHSA              FTYLCGFIKQKLALGGSIAVKITEHSWNADLYKLMGHFSWWTAFVTNVNASSSEAFLIGA 6984
TR|Q6UZF5|Q6UZF5_CVHSA            FTYLCGFIKQKLALGGSIAVKITEHSWNADLYKLMGHFSWWTAFVTNVNASSSEAFLIGA 6984
TR|Q6UZF1|Q6UZF1_CVHSA            FTYLCGFIKQKLALGGSIAVKITEHSWNADLYKLMGHFSWWTAFVTNVNASSSEAFLIGA 6984
TR|Q6JH48|Q6JH48_CVHSA            FTYLCGFIKQKLALGGSIAVKITEHSWNADLYKLMGHFSWWTAFVTNVNASSSEAFLIGA 6984
TR|Q692E6|Q692E6_CVHSA            FTYLCGFIKQKLALGGSIAVKITEHSWNADLYKLMGHFSWWTAFVTNVNASSSEAFLIGA 6984
TR|A0A0K1YZY7|A0A0K1YZY7_CVHSA    FTYLCGFIKQKLALGGSAAVKITEHSWNADLYKLMGHFSWWTAFVTNVNASSSEAFLIGV 6984
SP|P0C6W2|R1AB_BCHK3              FTYLCGFIKQKLALGGSVAVKITEHSWNADLYKLMGHFSWWTAFVTNVNASSSEAFLIGV 6978
SP|P0C6W6|R1AB_BCRP3              FTYLCGFIKQKLALGGSVAVKITEHSWNADLYKLMGHFSWWTAFVTNVNASSSEAFLIGV 6982
SP|P0C6V9|R1AB_BC279              FTYLCGFIKQKLALGGSVAVKITEHSWNADLYKLMGHFSWWTAFVTNVNASSSEAFLIGV 6990
TR|A0A0U1WHI4|A0A0U1WHI4_CVHSA    FTYLCGFIKQKLALGGSVAVKITEHSWNADLYKLMGYFSWWTAFVTNVNASSSEAFLIGV 6979
TR|A0A0U1WHG0|A0A0U1WHG0_CVHSA    FTYLCGFIKQKLALGGSVAVKITEHSWNADLYKLMGHFSWWTAFVTNVNASSSEAFLIGV 6979
TR|A0A166ZL34|A0A166ZL34_9NIDO    FTYLCGFIKQKLALGGSVAVKITEHSWNADLYKLMGHFSWWTAFVTNVNASSSEAFLIGV 6786
TR|R9QTB2|R9QTB2_CVHSA            ------------------------------------------------------------
TR|R9QTH2|R9QTH2_CVHSA            ------------------------------------------------------------
SP|P0C6U8|R1A_CVHSA               ------------------------------------------------------------
TR|Q6JH47|Q6JH47_CVHSA            ------------------------------------------------------------
TR|Q692E5|Q692E5_CVHSA            ------------------------------------------------------------
SP|P0C6F8|R1A_BCHK3               ------------------------------------------------------------
TR|A0A0K1Z0N1|A0A0K1Z0N1_CVHSA    ------------------------------------------------------------
SP|P0C6F5|R1A_BC279               ------------------------------------------------------------
SP|P0C6T7|R1A_BCRP3               ------------------------------------------------------------

QHN73794                          NYLGKPREQIDGYVMHANYIFWRNTNPIQLSSYSLFDMSKFPLKLRGTAVMSLKEGQIND 7067
SP|P0C6X7|R1AB_CVHSA              NYLGKPKEQIDGYTMHANYIFWRNTNPIQLSSYSLFDMSKFPLKLRGTAVMSLKENQIND 7044
TR|Q6UZF5|Q6UZF5_CVHSA            NYLGKPKEQIDGYTMHANYIFWRNTNPIQLSSYSLFDMSKFPLKLRGTAVMSLKENQIND 7044
TR|Q6UZF1|Q6UZF1_CVHSA            NYLGKPKEQIDGYTMHANYIFWRNTNPIQLSSYSLFDMSKFPLKLRGTAVMSLKENQIND 7044
TR|Q6JH48|Q6JH48_CVHSA            NYLGKPKEQIDGYTMHANYIFWRNTNPIQLSSYSLFDMSKFPLKLRGTAVMSLKENQIND 7044
TR|Q692E6|Q692E6_CVHSA            NYLGKPKEQIDGYTMHANYIFWRNTNPIQLSSYSLFDMSKFPLKLRGTAVMSLKENQIND 7044
TR|A0A0K1YZY7|A0A0K1YZY7_CVHSA    NYLGKPKEQIDGYTMHANYIFWRNTNPIQLSSYSLFDMSKFPLKLRGTAVMFLKENQIND 7044
SP|P0C6W2|R1AB_BCHK3              NYLGKPKEQIDGYTMHANYIFWRNTNPIQLSSYSLFDMSKFPLKLRGTAVMSLKENQIND 7038
SP|P0C6W6|R1AB_BCRP3              NYLGKPKEQIDGYTMHANYIFWRNTNPIQLSSYSLFDMSKFPLKLRGTAVMSLKENQIND 7042
SP|P0C6V9|R1AB_BC279              NYLGKLREQIDGYTMHANYIFWRNTNPIQLSSYSLFDMSKFPLKLRGTAVMSLKENQIND 7050
TR|A0A0U1WHI4|A0A0U1WHI4_CVHSA    NYLGKPKEQIDGYTMHANYVFWRNTNPIQLSSYSLFDMSKFPLKLRGTAVMSLKENQIND 7039
TR|A0A0U1WHG0|A0A0U1WHG0_CVHSA    NYLGKPKEQIDGYTMHANYIFWRNTNPIQLSSYSLFDMSKFPLKLRGTAVMSLKENQIND 7039
TR|A0A166ZL34|A0A166ZL34_9NIDO    NYLGKPKEQIDGYTMHANYIFWRNTNPIQLSSYSLFDMSKFPLKLRGTAVMSLKENQIND 6846
TR|R9QTB2|R9QTB2_CVHSA            ------------------------------------------------------------
TR|R9QTH2|R9QTH2_CVHSA            ------------------------------------------------------------
SP|P0C6U8|R1A_CVHSA               ------------------------------------------------------------
TR|Q6JH47|Q6JH47_CVHSA            ------------------------------------------------------------
TR|Q692E5|Q692E5_CVHSA            ------------------------------------------------------------
SP|P0C6F8|R1A_BCHK3               ------------------------------------------------------------
TR|A0A0K1Z0N1|A0A0K1Z0N1_CVHSA    ------------------------------------------------------------
SP|P0C6F5|R1A_BC279               ------------------------------------------------------------
SP|P0C6T7|R1A_BCRP3               ------------------------------------------------------------
```
