# Supplementary material for: Structural Genomics of SARS-CoV-2 Indicates Evolutionary Conserved Functional Regions of Viral Proteins
Source: Viruses. 2020 Mar 25;12(4):360. doi: 10.3390/v12040360 (PMC7232164; doi:10.3390/v12040360)
Supplement: Supplementary file 1 [file viruses-12-00360-s001.zip › wNsp5-SARS_LBS1_mapped.pdf]

Supplementary File 4. Ligand binding sites extracted from SARS protein targets and mapped on the sequence of wNsp5 protein aligned with the closest homologs found in UniProt. Shown is the first group of ligands.

Ligand BS:

benzyl (2-oxopropyl)carbamate (3D62-959, SARS)

N-[(1R)-2-(tert-butylamino)-2-oxo-1-(pyridin-3-yl)ethyl]-N-(4-tert-butylphenyl)furan-2-carboxamide (3V3M-0EN, SARS)

N-[4-(acetylamino)phenyl]-2-(1H-benzotriazol-1-yl)-N-[(1R)-2-[(2-methylbutan-2-yl)amino]-1-(1-methyl-1H-pyrrol-2-yl)-2-oxoethyl]acetamide (4MDS-23H, SARS)

(2S)-2-({[(3S,4aR,8aS)-2-(4-bromobenzoyl)decahydroisoquinolin-3-yl]methyl}amino)-3-(1H-imidazol-5-yl)propanal (4TWW-3A7, SARS)

(2S)-2-({[(3S,4aR,8aS)-2-(biphenyl-4-ylcarbonyl)decahydroisoquinolin-3-yl]methyl}amino)-3-(1H-imidazol-5-yl)propanal (4TWY-3BL, SARS)

(2S)-2-({[(3R,4aS,8aR)-2-(biphenyl-4-ylcarbonyl)decahydroisoquinolin-3-yl]methyl}amino)-3-(1H-imidazol-5-yl)propanal (4WY3-3X5, SARS)

N-(3-FUROYL)-D-VALYL-L-VALYL-N~1~-((1R,2Z)-4-ETHOXY-4-OXO-1-{[(3S)-2-OXOPYRROLIDIN-3-YL]METHYL}BUT-2-ENYL)-D-LEUCINAMIDE (2AMD-9IN, SARS)

(5S,8S,14R)-ETHYL 11-(3-AMINO-3-OXOPROPYL)-8-BENZYL-14-HYDROXY-5-ISOBUTYL-3,6,9,12-TETRAOXO-1-PHENYL-2-OXA-4,7,10,11-TETRAAZAPENTADECAN-15-OATE (2A5K-AZP, SARS)

N-((3S,6R)-6-((S,E)-4-ETHOXYCARBONYL-1-((S)-2-OXOPYRROLIDIN-3-YL)BUT-3-EN-2-YLCARBAMOYL)-2,9-DIMETHYL-4-OXODEC-8-EN-3-YL)-5-METHYLISOXAZOLE-3-CARBOXAMIDE (2ALV-CY6, SARS)

ETHYL (4R)-4-{[(2R,5S)-5-{[N-(TERT-BUTOXYCARBONYL)-L-SERYL]AMINO}-6-METHYL-2-(3-METHYLBUT-2-EN-1-YL)-4-OXOHEPTANOYL]AMINO}-5-[(3R)-2-OXOPYRROLIDIN-3-YL]PENTANOATE (2QIQ-CYV, SARS)

ETHYL (2E,4S)-4-[((2R)-2-{[N-(TERT-BUTOXYCARBONYL)-L-VALYL]AMINO}-2-PHENYLETHANOYL)AMINO]-5-[(3S)-2-OXOPYRROLIDIN-3-YL]PENT-2-ENOATE (2D2D-ENB, SARS)

ETHYL (4R)-4-({N-[(BENZYLOXY)CARBONYL]-L-PHENYLALANYL}AMINO)-5-[(3S)-2-OXOPYRROLIDIN-3-YL]PENTANOATE (3SZN-G75, SARS)

ETHYL (4R)-4-{[N-(TERT-BUTOXYCARBONYL)-L-PHENYLALANYL]AMINO}-5-[(3S)-2-OXOPYRROLIDIN-3-YL]PENTANOATE (3TIT-G81, SARS)

```
QHN73794                          EAACCHLAKALNDFSNSGSDVLYQPPQTSITSAVLQSGFRKMAFPSGKVEGCMVQVTCGT 3287
SP|P0C6X7|R1AB_CVHSA              EAACCHLAKALNDFSNSGADVLYQPPQTSITSAVLQSGFRKMAFPSGKVEGCMVQVTCGT 3264
TR|Q6UZF5|Q6UZF5_CVHSA            EAACCHLAKALNDFSNSGADVLYQPPQTSITSAVLQSGFRKMAFPSGKVEGCMVQVTCGT 3264
TR|Q6UZF1|Q6UZF1_CVHSA            EAACCHLAKALNDFSNSGADVLYQPPQTSITSAVLQSGFRKMAFPSGKVEGCMVQVTCGT 3264
TR|Q6JH48|Q6JH48_CVHSA            EAACCHLAKALNDFSNSGADVLYQPPQTSITSAVLQSGFRKMAFPSGKVEGCMVQVTCGT 3264
TR|Q692E6|Q692E6_CVHSA            EAACCHLAKALNDFSNSGADVLYQPPQTSITSAVLQSGFRKMAFPSGKVEGCMVQVTCGT 3264
TR|A0A0K1YZY7|A0A0K1YZY7_CVHSA    EAACCHLAKALNDFSNSGSDVLYQPPQTSITSAVLQSGFRKMAFPSGKVEGCMVQVTCGT 3264
SP|P0C6W2|R1AB_BCHK3              EAACCHLAKALNDFSNSGADVLYQPPQTSITSAVLQSGFRKMAFPSGKVEGCMVQVTCGT 3258
SP|P0C6W6|R1AB_BCRP3              EAACCHLAKALNDFSNSGADVLYQPPQTSITSAVLQSGFRKMAFPSGKVEGCMVQVTCGT 3262
SP|P0C6V9|R1AB_BC279              EAACCHLAKALNDFSNSGADVLYQPPQTSITSAVLQSGFRKMAFPSGKVEGCMVQVTCGT 3270
TR|A0A0U1WHI4|A0A0U1WHI4_CVHSA    EAACCHLAKALNDFSNSGADVLYQPPQTSITSAVLQSGFRRMAFPSGKVEGCMVQVTCGT 3259
TR|A0A0U1WHG0|A0A0U1WHG0_CVHSA    EAACCHLAKALNDFSNSGADVLYQPPQTSITSAVLQSGFKKVAFPSGKVEGCMVQVTCGT 3259
TR|A0A166ZL34|A0A166ZL34_9NIDO    EAACCHLAKALNDFSNSGADVLYQPPQTSITSAVLQSGFKKMAFPSGKVEGCMVQVTCGT 3066
TR|R9QTB2|R9QTB2_CVHSA            EAACCHLAKALNDFSNSGADVLYQPPQTSITSAVLQSGFRKMAFPSGKVEGCMVQVTCGT 3256
TR|R9QTH2|R9QTH2_CVHSA            EAACCHLAKALNDFSNSGSDVLYQPPQTSITSAVLQSGFRKMAFPSGKVEGCMVQVTCGT 3265
SP|P0C6U8|R1A_CVHSA               EAACCHLAKALNDFSNSGADVLYQPPQTSITSAVLQSGFRKMAFPSGKVEGCMVQVTCGT 3264
TR|Q6JH47|Q6JH47_CVHSA            EAACCHLAKALNDFSNSGADVLYQPPQTSITSAVLQSGFRKMAFPSGKVEGCMVQVTCGT 3264
TR|Q692E5|Q692E5_CVHSA            EAACCHLAKALNDFSNSGADVLYQPPQTSITSAVLQSGFRKMAFPSGKVEGCMVQVTCGT 3264
SP|P0C6F8|R1A_BCHK3               EAACCHLAKALNDFSNSGADVLYQPPQTSITSAVLQSGFRKMAFPSGKVEGCMVQVTCGT 3258
TR|A0A0K1Z0N1|A0A0K1Z0N1_CVHSA    EAACCHLAKALNDFSNSGSDVLYQPPQTSITSAVLQSGFRKMAFPSGKVEGCMVQVTCGT 3264
SP|P0C6F5|R1A_BC279               EAACCHLAKALNDFSNSGADVLYQPPQTSITSAVLQSGFRKMAFPSGKVEGCMVQVTCGT 3270
SP|P0C6T7|R1A_BCRP3               EAACCHLAKALNDFSNSGADVLYQPPQTSITSAVLQSGFRKMAFPSGKVEGCMVQVTCGT 3262
                                  ******************:********************:::******************
```

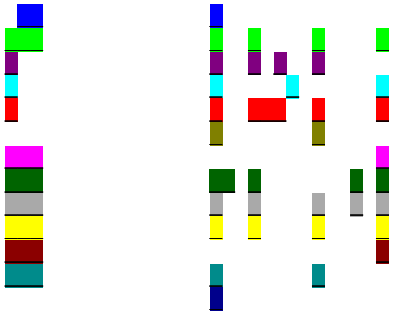

```
QHN73794                               TTLNGLWLDDVVYCPRHVICTSEDMLNPNYEDLLIRKSNHNFLVQAGNVQLRVIGHSMQN 3347
SP|P0C6X7|R1AB_CVHSA                   TTLNGLWLDDTVYCPRHVICTAEDMLNPNYEDLLIRKSNHSFLVQAGNVQLRVIGHSMQN 3324
TR|Q6UZF5|Q6UZF5_CVHSA                 TTLNGLWLDDTVYCPRHVICTAEDMLNPNYEDLLIRKSNHSFLVQAGNVQLRVIGHSMQN 3324
TR|Q6UZF1|Q6UZF1_CVHSA                 TTLNGLWLDDTVYCPRHVICTAEDMLNPNYEDLLIRKSNHSFLVQAGNVQLRVIGHSMQN 3324
TR|Q6JH48|Q6JH48_CVHSA                 TTLNGLWLDDTVYCPRHVICTAEDMLNPNYEDLLIRKANHSFLVQAGNVQLRVIGHSMQN 3324
TR|Q692E6|Q692E6_CVHSA                 TTLNGLWLDDTVYCPRHVICTAEDMLNPNYEDLLIRKSNHSFLVQAGNVQLRVIGHSMQN 3324
TR|A0A0K1YZY7|A0A0K1YZY7_CVHSA         TTLNGLWLDDTVYCPRHVICTAEDMLNPNYEDLLIRKSNHSFLVQAGNVQLRVIGHSMQN 3324
SP|P0C6W2|R1AB_BCHK3                   TTLNGLWLDDTVYCPRHVVCTAEDMLNPNYDDLLIRKSNHSFLVQAGNVQLRVIGHSMQN 3318
SP|P0C6W6|R1AB_BCRP3                   TTLNGLWLDDTVYCPRHVICTAEDMLNPNYEDLLIRKSNHSFLVQAGNVQLRVIGHSMQN 3322
SP|P0C6V9|R1AB_BC279                   TTLNGLWLDDTVYCPRHVICTAEDMLNPNYEDLLIRKSNHSFLVQAGNVQLRVIGHSMQN 3330
TR|A0A0U1WHI4|A0A0U1WHI4_CVHSA         TTLNGLWLDDTVYCPRHVICTAEDMLNPNYEDLLIRKSNHSFLVQAGNVQLRVIGHSMQN 3319
TR|A0A0U1WHG0|A0A0U1WHG0_CVHSA         TTLNGLWLDDTVYCPRHVVCTVEDMLNPNYEDLLIRKSNHSFLVQTGNVQLRVIGHSMQN 3319
TR|A0A166ZL34|A0A166ZL34_9NIDO         TTLNGLWLDDTVYCPRHVVCTVEDMLNPNYEDLLIRKSNHSFLVQTGNVQLRVIGHSMQN 3126
TR|R9QTB2|R9QTB2_CVHSA                 TTLNGLWLDDTVYCPRHVICTAEDMLNPNYEDLLIRKSNHSFLVQAGNVQLRVIGHSMQN 3316
TR|R9QTH2|R9QTH2_CVHSA                 TTLNGLWLDDTVYCPRHVICTAEDMLNPNYEDLLIRKSNHSFLVQAGNVQLRVIGHSMQN 3325
SP|P0C6U8|R1A_CVHSA                    TTLNGLWLDDTVYCPRHVICTAEDMLNPNYEDLLIRKSNHSFLVQAGNVQLRVIGHSMQN 3324
TR|Q6JH47|Q6JH47_CVHSA                 TTLNGLWLDDTVYCPRHVICTAEDMLNPNYEDLLIRKANHSFLVQAGNVQLRVIGHSMQN 3324
TR|Q692E5|Q692E5_CVHSA                 TTLNGLWLDDTVYCPRHVICTAEDMLNPNYEDLLIRKSNHSFLVQAGNVQLRVIGHSMQN 3324
SP|P0C6F8|R1A_BCHK3                    TTLNGLWLDDTVYCPRHVVCTAEDMLNPNYDDLLIRKSNHSFLVQAGNVQLRVIGHSMQN 3318
TR|A0A0K1Z0N1|A0A0K1Z0N1_CVHSA         TTLNGLWLDDTVYCPRHVICTAEDMLNPNYEDLLIRKSNHSFLVQAGNVQLRVIGHSMQN 3324
SP|P0C6F5|R1A_BC279                    TTLNGLWLDDTVYCPRHVICTAEDMLNPNYEDLLIRKSNHSFLVQAGNVQLRVIGHSMQN 3330
SP|P0C6T7|R1A_BCRP3                    TTLNGLWLDDTVYCPRHVICTAEDMLNPNYEDLLIRKSNHSFLVQAGNVQLRVIGHSMQN 3322
                                       **********.*******:** ********:******:**.****:**************
```

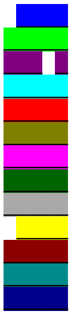

```
QHN73794                               CVLKLKVDTANPKTPKYKFVRIQPGQTFSVLACYNGSPSGVYQCAMRPNFTIKGSFLNGS 3407
SP|P0C6X7|R1AB_CVHSA                   CLLRLKVDTSNPKTPKYKFVRIQPGQTFSVLACYNGSPSGVYQCAMRPNHTIKGSFLNGS 3384
TR|Q6UZF5|Q6UZF5_CVHSA                 CLLRLKVDTSNPKTPKYKFVRIQPGQTFSVLACYNGSPSGVYQCAMRPNHTIKGSFLNGS 3384
TR|Q6UZF1|Q6UZF1_CVHSA                 CLLRLKVDTSNPKTPKYKFVRIQPGQTFSVLACYNGSPSGVYQCAMRPNHTIKGSFLNGS 3384
TR|Q6JH48|Q6JH48_CVHSA                 CLLRLKVDTSNPKTPKYKFVRIQPGQTFSVLACYNGSPSGVYQCAMRPNHTIKGSFLNGS 3384
TR|Q692E6|Q692E6_CVHSA                 CLLRLKVDTSNPKTPKYKFVRIQPGQTFSVLACYNGSPSGVYQCAMRPNHTIKGSFLNGS 3384
TR|A0A0K1YZY7|A0A0K1YZY7_CVHSA         CLLRLKVDTSNPKTPKYKFVRIQPGQTFSVLACYNGSPSGVYQCAMRPNHTIKGSFLNGS 3384
SP|P0C6W2|R1AB_BCHK3                   CLLRLKVDTSNPKTPKYKFVRIQPGQTFSVLACYNGSPSGVYQCAMRPNHTIKGSFLNGS 3378
SP|P0C6W6|R1AB_BCRP3                   CLLRLKVDTSNPKTPKYKFVRIQPGQTFSVLACYNGSPSGVYQCAMRPNHTIKGSFLNGS 3382
SP|P0C6V9|R1AB_BC279                   CLLRLKVDTSNPKTPKYKFVRIQPGQTFSVLACYNGSPSGVYQCAMRPNYTIKGSFLNGS 3390
TR|A0A0U1WHI4|A0A0U1WHI4_CVHSA         CLLRLKVDTSNPKTPKYKFVRIQPGQTFSVLACYNGSPSGVYQCAMRPNYTIKGSFLNGS 3379
TR|A0A0U1WHG0|A0A0U1WHG0_CVHSA         CLLRLKVDTSNPKTPKYKFVRIQPGQTFSVLACYNGSPSGVYQCAMRPNYTIKGSFLNGS 3379
TR|A0A166ZL34|A0A166ZL34_9NIDO         CLLRLKVDTSNPKTPKYKFVRIQPGQTFSVLACYNGSPSGVYQCAMRPNYTIKGSFLNGS 3186
TR|R9QTB2|R9QTB2_CVHSA                 CLLRLKVDTSNPKTPKYKFVRIQPGQTFSVLACYNGSPSGVYQCAMRPNHTIKGSFLNGS 3376
TR|R9QTH2|R9QTH2_CVHSA                 CLLRLKVDTSNPKTPKYKFVRIQPGQTFSVLACYNGSPSGVYQCAMRPNHTIKGSFLNGS 3385
SP|P0C6U8|R1A_CVHSA                    CLLRLKVDTSNPKTPKYKFVRIQPGQTFSVLACYNGSPSGVYQCAMRPNHTIKGSFLNGS 3384
TR|Q6JH47|Q6JH47_CVHSA                 CLLRLKVDTSNPKTPKYKFVRIQPGQTFSVLACYNGSPSGVYQCAMRPNHTIKGSFLNGS 3384
TR|Q692E5|Q692E5_CVHSA                 CLLRLKVDTSNPKTPKYKFVRIQPGQTFSVLACYNGSPSGVYQCAMRPNHTIKGSFLNGS 3384
SP|P0C6F8|R1A_BCHK3                    CLLRLKVDTSNPKTPKYKFVRIQPGQTFSVLACYNGSPSGVYQCAMRPNHTIKGSFLNGS 3378
TR|A0A0K1Z0N1|A0A0K1Z0N1_CVHSA         CLLRLKVDTSNPKTPKYKFVRIQPGQTFSVLACYNGSPSGVYQCAMRPNHTIKGSFLNGS 3384
SP|P0C6F5|R1A_BC279                    CLLRLKVDTSNPKTPKYKFVRIQPGQTFSVLACYNGSPSGVYQCAMRPNYTIKGSFLNGS 3390
SP|P0C6T7|R1A_BCRP3                    CLLRLKVDTSNPKTPKYKFVRIQPGQTFSVLACYNGSPSGVYQCAMRPNHTIKGSFLNGS 3382
                                       *:*:*****:***************************************.**********
```

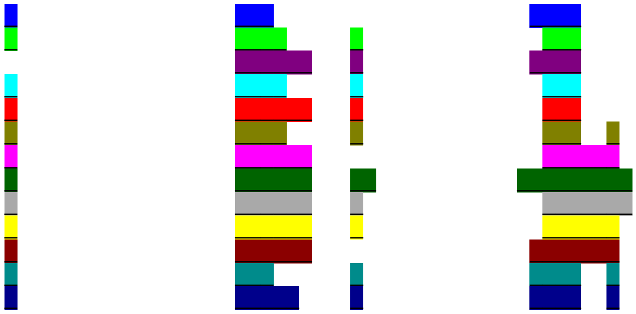

```
QHN73794                           CGSVGFNIDYDCVSFCYMHHMELPTGVHAGTDLEGNFYGPFVDRQTAQAAGTDTTITVNV 3467
SP|P0C6X7|R1AB_CVHSA               CGSVGFNIDYDCVSFCYMHHMELPTGVHAGTDLEGKFYGPFVDRQTAQAAGTDTTITLNV 3444
TR|Q6UZF5|Q6UZF5_CVHSA             CGSVGFNIDYDCVSFCYMHHMELPTGVHAGTDLEGKFYGPFVDRQTAQAAGTDTTITLNV 3444
TR|Q6UZF1|Q6UZF1_CVHSA             CGSVGFNIDYDCVSFCYMHHMELPTGVHAGTDLEGKFYGPFVDRQTAQAAGTDTTITLNV 3444
TR|Q6JH48|Q6JH48_CVHSA             CGSVGFNIDYDCVSFCYMHHMELPTGVHAGTDLEGKFYGPFVDRQTAQAAGTDTTITLNV 3444
TR|Q692E6|Q692E6_CVHSA             CGSVGFNIDYDCVSFCYMHHMELPTGVHAGTDLEGKFYGPFVDRQTAQAAGTDTTITLNV 3444
TR|A0A0K1YZY7|A0A0K1YZY7_CVHSA     CGSVGFNIDYDCVSFCYMHHMELPTGVHAGTDLEGKFYGPFVDRQTAQAAGTDTTITLNV 3444
SP|P0C6W2|R1AB_BCHK3               CGSVGFNIDYDCVSFCYMHHMELPTGVHAGTDLEGKFYGPFVDRQTAQAAGTDTTITLNV 3438
SP|P0C6W6|R1AB_BCRP3               CGSVGFNIDYDCVSFCYMHHMELPTEVHAGTDLEGKFYGPFVDRQTAQAAGTDTTITLNV 3442
SP|P0C6V9|R1AB_BC279               CGSVGFNIDYDCVSFCYMHHMELPTGVHAGTDLEGKFYGPFVDRQTAQAAGTDTTITLNV 3450
TR|A0A0U1WHI4|A0A0U1WHI4_CVHSA     CGSVGFNIDYDCVSFCYMHHMELPTGVHAGTDLEGKFYGPFVDRQTAQAAGTDTTITLNV 3439
TR|A0A0U1WHG0|A0A0U1WHG0_CVHSA     CGSVGFNIDYDCVSFCYMHHMELPTGVHAGTDLEGKFYGPFVDRQTAQAAGTDTTITLNV 3439
TR|A0A166ZL34|A0A166ZL34_9NIDO     CGSVGFNIDYDCVSFCYMHHMELPTGVHAGTDLEGKFYGPFVDRQTAQAAGTDTAITLNV 3246
TR|R9QTB2|R9QTB2_CVHSA             CGSVGFNIDYDCVSFCYMHHMELPTGVHAGTDLEGKFYGPFVDRQTAQAAGTDTTITLNV 3436
TR|R9QTH2|R9QTH2_CVHSA             CGSVGFNIDYDCVSFCYMHHMELPTGVHAGTDLEGKFYGPFVDRQTAQAAGTDTTITLNV 3445
SP|P0C6U8|R1A_CVHSA                CGSVGFNIDYDCVSFCYMHHMELPTGVHAGTDLEGKFYGPFVDRQTAQAAGTDTTITLNV 3444
TR|Q6JH47|Q6JH47_CVHSA             CGSVGFNIDYDCVSFCYMHHMELPTGVHAGTDLEGKFYGPFVDRQTAQAAGTDTTITLNV 3444
TR|Q692E5|Q692E5_CVHSA             CGSVGFNIDYDCVSFCYMHHMELPTGVHAGTDLEGKFYGPFVDRQTAQAAGTDTTITLNV 3444
SP|P0C6F8|R1A_BCHK3                CGSVGFNIDYDCVSFCYMHHMELPTGVHAGTDLEGKFYGPFVDRQTAQAAGTDTTITLNV 3438
TR|A0A0K1Z0N1|A0A0K1Z0N1_CVHSA     CGSVGFNIDYDCVSFCYMHHMELPTGVHAGTDLEGKFYGPFVDRQTAQAAGTDTTITLNV 3444
SP|P0C6F5|R1A_BC279                CGSVGFNIDYDCVSFCYMHHMELPTGVHAGTDLEGKFYGPFVDRQTAQAAGTDTTITLNV 3450
SP|P0C6T7|R1A_BCRP3                CGSVGFNIDYDCVSFCYMHHMELPTEVHAGTDLEGKFYGPFVDRQTAQAAGTDTTITLNV 3442
                                   ************************* *********:*****************:**:**

QHN73794                           LAWLYAAVINGDRWFLNRFTTTLNDFNLVAMKYNYEPLTQDHVDILGPLSAQTGIAVLDM 3527
SP|P0C6X7|R1AB_CVHSA               LAWLYAAVINGDRWFLNRFTTTLNDFNLVAMKYNYEPLTQDHVDILGPLSAQTGIAVLDM 3504
TR|Q6UZF5|Q6UZF5_CVHSA             LAWLYAAVINGDRWFLNRFTTTLNDFNLVAMKYNYEPLTQDHVDILGPLSAQTGIAVLDM 3504
TR|Q6UZF1|Q6UZF1_CVHSA             LAWLYAAVINGDRWFLNRFTTTLNDFNLVAMKYNYEPLTQDHVDILGPLSAQTGIAVLDM 3504
TR|Q6JH48|Q6JH48_CVHSA             LAWLYAAVINGDRWFLNRFTTTLNDFNLVAMKYNYEPLTQDHVDILGPLSAQTGIAVLDM 3504
TR|Q692E6|Q692E6_CVHSA             LAWLYAAVINGDRWFLNRFTTTLNDFNLVAMKYNYEPLTQDHVDILGPLSAQTGIAVLDM 3504
TR|A0A0K1YZY7|A0A0K1YZY7_CVHSA     LAWLYAAVINGDRWFLNRFTTTLNDFNLVAMKYNYEPLTQDHVDVLGPLSAQTGIAVLDM 3504
SP|P0C6W2|R1AB_BCHK3               LAWLYAAVINGDRWFLNRFTTTLNDFNLVAMKYNYEPLTQDHVDILGPLSAQTGIAVLDM 3498
SP|P0C6W6|R1AB_BCRP3               LAWLYAAVINGDRWFLNRFTTTLNDFNLVAMKYNYEPLTQDHVDILGPLSAQTGIAVLDM 3502
SP|P0C6V9|R1AB_BC279               LAWLYAAVINGDRWFLNRFTTTLNDFNLVAMKYNYEPLTQDHVDILGPLSAQTGIAVLDM 3510
TR|A0A0U1WHI4|A0A0U1WHI4_CVHSA     LAWLYAAVINGDRWFLNRFTTTLNDFNLVAMKYNYEPLTQDHVDILGPLSAQTGIAVLDM 3499
TR|A0A0U1WHG0|A0A0U1WHG0_CVHSA     LAWLYAAVINGDRWFLNRFTTTLNDFNLVAMKYNYEPLTQDHVDILGPLSAQTGIAVLDM 3499
TR|A0A166ZL34|A0A166ZL34_9NIDO     LAWLYAAVINGDRWFLNRFTTTLNDFNLVAMKYNYEPLTQDHVDILGPLSAQTGIAVLDM 3306
TR|R9QTB2|R9QTB2_CVHSA             LAWLYAAVINGDRWFLNRFTTTLNDFNLVAMKYNYEPLTQDHVDILGPLSAQTGIAVLDM 3496
TR|R9QTH2|R9QTH2_CVHSA             LAWLYAAVINGDRWFLNRFTTTLNDFNLVAMKYNYEPLTQDHVDILGPLSAQTGIAVLDM 3505
SP|P0C6U8|R1A_CVHSA                LAWLYAAVINGDRWFLNRFTTTLNDFNLVAMKYNYEPLTQDHVDILGPLSAQTGIAVLDM 3504
TR|Q6JH47|Q6JH47_CVHSA             LAWLYAAVINGDRWFLNRFTTTLNDFNLVAMKYNYEPLTQDHVDILGPLSAQTGIAVLDM 3504
TR|Q692E5|Q692E5_CVHSA             LAWLYAAVINGDRWFLNRFTTTLNDFNLVAMKYNYEPLTQDHVDILGPLSAQTGIAVLDM 3504
SP|P0C6F8|R1A_BCHK3                LAWLYAAVINGDRWFLNRFTTTLNDFNLVAMKYNYEPLTQDHVDILGPLSAQTGIAVLDM 3498
TR|A0A0K1Z0N1|A0A0K1Z0N1_CVHSA     LAWLYAAVINGDRWFLNRFTTTLNDFNLVAMKYNYEPLTQDHVDVLGPLSAQTGIAVLDM 3504
SP|P0C6F5|R1A_BC279                LAWLYAAVINGDRWFLNRFTTTLNDFNLVAMKYNYEPLTQDHVDILGPLSAQTGIAVLDM 3510
SP|P0C6T7|R1A_BCRP3                LAWLYAAVINGDRWFLNRFTTTLNDFNLVAMKYNYEPLTQDHVDILGPLSAQTGIAVLDM 3502
                                   ********************************************:***************
```

```
QHN73794                           CASLKELLQNGMNGRTILGSALLEDEFTPFDVVRQCSGVTFQSAVKRTIKGTHHWLLLTI 3587
SP|P0C6X7|R1AB_CVHSA               CAALKELLQNGMNGRTILGSTILEDEFTPFDVVRQCSGVTFQGKFKKIVKGTHHWMLLTF 3564
TR|Q6UZF5|Q6UZF5_CVHSA             CAALKELLQNGMNGRTILGSTILEDEFTPFDVVRQCSGVTFQGKFKKIVKGTHHWMLLTF 3564
TR|Q6UZF1|Q6UZF1_CVHSA             CAALKELLQNGMNGRTILGSTILEDEFTPFDVVRQCSGVTFQGKFKKIVKGTHHWMLLTF 3564
TR|Q6JH48|Q6JH48_CVHSA             CAALKELLQNGMNGRTILGSTILEDEFTPFDVVRQCSGVTFQGKFKKIVKGTHHWMLLTF 3564
TR|Q692E6|Q692E6_CVHSA             CAALKELLQNGMNGRTILGSTILEDEFTPFDVVRQCSGVTFQGKFKKIVKGTHHWMLLTF 3564
TR|A0A0K1YZY7|A0A0K1YZY7_CVHSA CAALKELLQNGMNGRTILGSTILEDEFTPFDVVRQCSGVTFQGKFKKIVKGTHHWMLLTF 3564
SP|P0C6W2|R1AB_BCHK3               CAALKELLQNGMNGRTILGSTILEDEFTPFDVVRQCSGVTFQGKFKKIVKGTHHWMLLTF 3558
SP|P0C6W6|R1AB_BCRP3               CAALKELLQNGMNGRTILGSTILEDEFTPFDVVRQCSGVTFQGKFKRIVKGTHHWMLLTF 3562
SP|P0C6V9|R1AB_BC279               CAALKELLQNGMNGRTILGSTILEDEFTPFDVVRQCSGVTFQGKFKKIVKGTHHWMLLTF 3570
TR|A0A0U1WHI4|A0A0U1WHI4_CVHSA CAALKELLQNGMNGRTILGSTILEDEFTPFDVVRQCSGVTFQGKFKKIVKGTHHWMLLTF 3559
TR|A0A0U1WHG0|A0A0U1WHG0_CVHSA CAALKELLQNGMNGRTILGSTILEDEFTPFDVVRQCSGVTFQGKFKKIVKGTHHWMLLTF 3559
TR|A0A166ZL34|A0A166ZL34_9NIDO CAALKELLQNGMNGRTILGSTILEDEFTPFDVVRQCSGVTFQGKFKKIVKGTHHWMLLTF 3366
TR|R9QTB2|R9QTB2_CVHSA             CAALKELLQNGMNGRTILGSTILEDEFTPFDVVRQCSGVTFQGKFKKIVKGTHHWMLLTF 3556
TR|R9QTH2|R9QTH2_CVHSA             CAALKELLQNGMNGRTILGSTILEDEFTPFDVVRQCSGVTFQGKFKKIVKGTHHWVLLTF 3565
SP|P0C6U8|R1A_CVHSA                CAALKELLQNGMNGRTILGSTILEDEFTPFDVVRQCSGVTFQGKFKKIVKGTHHWMLLTF 3564
TR|Q6JH47|Q6JH47_CVHSA             CAALKELLQNGMNGRTILGSTILEDEFTPFDVVRQCSGVTFQGKFKKIVKGTHHWMLLTF 3564
TR|Q692E5|Q692E5_CVHSA             CAALKELLQNGMNGRTILGSTILEDEFTPFDVVRQCSGVTFQGKFKKIVKGTHHWMLLTF 3564
SP|P0C6F8|R1A_BCHK3                CAALKELLQNGMNGRTILGSTILEDEFTPFDVVRQCSGVTFQGKFKKIVKGTHHWMLLTF 3558
TR|A0A0K1Z0N1|A0A0K1Z0N1_CVHSA CAALKELLQNGMNGRTILGSTILEDEFTPFDVVRQCSGVTFQGKFKKIVKGTHHWMLLTF 3564
SP|P0C6F5|R1A_BC279                CAALKELLQNGMNGRTILGSTILEDEFTPFDVVRQCSGVTFQGKFKKIVKGTHHWMLLTF 3570
SP|P0C6T7|R1A_BCRP3                CAALKELLQNGMNGRTILGSTILEDEFTPFDVVRQCSGVTFQGKFKRIVKGTHHWMLLTF 3562
                                   **:*****************::********************. .*: :******:***:
```
