# Supplementary material for: Structural Genomics of SARS-CoV-2 Indicates Evolutionary Conserved Functional Regions of Viral Proteins
Source: Viruses. 2020 Mar 25;12(4):360. doi: 10.3390/v12040360 (PMC7232164; doi:10.3390/v12040360)
Supplement: Supplementary file 1 [file viruses-12-00360-s001.zip › wN_LBS_mapped.pdf]

Supplementary File 1. Ligand binding sites mapped on the sequence of wN protein aligned with the closest homologs found in UniProt.

Ligand BS:

N~2~,N~2~-DIMETHYL-N~1~-(6-OXO-5,6-DIHYDROPHENANTHRIDIN-2-YL)GLYCINAMIDE (4KXJ-P34, OC43)

ADENOSINE MONOPHOSPHATE (4LI4-AMP, OC43)

URIDINE-5'-MONOPHOSPHATE (4LM7-U5P, OC43)

GUANOSINE-5'-MONOPHOSPHATE (4LM9-5GP, OC43)

CYTIDINE-5'-MONOPHOSPHATE (4LMC-C5P, OC43)

6-chloranyl-7-(2-morpholin-4-ylethylamino)quinoline-5,8-dione (4LMT-CQD,OC43)

CLUSTAL O(1.2.4) multiple sequence alignment

```
YP_009724397                   MSDNGPQ-NQRNAPRITFGGPSDSTGSNQNGERSGARSKQRRPQGLPNNTASWFTALTQH 59
SP|P59595|NCAP_CVHSA           MSDNGPQSNQRSAPRITFGGPTDSTDNNQNGGRNGARPKQRRPQGLPNNTASWFTALTQH 60
TR|Q6UZF2|Q6UZF2_CVHSA         MSDNGPQSNQRSAPRITFGGPTDSTDNNQNGGRNGARPKQRRPQGLPNNTASWFTALTQH 60
TR|Q6JH41|Q6JH41_CVHSA         MSDNGPQSNQRSAPRITFGGPTDSTDNNQNGGRNGARPKQRRPQGLPNNTASWFTALTQH 60
TR|Q6UZE8|Q6UZE8_CVHSA         MSDNGPQSNQRSAPRITFGGPTDSTDNNQNGGRNGARPKQRRPQGLPNNTASWFTALTQH 60
TR|Q692D7|Q692D7_CVHSA         MSDNGPQSNQRSAPRITFGGPTDSTDNNQNGGRNGARPKQRRPQGLPNNTASWFTALTQH 60
TR|R9QTB4|R9QTB4_CVHSA         MSDNGPQ-NQRSAPRITFGGPTDSTDNNQDGGRSGARPKQRRPQGLPNNTASWFTALTQH 59
SP|Q3I5I7|NCAP_BCRP3           MSDNGPQ-NQRSAPRITFGGPTDSTDNNQDGGRSGARPKQRRPQGLPNNTASWFTALTQH 59
TR|A0A0K1YZZ7|A0A0K1YZZ7_CVHSA MSDNGPH-NQRSASRITFGGPTDSTDNNQNGGRNGARPKQRRPQGLPNNTASWFTALTQH 59
TR|R9QTA2|R9QTA2_CVHSA         MSDNGPQQNQRSAPRITFGGPTDSADNNQDGGRSGARPKQRRPQGLPNNTASWFTALTQH 60
SP|Q3LZX4|NCAP_BCHK3           MSDNGPQ-SQRSAPRITFGGPADSNDNNQDGGRSGARPKQRRPQGLPNNTASWFTALTQH 59
TR|A0A0U1WHJ1|A0A0U1WHJ1_CVHSA MSDNGPQ-NQRSAPRITFGGPSDSTDNNQDGGRSGVRPKQRRPQGLPNNTASWFTALTQH 59
SP|Q0Q468|NCAP_BC279           MSDNGPQ-NQRSAPRITFGGPSDSTDNNQDGGRSGARPKQRRPQGLPNNTASWFTALTQH 59
TR|A0A166ZLE5|A0A166ZLE5_9NIDO MSDNGTQ-NQRSASRITFGGPSDSTDNNQDGGRSGARPKQRRPQGLPNNTASWFTALTQH 59
TR|A0A0U1UZD6|A0A0U1UZD6_CVHSA MSDNGTQ-NQRSASRITFGGPSDSTDNNQDGGRSGARPKQRRPQGLPNNTASWFTALTQH 59
                               ***** : .**.* *******:** ..**:* *.*.* **********************

YP_009724397                   GKEDLKFPRGQGVPINTNSSPDDQIGYYRRATRRIRGGDGKMKDLSPRWYFYYLGTGPEA 119
SP|P59595|NCAP_CVHSA           GKEELRFPRGQGVPINTNSGPDDQIGYYRRATRRVRGGDGKMKELSPRWYFYYLGTGPEA 120
TR|Q6UZF2|Q6UZF2_CVHSA         GKEELRFPRGQGVPINTNSGPDDQIGYYRRATRRVRGGDGKMKELSPRWYFYYLGTGPEA 120
TR|Q6JH41|Q6JH41_CVHSA         GKEELRFPRGQGVPINTNSGPDDQIGYYRRATRRVRGGDGKMKELSPRWYFYYLGTGPEA 120
TR|Q6UZE8|Q6UZE8_CVHSA         GKEELRFPRGQGVPINTNSGPDDQIGYYRRATRRVRGGDGKMKELSPRWYFYYLGTGPEA 120
TR|Q692D7|Q692D7_CVHSA         GKEELRFPRGQGVPINTNSGPDDQIGFYRRATRRVRGGDGKMKELSPRWYFYYLGTGPEA 120
TR|R9QTB4|R9QTB4_CVHSA         GKEELRFPRGQGVPINTNSGKDDQIGYYRRATRRVRGGDGKMKELSPRWYFYYLGTGPEA 119
SP|Q3I5I7|NCAP_BCRP3           GKEELRFPRGQGVPINTNSGKDDQIGYYRRATRRVRGGDGKMKELSPRWYFYYLGTGPEA 119
TR|A0A0K1YZZ7|A0A0K1YZZ7_CVHSA GKEELRFPQGQGVPINTNSGPDDQIGYYRRATRRVRGGDGKMKELSPRWYFYYLGTGPEA 119
TR|R9QTA2|R9QTA2_CVHSA         GKEELRFPRGQGVPINTNSGKDDQIGYYRRATRRVRGGDGKMKELSPRWYFYYLGTGPEA 120
SP|Q3LZX4|NCAP_BCHK3           GKEELRFPRGQGVPINTNSGKDDQIGYYRRATRRVRGGDGKMKELSPRWYFYYLGTGPEA 119
TR|A0A0U1WHJ1|A0A0U1WHJ1_CVHSA GKEGLKFPQGQGVPINTNSGRDDQIGYYRRATRRVRGGDGKMKELSPRWYFYYLGTGPEA 119
SP|Q0Q468|NCAP_BC279           GKEELRFPRGQGVPINTNSGKDDQIGYYRRATRRVRGGDGKMKKLSPRWYFYYLGTGPEA 119
TR|A0A166ZLE5|A0A166ZLE5_9NIDO GKEGLKFPQGQGVPINTNSGTDDQIGYYRRATRRVRGGDGKMKELSPRWYFYYLGTGPEA 119
TR|A0A0U1UZD6|A0A0U1UZD6_CVHSA GKEGLKFPQGQGVPINTNSGRDDQIGYYRRATRRVRGGDGKMKELSPRWYFYYLGTGPEA 119
                               *** *:**:**********. *****:*******:********.****************
```

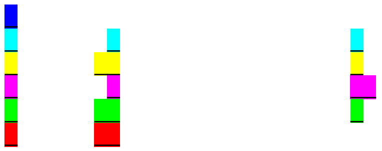

```
YP_009724397                        GLPYGANKDGIIWVATEGALNTPKDHIGTRNPANNAAIVLQLPQGTTLPKGFYAEGSRGG 179
SP|P59595|NCAP_CVHSA                SLPYGANKEGIVWVATEGALNTPKDHIGTRNPNNNAATVLQLPQGTTLPKGFYAEGSRGG 180
TR|Q6UZF2|Q6UZF2_CVHSA              SLPYGANKEGIVWVATEGALNTPKDHIGTRNPNNNAATVLQLPQGTTLPKGFYAEGSRGG 180
TR|Q6JH41|Q6JH41_CVHSA              SLPYGANKEGIVWVATEGALNTPKDHIGTRNPNNNAATVLQLPQGTTLPKGFYAEGSRGG 180
TR|Q6UZE8|Q6UZE8_CVHSA              SLPYGANKEGIVWVATEGALNTPKDHIGTRNPNNNAATVLQLPQGTTLPKGFYAEGSRGG 180
TR|Q692D7|Q692D7_CVHSA              SLPYGANKEGIVWVATEGALNTPKDHIGTRNPNNNAATVLQLPQGTTLPKGFYAEGSRGG 180
TR|R9QTB4|R9QTB4_CVHSA              SLPYGANKEGIIWVATEGALNTPKDHIGTRNPNNNAAIVLQLPQGTTLPKGFYAEGSRGG 179
SP|Q3I5I7|NCAP_BCRP3                SLPYGANKEGIVWVATEGALNTPKDHIGTRNPNNNAAIVLQLPQGTTLPKGFYAEGSRGG 179
TR|A0A0K1YZZ7|A0A0K1YZZ7_CVHSA SLPYGANKEGIIWVATEGALNTPKDHIGTRNPNNNAATVLQLPQGTTLPKGFYAEGSRGG 179
TR|R9QTA2|R9QTA2_CVHSA              SLPYGANKEGIVWVAIEGALNTPKDHIGTRNPNNNAAIVLQLPQGTTLPKGFYAEGSRGG 180
SP|Q3LZX4|NCAP_BCHK3                SLPYGANKEGIVWVATEGALNTPKDHIGTRNPNNNAAIVLQLPQGTTLPKGFYAEGSRGG 179
TR|A0A0U1WHJ1|A0A0U1WHJ1_CVHSA SLPYGANKEGIVWVATEGALNTPKDHIGTRNPNNNAAIVLQLPQGTTLPKGFYAEGSRNG 179
SP|Q0Q468|NCAP_BC279                SLPYGANKEGIVWVATEGALNTPKDHIGTRNPNNNAAIVLQLPQGTTLPKGFYAEGSRGG 179
TR|A0A166ZLE5|A0A166ZLE5_9NIDO SLPYGANKEGIVWVATEGALNTPKDHIGTRNPNNNAAIVLQLPQGTTLPKGFYAEGSRSG 179
TR|A0A0U1UZD6|A0A0U1UZD6_CVHSA SLPYGANKEGIVWVATEGALNTPKDHIGTRNPNNNAAIVLQLPQGTTLPKGFYAEGSRSG 179
                                    .*******:**:*** **************** **** ********************.*

YP_009724397                        SQASSRSSSRSRNSSRNSTPGSSRGTSPARMAGNGGDAALALLLLDRLNQLESKMSGKGQ 239
SP|P59595|NCAP_CVHSA                SQASSRSSSRSRGNSRNSTPGSSRGNSPARMASGGGETALALLLLDRLNQLESKVSGKGQ 240
TR|Q6UZF2|Q6UZF2_CVHSA              SQASSRSSSRSRGNSRNSTPGSSRGNSPARMASGGGETALALLLLDRLNQLESKVSGKGQ 240
TR|Q6JH41|Q6JH41_CVHSA              SQASSRSSSRSRGNSRNSTPGSSRGNSPARMASGGGETALALLLLDRLNQLESKVSGKGQ 240
TR|Q6UZE8|Q6UZE8_CVHSA              SQASSRSSSRSRGNSRNSTPGSSRGNSPARMASGGGETALALLLLDRLNQLESKVSGKGQ 240
TR|Q692D7|Q692D7_CVHSA              SQASSRSSSRSRGNSRNSTPGSSRGNSPARMASGGGETALALLLLDRLNQLESKVSGKGQ 240
TR|R9QTB4|R9QTB4_CVHSA              SQASSRSSSRSRGNSRNSTPGSSRGNSPARMASGGGETALALLLLDRLNQLESKVSGKGQ 239
SP|Q3I5I7|NCAP_BCRP3                SQASSRSSSRSRGNSRNSTPGSSRGNSPARMASGGGETALALLLLDRLNQLESKVSGRSQ 239
TR|A0A0K1YZZ7|A0A0K1YZZ7_CVHSA SQASSRSSSRSRGNSRNSTPGSSRGTSPARIASGGGETALALLLLDRLNQLESKVSGKGQ 239
TR|R9QTA2|R9QTA2_CVHSA              SQASSRSSSRSRGNSRNSTPGSSRGNSPARMASGGGETALALLLLDRLNQLESKVSGKGQ 240
SP|Q3LZX4|NCAP_BCHK3                SQSSSRSSSRSRGNSRNSTPGSSRGSSPARLASGGGETALALLLLDRLNQLESKVSGKGQ 239
TR|A0A0U1WHJ1|A0A0U1WHJ1_CVHSA SQASSRSSSRSRGNSRTSTPGSSRGNSPARVASGGGETALALLLLDRLNQLESKVSGKGQ 239
SP|Q0Q468|NCAP_BC279                SQASSRSSSRSRGNSRNSTPGSSRGNSPARMASGSGETALALLLLDRLNQLESKVSGKGQ 239
TR|A0A166ZLE5|A0A166ZLE5_9NIDO SQASSRSSSRSRGNSRTSTPGSSRGNSPARVASGGGETALALLLLDRLNQLESKVSGKGQ 239
TR|A0A0U1UZD6|A0A0U1UZD6_CVHSA SQASSRSSSRSRGNSRISTPGSSRGNSPARVASGGGETALALLLLDRLNQLESKVSGKGQ 239
                                    **:*********..** ********.****:*...*::****************:**:.*

YP_009724397                        QQQGQTVTKKSAAEASKKPRQKRTATKAYNVTQAFGRRGPEQTQGNFGDQELIRQGTDYK 299
SP|P59595|NCAP_CVHSA                QQQGQTVTKKSAAEASKKPRQKRTATKQYNVTQAFGRRGPEQTQGNFGDQDLIRQGTDYK 300
TR|Q6UZF2|Q6UZF2_CVHSA              QQQGQTVTKKSAAEASKKPRQKRTATKQYNVTQAFGRRGPEQTQGNFGDQDLIRQGTDYK 300
TR|Q6JH41|Q6JH41_CVHSA              QQQGQTVTKKSAAEASKKPRQKRTATKQYNVTQAFGRRGPEQTQGNFGDQDLIRQGTDYK 300
TR|Q6UZE8|Q6UZE8_CVHSA              QQQGQTVTKKSAAEASKKPRQKRTATKQYNVTQAFGRRGPEQTQGNFGDQDLIRQGTDYK 300
TR|Q692D7|Q692D7_CVHSA              QQQGQTVTKKSAAEASKKPRQKRTATKQYNVTQAFGRRGPEQTQGNFGDQDLIRQGTDYK 300
TR|R9QTB4|R9QTB4_CVHSA              QQQGQTVTKKSAAEASKKPRQKRTATKSYNVTQAFGRRGPEQTQGNFGDQDLIRQGTDYK 299
SP|Q3I5I7|NCAP_BCRP3                QQQGQTVTKKSAAEASKKPRQKRTATKQYNVTQAFGRRGPEQTQGNFGDQELIRQGTDYK 299
TR|A0A0K1YZZ7|A0A0K1YZZ7_CVHSA QQQGQTVTKKSAAEASKKPRQKRTATKQYNVTQAFGRRGPDQTQGNFGDQDLIRQGTDYK 299
TR|R9QTA2|R9QTA2_CVHSA              QQQGQTVTKKSAAEASKKPRQKRTATKQYNVTQAFGRRGPEQTQGNFGDQELIRQGTDYK 300
SP|Q3LZX4|NCAP_BCHK3                QQPGQTVTKKSAAEASKKPRQKRTATKQYNVTQAFGRRGPEQTQGNFGDQELIRQGIDYK 299
TR|A0A0U1WHJ1|A0A0U1WHJ1_CVHSA QQQGQTVTKKSASEASKKPRQKRTATKQYNVTQAFGRRGPDQTQGNFGDQELIRQGTDYK 299
SP|Q0Q468|NCAP_BC279                QQQGQTVTKKSAAEASKKPRQKRTATKSYNVTQAFGRRGPEQTQGNFGDQDLIRQGTDYK 299
TR|A0A166ZLE5|A0A166ZLE5_9NIDO QQQGQTVTKKSASEASKKPRQKRTATKQYNVTQAFGRRGPDQTQGNFGDQELIRQGTDYK 299
TR|A0A0U1UZD6|A0A0U1UZD6_CVHSA QQQGQTVTKKSASEASKKPRQKRTATKQYNVTQAFGRRGPDQTQGNFGDQELIRQGTDYK 299
                                    ** *********:************* ************:*********:***** ***

YP_009724397                        HWPQIAQFAPSASAFFGMSRIGMEVTPSGTWLTYTGAIKLDDKDPNFKDQVILLNKHIDA 359
SP|P59595|NCAP_CVHSA                HWPQIAQFAPSASAFFGMSRIGMEVTPSGTWLTYHGAIKLDDKDPQFKDNVILLNKHIDA 360
TR|Q6UZF2|Q6UZF2_CVHSA              HWPQIAQFAPSASAFFGMSRIGMEVTPSGTWLTYHGAIKLDDKDPQFKDNVILLNKHIDA 360
TR|Q6JH41|Q6JH41_CVHSA              HWPQIAQFAPSASAFFGMSRIGMEVTPSGTWLTYHGAIKLDDKDPQFKDNVILLNKHIDA 360
TR|Q6UZE8|Q6UZE8_CVHSA              HWPQIAQFAPSASAFFGMSRIGMEVTPSGTWLTYHGAIKLDDKDPQFKDNVILLNKHIDA 360
TR|Q692D7|Q692D7_CVHSA              HWPQIAQFAPSASAFFGMSRIGMEVTPSGTWLTYHGAIKLDDKDPQFKDNVILLNKHIDA 360
TR|R9QTB4|R9QTB4_CVHSA              HWPQIAQFAPSASAFFGMSRIGMEVTPSGTWLTYHGAIKLDDKDPQFKDNVILLNKHIDA 359
SP|Q3I5I7|NCAP_BCRP3                HWPQIAQFAPSASAFFGMSRIGMEVTPSGTWLTYHGAIKLDDKDPQFKDNVILLNKHIDA 359
TR|A0A0K1YZZ7|A0A0K1YZZ7_CVHSA HWPQIAQFAPSASAFFGMSRIGMEVTPSGTWLTYHGAIKLDDKDPQFKDNVILLNKHIDA 359
TR|R9QTA2|R9QTA2_CVHSA              HWPQIAQFAPSASAFFGMSRIGMEVTPSGTWLTYHGAIKLDDKDPQFKDNVILLNKHIDA 360
SP|Q3LZX4|NCAP_BCHK3                HWPQIAQFAPSASAFFGMSRIGMEVTPSGTWLTYHGAIKLDDKDPQFKDNVILLNKHIDA 359
TR|A0A0U1WHJ1|A0A0U1WHJ1_CVHSA HWPQIAQFAPSASAFFGMSRIGMEVTPSGTWLTYHGAIKLDDKDPQFKDNVILLNKHIDA 359
SP|Q0Q468|NCAP_BC279                YWPQIAQFAPSASAFFGMSRIGMEVTPLGTWLTYHGAIKLDDKDPQFKDNVILLNKHIDA 359
TR|A0A166ZLE5|A0A166ZLE5_9NIDO HWPQIAQFAPSASAFFGMSRIGMEVTPSGTWLTYHGAIKLDDKDPQFKDNVILLNKHIDA 359
TR|A0A0U1UZD6|A0A0U1UZD6_CVHSA HWPQIAQFAPSASAFFGMSRIGMEVTPSGTWLTYHGAIKLDDKDPQFKDNVILLNKHIDA 359
                                    :************************* ****** *********:***:**********
```

```
YP_009724397                    YKTFPPTEPKKDKKKKADETQALPQRQKKQQTVTLLPAADLDDFSKQLQQSMSSADSTQA 419
SP|P59595|NCAP_CVHSA            YKTFPPTEPKKDKKKKTDEAQPLPQRQKKQPTVTLLPAADMDDFSRQLQNSMSGASADST 420
TR|Q6UZF2|Q6UZF2_CVHSA          YKTFPPTEPKKDKKKKTDEAQPLPQRQKKQPTVTLLPAADMDDFSRQLQNSMSGASADST 420
TR|Q6JH41|Q6JH41_CVHSA          YKTFPPTEPKKDKKKKTDEAQPLPQRQKKQPTVTLLPAADMDDFSRQLQNSMSGASADST 420
TR|Q6UZE8|Q6UZE8_CVHSA          YKTFPPTEPKKDKKKKTDEAQPLPQRQKKQPTVTLLPAADMDDFSRQLQNSMSGASADST 420
TR|Q692D7|Q692D7_CVHSA          YKTFPPTEPKKDKKKKTDEAQPLPQRQKKQPTVTLLPAADMDDFSRQLQNSMSGASADST 420
TR|R9QTB4|R9QTB4_CVHSA          YKTFPPTEPKKDKKKKTDEAQPLPQRK-KQPTVTLLPAADMDDFSRQLQNSMSGASADST 418
SP|Q3I5I7|NCAP_BCRP3            YKIFPPTEPKKDKKKKTDEAQPLPQRQKKQPTVTLLPAADMDDFSRQLQNSMSGASADST 419
TR|A0A0K1YZZ7|A0A0K1YZZ7_CVHSA YKTFPPTEPKKDKKKKTDEAQPLPQRQKKQPTVTLLPAADMDDFSRQLQNSMSGASADST 419
TR|R9QTA2|R9QTA2_CVHSA          YKTFPPTEPKKDKKKKTDEAQPLPQRQKKQPTVTLLPAADMDDFSRQLQNSMSGASADST 420
SP|Q3LZX4|NCAP_BCHK3            YKTFPPTEPKKDKKKKTDEAQPLPQRQKKQPTVTLLPAADMDDFSRQLQHSMSGASADST 419
TR|A0A0U1WHJ1|A0A0U1WHJ1_CVHSA YKTFPPTEPKKDKKKKTDEAQPLPQRQKKQPTVTLLPAADMDDFSRQLQNSMSGASADST 419
SP|Q0Q468|NCAP_BC279            YKAFPPTEPKKDKKKKTDEAQPLPQRK-KQPTVTLLPAADMDDFSRQLQNSMSGASADST 418
TR|A0A166ZLE5|A0A166ZLE5_9NIDO YKTFPPTEPKKDKKKKTDEAQPLPQRK-KQPTVTLLPAADMDDFSRQLQNSMSGASADST 418
TR|A0A0U1UZD6|A0A0U1UZD6_CVHSA YKTFPPTEPKKDKKKKTDEAQPLPQRK-KQPTVTLLPAADMDDFSRQLQNSMSGASADST 418
                                ** *************:**:* ****: ** *********:****:***:***.*.: .:

YP_009724397                    --
SP|P59595|NCAP_CVHSA            QA 422
TR|Q6UZF2|Q6UZF2_CVHSA          QA 422
TR|Q6JH41|Q6JH41_CVHSA          QA 422
TR|Q6UZE8|Q6UZE8_CVHSA          QA 422
TR|Q692D7|Q692D7_CVHSA          QA 422
TR|R9QTB4|R9QTB4_CVHSA          QA 420
SP|Q3I5I7|NCAP_BCRP3            QA 421
TR|A0A0K1YZZ7|A0A0K1YZZ7_CVHSA QA 421
TR|R9QTA2|R9QTA2_CVHSA          QA 422
SP|Q3LZX4|NCAP_BCHK3            QA 421
TR|A0A0U1WHJ1|A0A0U1WHJ1_CVHSA QA 421
SP|Q0Q468|NCAP_BC279            QA 420
TR|A0A166ZLE5|A0A166ZLE5_9NIDO QA 420
TR|A0A0U1UZD6|A0A0U1UZD6_CVHSA QA 420
```
