# Supplementary material for: Structural Genomics of SARS-CoV-2 Indicates Evolutionary Conserved Functional Regions of Viral Proteins
Source: Viruses. 2020 Mar 25;12(4):360. doi: 10.3390/v12040360 (PMC7232164; doi:10.3390/v12040360)
Supplement: Supplementary file 1 [file viruses-12-00360-s001.zip › wNsp14_LBS_mapped.pdf]

Supplementary File 6. Ligand binding sites mapped on the sequence of wNsp14 protein aligned with the closest homologs found in UniProt.

GUANOSINE-P3-ADENOSINE-5',5'-TRIPHOSPHATE (5C8S-G3A, SARS)

S-ADENOSYL-L-HOMOCYSTEINE (5C8S-SAH, SARS)

S-ADENOSYLMETHIONINE (5C8T-SAM, SARS)

```
QHN73794                        VIFTQTTETAHSCNVNRFNVAITRAKVGILCIMSDRDLYDKLQFTSLEIPRRNVATLQAE 5927
SP|P0C6X7|R1AB_CVHSA            VIFTQTTETAHSCNVNRFNVAITRAKIGILCIMSDRDLYDKLQFTSLEIPRRNVATLQAE 5904
TR|Q6UZF5|Q6UZF5_CVHSA          VIFTQTTETAHSCNVNRFNVAITRAKIGILCIMSDRDLYDKLQFTSLEIPRRNVATLQAE 5904
TR|Q6UZF1|Q6UZF1_CVHSA          VIFTQTTETAHSCNVNRFNVAITRAKIGILCIMSDRDLYDKLQFTSLEIPRRNVATLQAE 5904
TR|Q6JH48|Q6JH48_CVHSA          VIFTQTTETAHSCNVNRFNVAITRAKIGILCIMSDRDLYDKLQFTSLEIPRRNVATLQAE 5904
TR|Q692E6|Q692E6_CVHSA          VIFTQTTETAHSCNVNRFNVAITRAKIGILCIMSDRDLYDKLQFTSLEIPRRNVATLQAE 5904
TR|A0A0K1YZY7|A0A0K1YZY7_CVHSA  VIFTQTTETAHSCNVNRFNVAITRAKIGILCIMSDRDLYDKLQFTSLEVPRRNVATLQAE 5904
SP|P0C6W2|R1AB_BCHK3            VIFTQTTETAHSCNVNRFNVAITRAKIGILCIMSDRDLYDKLQFTSLEVPRRNVATLQAE 5898
SP|P0C6W6|R1AB_BCRP3            VIFTQTTETAHSCNVNRFNVAITRAKIGILCIMSDRDLYDKLQFTSLEVPRRNVATLQAE 5902
SP|P0C6V9|R1AB_BC279            VIFTQTTETAHSCNVNRFNVAITRAKIGILCIMSDRDLYDKLQFTSLEVPRRNVATLQAE 5910
TR|A0A0U1WHI4|A0A0U1WHI4_CVHSA  VIFTQTTETAHSCNVNRFNVAITRAKIGILCIMSDRDLYDKLQFTSLEVPRRNVATLQAE 5899
TR|A0A0U1WHG0|A0A0U1WHG0_CVHSA  VIFTQTTETAHSCNVNRFNVAITRAKIGILCIMSDRDLYDKLQFTSLEVPRRNVATLQAE 5899
TR|A0A166ZL34|A0A166ZL34_9NIDO  VIFTQTTETAHSCNVNRFNVAITRAKIGILCIMSDRDLYDKLQFTSLEVPRRNVATLQAE 5706
TR|R9QTB2|R9QTB2_CVHSA          ------------------------------------------------------------
TR|R9QTH2|R9QTH2_CVHSA          ------------------------------------------------------------
SP|P0C6U8|R1A_CVHSA             ------------------------------------------------------------
TR|Q6JH47|Q6JH47_CVHSA          ------------------------------------------------------------
TR|Q692E5|Q692E5_CVHSA          ------------------------------------------------------------
SP|P0C6F8|R1A_BCHK3             ------------------------------------------------------------
TR|A0A0K1Z0N1|A0A0K1Z0N1_CVHSA  ------------------------------------------------------------
SP|P0C6F5|R1A_BC279             ------------------------------------------------------------
SP|P0C6T7|R1A_BCRP3             ------------------------------------------------------------


QHN73794                        NVTGLFKDCSKVITGLHPTQAPTHLSVDTKFKTEGLCVDIPGIPKDMTYRRLISMMGFKM 5987
SP|P0C6X7|R1AB_CVHSA            NVTGLFKDCSKIITGLHPTQAPTHLSVDIKFKTEGLCVDIPGIPKDMTYRRLISMMGFKM 5964
TR|Q6UZF5|Q6UZF5_CVHSA          NVTGLFKDCSKIITGLHPTQAPTHLSVDIKFKTEGLCVDIPGIPKDMTYRRLISMMGFKM 5964
TR|Q6UZF1|Q6UZF1_CVHSA          NVTGLFKDCSKIITGLHPTQAPTHLSVDIKFKTEGLCVDIPGIPKDMTYRRLISMMGFKM 5964
TR|Q6JH48|Q6JH48_CVHSA          NVTGLFKDCSKIITGLHPTQAPTHLSVDIKFKTEGLCVDIPGIPKDMTYRRLISMMGFKM 5964
TR|Q692E6|Q692E6_CVHSA          NVTGLFKDCSKIITGLHPTQAPTHLSVDIKFKTEGLCVDIPGIPKDMTYRRLISMMGFKM 5964
TR|A0A0K1YZY7|A0A0K1YZY7_CVHSA  NVTGLFKDCSKIITGLHPTQAPTHLSVDTKFKTEGLCVDIPGIPKDMTYRRLISMMGFKM 5964
SP|P0C6W2|R1AB_BCHK3            NVTGLFKDCSKIITGLHPTQAPTHLSVDTKFKTEGLCVDIPGIPKDMTYRRLISMMGFKM 5958
SP|P0C6W6|R1AB_BCRP3            NVTGLFKDCSKIITGLHPTQAPTHLSVDTKFKTEGLCVDIPGIPKDMTYRRLISMMGFKM 5962
SP|P0C6V9|R1AB_BC279            NVTGLFKDCSKIITGLHPTQAPTHLSVDTKFKTEGLCVDIPGIPKDMTYRRLISMMGFKM 5970
TR|A0A0U1WHI4|A0A0U1WHI4_CVHSA  NVTGLFKDCSKIITGLHPTQAPTHLSVDTKFKTEGLCVDIPGIPKDMTYRRLISMMGFKM 5959
TR|A0A0U1WHG0|A0A0U1WHG0_CVHSA  NVTGLFKDCSKIITGLHPTQAPTHLSVDTKFKTEGLCVDIPGIPKDMTYRRLISMMGFKM 5959
TR|A0A166ZL34|A0A166ZL34_9NIDO  NVTGLFKDCSKIITGLHPTQAPTHLSVDTKFKTEGLCVDIPGIPKDMTYRRLISMMGFKM 5766
TR|R9QTB2|R9QTB2_CVHSA          ------------------------------------------------------------
TR|R9QTH2|R9QTH2_CVHSA          ------------------------------------------------------------
SP|P0C6U8|R1A_CVHSA             ------------------------------------------------------------
TR|Q6JH47|Q6JH47_CVHSA          ------------------------------------------------------------
TR|Q692E5|Q692E5_CVHSA          ------------------------------------------------------------
SP|P0C6F8|R1A_BCHK3             ------------------------------------------------------------
TR|A0A0K1Z0N1|A0A0K1Z0N1_CVHSA  ------------------------------------------------------------
SP|P0C6F5|R1A_BC279             ------------------------------------------------------------
SP|P0C6T7|R1A_BCRP3             ------------------------------------------------------------


QHN73794                        NYQVNGYPNMFITREEAIRHVRAWIGFDVEGCHATREAVGTNLPLQLGFSTGVNLVAVPT 6047
SP|P0C6X7|R1AB_CVHSA            NYQVNGYPNMFITREEAIRHVRAWIGFDVEGCHATRDAVGTNLPLQLGFSTGVNLVAVPT 6024
TR|Q6UZF5|Q6UZF5_CVHSA          NYQVNGYPNMFITREEAIRHVRAWIGFDVEGCHATRDAVGTNLPLQLGFSTGVNLVAVPT 6024
TR|Q6UZF1|Q6UZF1_CVHSA          NYQVNGYPNMFITREEAIRHVRAWIGFDVEGCHATRDAVGTNLPLQLGFSTGVNLVAVPT 6024
TR|Q6JH48|Q6JH48_CVHSA          NYQVNGYPNMFITREEAIRHVRAWIGFDVEGCHATRDAVGTNLPLQLGFSTGVNLVAVPT 6024
TR|Q692E6|Q692E6_CVHSA          NYQVNGYPNMFITREEAIRHVRAWIGFDVEGCHATRDAVGTNLPLQLGFSTGVNLVAVPT 6024
TR|A0A0K1YZY7|A0A0K1YZY7_CVHSA  NYQVNGYPNMFITREEAIRHVRAWIGFDVEGCHATRDAVGTNLPLQLGFSTGVNLVAVPT 6024
SP|P0C6W2|R1AB_BCHK3            NYQVNGYPNMFITREEAIRHVRAWIGFDVEGCHATRDAVGTNLPLQLGFSTGVNLVAVPT 6018
SP|P0C6W6|R1AB_BCRP3            NYQVNGYPNMFITREEAIRHVRAWIGFDVEGCHATRDAVGTNLPLQLGFSTGVNLVAIPT 6022
SP|P0C6V9|R1AB_BC279            NYQVNGYPNMFITREEAIRHVRAWIGFDVEGCHATRDAVGTNLPLQLGFSTGVNLVAVPT 6030
TR|A0A0U1WHI4|A0A0U1WHI4_CVHSA  NYQVNGYPNMFITREEAIRHVRAWIGFDVEGCHATRDAVGTNLPLQLGFSTGVNLVAVPT 6019
TR|A0A0U1WHG0|A0A0U1WHG0_CVHSA  NYQVNGYPNMFITREEAIRHVRAWIGFDVEGCHATRDAVGTNLPLQLGFSTGVNLVAVPT 6019
TR|A0A166ZL34|A0A166ZL34_9NIDO  NYQVNGYPNMFITREEAIRHVRAWIGFDVEGCHATRDAVGTNLPLQLGFSTGVNLVAVPT 5826
TR|R9QTB2|R9QTB2_CVHSA          ------------------------------------------------------------
TR|R9QTH2|R9QTH2_CVHSA          ------------------------------------------------------------
SP|P0C6U8|R1A_CVHSA             ------------------------------------------------------------
TR|Q6JH47|Q6JH47_CVHSA          ------------------------------------------------------------
TR|Q692E5|Q692E5_CVHSA          ------------------------------------------------------------
SP|P0C6F8|R1A_BCHK3             ------------------------------------------------------------
TR|A0A0K1Z0N1|A0A0K1Z0N1_CVHSA  ------------------------------------------------------------
SP|P0C6F5|R1A_BC279             ------------------------------------------------------------
SP|P0C6T7|R1A_BCRP3             ------------------------------------------------------------
```

```
QHN73794                           GYVDTPNNTDFSRVSAKPPPGDQFKHLIPLMYKGLPWNVVRIKIVQMLSDTLKNLSDRVV 6107
SP|P0C6X7|R1AB_CVHSA               GYVDTENNTEFTRVNAKPPPGDQFKHLIPLMYKGLPWNVVRIKIVQMLSDTLKGLSDRVV 6084
TR|Q6UZF5|Q6UZF5_CVHSA             GYVDTENNTEFTRVNAKPPPGDQFKHLIPLMYKGLPWNVVRIKIVQMLSDTLKGLSDRVV 6084
TR|Q6UZF1|Q6UZF1_CVHSA             GYVDTENNTEFTRVNAKPPPGDQFKHLIPLMYKGLPWNVVRIKIVQMLSDTLKGLSDRVV 6084
TR|Q6JH48|Q6JH48_CVHSA             GYVDTENNTEFTRVNAKPPPGDQFKHLIPLMYKGLPWNVVRIKIVQMLSDTLKGLSDRVV 6084
TR|Q692E6|Q692E6_CVHSA             GYVDTENNTEFTRVNAKPPPGDQFKHLIPLMYKGLPWNVVRIKIVQMLSDTLKGLSDRVV 6084
TR|A0A0K1YZY7|A0A0K1YZY7_CVHSA GYVDTENNTEFTRVNAKPPPGDQFKHLIPLMYKGLPWNVVRIKIVQMLSDTLKGLSDRVV 6084
SP|P0C6W2|R1AB_BCHK3               GYVDTENSTEFTRVNAKPPPGDQFKHLIPLMYKGLPWNVVRIKIVQMLSDTLKGLSDRVV 6078
SP|P0C6W6|R1AB_BCRP3               GYVDTENNTEFTRVNAKPPPGDQFKHLIPLMYKGLPWNVVRIKIVQMLSDTLKGLSDRVV 6082
SP|P0C6V9|R1AB_BC279               GYVDTENNTEFTRVNAKPPPGDQFKHLIPLMYKGLPWNVVRIKIVQMLSDTLKGLSDRVV 6090
TR|A0A0U1WHI4|A0A0U1WHI4_CVHSA GYVDTENNTEFTRVNAKPPPGDQFKHLIPLMYKGLPWNVVRIKIVQMLSDTLKGLSDRVV 6079
TR|A0A0U1WHG0|A0A0U1WHG0_CVHSA GYVDTENNTEFTRVNAKPPPGDQFKHLIPLMYKGLPWSVVRIKIVQMLSDTLKGLSDRVV 6079
TR|A0A166ZL34|A0A166ZL34_9NIDO GYVDTENNTEFTRVNAKPPPGDQFKHLIPLMYKGLPWSVVRIKIVQMLSDTLKGLSDRVV 5886
TR|R9QTB2|R9QTB2_CVHSA             ------------------------------------------------------------
TR|R9QTH2|R9QTH2_CVHSA             ------------------------------------------------------------
SP|P0C6U8|R1A_CVHSA                ------------------------------------------------------------
TR|Q6JH47|Q6JH47_CVHSA             ------------------------------------------------------------
TR|Q692E5|Q692E5_CVHSA             ------------------------------------------------------------
SP|P0C6F8|R1A_BCHK3                ------------------------------------------------------------
TR|A0A0K1Z0N1|A0A0K1Z0N1_CVHSA ------------------------------------------------------------
SP|P0C6F5|R1A_BC279                ------------------------------------------------------------
SP|P0C6T7|R1A_BCRP3                ------------------------------------------------------------

QHN73794                           FVLWAHGFELTSMKYFVKIGPERTCCLCDRRATCFSTASDTYACWHHSIGFDYVYNPFMI 6167
SP|P0C6X7|R1AB_CVHSA               FVLWAHGFELTSMKYFVKIGPERTCCLCDKRATCFSTSSDTYACWNHSVGFDYVYNPFMI 6144
TR|Q6UZF5|Q6UZF5_CVHSA             FVLWAHGFELTSMKYFVKIGPERTCCLCDKRATCFSTSSDTYACWNHSVGFDYVYNPFMI 6144
TR|Q6UZF1|Q6UZF1_CVHSA             FVLWAHGFELTSMKYFVKIGPERTCCLCDKRATCFSTSSDTYACWNHSVGFDYVYNPFMI 6144
TR|Q6JH48|Q6JH48_CVHSA             FVLWAHGFELTSMKYFVKIGPERTCCLCDKRATCFSTSSDTYACWNHSVGFDYVYNPFMI 6144
TR|Q692E6|Q692E6_CVHSA             FVLWAHGFELTSMKYFVKIGPERTCCLCDKRATCFSTSSDTYACWNHSVGFDYVYNPFMI 6144
TR|A0A0K1YZY7|A0A0K1YZY7_CVHSA FVLWAHGFELTSMKYFVKIGPERTCCLCDKRATCFSTSSDTYACWNHSVGFDYVYNPFMI 6144
SP|P0C6W2|R1AB_BCHK3               FVLWAHGFELTSMKYFVKIGPERTCCLCDKRATCFSTSSDTYACWNHSVGFDYVYNPFMI 6138
SP|P0C6W6|R1AB_BCRP3               FVLWAHGFELTSMKYFVKIGPERTCCLCDKRATCFSTSSDTYACWNHSVGFDYVYNPFMI 6142
SP|P0C6V9|R1AB_BC279               FVLWAHGFELTSMKYFVKIGPERTCCLCDRRATCFSTSSDTYACWNHSVGFDYVYNPFMI 6150
TR|A0A0U1WHI4|A0A0U1WHI4_CVHSA FVLWAHGFELTSMKYFVKIGPERTCCLCDKRATCFSTSSDTYACWNHSVGFDYVYNPFMI 6139
TR|A0A0U1WHG0|A0A0U1WHG0_CVHSA FVLWAHGFELTSMKYFVKIGSERTCCLCDKRATCFSTSSDTYACWNHSVGFDYVYNPFMI 6139
TR|A0A166ZL34|A0A166ZL34_9NIDO FVLWAHGFELTSMKYFVKIGSERTCCLCDKRATCFSTSSDTYACWNHSVGFDYVYNPFMI 5946
TR|R9QTB2|R9QTB2_CVHSA             ------------------------------------------------------------
TR|R9QTH2|R9QTH2_CVHSA             ------------------------------------------------------------
SP|P0C6U8|R1A_CVHSA                ------------------------------------------------------------
TR|Q6JH47|Q6JH47_CVHSA             ------------------------------------------------------------
TR|Q692E5|Q692E5_CVHSA             ------------------------------------------------------------
SP|P0C6F8|R1A_BCHK3                ------------------------------------------------------------
TR|A0A0K1Z0N1|A0A0K1Z0N1_CVHSA ------------------------------------------------------------
SP|P0C6F5|R1A_BC279                ------------------------------------------------------------
SP|P0C6T7|R1A_BCRP3                ------------------------------------------------------------

QHN73794                           DVQQWGFTGNLQSNHDLYCQVHGNAHVASCDAIMTRCLAVHECFVKRVDWTIEYPIIGDE 6227
SP|P0C6X7|R1AB_CVHSA               DVQQWGFTGNLQSNHDQHCQVHGNAHVASCDAIMTRCLAVHECFVKRVDWSVEYPIIGDE 6204
TR|Q6UZF5|Q6UZF5_CVHSA             DVQQWGFTGNLQSNHDQHCQVHGNAHVASCDAIMTRCLAVHECFVKRVDWSVEYPIIGDE 6204
TR|Q6UZF1|Q6UZF1_CVHSA             DVQQWGFTGNLQSNHDQHCQVHGNAHVASCDAIMTRCLAVHECFVKRVDWSVEYPIIGDE 6204
TR|Q6JH48|Q6JH48_CVHSA             DVQQWGFTGNLQSNHDQHCQVHGNAHVASCDAIMTRCLAVHECFVKRVDWSVEYPIIGDE 6204
TR|Q692E6|Q692E6_CVHSA             DVQQWGFTGNLQSNHDQHCQVHGNAHVASCDAIMTRCLAVHECFVKRVDWSVEYPIIGDE 6204
TR|A0A0K1YZY7|A0A0K1YZY7_CVHSA DVQQWGFTGNLQSNHDQHCQVHGNAHVASCDAIMTRCLAVHECFVKRVDWSVEYPIIGDE 6204
SP|P0C6W2|R1AB_BCHK3               DVQQWGFTGNLQSNHDQHCQVHGNAHVASCDAIMTRCLAVHECFVKRVDWSVEYPIIGDE 6198
SP|P0C6W6|R1AB_BCRP3               DVQQWGFTGNLQSNHDQHCQVHGNAHVASCDAIMTRCLAVHECFVKRVDWSVEYPIIGDE 6202
SP|P0C6V9|R1AB_BC279               DVQQWGFTGNLQSNHDQHCQVHGNAHVASCDAIMTRCLAVHECFVKRVDWSVEYPIIGDE 6210
TR|A0A0U1WHI4|A0A0U1WHI4_CVHSA DVQQWGLTGNLQSNHDQHCQVHGNAHVASCDAIMTRCLAVHECFVKRVDWSVEYPIIGDE 6199
TR|A0A0U1WHG0|A0A0U1WHG0_CVHSA DVQQWGFTGNLQSNHDQHCQVHGNAHVASCDAIMTRCLAVHECFVKRVDWSVEYPVIGDE 6199
TR|A0A166ZL34|A0A166ZL34_9NIDO DVQQWGFTGNLQSNHDQHCQVHGNAHVASCDAIMTRCLAVHECFVKRVDWSVEYPVIGDE 6006
TR|R9QTB2|R9QTB2_CVHSA             ------------------------------------------------------------
TR|R9QTH2|R9QTH2_CVHSA             ------------------------------------------------------------
SP|P0C6U8|R1A_CVHSA                ------------------------------------------------------------
TR|Q6JH47|Q6JH47_CVHSA             ------------------------------------------------------------
TR|Q692E5|Q692E5_CVHSA             ------------------------------------------------------------
SP|P0C6F8|R1A_BCHK3                ------------------------------------------------------------
TR|A0A0K1Z0N1|A0A0K1Z0N1_CVHSA ------------------------------------------------------------
SP|P0C6F5|R1A_BC279                ------------------------------------------------------------
SP|P0C6T7|R1A_BCRP3                ------------------------------------------------------------
```

```
QHN73794                            LKINAACRKVQHMVVKAALLADKFPVLHDIGNPKAIKCVPQADVEWKFYDAQPCSDKAYK 6287
SP|P0C6X7|R1AB_CVHSA                LRVNSACRKVQHMVVKSALLADKFPVLHDIGNPKAIKCVPQAEVEWKFYDAQPCSDKAYK 6264
TR|Q6UZF5|Q6UZF5_CVHSA              LRVNSACRKVQHMVVKSALLADKFPVLHDIGNPKAIKCVPQAEVEWKFYDAQPCSDKAYK 6264
TR|Q6UZF1|Q6UZF1_CVHSA              LRVNSACRKVQHMVVKSALLADKFPVLHDIGNPKAIKCVPQAEVEWKFYDAQPCSDKAYK 6264
TR|Q6JH48|Q6JH48_CVHSA              LRVNSACRKVQHMVVKSALLADKFPVLHDIGNPKAIKCVPQAEVEWKFYDAQPCSDKAYK 6264
TR|Q692E6|Q692E6_CVHSA              LRVNSACRKVQHMVVKSALLADKFPVLHDIGNPKAIKCVPQAEVEWKFYDAQPCSDKAYK 6264
TR|A0A0K1YZY7|A0A0K1YZY7_CVHSA      LKINSACRKVQHMVVKSALLADKFPVLHDIGNPKAIKCVPQAEVEWKFYDAQPCSDKAYK 6264
SP|P0C6W2|R1AB_BCHK3                LKINAACRKVQHMVVKSALLADKFTVLHDIGNPKAIRCVPQAEVDWKFYDAQPCSDKAYK 6258
SP|P0C6W6|R1AB_BCRP3                LKINSACRKVQHMVVKSALLADKFPVLHDIGNPKAIKCVPQAEVEWKFYDAQPCSDKAYK 6262
SP|P0C6V9|R1AB_BC279                LKINAACRKVQHMVVKSALLADKFSVLHDIGNPKAIKCVPQAEVDWKFYDAQPCSDKAYK 6270
TR|A0A0U1WHI4|A0A0U1WHI4_CVHSA      LKINAACRKVQHMVVKSALLADKFPVLHDIGNPKAIKCVPQADVEWKFYDAQPCSDKAYK 6259
TR|A0A0U1WHG0|A0A0U1WHG0_CVHSA      LKINAACRKVQHMVVKSALLADKFPVLHDIGNPKAIKCVPQADVEWKFYDVQPCSDKAYK 6259
TR|A0A166ZL34|A0A166ZL34_9NIDO      LKINAACRKVQHMVVKSALLADKFPVLHDIGNPKAIKCVPQADVEWKFYDVQPCSDKAYK 6066
TR|R9QTB2|R9QTB2_CVHSA              ------------------------------------------------------------
TR|R9QTH2|R9QTH2_CVHSA              ------------------------------------------------------------
SP|P0C6U8|R1A_CVHSA                 ------------------------------------------------------------
TR|Q6JH47|Q6JH47_CVHSA              ------------------------------------------------------------
TR|Q692E5|Q692E5_CVHSA              ------------------------------------------------------------
SP|P0C6F8|R1A_BCHK3                 ------------------------------------------------------------
TR|A0A0K1Z0N1|A0A0K1Z0N1_CVHSA      ------------------------------------------------------------
SP|P0C6F5|R1A_BC279                 ------------------------------------------------------------
SP|P0C6T7|R1A_BCRP3                 ------------------------------------------------------------

QHN73794                            IEELFYSYATHSDKFTDGVCLFWNCNVDRYPANSIVCRFDTRVLSNLNLPGCDGGSLYVN 6347
SP|P0C6X7|R1AB_CVHSA                IEELFYSYATHHDKFTDGVCLFWNCNVDRYPANAIVCRFDTRVLSNLNLPGCDGGSLYVN 6324
TR|Q6UZF5|Q6UZF5_CVHSA              IEELFYSYATHHDKFTDGVCLFWNCNVDRYPANAIVCRFDTRVLSNLNLPGCDGGSLYVN 6324
TR|Q6UZF1|Q6UZF1_CVHSA              IEELFYSYATHHDKFTDGVCLFWNCNVDRYPANAIVCRFDTRVLSNLNLPGCDGGSLYVN 6324
TR|Q6JH48|Q6JH48_CVHSA              IEELFYSYATHHDKFTDGVCLFWNCNVDRYPANAIVCRFDTRVLSNLNLPGCDGGSLYVN 6324
TR|Q692E6|Q692E6_CVHSA              IEELFYSYATHHDKFTDGVCLFWNCNVDRYPANAIVCRFDTRVLSNLNLPGCDGGSLYVN 6324
TR|A0A0K1YZY7|A0A0K1YZY7_CVHSA      IEELFYSYATHHDKFTDGVCLFWNCNVDRYPANAIVCRFDTRVLSNLNLPGCDGGSLYVN 6324
SP|P0C6W2|R1AB_BCHK3                IEELFYSYATHHDKFTDGVCLFWNCNVDRYPANAIVCRFDTRVLSNLNLPGCDGGSLYVN 6318
SP|P0C6W6|R1AB_BCRP3                IEELFYSYATHHDKFTDGVCLFWNCNVDRYPANAIVCRFDTRVLSNLNLPGCDGGSLYVN 6322
SP|P0C6V9|R1AB_BC279                IEELFYSYATHHDKFTDGVCLFWNCNVDRYPANAIVCRFDTRVLSNLNLPGCDGGSLYVN 6330
TR|A0A0U1WHI4|A0A0U1WHI4_CVHSA      IEELFYSYATHHDKFTDGVCLFWNCNVDRYPANAIVCRFDTRVLSNLNLPGCDGGSLYVN 6319
TR|A0A0U1WHG0|A0A0U1WHG0_CVHSA      IEELFYSYATHHDKFTDGVCLFWNCNVDRYPANAIVCRFDTRVLSNLNLPGCDGGSLYVN 6319
TR|A0A166ZL34|A0A166ZL34_9NIDO      IEELFYSYATHHDKFTDGVCLFWNCNVDRYPANAIVCRFDTRVLSNLNLPGCDGGSLYVN 6126
TR|R9QTB2|R9QTB2_CVHSA              ------------------------------------------------------------
TR|R9QTH2|R9QTH2_CVHSA              ------------------------------------------------------------
SP|P0C6U8|R1A_CVHSA                 ------------------------------------------------------------
TR|Q6JH47|Q6JH47_CVHSA              ------------------------------------------------------------
TR|Q692E5|Q692E5_CVHSA              ------------------------------------------------------------
SP|P0C6F8|R1A_BCHK3                 ------------------------------------------------------------
TR|A0A0K1Z0N1|A0A0K1Z0N1_CVHSA      ------------------------------------------------------------
SP|P0C6F5|R1A_BC279                 ------------------------------------------------------------
SP|P0C6T7|R1A_BCRP3                 ------------------------------------------------------------

QHN73794                            KHAFHTPAFDKSAFVNLKQLPFFYYSDSPCESHGKQVVSDIDYVPLKSATCITRCNLGGA 6407
SP|P0C6X7|R1AB_CVHSA                KHAFHTPAFDKSAFTNLKQLPFFYYSDSPCESHGKQVVSDIDYVPLKSATCITRCNLGGA 6384
TR|Q6UZF5|Q6UZF5_CVHSA              KHAFHTPAFDKSAFTNLKQLPFFYYSDSPCESHGKQVVSDIDYVPLKSATCITRCNLGGA 6384
TR|Q6UZF1|Q6UZF1_CVHSA              KHAFHTPAFDKSAFTNLKQLPFFYYSDSPCESHGKQVVSDIDYVPLKSATCITRCNLGGA 6384
TR|Q6JH48|Q6JH48_CVHSA              KHAFHTPAFDKSAFTNLKQLPFFYYSDSPCESHGKQVVSDIDYVPLKSATCITRCNLGGA 6384
TR|Q692E6|Q692E6_CVHSA              KHAFHTPAFDKSAFTNLKQLPFFYYSDSPCESHGKQVVSDIDYVPLKSATCITRCNLGGA 6384
TR|A0A0K1YZY7|A0A0K1YZY7_CVHSA      KHAFHTPAFDKSAFTNLKQLPFFYYSDSPCESHGKQVVSDIDYVPLKSATCITRCNLGGA 6384
SP|P0C6W2|R1AB_BCHK3                KHAFHTPAFDKSAFTHLKQLPFFYYSDSPCESHGKQVVSDIDYVPLKSATCITRCNLGGA 6378
SP|P0C6W6|R1AB_BCRP3                KHAFHTPAFDKSAFTNLKQLPFFYYSDSPCESHGKQVVSDIDYVPLKSATCITRCNLGGA 6382
SP|P0C6V9|R1AB_BC279                KHAFHTPAFDKSAFTYLKQLPFFYYSDSPCESHGKQVVSDIDYVPLKSATCITRCNLGGA 6390
TR|A0A0U1WHI4|A0A0U1WHI4_CVHSA      KHAFHTPAFDKSAFSNLKQLPFFYYSDSPCESHGKQVVSDIDYVPLKSATCITRCNLGGA 6379
TR|A0A0U1WHG0|A0A0U1WHG0_CVHSA      KHAFHTPAFDKSAFTHLKQLPFFYYSDSPCESHGKQVVSDIDYVPLKSATCITRCNLGGA 6379
TR|A0A166ZL34|A0A166ZL34_9NIDO      KHAFYTPAFDKSAFTHLKQLPFFYYSDSPCESHGKQVVSDIDYVPLKSATCITRCNLGGA 6186
TR|R9QTB2|R9QTB2_CVHSA              ------------------------------------------------------------
TR|R9QTH2|R9QTH2_CVHSA              ------------------------------------------------------------
SP|P0C6U8|R1A_CVHSA                 ------------------------------------------------------------
TR|Q6JH47|Q6JH47_CVHSA              ------------------------------------------------------------
TR|Q692E5|Q692E5_CVHSA              ------------------------------------------------------------
SP|P0C6F8|R1A_BCHK3                 ------------------------------------------------------------
TR|A0A0K1Z0N1|A0A0K1Z0N1_CVHSA      ------------------------------------------------------------
SP|P0C6F5|R1A_BC279                 ------------------------------------------------------------
SP|P0C6T7|R1A_BCRP3                 ------------------------------------------------------------
```

```
QHN73794                            VCRHHANEYRLYLDAYNMMISAGFSLWVYKQFDTYNLWNTFTRLQSLENVAFNVVNKGHF 6467
SP|P0C6X7|R1AB_CVHSA                VCRHHANEYRQYLDAYNMMISAGFSLWIYKQFDTYNLWNTFTRLQSLENVAYNVVNKGHF 6444
TR|Q6UZF5|Q6UZF5_CVHSA              VCRHHANEYRQYLDAYNMMISAGFSLWIYKQFDTYNLWNTFTRLQSLENVAYNVVNKGHF 6444
TR|Q6UZF1|Q6UZF1_CVHSA              VCRHHANEYRQYLDAYNMMISAGFSLWIYKQFDTYNLWNTFTRLQSLENVAYNVVNKGHF 6444
TR|Q6JH48|Q6JH48_CVHSA              VCRHHANEYRQYLDAYNMMISAGFSLWIYKQFDTYNLWNTFTRLQSLENVAYNVVNKGHF 6444
TR|Q692E6|Q692E6_CVHSA              VCRHHANEYRQYLDAYNMMISAGFSLWIYKQFDTYNLWNTFTRLQSLENVAYNVVNKGHF 6444
TR|A0A0K1YZY7|A0A0K1YZY7_CVHSA      VCRHHANEYRQYLDAYNMMISAGFSLWIYKQFDTYNLWNTFTRLQSLENVAYNVVNKGHF 6444
SP|P0C6W2|R1AB_BCHK3                VCRHHANEYRQYLDAYNMMISAGFSLWIYKQFDTYNLWNTFTKLQSLENVAYNVVNKGHF 6438
SP|P0C6W6|R1AB_BCRP3                VCRHHANEYRQYLDAYNMMISAGFSLWIYKQFDTYNLWNTFTRLQSLENVAYNVVNKGHF 6442
SP|P0C6V9|R1AB_BC279                VCRRHANEYRQYLDAYNMMISAGFSLWIYKQFDTYNLWNTFTRLQSLENVAYNVVNKGHF 6450
TR|A0A0U1WHI4|A0A0U1WHI4_CVHSA      VCRHHANEYRQYLDAYNMMISAGFSLWIYKQFDTYNLWNTFTRLQSLENVAYNVINKGHF 6439
TR|A0A0U1WHG0|A0A0U1WHG0_CVHSA      VCRHHANEYRQYLDAYNMMISAGFSLWIYKQFDTYNLWNTFTRLQSLENVAYNVINKGHF 6439
TR|A0A166ZL34|A0A166ZL34_9NIDO      VCRHHANEYRQYLDAYNMMISAGFSLWIYKQFDTYNLWNTFTRLQSLENVAYNVINKGHF 6246
TR|R9QTB2|R9QTB2_CVHSA              ------------------------------------------------------------
TR|R9QTH2|R9QTH2_CVHSA              ------------------------------------------------------------
SP|P0C6U8|R1A_CVHSA                 ------------------------------------------------------------
TR|Q6JH47|Q6JH47_CVHSA              ------------------------------------------------------------
TR|Q692E5|Q692E5_CVHSA              ------------------------------------------------------------
SP|P0C6F8|R1A_BCHK3                 ------------------------------------------------------------
TR|A0A0K1Z0N1|A0A0K1Z0N1_CVHSA      ------------------------------------------------------------
SP|P0C6F5|R1A_BC279                 ------------------------------------------------------------
SP|P0C6T7|R1A_BCRP3                 ------------------------------------------------------------
```
