# Supplementary material for: Structural Genomics of SARS-CoV-2 Indicates Evolutionary Conserved Functional Regions of Viral Proteins
Source: Viruses. 2020 Mar 25;12(4):360. doi: 10.3390/v12040360 (PMC7232164; doi:10.3390/v12040360)
Supplement: Supplementary file 1 [file viruses-12-00360-s001.zip › wNsp5-nonSARS_LBS_mapped.pdf]

Supplementary File 3. Ligand binding sites extracted from non-SARS protein targets and mapped on the sequence of wNsp5 protein aligned with the closest homologs found in UniProt.

Ligand BS:

N-{4-[(1H-benzotriazol-1-ylacetyl)(thiophen-3-ylmethyl)amino]phenyl}thiophene-2-carboxamide (4YOI-4F4, HKU4)

N-[4-(acetylamino)phenyl]-2-(1H-benzotriazol-1-yl)-N-[(1R)-2-(tert-butylamino)-2-oxo-1-(thiophen-3-yl)ethyl]acetamide (4YOG-4F5, HKU4)

ADENOSINE-5-DIPHOSPHORIBOSE (4MEA-APR, HKU4)

N-{4-[(1H-benzotriazol-1-ylacetyl)(thiophen-3-ylmethyl)amino]phenyl}benzamide (4YOJ-RFM, HKU4)

(1R,2S)-2-{[N-({[4-benzyl-1-(tert-butoxycarbonyl)piperidin-4-yl]oxy}carbonyl)-L-leucyl]amino}-1-hydroxy-3-[(3S)-2-oxopyrrolidin-3-yl]propane-1-sulfonic acid (5WKL-AVY, MERS)

(1S,2S)-2-[(N-{[2-(3-chlorophenyl)ethoxy]carbonyl}-L-leucyl)amino]-1-hydroxy-3-[(3S)-2-oxopyrrolidin-3-yl]propane-1-sulfonic acid (5WKK-AW4, MERS)

(1R,2S)-2-({N-[(benzyloxy)carbonyl]-L-leucyl}amino)-1-hydroxy-3-[(3S)-2-oxopyrrolidin-3-yl]propane-1-sulfonic acid (5WKJ-B1S, MERS)

(1R,2S)-2-[(N-{[2-(3-chlorophenyl)ethoxy]carbonyl}-L-leucyl)amino]-1-hydroxy-3-[(3S)-2-oxopyrrolidin-3-yl]propane-1-sulfonic acid (5WKK-B3G, MERS)

(1S,2S)-2-{[N-({[4-benzyl-1-(tert-butoxycarbonyl)piperidin-4-yl]oxy}carbonyl)-L-leucyl]amino}-1-hydroxy-3-[(3S)-2-oxopyrrolidin-3-yl]propane-1-sulfonic acid (5WKL-B3J, MERS)

(1R,2S)-2-{[N-({[1-(tert-butoxycarbonyl)-4-ethylpiperidin-4-yl]oxy}carbonyl)-L-leucyl]amino}-1-hydroxy-3-[(3S)-2-oxopyrrolidin-3-yl]propane-1-sulfonic acid (5WKM-B6Y, MERS)

(1S,2S)-2-({N-[(benzyloxy)carbonyl]-L-leucyl}amino)-1-hydroxy-3-[(3S)-2-oxopyrrolidin-3-yl]propane-1-sulfonic acid (5WKJ-K36, MERS)

(1S,2S)-2-{[N-({[1-(tert-butoxycarbonyl)-4-ethylpiperidin-4-yl]oxy}carbonyl)-L-leucyl]amino}-1-hydroxy-3-[(3S)-2-oxopyrrolidin-3-yl]propane-1-sulfonic acid (5WKM-N02, MERS)

(S)-N-benzyl-3-((S)-2-cinnamamido-3-phenylpropanamido)-2-oxo-4-((S)-2-oxopyrrolidin-3-yl)butanamide (6FV2-D03, NL63)

(2~{S})-4-methyl-~{N}-[(2~{S},3~{R})-3-oxidanyl-4-oxidanylidene-1-[(3~{S})-2-oxidanylidenepyrrolidin-3-yl]-4-[(phenylmethyl)amino]butan-2-yl]-2-[[(~{E})-3-phenylprop-2-enoyl]amino]pentanamide (6FV1-E8E, NL63)

```
QHN73794                          EAACCHLAKALNDFSNSGSDVLYQPPQTSITSAVLQSGFRKMAFPSGKVEGCMVQVTCGT 3287
SP|P0C6X7|R1AB_CVHSA              EAACCHLAKALNDFSNSGADVLYQPPQTSITSAVLQSGFRKMAFPSGKVEGCMVQVTCGT 3264
TR|Q6UZF5|Q6UZF5_CVHSA            EAACCHLAKALNDFSNSGADVLYQPPQTSITSAVLQSGFRKMAFPSGKVEGCMVQVTCGT 3264
TR|Q6UZF1|Q6UZF1_CVHSA            EAACCHLAKALNDFSNSGADVLYQPPQTSITSAVLQSGFRKMAFPSGKVEGCMVQVTCGT 3264
TR|Q6JH48|Q6JH48_CVHSA            EAACCHLAKALNDFSNSGADVLYQPPQTSITSAVLQSGFRKMAFPSGKVEGCMVQVTCGT 3264
TR|Q692E6|Q692E6_CVHSA            EAACCHLAKALNDFSNSGADVLYQPPQTSITSAVLQSGFRKMAFPSGKVEGCMVQVTCGT 3264
TR|A0A0K1YZY7|A0A0K1YZY7_CVHSA    EAACCHLAKALNDFSNSGSDVLYQPPQTSITSAVLQSGFRKMAFPSGKVEGCMVQVTCGT 3264
SP|P0C6W2|R1AB_BCHK3              EAACCHLAKALNDFSNSGADVLYQPPQTSITSAVLQSGFRKMAFPSGKVEGCMVQVTCGT 3258
SP|P0C6W6|R1AB_BCRP3              EAACCHLAKALNDFSNSGADVLYQPPQTSITSAVLQSGFRKMAFPSGKVEGCMVQVTCGT 3262
SP|P0C6V9|R1AB_BC279              EAACCHLAKALNDFSNSGADVLYQPPQTSITSAVLQSGFRKMAFPSGKVEGCMVQVTCGT 3270
TR|A0A0U1WHI4|A0A0U1WHI4_CVHSA    EAACCHLAKALNDFSNSGADVLYQPPQTSITSAVLQSGFRRMAFPSGKVEGCMVQVTCGT 3259
TR|A0A0U1WHG0|A0A0U1WHG0_CVHSA    EAACCHLAKALNDFSNSGADVLYQPPQTSITSAVLQSGFKKVAFPSGKVEGCMVQVTCGT 3259
TR|A0A166ZL34|A0A166ZL34_9NIDO    EAACCHLAKALNDFSNSGADVLYQPPQTSITSAVLQSGFKKMAFPSGKVEGCMVQVTCGT 3066
TR|R9QTB2|R9QTB2_CVHSA            EAACCHLAKALNDFSNSGADVLYQPPQTSITSAVLQSGFRKMAFPSGKVEGCMVQVTCGT 3256
TR|R9QTH2|R9QTH2_CVHSA            EAACCHLAKALNDFSNSGSDVLYQPPQTSITSAVLQSGFRKMAFPSGKVEGCMVQVTCGT 3265
SP|P0C6U8|R1A_CVHSA               EAACCHLAKALNDFSNSGADVLYQPPQTSITSAVLQSGFRKMAFPSGKVEGCMVQVTCGT 3264
TR|Q6JH47|Q6JH47_CVHSA            EAACCHLAKALNDFSNSGADVLYQPPQTSITSAVLQSGFRKMAFPSGKVEGCMVQVTCGT 3264
TR|Q692E5|Q692E5_CVHSA            EAACCHLAKALNDFSNSGADVLYQPPQTSITSAVLQSGFRKMAFPSGKVEGCMVQVTCGT 3264
SP|P0C6F8|R1A_BCHK3               EAACCHLAKALNDFSNSGADVLYQPPQTSITSAVLQSGFRKMAFPSGKVEGCMVQVTCGT 3258
TR|A0A0K1Z0N1|A0A0K1Z0N1_CVHSA    EAACCHLAKALNDFSNSGSDVLYQPPQTSITSAVLQSGFRKMAFPSGKVEGCMVQVTCGT 3264
SP|P0C6F5|R1A_BC279               EAACCHLAKALNDFSNSGADVLYQPPQTSITSAVLQSGFRKMAFPSGKVEGCMVQVTCGT 3270
SP|P0C6T7|R1A_BCRP3               EAACCHLAKALNDFSNSGADVLYQPPQTSITSAVLQSGFRKMAFPSGKVEGCMVQVTCGT 3262
                                  ******************:********************:::******************
```

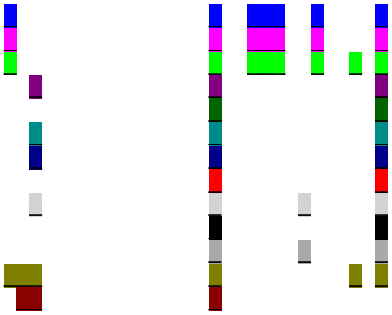

```
QHN73794                        TTLNGLWLDDVVYCPRHVICTSEDMLNPNYEDLLIRKSNHNFLVQAGNVQLRVIGHSMQN 3347
SP|P0C6X7|R1AB_CVHSA            TTLNGLWLDDTVYCPRHVICTAEDMLNPNYEDLLIRKSNHSFLVQAGNVQLRVIGHSMQN 3324
TR|Q6UZF5|Q6UZF5_CVHSA          TTLNGLWLDDTVYCPRHVICTAEDMLNPNYEDLLIRKSNHSFLVQAGNVQLRVIGHSMQN 3324
TR|Q6UZF1|Q6UZF1_CVHSA          TTLNGLWLDDTVYCPRHVICTAEDMLNPNYEDLLIRKSNHSFLVQAGNVQLRVIGHSMQN 3324
TR|Q6JH48|Q6JH48_CVHSA          TTLNGLWLDDTVYCPRHVICTAEDMLNPNYEDLLIRKANHSFLVQAGNVQLRVIGHSMQN 3324
TR|Q692E6|Q692E6_CVHSA          TTLNGLWLDDTVYCPRHVICTAEDMLNPNYEDLLIRKSNHSFLVQAGNVQLRVIGHSMQN 3324
TR|A0A0K1YZY7|A0A0K1YZY7_CVHSA  TTLNGLWLDDTVYCPRHVICTAEDMLNPNYEDLLIRKSNHSFLVQAGNVQLRVIGHSMQN 3324
SP|P0C6W2|R1AB_BCHK3            TTLNGLWLDDTVYCPRHVVCTAEDMLNPNYDDLLIRKSNHSFLVQAGNVQLRVIGHSMQN 3318
SP|P0C6W6|R1AB_BCRP3            TTLNGLWLDDTVYCPRHVICTAEDMLNPNYEDLLIRKSNHSFLVQAGNVQLRVIGHSMQN 3322
SP|P0C6V9|R1AB_BC279            TTLNGLWLDDTVYCPRHVICTAEDMLNPNYEDLLIRKSNHSFLVQAGNVQLRVIGHSMQN 3330
TR|A0A0U1WHI4|A0A0U1WHI4_CVHSA  TTLNGLWLDDTVYCPRHVICTAEDMLNPNYEDLLIRKSNHSFLVQAGNVQLRVIGHSMQN 3319
TR|A0A0U1WHG0|A0A0U1WHG0_CVHSA  TTLNGLWLDDTVYCPRHVVCTVEDMLNPNYEDLLIRKSNHSFLVQTGNVQLRVIGHSMQN 3319
TR|A0A166ZL34|A0A166ZL34_9NIDO  TTLNGLWLDDTVYCPRHVVCTVEDMLNPNYEDLLIRKSNHSFLVQTGNVQLRVIGHSMQN 3126
TR|R9QTB2|R9QTB2_CVHSA          TTLNGLWLDDTVYCPRHVICTAEDMLNPNYEDLLIRKSNHSFLVQAGNVQLRVIGHSMQN 3316
TR|R9QTH2|R9QTH2_CVHSA          TTLNGLWLDDTVYCPRHVICTAEDMLNPNYEDLLIRKSNHSFLVQAGNVQLRVIGHSMQN 3325
SP|P0C6U8|R1A_CVHSA             TTLNGLWLDDTVYCPRHVICTAEDMLNPNYEDLLIRKSNHSFLVQAGNVQLRVIGHSMQN 3324
TR|Q6JH47|Q6JH47_CVHSA          TTLNGLWLDDTVYCPRHVICTAEDMLNPNYEDLLIRKANHSFLVQAGNVQLRVIGHSMQN 3324
TR|Q692E5|Q692E5_CVHSA          TTLNGLWLDDTVYCPRHVICTAEDMLNPNYEDLLIRKSNHSFLVQAGNVQLRVIGHSMQN 3324
SP|P0C6F8|R1A_BCHK3             TTLNGLWLDDTVYCPRHVVCTAEDMLNPNYDDLLIRKSNHSFLVQAGNVQLRVIGHSMQN 3318
TR|A0A0K1Z0N1|A0A0K1Z0N1_CVHSA  TTLNGLWLDDTVYCPRHVICTAEDMLNPNYEDLLIRKSNHSFLVQAGNVQLRVIGHSMQN 3324
SP|P0C6F5|R1A_BC279             TTLNGLWLDDTVYCPRHVICTAEDMLNPNYEDLLIRKSNHSFLVQAGNVQLRVIGHSMQN 3330
SP|P0C6T7|R1A_BCRP3             TTLNGLWLDDTVYCPRHVICTAEDMLNPNYEDLLIRKSNHSFLVQAGNVQLRVIGHSMQN 3322
                                **********.*******:** ********:******:**.****:**************
```

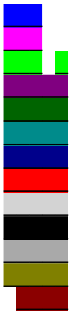

```
QHN73794                        CVLKLKVDTANPKTPKYKFVRIQPGQTFSVLACYNGSPSGVYQCAMRPNFTIKGSFLNGS 3407
SP|P0C6X7|R1AB_CVHSA            CLLRLKVDTSNPKTPKYKFVRIQPGQTFSVLACYNGSPSGVYQCAMRPNHTIKGSFLNGS 3384
TR|Q6UZF5|Q6UZF5_CVHSA          CLLRLKVDTSNPKTPKYKFVRIQPGQTFSVLACYNGSPSGVYQCAMRPNHTIKGSFLNGS 3384
TR|Q6UZF1|Q6UZF1_CVHSA          CLLRLKVDTSNPKTPKYKFVRIQPGQTFSVLACYNGSPSGVYQCAMRPNHTIKGSFLNGS 3384
TR|Q6JH48|Q6JH48_CVHSA          CLLRLKVDTSNPKTPKYKFVRIQPGQTFSVLACYNGSPSGVYQCAMRPNHTIKGSFLNGS 3384
TR|Q692E6|Q692E6_CVHSA          CLLRLKVDTSNPKTPKYKFVRIQPGQTFSVLACYNGSPSGVYQCAMRPNHTIKGSFLNGS 3384
TR|A0A0K1YZY7|A0A0K1YZY7_CVHSA  CLLRLKVDTSNPKTPKYKFVRIQPGQTFSVLACYNGSPSGVYQCAMRPNHTIKGSFLNGS 3384
SP|P0C6W2|R1AB_BCHK3            CLLRLKVDTSNPKTPKYKFVRIQPGQTFSVLACYNGSPSGVYQCAMRPNHTIKGSFLNGS 3378
SP|P0C6W6|R1AB_BCRP3            CLLRLKVDTSNPKTPKYKFVRIQPGQTFSVLACYNGSPSGVYQCAMRPNHTIKGSFLNGS 3382
SP|P0C6V9|R1AB_BC279            CLLRLKVDTSNPKTPKYKFVRIQPGQTFSVLACYNGSPSGVYQCAMRPNYTIKGSFLNGS 3390
TR|A0A0U1WHI4|A0A0U1WHI4_CVHSA  CLLRLKVDTSNPKTPKYKFVRIQPGQTFSVLACYNGSPSGVYQCAMRPNYTIKGSFLNGS 3379
TR|A0A0U1WHG0|A0A0U1WHG0_CVHSA  CLLRLKVDTSNPKTPKYKFVRIQPGQTFSVLACYNGSPSGVYQCAMRPNYTIKGSFLNGS 3379
TR|A0A166ZL34|A0A166ZL34_9NIDO  CLLRLKVDTSNPKTPKYKFVRIQPGQTFSVLACYNGSPSGVYQCAMRPNYTIKGSFLNGS 3186
TR|R9QTB2|R9QTB2_CVHSA          CLLRLKVDTSNPKTPKYKFVRIQPGQTFSVLACYNGSPSGVYQCAMRPNHTIKGSFLNGS 3376
TR|R9QTH2|R9QTH2_CVHSA          CLLRLKVDTSNPKTPKYKFVRIQPGQTFSVLACYNGSPSGVYQCAMRPNHTIKGSFLNGS 3385
SP|P0C6U8|R1A_CVHSA             CLLRLKVDTSNPKTPKYKFVRIQPGQTFSVLACYNGSPSGVYQCAMRPNHTIKGSFLNGS 3384
TR|Q6JH47|Q6JH47_CVHSA          CLLRLKVDTSNPKTPKYKFVRIQPGQTFSVLACYNGSPSGVYQCAMRPNHTIKGSFLNGS 3384
TR|Q692E5|Q692E5_CVHSA          CLLRLKVDTSNPKTPKYKFVRIQPGQTFSVLACYNGSPSGVYQCAMRPNHTIKGSFLNGS 3384
SP|P0C6F8|R1A_BCHK3             CLLRLKVDTSNPKTPKYKFVRIQPGQTFSVLACYNGSPSGVYQCAMRPNHTIKGSFLNGS 3378
TR|A0A0K1Z0N1|A0A0K1Z0N1_CVHSA  CLLRLKVDTSNPKTPKYKFVRIQPGQTFSVLACYNGSPSGVYQCAMRPNHTIKGSFLNGS 3384
SP|P0C6F5|R1A_BC279             CLLRLKVDTSNPKTPKYKFVRIQPGQTFSVLACYNGSPSGVYQCAMRPNYTIKGSFLNGS 3390
SP|P0C6T7|R1A_BCRP3             CLLRLKVDTSNPKTPKYKFVRIQPGQTFSVLACYNGSPSGVYQCAMRPNHTIKGSFLNGS 3382
                                *:*:*****:****************************************.*********
```

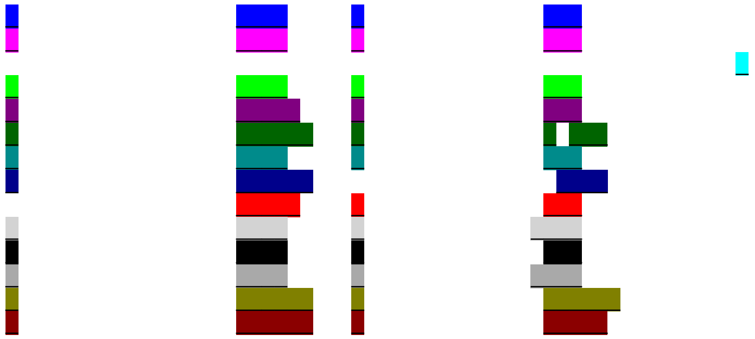

```
QHN73794                          CGSVGFNIDYDCVSFCYMHHMELPTGVHAGTDLEGNFYGPFVDRQTAQAAGTDTTITVNV 3467
SP|P0C6X7|R1AB_CVHSA              CGSVGFNIDYDCVSFCYMHHMELPTGVHAGTDLEGKFYGPFVDRQTAQAAGTDTTITLNV 3444
TR|Q6UZF5|Q6UZF5_CVHSA            CGSVGFNIDYDCVSFCYMHHMELPTGVHAGTDLEGKFYGPFVDRQTAQAAGTDTTITLNV 3444
TR|Q6UZF1|Q6UZF1_CVHSA            CGSVGFNIDYDCVSFCYMHHMELPTGVHAGTDLEGKFYGPFVDRQTAQAAGTDTTITLNV 3444
TR|Q6JH48|Q6JH48_CVHSA            CGSVGFNIDYDCVSFCYMHHMELPTGVHAGTDLEGKFYGPFVDRQTAQAAGTDTTITLNV 3444
TR|Q692E6|Q692E6_CVHSA            CGSVGFNIDYDCVSFCYMHHMELPTGVHAGTDLEGKFYGPFVDRQTAQAAGTDTTITLNV 3444
TR|A0A0K1YZY7|A0A0K1YZY7_CVHSA    CGSVGFNIDYDCVSFCYMHHMELPTGVHAGTDLEGKFYGPFVDRQTAQAAGTDTTITLNV 3444
SP|P0C6W2|R1AB_BCHK3              CGSVGFNIDYDCVSFCYMHHMELPTGVHAGTDLEGKFYGPFVDRQTAQAAGTDTTITLNV 3438
SP|P0C6W6|R1AB_BCRP3              CGSVGFNIDYDCVSFCYMHHMELPTEVHAGTDLEGKFYGPFVDRQTAQAAGTDTTITLNV 3442
SP|P0C6V9|R1AB_BC279              CGSVGFNIDYDCVSFCYMHHMELPTGVHAGTDLEGKFYGPFVDRQTAQAAGTDTTITLNV 3450
TR|A0A0U1WHI4|A0A0U1WHI4_CVHSA    CGSVGFNIDYDCVSFCYMHHMELPTGVHAGTDLEGKFYGPFVDRQTAQAAGTDTTITLNV 3439
TR|A0A0U1WHG0|A0A0U1WHG0_CVHSA    CGSVGFNIDYDCVSFCYMHHMELPTGVHAGTDLEGKFYGPFVDRQTAQAAGTDTTITLNV 3439
TR|A0A166ZL34|A0A166ZL34_9NIDO    CGSVGFNIDYDCVSFCYMHHMELPTGVHAGTDLEGKFYGPFVDRQTAQAAGTDTAITLNV 3246
TR|R9QTB2|R9QTB2_CVHSA            CGSVGFNIDYDCVSFCYMHHMELPTGVHAGTDLEGKFYGPFVDRQTAQAAGTDTTITLNV 3436
TR|R9QTH2|R9QTH2_CVHSA            CGSVGFNIDYDCVSFCYMHHMELPTGVHAGTDLEGKFYGPFVDRQTAQAAGTDTTITLNV 3445
SP|P0C6U8|R1A_CVHSA               CGSVGFNIDYDCVSFCYMHHMELPTGVHAGTDLEGKFYGPFVDRQTAQAAGTDTTITLNV 3444
TR|Q6JH47|Q6JH47_CVHSA            CGSVGFNIDYDCVSFCYMHHMELPTGVHAGTDLEGKFYGPFVDRQTAQAAGTDTTITLNV 3444
TR|Q692E5|Q692E5_CVHSA            CGSVGFNIDYDCVSFCYMHHMELPTGVHAGTDLEGKFYGPFVDRQTAQAAGTDTTITLNV 3444
SP|P0C6F8|R1A_BCHK3               CGSVGFNIDYDCVSFCYMHHMELPTGVHAGTDLEGKFYGPFVDRQTAQAAGTDTTITLNV 3438
TR|A0A0K1Z0N1|A0A0K1Z0N1_CVHSA    CGSVGFNIDYDCVSFCYMHHMELPTGVHAGTDLEGKFYGPFVDRQTAQAAGTDTTITLNV 3444
SP|P0C6F5|R1A_BC279               CGSVGFNIDYDCVSFCYMHHMELPTGVHAGTDLEGKFYGPFVDRQTAQAAGTDTTITLNV 3450
SP|P0C6T7|R1A_BCRP3               CGSVGFNIDYDCVSFCYMHHMELPTEVHAGTDLEGKFYGPFVDRQTAQAAGTDTTITLNV 3442
                                  ************************* *********:******************:**:**

QHN73794                          LAWLYAAVINGDRWFLNRFTTTLNDFNLVAMKYNYEPLTQDHVDILGPLSAQTGIAVLDM 3527
SP|P0C6X7|R1AB_CVHSA              LAWLYAAVINGDRWFLNRFTTTLNDFNLVAMKYNYEPLTQDHVDILGPLSAQTGIAVLDM 3504
TR|Q6UZF5|Q6UZF5_CVHSA            LAWLYAAVINGDRWFLNRFTTTLNDFNLVAMKYNYEPLTQDHVDILGPLSAQTGIAVLDM 3504
TR|Q6UZF1|Q6UZF1_CVHSA            LAWLYAAVINGDRWFLNRFTTTLNDFNLVAMKYNYEPLTQDHVDILGPLSAQTGIAVLDM 3504
TR|Q6JH48|Q6JH48_CVHSA            LAWLYAAVINGDRWFLNRFTTTLNDFNLVAMKYNYEPLTQDHVDILGPLSAQTGIAVLDM 3504
TR|Q692E6|Q692E6_CVHSA            LAWLYAAVINGDRWFLNRFTTTLNDFNLVAMKYNYEPLTQDHVDILGPLSAQTGIAVLDM 3504
TR|A0A0K1YZY7|A0A0K1YZY7_CVHSA    LAWLYAAVINGDRWFLNRFTTTLNDFNLVAMKYNYEPLTQDHVDVLGPLSAQTGIAVLDM 3504
SP|P0C6W2|R1AB_BCHK3              LAWLYAAVINGDRWFLNRFTTTLNDFNLVAMKYNYEPLTQDHVDILGPLSAQTGIAVLDM 3498
SP|P0C6W6|R1AB_BCRP3              LAWLYAAVINGDRWFLNRFTTTLNDFNLVAMKYNYEPLTQDHVDILGPLSAQTGIAVLDM 3502
SP|P0C6V9|R1AB_BC279              LAWLYAAVINGDRWFLNRFTTTLNDFNLVAMKYNYEPLTQDHVDILGPLSAQTGIAVLDM 3510
TR|A0A0U1WHI4|A0A0U1WHI4_CVHSA    LAWLYAAVINGDRWFLNRFTTTLNDFNLVAMKYNYEPLTQDHVDILGPLSAQTGIAVLDM 3499
TR|A0A0U1WHG0|A0A0U1WHG0_CVHSA    LAWLYAAVINGDRWFLNRFTTTLNDFNLVAMKYNYEPLTQDHVDILGPLSAQTGIAVLDM 3499
TR|A0A166ZL34|A0A166ZL34_9NIDO    LAWLYAAVINGDRWFLNRFTTTLNDFNLVAMKYNYEPLTQDHVDILGPLSAQTGIAVLDM 3306
TR|R9QTB2|R9QTB2_CVHSA            LAWLYAAVINGDRWFLNRFTTTLNDFNLVAMKYNYEPLTQDHVDILGPLSAQTGIAVLDM 3496
TR|R9QTH2|R9QTH2_CVHSA            LAWLYAAVINGDRWFLNRFTTTLNDFNLVAMKYNYEPLTQDHVDILGPLSAQTGIAVLDM 3505
SP|P0C6U8|R1A_CVHSA               LAWLYAAVINGDRWFLNRFTTTLNDFNLVAMKYNYEPLTQDHVDILGPLSAQTGIAVLDM 3504
TR|Q6JH47|Q6JH47_CVHSA            LAWLYAAVINGDRWFLNRFTTTLNDFNLVAMKYNYEPLTQDHVDILGPLSAQTGIAVLDM 3504
TR|Q692E5|Q692E5_CVHSA            LAWLYAAVINGDRWFLNRFTTTLNDFNLVAMKYNYEPLTQDHVDILGPLSAQTGIAVLDM 3504
SP|P0C6F8|R1A_BCHK3               LAWLYAAVINGDRWFLNRFTTTLNDFNLVAMKYNYEPLTQDHVDILGPLSAQTGIAVLDM 3498
TR|A0A0K1Z0N1|A0A0K1Z0N1_CVHSA    LAWLYAAVINGDRWFLNRFTTTLNDFNLVAMKYNYEPLTQDHVDVLGPLSAQTGIAVLDM 3504
SP|P0C6F5|R1A_BC279               LAWLYAAVINGDRWFLNRFTTTLNDFNLVAMKYNYEPLTQDHVDILGPLSAQTGIAVLDM 3510
SP|P0C6T7|R1A_BCRP3               LAWLYAAVINGDRWFLNRFTTTLNDFNLVAMKYNYEPLTQDHVDILGPLSAQTGIAVLDM 3502
                                  ********************************************:***************
```

```
QHN73794                       CASLKELLQNGMNGRTILGSALLEDEFTPFDVVRQCSGVTFQSAVKRTIKGTHHWLLLTI 3587
SP|P0C6X7|R1AB_CVHSA           CAALKELLQNGMNGRTILGSTILEDEFTPFDVVRQCSGVTFQGKFKKIVKGTHHWMLLTF 3564
TR|Q6UZF5|Q6UZF5_CVHSA         CAALKELLQNGMNGRTILGSTILEDEFTPFDVVRQCSGVTFQGKFKKIVKGTHHWMLLTF 3564
TR|Q6UZF1|Q6UZF1_CVHSA         CAALKELLQNGMNGRTILGSTILEDEFTPFDVVRQCSGVTFQGKFKKIVKGTHHWMLLTF 3564
TR|Q6JH48|Q6JH48_CVHSA         CAALKELLQNGMNGRTILGSTILEDEFTPFDVVRQCSGVTFQGKFKKIVKGTHHWMLLTF 3564
TR|Q692E6|Q692E6_CVHSA         CAALKELLQNGMNGRTILGSTILEDEFTPFDVVRQCSGVTFQGKFKKIVKGTHHWMLLTF 3564
TR|A0A0K1YZY7|A0A0K1YZY7_CVHSA CAALKELLQNGMNGRTILGSTILEDEFTPFDVVRQCSGVTFQGKFKKIVKGTHHWMLLTF 3564
SP|P0C6W2|R1AB_BCHK3           CAALKELLQNGMNGRTILGSTILEDEFTPFDVVRQCSGVTFQGKFKKIVKGTHHWMLLTF 3558
SP|P0C6W6|R1AB_BCRP3           CAALKELLQNGMNGRTILGSTILEDEFTPFDVVRQCSGVTFQGKFKRIVKGTHHWMLLTF 3562
SP|P0C6V9|R1AB_BC279           CAALKELLQNGMNGRTILGSTILEDEFTPFDVVRQCSGVTFQGKFKKIVKGTHHWMLLTF 3570
TR|A0A0U1WHI4|A0A0U1WHI4_CVHSA CAALKELLQNGMNGRTILGSTILEDEFTPFDVVRQCSGVTFQGKFKKIVKGTHHWMLLTF 3559
TR|A0A0U1WHG0|A0A0U1WHG0_CVHSA CAALKELLQNGMNGRTILGSTILEDEFTPFDVVRQCSGVTFQGKFKKIVKGTHHWMLLTF 3559
TR|A0A166ZL34|A0A166ZL34_9NIDO CAALKELLQNGMNGRTILGSTILEDEFTPFDVVRQCSGVTFQGKFKKIVKGTHHWMLLTF 3366
TR|R9QTB2|R9QTB2_CVHSA         CAALKELLQNGMNGRTILGSTILEDEFTPFDVVRQCSGVTFQGKFKKIVKGTHHWMLLTF 3556
TR|R9QTH2|R9QTH2_CVHSA         CAALKELLQNGMNGRTILGSTILEDEFTPFDVVRQCSGVTFQGKFKKIVKGTHHWVLLTF 3565
SP|P0C6U8|R1A_CVHSA            CAALKELLQNGMNGRTILGSTILEDEFTPFDVVRQCSGVTFQGKFKKIVKGTHHWMLLTF 3564
TR|Q6JH47|Q6JH47_CVHSA         CAALKELLQNGMNGRTILGSTILEDEFTPFDVVRQCSGVTFQGKFKKIVKGTHHWMLLTF 3564
TR|Q692E5|Q692E5_CVHSA         CAALKELLQNGMNGRTILGSTILEDEFTPFDVVRQCSGVTFQGKFKKIVKGTHHWMLLTF 3564
SP|P0C6F8|R1A_BCHK3            CAALKELLQNGMNGRTILGSTILEDEFTPFDVVRQCSGVTFQGKFKKIVKGTHHWMLLTF 3558
TR|A0A0K1Z0N1|A0A0K1Z0N1_CVHSA CAALKELLQNGMNGRTILGSTILEDEFTPFDVVRQCSGVTFQGKFKKIVKGTHHWMLLTF 3564
SP|P0C6F5|R1A_BC279            CAALKELLQNGMNGRTILGSTILEDEFTPFDVVRQCSGVTFQGKFKKIVKGTHHWMLLTF 3570
SP|P0C6T7|R1A_BCRP3            CAALKELLQNGMNGRTILGSTILEDEFTPFDVVRQCSGVTFQGKFKRIVKGTHHWMLLTF 3562
                               **:*****************::********************. .*: :******:***:
```
