# Supplementary material for: Structural Genomics of SARS-CoV-2 Indicates Evolutionary Conserved Functional Regions of Viral Proteins
Source: Viruses. 2020 Mar 25;12(4):360. doi: 10.3390/v12040360 (PMC7232164; doi:10.3390/v12040360)
Supplement: Supplementary file 1 [file viruses-12-00360-s001.zip › wNsp3-domain5_LBS_mapped.pdf]

Supplementary File 2. Ligand binding sites mapped on the sequence of wNsp3 protein aligned with the closest homologs found in UniProt.

5-amino-2-methyl-N-[(1R)-1-naphthalen-1-ylethyl]benzamide (3E9S-TTT, SARS)

N-[(4-fluorophenyl)methyl]-1-[(1R)-1-naphthalen-1-ylethyl]piperidine-4-carboxamide (4OVZ-P85, SARS)

N-[(3-fluorophenyl)methyl]-1-[(1R)-1-naphthalen-1-ylethyl]piperidine-4-carboxamide (4OW0-S88, SARS)

```
QHN73794                          GEVITFDNLKTLLSLREVRTIKVFTTVDNINLHTQVVDMSMTYGQQFGPTYLDGADVTKI 1607
SP|P0C6X7|R1AB_CVHSA              GEVLSLDKLKSLLSLREVKTIKVFTTVDNTNLHTQLVDMSMTYGQQFGPTYLDGADVTKI 1584
TR|Q6UZF5|Q6UZF5_CVHSA            GEVLSLDKLKSLLSLREVKTIKVFTTVDNTNLHTQLVDMSMTYGQQFGPTYLDGADVTKI 1584
TR|Q6UZF1|Q6UZF1_CVHSA            GEVLSLDKLKSLLSLREVKTIKVFTTVDNTNLHTQLVDMSMTYGQQFGPTYLDGADVTKI 1584
TR|Q6JH48|Q6JH48_CVHSA            GEVLSLDKLKSLLSLREVKTIKVFTTVDNTNLHTQLVDMSMTYGQQFGPTYLDGADVTKI 1584
TR|Q692E6|Q692E6_CVHSA            GEVLSLDKLKSLLSLREVKTIKVFTTVDNTNLHTQLVDMSMTYGQQFGPTYLDGADVTKI 1584
TR|A0A0K1YZY7|A0A0K1YZY7_CVHSA GEVQPLDKLKSLLSLREVKTIKVFTTVDNTNLHTQIVDMSMTYGQQFGPTYLDGADVTKI 1584
SP|P0C6W2|R1AB_BCHK3              GEVLPLDKLKSLLSLREVKTIKVFTTVDNTNLHTHIVDMSMTYGQQFGPTYLDGADVTKI 1578
SP|P0C6W6|R1AB_BCRP3              GEVLPLDKLKSLLSLREVKTIKVFTTVDNTNLHTQLVDMSMTYGQQLGPTYLEGADVTKI 1582
SP|P0C6V9|R1AB_BC279              GEVLLLDKLKSLLSLREVRTIKVFTTVDNTNLHTQIVDMSMTYGQQFGPTYLDGADVTKI 1590
TR|A0A0U1WHI4|A0A0U1WHI4_CVHSA GEVLPLDKLKSLLSLREVKTIKVFTTVDNTNLHTQLVDMSVTYGQHFGPTYLDGADVTKV 1579
TR|A0A0U1WHG0|A0A0U1WHG0_CVHSA GEVLPLDKLKSLLSLREVKTIKVFTTVDNTNLHTQVVDMSMTYGQHFGSTYLDGADVTKV 1579
TR|A0A166ZL34|A0A166ZL34_9NIDO GEVLPLDKLKSLLSLREVKTIKVFTTVDNTNLHTQVVDMSMTYGQHFGSTYLDGADVTKV 1579
TR|R9QTB2|R9QTB2_CVHSA            GEVLPLDKLKSLLSLREVKTIKVFTTVDNTNLHTQIVDMSMTYGQQFGSTYLDGADVTKI 1576
TR|R9QTH2|R9QTH2_CVHSA            GEVLPLDKLKSLLSLREVKTIKVFTTVDNTNLHTQLVDMSMTYGQQFGPTYLDGADVTKI 1585
SP|P0C6U8|R1A_CVHSA               GEVLSLDKLKSLLSLREVKTIKVFTTVDNTNLHTQLVDMSMTYGQQFGPTYLDGADVTKI 1584
TR|Q6JH47|Q6JH47_CVHSA            GEVLSLDKLKSLLSLREVKTIKVFTTVDNTNLHTQLVDMSMTYGQQFGPTYLDGADVTKI 1584
TR|Q692E5|Q692E5_CVHSA            GEVLSLDKLKSLLSLREVKTIKVFTTVDNTNLHTQLVDMSMTYGQQFGPTYLDGADVTKI 1584
SP|P0C6F8|R1A_BCHK3               GEVLPLDKLKSLLSLREVKTIKVFTTVDNTNLHTHIVDMSMTYGQQFGPTYLDGADVTKI 1578
TR|A0A0K1Z0N1|A0A0K1Z0N1_CVHSA GEVQPLDKLKSLLSLREVKTIKVFTTVDNTNLHTQIVDMSMTYGQQFGPTYLDGADVTKI 1584
SP|P0C6F5|R1A_BC279               GEVLLLDKLKSLLSLREVRTIKVFTTVDNTNLHTQIVDMSMTYGQQFGPTYLDGADVTKI 1590
SP|P0C6T7|R1A_BCRP3               GEVLPLDKLKSLLSLREVKTIKVFTTVDNTNLHTQLVDMSMTYGQQLGPTYLEGADVTKI 1582
                                  ***  :*:**:*******:********** ****::****:****::* ***:******:

QHN73794                          KPHNSHEGKTFYVLPNDDTLRVEAFEYYHTTDPSFLGRYMSALNHTKKWKYPQVNGLTSI 1667
SP|P0C6X7|R1AB_CVHSA              KPHVNHEGKTFFVLPSDDTLRSEAFEYYHTLDESFLGRYMSALNHTKKWKFPQVGGLTSI 1644
TR|Q6UZF5|Q6UZF5_CVHSA            KPHVNHEGKTFFVLPSDDTLRSEAFEYYHTLDESFLGRYMSALNHTKKWKFPQVGGLTSI 1644
TR|Q6UZF1|Q6UZF1_CVHSA            KPHVNHEGKTFFVLPSDDTLRSEAFEYYHTLDESFLGRYMSALNHTKKWKFPQVGGLTSI 1644
TR|Q6JH48|Q6JH48_CVHSA            KPHVNHEGKTFFVLPSDDTLRSEAFEYYHTLDESFLGRYMSALNHTKKWKFPQVGGLTSI 1644
TR|Q692E6|Q692E6_CVHSA            KPHVNHEGKTFFVLPSDDTLRSEAFEYYHTLDESFLGRYMSALNHTKKWKFPQVGGLTSI 1644
TR|A0A0K1YZY7|A0A0K1YZY7_CVHSA KPHVNHEGKTFYVLPSDDTLRSEAFEYYHTLDESFLGRYMSALNHTKKWKFPQVGGLTSI 1644
SP|P0C6W2|R1AB_BCHK3              KPHVNHEGKTFFVLPSDDTLRSEAFEYYHTIDESFLGRYMSALNHTKKWKFPQVGGLTSI 1638
SP|P0C6W6|R1AB_BCRP3              KPHVNHEGKTFFVLPSDDTLRSEAFEYYHTLDESFLGRYMSALNHTKKWKFPQVGGLTSI 1642
SP|P0C6V9|R1AB_BC279              KPHAKHEGKTFFVLPSDDTLRSEAFEYYHTLDESFLGRYMSALNHTKKWKFPQIGGLTSI 1650
TR|A0A0U1WHI4|A0A0U1WHI4_CVHSA KPHVNHEGKTFFVLPSDDTLRSEAFEYYHTLDESFLGRYMSALSHTKKWKFPQVGGLTSI 1639
TR|A0A0U1WHG0|A0A0U1WHG0_CVHSA KPHVNHEGKTFFVLPSDDTLRSEAFEYYHTLDESFLGRYMSALNHTKKWKFPQVGGLTSI 1639
TR|A0A166ZL34|A0A166ZL34_9NIDO KPHVNHEGKTFFVLPSDDTLRSEAFGYYHTLDESFLGRYMSALNHTKKWKFPQVGGLTSI 1639
TR|R9QTB2|R9QTB2_CVHSA            KPHVNHEGKTFFVLPSDDTLRSEAFEYYHTLDESFLGRYMSALSHTKKWKFPQVGGLTSI 1636
TR|R9QTH2|R9QTH2_CVHSA            KPHVNHEGKTFFVLPSDDTLRSEAFEYYHTLDESFLGRYMSALNHTKKWKFPQVGGLTSI 1645
SP|P0C6U8|R1A_CVHSA               KPHVNHEGKTFFVLPSDDTLRSEAFEYYHTLDESFLGRYMSALNHTKKWKFPQVGGLTSI 1644
TR|Q6JH47|Q6JH47_CVHSA            KPHVNHEGKTFFVLPSDDTLRSEAFEYYHTLDESFLGRYMSALNHTKKWKFPQVGGLTSI 1644
TR|Q692E5|Q692E5_CVHSA            KPHVNHEGKTFFVLPSDDTLRSEAFEYYHTLDESFLGRYMSALNHTKKWKFPQVGGLTSI 1644
SP|P0C6F8|R1A_BCHK3               KPHVNHEGKTFFVLPSDDTLRSEAFEYYHTIDESFLGRYMSALNHTKKWKFPQVGGLTSI 1638
TR|A0A0K1Z0N1|A0A0K1Z0N1_CVHSA KPHVNHEGKTFYVLPSDDTLRSEAFEYYHTLDESFLGRYMSALNHTKKWKFPQVGGLTSI 1644
SP|P0C6F5|R1A_BC279               KPHAKHEGKTFFVLPSDDTLRSEAFEYYHTLDESFLGRYMSALNHTKKWKFPQIGGLTSI 1650
SP|P0C6T7|R1A_BCRP3               KPHVNHEGKTFFVLPSDDTLRSEAFEYYHTLDESFLGRYMSALNHTKKWKFPQVGGLTSI 1642
                                  *** .******:***.***** *** **** * **********.******:**:.*****

QHN73794                          KWADNNCYLATALLTLQQIELKFNPPALQDAYYRARAGEAANFCALILAYCNKTVGELGD 1727
SP|P0C6X7|R1AB_CVHSA              KWADNNCYLSSVLLALQQLEVKFNAPALQEAYYRARAGDAANFCALILAYSNKTVGELGD 1704
TR|Q6UZF5|Q6UZF5_CVHSA            KWADNNCYLSSVLLALQQLEVKFNAPALQEAYYRARAGDAANFCALILAYSNKTVGELGD 1704
TR|Q6UZF1|Q6UZF1_CVHSA            KWADNNCYLSSVLLALQQLEVKFNAPALQEAYYRARAGDAANFCALILAYSNKTVGELGD 1704
TR|Q6JH48|Q6JH48_CVHSA            KWADNNCYLSSVLLALQQLEVKFNAPALQEAYYRARAGDAANFCALILAYSNKTVGELGD 1704
TR|Q692E6|Q692E6_CVHSA            KWADNNCYLSSVLLALQQLEVKFNAPALQEAYYRARAGDAANFCALILAYSNKTVGELGD 1704
TR|A0A0K1YZY7|A0A0K1YZY7_CVHSA KWADNNCYLSSVLLALQQIEVKFNAPALQEAYYRARAGDAANFCALILAYSNKTVGELGD 1704
SP|P0C6W2|R1AB_BCHK3              KWADNNCYLSSVLLALQQVEVKFNAPALQEAYYRARAGDAANFCALILAYSNKTVGELGD 1698
SP|P0C6W6|R1AB_BCRP3              KWADNNCYLSSVLLALQQIEVKFNAPALQEAYYRARAGDAANFCALILAYSNKTVGELGD 1702
SP|P0C6V9|R1AB_BC279              KWADNNCYLSSVLLALQQIEVKFNAPALQEAYYRARAGDAANFCALILAYSNRTVGELGD 1710
TR|A0A0U1WHI4|A0A0U1WHI4_CVHSA KWADNNCYLSSVLLALQQIEVKFNAPALQEAYYRARAGDAANFCALILAYSNKTVGELGD 1699
TR|A0A0U1WHG0|A0A0U1WHG0_CVHSA KWADNNCYLSSVLLALQQIEVKFNAPALQEAYYRARAGEAANFCALILAYSNKTVGELGD 1699
TR|A0A166ZL34|A0A166ZL34_9NIDO KWADNNCYLSSVLLALQQIEVKFNAPALQEAYYRARAGDAANFCALTLAYSNKTVGDLGD 1699
TR|R9QTB2|R9QTB2_CVHSA            KWADNNCYLSSVLLALQQIEVKFNAPALQEAYYRARAGDAANFCALILAYSYKTVGELGD 1696
TR|R9QTH2|R9QTH2_CVHSA            KWADNNCYLSSVLLALQQIEVKFNAPALQEAYYRARAGDAANFCALILAYSNKTVGELGD 1705
SP|P0C6U8|R1A_CVHSA               KWADNNCYLSSVLLALQQLEVKFNAPALQEAYYRARAGDAANFCALILAYSNKTVGELGD 1704
TR|Q6JH47|Q6JH47_CVHSA            KWADNNCYLSSVLLALQQLEVKFNAPALQEAYYRARAGDAANFCALILAYSNKTVGELGD 1704
TR|Q692E5|Q692E5_CVHSA            KWADNNCYLSSVLLALQQLEVKFNAPALQEAYYRARAGDAANFCALILAYSNKTVGELGD 1704
SP|P0C6F8|R1A_BCHK3               KWADNNCYLSSVLLALQQVEVKFNAPALQEAYYRARAGDAANFCALILAYSNKTVGELGD 1698
TR|A0A0K1Z0N1|A0A0K1Z0N1_CVHSA KWADNNCYLSSVLLALQQIEVKFNAPALQEAYYRARAGDAANFCALILAYSNKTVGELGD 1704
SP|P0C6F5|R1A_BC279               KWADNNCYLSSVLLALQQIEVKFNAPALQEAYYRARAGDAANFCALILAYSNRTVGELGD 1710
SP|P0C6T7|R1A_BCRP3               KWADNNCYLSSVLLALQQIEVKFNAPALQEAYYRARAGDAANFCALILAYSNKTVGELGD 1702
                                  *********::.**:***:*:*** ****:********:******* ***. :***:***
```

```
QHN73794                       VRETMSYLFQHANLDSCKRVLNVVCKTCGQQQTTLKGVEAVMYMGTLSYEQFKKGVQIPC 1787
SP|P0C6X7|R1AB_CVHSA           VRETMTHLLQHANLESAKRVLNVVCKHCGQKTTTLTGVEAVMYMGTLSYDNLKTGVSIPC 1764
TR|Q6UZF5|Q6UZF5_CVHSA         VRETMTHLLQHANLESAKRVLNVVCKHCGQKTTTLTGVEAVMYMGTLSYDNLKTGVSIPC 1764
TR|Q6UZF1|Q6UZF1_CVHSA         VRETMTHLLQHANLESAKRVLNVVCKHCGQKTTTLTGVEAVMYMGTLSYDNLKTGVSIPC 1764
TR|Q6JH48|Q6JH48_CVHSA         VRETMTHLLQHANLESAKRVLNVVCKHCGQKTTTLTGVEAVMYMGTLSYDNLKTGVSIPC 1764
TR|Q692E6|Q692E6_CVHSA         VRETMTHLLQHANLESAKRVLNVVCKHCGQKTTTLTGVEAVMYMGTLSYDNLKTGVSIPC 1764
TR|A0A0K1YZY7|A0A0K1YZY7_CVHSA VRETMTHLLQHANLESAKRVLNVVCKHCGQKTTTLTGVEAVMYMGTLSYDNLKTGVSIPC 1764
SP|P0C6W2|R1AB_BCHK3           VRETMTHLLQHANLESAKRVLNVVCKHCGQKTTTLKGVEAVMYMGTLSYDELKTGVSIPC 1758
SP|P0C6W6|R1AB_BCRP3           VRETMTHLLQHANLESAKRVLNVVCKHCGQKTTTLTGVEAVMYMGTLSYDNLKMGVSIPC 1762
SP|P0C6V9|R1AB_BC279           VRETMTHLLQHANLESAKRVLNVVCKTCGQKSTTLTGVEAVMYMGTLSYEELKTGVTIPC 1770
TR|A0A0U1WHI4|A0A0U1WHI4_CVHSA VRETMTHLLQHANLEFAKRVLNLVCKHCGQKTTTLTGVEAVMYMGTLSYDELKTGVSIPC 1759
TR|A0A0U1WHG0|A0A0U1WHG0_CVHSA VRETMTHLLQHANLEFAKRVLNLVCKHCGQKTTTLTGVEAVMYMGTLSYDELKTGVSIPC 1759
TR|A0A166ZL34|A0A166ZL34_9NIDO VRETMTHLLQHANLEFAKRVLNLVCKHCGQKTTTLTGVEAVMYMGTLSYDELKTGVSIPC 1759
TR|R9QTB2|R9QTB2_CVHSA         VRETMTHLLQHANLESAKRVLNVVCKHCGQKTTTLTGVEAVMYMGTLSYDELKTGVSIPC 1756
TR|R9QTH2|R9QTH2_CVHSA         VRETMAHLLQHANLESAKRVLNVVCKHCGQKTTTLTGVEAVMYMGTLSYDNLKTGVSVPC 1765
SP|P0C6U8|R1A_CVHSA            VRETMTHLLQHANLESAKRVLNVVCKHCGQKTTTLTGVEAVMYMGTLSYDNLKTGVSIPC 1764
TR|Q6JH47|Q6JH47_CVHSA         VRETMTHLLQHANLESAKRVLNVVCKHCGQKTTTLTGVEAVMYMGTLSYDNLKTGVSIPC 1764
TR|Q692E5|Q692E5_CVHSA         VRETMTHLLQHANLESAKRVLNVVCKHCGQKTTTLTGVEAVMYMGTLSYDNLKTGVSIPC 1764
SP|P0C6F8|R1A_BCHK3            VRETMTHLLQHANLESAKRVLNVVCKHCGQKTTTLKGVEAVMYMGTLSYDELKTGVSIPC 1758
TR|A0A0K1Z0N1|A0A0K1Z0N1_CVHSA VRETMTHLLQHANLESAKRVLNVVCKHCGQKTTTLTGVEAVMYMGTLSYDNLKTGVSIPC 1764
SP|P0C6F5|R1A_BC279            VRETMTHLLQHANLESAKRVLNVVCKTCGQKSTTLTGVEAVMYMGTLSYEELKTGVTIPC 1770
SP|P0C6T7|R1A_BCRP3            VRETMTHLLQHANLESAKRVLNVVCKHCGQKTTTLTGVEAVMYMGTLSYDNLKMGVSIPC 1762
                               *****::*:*****: .*****:*** ***: ***.**************::* ** :**

QHN73794                       TCGKQATKYLVQQESPFVMMSAPPAQYELKHGTFTCASEYTGNYQCGHYKHITSKETLYC 1847
SP|P0C6X7|R1AB_CVHSA           VCGRDATQYLVQQESSFVMMSAPPAEYKLQQGTFLCANEYTGNYQCGHYTHITAKETLYR 1824
TR|Q6UZF5|Q6UZF5_CVHSA         VCGRDATQYLVQQESSFVMMSAPPAEYKLQQGTFLCANEYTGNYQCGHYTHITAKETLYR 1824
TR|Q6UZF1|Q6UZF1_CVHSA         VCGRDATQYLVQQESSFVMMSAPPAEYKLQQGTFLCANEYTGNYQCGHYTHITAKETLYR 1824
TR|Q6JH48|Q6JH48_CVHSA         VCGRDATQYLVQQESSFVMMSAPPAEYKLQQGTFLCANEYTGNYQCGHYTHITAKETLYR 1824
TR|Q692E6|Q692E6_CVHSA         VCGRDATQYLVQQESSFVMMSAPPAEYKLQQGTFLCANEYTGNYQCGHYTHITAKETLYR 1824
TR|A0A0K1YZY7|A0A0K1YZY7_CVHSA VCGRDATQYLIQQESSFVMMSAPPAEYKLQQGTFLCANEYTGNYQCGHYTHVTAKETLYR 1824
SP|P0C6W2|R1AB_BCHK3           VCGRNATQYLVQQESSFVMMSAPPAEYKLQQGAFLCANEYTGNYQCGHYTHITAKETLYR 1818
SP|P0C6W6|R1AB_BCRP3           VCGRDATQYLVQQESSFVMMSAPPAEYKLQQGTFLCANEYTGNYQCGHYTHITAKETLYR 1822
SP|P0C6V9|R1AB_BC279           ICGRDATQYLVQQESSFVMMSAPPSEYTLQQGAFLCANEYTGSYQCGHYTHVTVKETLYR 1830
TR|A0A0U1WHI4|A0A0U1WHI4_CVHSA VCGRGATQYLVQQESSFVMMSAPPAEYKLQQGTFLCANEYTGNYQCGHYTHITAKETLYR 1819
TR|A0A0U1WHG0|A0A0U1WHG0_CVHSA VCGRDATQYLVQQESSFVMMSAPPAEYKLQQSTFLCANEYTGNYQCGHYTHITAKETLYR 1819
TR|A0A166ZL34|A0A166ZL34_9NIDO VCGRDATQYLVQQESSFVMMSAPPAEYKLQQGTFLCANEYTGNYQCGHYTHITAKETLYH 1819
TR|R9QTB2|R9QTB2_CVHSA         VCGRNATQYLVQQESSFVMMSAPPAEYKLQQGTFLCANEYTGNYQCGHYTHITAKETLYR 1816
TR|R9QTH2|R9QTH2_CVHSA         VCGRDATQYLVQQESSFVMMSAPPAEYKLQQGTFLCANEYTGNYQCGHYTHITAKETLYR 1825
SP|P0C6U8|R1A_CVHSA            VCGRDATQYLVQQESSFVMMSAPPAEYKLQQGTFLCANEYTGNYQCGHYTHITAKETLYR 1824
TR|Q6JH47|Q6JH47_CVHSA         VCGRDATQYLVQQESSFVMMSAPPAEYKLQQGTFLCANEYTGNYQCGHYTHITAKETLYR 1824
TR|Q692E5|Q692E5_CVHSA         VCGRDATQYLVQQESSFVMMSAPPAEYKLQQGTFLCANEYTGNYQCGHYTHITAKETLYR 1824
SP|P0C6F8|R1A_BCHK3            VCGRNATQYLVQQESSFVMMSAPPAEYKLQQGAFLCANEYTGNYQCGHYTHITAKETLYR 1818
TR|A0A0K1Z0N1|A0A0K1Z0N1_CVHSA VCGRDATQYLIQQESSFVMMSAPPAEYKLQQGTFLCANEYTGNYQCGHYTHVTAKETLYR 1824
SP|P0C6F5|R1A_BC279            ICGRDATQYLVQQESSFVMMSAPPSEYTLQQGAFLCANEYTGSYQCGHYTHVTVKETLYR 1830
SP|P0C6T7|R1A_BCRP3            VCGRDATQYLVQQESSFVMMSAPPAEYKLQQGTFLCANEYTGNYQCGHYTHITAKETLYR 1822
                                **: **:**:**** ********::* *::.:* **.****.******.*:* *****

QHN73794                       IDGALLTKSSEYKGPITDVFYKENSYTTTIKPVTYKLDGVVCTEIDPKLDNYYKKDNSYF 1907
SP|P0C6X7|R1AB_CVHSA           IDGAHLTKMSEYKGPVTDVFYKETSYTTTIKPVSYKLDGVTYTEIEPKLDGYYKKDNAYY 1884
TR|Q6UZF5|Q6UZF5_CVHSA         IDGAHLTKMSEYKGPVTDVFYKETSYTTTIKPVSYKLDGVTYTEIEPKLDGYYKKDNAYY 1884
TR|Q6UZF1|Q6UZF1_CVHSA         IDGAHLTKMSEYKGPVTDVFYKETSYTTTIKPVSYKLDGVTYTEIEPKLDGYYKKDNAYY 1884
TR|Q6JH48|Q6JH48_CVHSA         IDGAHLTKMSEYKGPVTDVFYKETSYTTTIKPVSYKLDGVTYTEIEPKLDGYYKKDNAYY 1884
TR|Q692E6|Q692E6_CVHSA         IDGAHLTKMSEYKGPVTDVFYKETSYTTTIKPVSYKLDGVTYTEIEPKLDGYYKKDNAYY 1884
TR|A0A0K1YZY7|A0A0K1YZY7_CVHSA IDGAHLTKMSEYKGPVTDVFYKETSYTTTIKPVSYKLDGVTYTEIEPKLDGYYKKDNAYY 1884
SP|P0C6W2|R1AB_BCHK3           VDGAHLTKMSEYKGPVTDVFYKETSYTTAIKPVSYKLDGVTYTEIEPKLDGYYKKGNAYY 1878
SP|P0C6W6|R1AB_BCRP3           IDGAHLTKMSEYKGPVTDVFYKETSYTTTIKPVSYKLDGVTYTEIEPKLDGYYKKDNAYY 1882
SP|P0C6V9|R1AB_BC279           IDGAYLTKMSEYKGPVTDVFYKEISYTTTIKPVSYKLDGVIYTEIQPKLDEYYKKDNAYY 1890
TR|A0A0U1WHI4|A0A0U1WHI4_CVHSA IDGAHLTKMSEYKGPVTDVFYKETSYTTTIKPVSYKLDGVTYTEIEPKLDGYYKKDNAYY 1879
TR|A0A0U1WHG0|A0A0U1WHG0_CVHSA IDGAHLTKMSEYKGPVTDVFYKETSYTTTIKPVSYKLDGVTYTEIEPKLDGYYKKDNAYY 1879
TR|A0A166ZL34|A0A166ZL34_9NIDO IDGAHLTKMSEYKGPVTDVFYKETSYTTTIKPVSYKLDGVTYTEIEPKLDGYYKKDNAY- 1878
TR|R9QTB2|R9QTB2_CVHSA         IDGAHLTKMSEYKGPVTDVFYKETSYTTTIKPVSYKLDGVTYTEIEPKLDGYYKKDNAYY 1876
TR|R9QTH2|R9QTH2_CVHSA         IDGAHLTKMSEYKGPVTDVFYKETSYTTTIKPVSYKLDGVTYTEIEPKLDGYYKKDNAYY 1885
SP|P0C6U8|R1A_CVHSA            IDGAHLTKMSEYKGPVTDVFYKETSYTTTIKPVSYKLDGVTYTEIEPKLDGYYKKDNAYY 1884
TR|Q6JH47|Q6JH47_CVHSA         IDGAHLTKMSEYKGPVTDVFYKETSYTTTIKPVSYKLDGVTYTEIEPKLDGYYKKDNAYY 1884
TR|Q692E5|Q692E5_CVHSA         IDGAHLTKMSEYKGPVTDVFYKETSYTTTIKPVSYKLDGVTYTEIEPKLDGYYKKDNAYY 1884
SP|P0C6F8|R1A_BCHK3            VDGAHLTKMSEYKGPVTDVFYKETSYTTAIKPVSYKLDGVTYTEIEPKLDGYYKKGNAYY 1878
TR|A0A0K1Z0N1|A0A0K1Z0N1_CVHSA IDGAHLTKMSEYKGPVTDVFYKETSYTTTIKPVSYKLDGVTYTEIEPKLDGYYKKDNAYY 1884
SP|P0C6F5|R1A_BC279            IDGAYLTKMSEYKGPVTDVFYKEISYTTTIKPVSYKLDGVIYTEIQPKLDEYYKKDNAYY 1890
SP|P0C6T7|R1A_BCRP3            IDGAHLTKMSEYKGPVTDVFYKETSYTTTIKPVSYKLDGVTYTEIEPKLDGYYKKDNAYY 1882
                               :*** *** ******:******* ****:****:******  ***:**** ****.*:*
```
