# Supplementary material for: Structural Genomics of SARS-CoV-2 Indicates Evolutionary Conserved Functional Regions of Viral Proteins
Source: Viruses. 2020 Mar 25;12(4):360. doi: 10.3390/v12040360 (PMC7232164; doi:10.3390/v12040360)
Supplement: Supplementary file 1 [file viruses-12-00360-s001.zip › SuppTable_S1.pdf]

Supplementary Table S1. The closest UniProt blast search hits for 2019-nCoV proteins and their comparison with the three novel isolates of BatCoV (2013, 2015, and 2017)

2019-nCov protein	UniProt ID	Virus/Strain_Isolate/Year	Seq ID, %	2017 BatCoV Seq ID, %	2013 BatCoV Seq ID, %	NCBI ID 2017	NCBI ID 2015	NCBI ID 2013
wPRF1ab	A0A166ZL34	Bat coronavirus/JTMC15/2013	91.3	95.7	98.6	AVP78030.1	AVP78041.1	QHR63299.1
	P0C6X7	Human SARS-CoV/Urbani/2003	86.2					
	A0A0U1WHI4	BtRf-BetaCoV/SX2013/2013	85.8					
wN	R9QTB4	Bat coronavirus/Rp/Shaanxi2011/2011	91.0	94.3	99.1	AVP78038.1	AVP78049.1	QHR63308.1
	P59595	Human SARS-CoV/Urbani/2003	90.5					
	A0A0U1WHI6	BtRf-BetaCoV/SX2013/2013	89.8					
wORF3a	Q0Q474	Bat coronavirus, BtCoV/279/2005	74.5	91.0	97.8	AVP78032.1	AVP78043.1	QHR63301.1
	P59632	Human SARS-CoV /Urbani/2003	72.7					
	A0A0U1UZ48	BtRf-BetaCoV/SX2013/2013	70.9					
wE	Q3I5J3	Bat coronavirus, BtCoV/Rp3/2004	94.7	100.0	100.0	AVP78033.1	AVP78044.1	QHR63302.1
	P59637	Human SARS-CoV/Urbani/2003	94.7					
	A0A0U1WJY0	BtRf-BetaCoV/SX2013/2013	92.1					
wM	Q0Q472	Bat coronavirus, BtCoV/279/2005	91.7	98.7	99.6	AVP78034.1		QHR63303.1
	P59596	Human SARS-CoV/Urbani/2003	90.5					
	A0A0U1WHH9	BtRf-BetaCoV/SX2013/2013	90.4					
wORF6	Q3I5J1	Bat coronavirus, BtCoV/Rp3/2004	68.9	93.4	100.0	AVP78035.1	AVP78046.1	QHR63304.1
	P59634	Human SARS-CoV/Urbani/2003	68.9					
	A0A0U1WHI0	BtRf-BetaCoV/SX2013/2013	68.9					
wORF7a	Q3I5J0	Bat coronavirus, BtCoV/Rp3/2004	88.5	88.4	97.5	AVP78036.1	AVP78047.1	QHR63305.1
	A0A0U1UZE3	BtRf-BetaCoV/SX2013/2013	86.1					
	P59635	Human SARS-CoV/Urbani/2003	85.2					
wORF7b	Q3I5I9	Bat coronavirus, BtCoV/Rp3/2004	85.7	N/A	97.7			QHR63306.1
	A0A0U1WHI9	BtRf-BetaCoV/SX2013/2013	85.7					
	Q7TFA1	Human SARS-CoV/Urbani/2003	85.4					
wORF8	Q0Q469	Bat coronavirus, BtCoV/279/2005	57.0	94.2	95.0	AVP78037.1	AVP78048.1	QHR63307.1
wS	Q3LZX1	Bat coronavirus, BtCoV/HKU3-1/2005	76.0	81.0	97.4	AVP78031.1	AVP78042.1	QHR63300.1
	P59594	Human SARS-CoV/Urbani/2003	76.0					
	A0A0U1WHI6	BtRf-BetaCoV/SX2013/2013	73.7					
wORF10	FJ882928.1	Human SARS-CoV/ExoN1 isolate P1pp1/2007	84.2	97.4	100	MG772933.1 (translated)		MN996532.1 (translated)
	DQ648857.1	Bat coronavirus, BtCoV/279/2005	81.6					
