# Supplementary material for: Structural Genomics of SARS-CoV-2 Indicates Evolutionary Conserved Functional Regions of Viral Proteins
Source: Viruses. 2020 Mar 25;12(4):360. doi: 10.3390/v12040360 (PMC7232164; doi:10.3390/v12040360)
Supplement: Supplementary file 1 [file viruses-12-00360-s001.zip › wNsp5-SARS_LBS2_mapped.pdf]

Supplementary File 5. Ligand binding sites extracted from SARS protein targets and mapped on the sequence of wNsp5 protein aligned with the closest homologs found in UniProt. Shown is the second group of ligands.

Ligand BS:

ETHYL (5S,8S,11R)-8-BENZYL-5-(3-TERT-BUTOXY-3-OXOPROPYL)-3,6,9-TRIOXO-11-{[(3S)-2-OXOPYRROLIDIN-3-YL]METHYL}-1-PHENYL-2-OXA-4,7,10-TRIAZATETRADECAN-14-OATE (3TIU-G82, SARS)

ETHYL (5S,8S,11R)-8-BENZYL-5-(2-TERT-BUTOXY-2-OXOETHYL)-3,6,9-TRIOXO-11-{[(3S)-2-OXOPYRROLIDIN-3-YL]METHYL}-1-PHENYL-2-OXA-4,7,10-TRIAZATETRADECAN-14-OATE (3TNS-G83, SARS)

N-[(benzyloxy)carbonyl]-O-tert-butyl-L-seryl-N-{(2R)-5-ethoxy-5-oxo-1-[(3S)-2-oxopyrrolidin-3-yl]pentan-2-yl}-L-phenylalaninamide (3TNT-G85, SARS)

N-[(5-METHYLISOXAZOL-3-YL)CARBONYL]-L-ALANYL-L-VALYL-N~1~-((1S)-4-ETHOXY-4-OXO-1-{[(3S)-2-OXOPYRROLIDIN-3-YL]METHYL}BUT-2-ENYL)-L-LEUCINAMIDE (1WOF-I12, SARS)

N-[(BENZYLOXY)CARBONYL]-O-(TERT-BUTYL)-L-THREONYL-3-CYCLOHEXYL-N-[(1S)-2-HYDROXY-1-{[(3S)-2-OXOPYRROLIDIN-3-YL]METHYL}ETHYL]-L-ALANINAMIDE (2GX4-NOL, SARS)

N-[(2S)-1-hydroxy-3-phenylpropan-2-yl]-Nalpha-[(2E)-3-phenylprop-2-enoyl]-L-phenylalaninamide (3SN8-S89, SARS)

(2S)-3-(1H-imidazol-5-yl)-2-({[(3S,4aR,8aS)-2-(N-phenyl-beta-alanyl)decahydroisoquinolin-3-yl]methyl}amino)propanal (5C5O-SDJ, SARS)

(2S)-3-(1H-imidazol-5-yl)-2-({[(3R,4aS,8aR)-2-(N-phenyl-beta-alanyl)decahydroisoquinolin-3-yl]methyl}amino)propanal (5C5N-SLH, SARS)

4-(DIMETHYLAMINO)BENZOIC ACID (2V6N-XP1, SARS)

N-[(benzyloxy)carbonyl]-3-[(2,2-dimethylpropanoyl)amino]-L-alanyl-N-[(1R)-4-oxo-1-{[(3S)-2-oxopyrrolidin-3-yl]methyl}pentyl]-L-leucinamide (2ZU4-ZU3, SARS)

N-[(benzyloxy)carbonyl]-O-tert-butyl-L-threonyl-N-[(1R)-4-cyclopropyl-4-oxo-1-{[(3S)-2-oxopyrrolidin-3-yl]methyl}butyl]-L-leucinamide (2ZU5-ZU5, SARS)

(5S,8S,14R)-ETHYL 11-(3-AMINO-3-OXOPROPYL)-8-BENZYL-14-HYDROXY-5-ISOBUTYL-3,6,9,12-TETRAOXO-1-PHENYL-2-OXA-4,7,10,11-TETRAAZAPENTADECAN-15-OATE (2GTB-AZP, SARS)

```
QHN73794                     EAACCHLAKALNDFSNSGSDVLYQPPQTSITSAVLQSGFRKMAFPSGKVEGCMVQVTCGT 3287
SP|P0C6X7|R1AB_CVHSA         EAACCHLAKALNDFSNSGADVLYQPPQTSITSAVLQSGFRKMAFPSGKVEGCMVQVTCGT 3264
TR|Q6UZF5|Q6UZF5_CVHSA       EAACCHLAKALNDFSNSGADVLYQPPQTSITSAVLQSGFRKMAFPSGKVEGCMVQVTCGT 3264
TR|Q6UZF1|Q6UZF1_CVHSA       EAACCHLAKALNDFSNSGADVLYQPPQTSITSAVLQSGFRKMAFPSGKVEGCMVQVTCGT 3264
TR|Q6JH48|Q6JH48_CVHSA       EAACCHLAKALNDFSNSGADVLYQPPQTSITSAVLQSGFRKMAFPSGKVEGCMVQVTCGT 3264
TR|Q692E6|Q692E6_CVHSA       EAACCHLAKALNDFSNSGADVLYQPPQTSITSAVLQSGFRKMAFPSGKVEGCMVQVTCGT 3264
TR|A0A0K1YZY7|A0A0K1YZY7_CVHSA EAACCHLAKALNDFSNSGSDVLYQPPQTSITSAVLQSGFRKMAFPSGKVEGCMVQVTCGT 3264
SP|P0C6W2|R1AB_BCHK3         EAACCHLAKALNDFSNSGADVLYQPPQTSITSAVLQSGFRKMAFPSGKVEGCMVQVTCGT 3258
SP|P0C6W6|R1AB_BCRP3         EAACCHLAKALNDFSNSGADVLYQPPQTSITSAVLQSGFRKMAFPSGKVEGCMVQVTCGT 3262
SP|P0C6V9|R1AB_BC279         EAACCHLAKALNDFSNSGADVLYQPPQTSITSAVLQSGFRKMAFPSGKVEGCMVQVTCGT 3270
TR|A0A0U1WHI4|A0A0U1WHI4_CVHSA EAACCHLAKALNDFSNSGADVLYQPPQTSITSAVLQSGFRRMAFPSGKVEGCMVQVTCGT 3259
TR|A0A0U1WHG0|A0A0U1WHG0_CVHSA EAACCHLAKALNDFSNSGADVLYQPPQTSITSAVLQSGFKKVAFPSGKVEGCMVQVTCGT 3259
TR|A0A166ZL34|A0A166ZL34_9NIDO EAACCHLAKALNDFSNSGADVLYQPPQTSITSAVLQSGFKKMAFPSGKVEGCMVQVTCGT 3066
TR|R9QTB2|R9QTB2_CVHSA       EAACCHLAKALNDFSNSGADVLYQPPQTSITSAVLQSGFRKMAFPSGKVEGCMVQVTCGT 3256
TR|R9QTH2|R9QTH2_CVHSA       EAACCHLAKALNDFSNSGSDVLYQPPQTSITSAVLQSGFRKMAFPSGKVEGCMVQVTCGT 3265
SP|P0C6U8|R1A_CVHSA          EAACCHLAKALNDFSNSGADVLYQPPQTSITSAVLQSGFRKMAFPSGKVEGCMVQVTCGT 3264
TR|Q6JH47|Q6JH47_CVHSA       EAACCHLAKALNDFSNSGADVLYQPPQTSITSAVLQSGFRKMAFPSGKVEGCMVQVTCGT 3264
TR|Q692E5|Q692E5_CVHSA       EAACCHLAKALNDFSNSGADVLYQPPQTSITSAVLQSGFRKMAFPSGKVEGCMVQVTCGT 3264
SP|P0C6F8|R1A_BCHK3          EAACCHLAKALNDFSNSGADVLYQPPQTSITSAVLQSGFRKMAFPSGKVEGCMVQVTCGT 3258
TR|A0A0K1Z0N1|A0A0K1Z0N1_CVHSA EAACCHLAKALNDFSNSGSDVLYQPPQTSITSAVLQSGFRKMAFPSGKVEGCMVQVTCGT 3264
SP|P0C6F5|R1A_BC279          EAACCHLAKALNDFSNSGADVLYQPPQTSITSAVLQSGFRKMAFPSGKVEGCMVQVTCGT 3270
SP|P0C6T7|R1A_BCRP3          EAACCHLAKALNDFSNSGADVLYQPPQTSITSAVLQSGFRKMAFPSGKVEGCMVQVTCGT 3262
                             ******************:********************:::******************
```

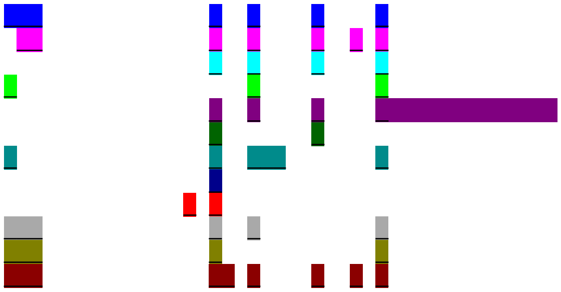

```
QHN73794                           TTLNGLWLDDVVYCPRHVICTSEDMLNPNYEDLLIRKSNHNFLVQAGNVQLRVIGHSMQN 3347
SP|P0C6X7|R1AB_CVHSA               TTLNGLWLDDTVYCPRHVICTAEDMLNPNYEDLLIRKSNHSFLVQAGNVQLRVIGHSMQN 3324
TR|Q6UZF5|Q6UZF5_CVHSA             TTLNGLWLDDTVYCPRHVICTAEDMLNPNYEDLLIRKSNHSFLVQAGNVQLRVIGHSMQN 3324
TR|Q6UZF1|Q6UZF1_CVHSA             TTLNGLWLDDTVYCPRHVICTAEDMLNPNYEDLLIRKSNHSFLVQAGNVQLRVIGHSMQN 3324
TR|Q6JH48|Q6JH48_CVHSA             TTLNGLWLDDTVYCPRHVICTAEDMLNPNYEDLLIRKANHSFLVQAGNVQLRVIGHSMQN 3324
TR|Q692E6|Q692E6_CVHSA             TTLNGLWLDDTVYCPRHVICTAEDMLNPNYEDLLIRKSNHSFLVQAGNVQLRVIGHSMQN 3324
TR|A0A0K1YZY7|A0A0K1YZY7_CVHSA     TTLNGLWLDDTVYCPRHVICTAEDMLNPNYEDLLIRKSNHSFLVQAGNVQLRVIGHSMQN 3324
SP|P0C6W2|R1AB_BCHK3               TTLNGLWLDDTVYCPRHVVCTAEDMLNPNYDDLLIRKSNHSFLVQAGNVQLRVIGHSMQN 3318
SP|P0C6W6|R1AB_BCRP3               TTLNGLWLDDTVYCPRHVICTAEDMLNPNYEDLLIRKSNHSFLVQAGNVQLRVIGHSMQN 3322
SP|P0C6V9|R1AB_BC279               TTLNGLWLDDTVYCPRHVICTAEDMLNPNYEDLLIRKSNHSFLVQAGNVQLRVIGHSMQN 3330
TR|A0A0U1WHI4|A0A0U1WHI4_CVHSA     TTLNGLWLDDTVYCPRHVICTAEDMLNPNYEDLLIRKSNHSFLVQAGNVQLRVIGHSMQN 3319
TR|A0A0U1WHG0|A0A0U1WHG0_CVHSA     TTLNGLWLDDTVYCPRHVVCTVEDMLNPNYEDLLIRKSNHSFLVQTGNVQLRVIGHSMQN 3319
TR|A0A166ZL34|A0A166ZL34_9NIDO     TTLNGLWLDDTVYCPRHVVCTVEDMLNPNYEDLLIRKSNHSFLVQTGNVQLRVIGHSMQN 3126
TR|R9QTB2|R9QTB2_CVHSA             TTLNGLWLDDTVYCPRHVICTAEDMLNPNYEDLLIRKSNHSFLVQAGNVQLRVIGHSMQN 3316
TR|R9QTH2|R9QTH2_CVHSA             TTLNGLWLDDTVYCPRHVICTAEDMLNPNYEDLLIRKSNHSFLVQAGNVQLRVIGHSMQN 3325
SP|P0C6U8|R1A_CVHSA                TTLNGLWLDDTVYCPRHVICTAEDMLNPNYEDLLIRKSNHSFLVQAGNVQLRVIGHSMQN 3324
TR|Q6JH47|Q6JH47_CVHSA             TTLNGLWLDDTVYCPRHVICTAEDMLNPNYEDLLIRKANHSFLVQAGNVQLRVIGHSMQN 3324
TR|Q692E5|Q692E5_CVHSA             TTLNGLWLDDTVYCPRHVICTAEDMLNPNYEDLLIRKSNHSFLVQAGNVQLRVIGHSMQN 3324
SP|P0C6F8|R1A_BCHK3                TTLNGLWLDDTVYCPRHVVCTAEDMLNPNYDDLLIRKSNHSFLVQAGNVQLRVIGHSMQN 3318
TR|A0A0K1Z0N1|A0A0K1Z0N1_CVHSA     TTLNGLWLDDTVYCPRHVICTAEDMLNPNYEDLLIRKSNHSFLVQAGNVQLRVIGHSMQN 3324
SP|P0C6F5|R1A_BC279                TTLNGLWLDDTVYCPRHVICTAEDMLNPNYEDLLIRKSNHSFLVQAGNVQLRVIGHSMQN 3330
SP|P0C6T7|R1A_BCRP3                TTLNGLWLDDTVYCPRHVICTAEDMLNPNYEDLLIRKSNHSFLVQAGNVQLRVIGHSMQN 3322
                                   **********.*******:** ********:******:**.****:**************
```

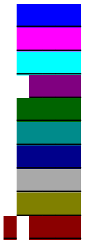

```
QHN73794                           CVLKLKVDTANPKTPKYKFVRIQPGQTFSVLACYNGSPSGVYQCAMRPNFTIKGSFLNGS 3407
SP|P0C6X7|R1AB_CVHSA               CLLRLKVDTSNPKTPKYKFVRIQPGQTFSVLACYNGSPSGVYQCAMRPNHTIKGSFLNGS 3384
TR|Q6UZF5|Q6UZF5_CVHSA             CLLRLKVDTSNPKTPKYKFVRIQPGQTFSVLACYNGSPSGVYQCAMRPNHTIKGSFLNGS 3384
TR|Q6UZF1|Q6UZF1_CVHSA             CLLRLKVDTSNPKTPKYKFVRIQPGQTFSVLACYNGSPSGVYQCAMRPNHTIKGSFLNGS 3384
TR|Q6JH48|Q6JH48_CVHSA             CLLRLKVDTSNPKTPKYKFVRIQPGQTFSVLACYNGSPSGVYQCAMRPNHTIKGSFLNGS 3384
TR|Q692E6|Q692E6_CVHSA             CLLRLKVDTSNPKTPKYKFVRIQPGQTFSVLACYNGSPSGVYQCAMRPNHTIKGSFLNGS 3384
TR|A0A0K1YZY7|A0A0K1YZY7_CVHSA     CLLRLKVDTSNPKTPKYKFVRIQPGQTFSVLACYNGSPSGVYQCAMRPNHTIKGSFLNGS 3384
SP|P0C6W2|R1AB_BCHK3               CLLRLKVDTSNPKTPKYKFVRIQPGQTFSVLACYNGSPSGVYQCAMRPNHTIKGSFLNGS 3378
SP|P0C6W6|R1AB_BCRP3               CLLRLKVDTSNPKTPKYKFVRIQPGQTFSVLACYNGSPSGVYQCAMRPNHTIKGSFLNGS 3382
SP|P0C6V9|R1AB_BC279               CLLRLKVDTSNPKTPKYKFVRIQPGQTFSVLACYNGSPSGVYQCAMRPNYTIKGSFLNGS 3390
TR|A0A0U1WHI4|A0A0U1WHI4_CVHSA     CLLRLKVDTSNPKTPKYKFVRIQPGQTFSVLACYNGSPSGVYQCAMRPNYTIKGSFLNGS 3379
TR|A0A0U1WHG0|A0A0U1WHG0_CVHSA     CLLRLKVDTSNPKTPKYKFVRIQPGQTFSVLACYNGSPSGVYQCAMRPNYTIKGSFLNGS 3379
TR|A0A166ZL34|A0A166ZL34_9NIDO     CLLRLKVDTSNPKTPKYKFVRIQPGQTFSVLACYNGSPSGVYQCAMRPNYTIKGSFLNGS 3186
TR|R9QTB2|R9QTB2_CVHSA             CLLRLKVDTSNPKTPKYKFVRIQPGQTFSVLACYNGSPSGVYQCAMRPNHTIKGSFLNGS 3376
TR|R9QTH2|R9QTH2_CVHSA             CLLRLKVDTSNPKTPKYKFVRIQPGQTFSVLACYNGSPSGVYQCAMRPNHTIKGSFLNGS 3385
SP|P0C6U8|R1A_CVHSA                CLLRLKVDTSNPKTPKYKFVRIQPGQTFSVLACYNGSPSGVYQCAMRPNHTIKGSFLNGS 3384
TR|Q6JH47|Q6JH47_CVHSA             CLLRLKVDTSNPKTPKYKFVRIQPGQTFSVLACYNGSPSGVYQCAMRPNHTIKGSFLNGS 3384
TR|Q692E5|Q692E5_CVHSA             CLLRLKVDTSNPKTPKYKFVRIQPGQTFSVLACYNGSPSGVYQCAMRPNHTIKGSFLNGS 3384
SP|P0C6F8|R1A_BCHK3                CLLRLKVDTSNPKTPKYKFVRIQPGQTFSVLACYNGSPSGVYQCAMRPNHTIKGSFLNGS 3378
TR|A0A0K1Z0N1|A0A0K1Z0N1_CVHSA     CLLRLKVDTSNPKTPKYKFVRIQPGQTFSVLACYNGSPSGVYQCAMRPNHTIKGSFLNGS 3384
SP|P0C6F5|R1A_BC279                CLLRLKVDTSNPKTPKYKFVRIQPGQTFSVLACYNGSPSGVYQCAMRPNYTIKGSFLNGS 3390
SP|P0C6T7|R1A_BCRP3                CLLRLKVDTSNPKTPKYKFVRIQPGQTFSVLACYNGSPSGVYQCAMRPNHTIKGSFLNGS 3382
                                   *:*:*****:***************************************.**********
```

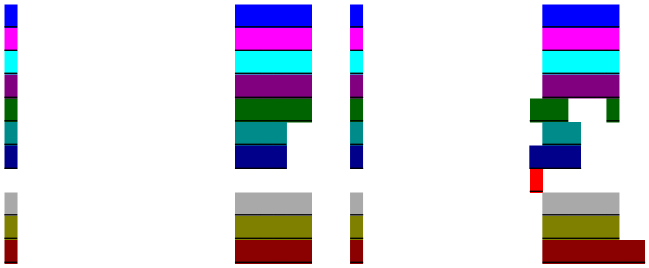

```
QHN73794                        CGSVGFNIDYDCVSFCYMHHMELPTGVHAGTDLEGNFYGPFVDRQTAQAAGTDTTITVNV 3467
SP|P0C6X7|R1AB_CVHSA            CGSVGFNIDYDCVSFCYMHHMELPTGVHAGTDLEGKFYGPFVDRQTAQAAGTDTTITLNV 3444
TR|Q6UZF5|Q6UZF5_CVHSA          CGSVGFNIDYDCVSFCYMHHMELPTGVHAGTDLEGKFYGPFVDRQTAQAAGTDTTITLNV 3444
TR|Q6UZF1|Q6UZF1_CVHSA          CGSVGFNIDYDCVSFCYMHHMELPTGVHAGTDLEGKFYGPFVDRQTAQAAGTDTTITLNV 3444
TR|Q6JH48|Q6JH48_CVHSA          CGSVGFNIDYDCVSFCYMHHMELPTGVHAGTDLEGKFYGPFVDRQTAQAAGTDTTITLNV 3444
TR|Q692E6|Q692E6_CVHSA          CGSVGFNIDYDCVSFCYMHHMELPTGVHAGTDLEGKFYGPFVDRQTAQAAGTDTTITLNV 3444
TR|A0A0K1YZY7|A0A0K1YZY7_CVHSA  CGSVGFNIDYDCVSFCYMHHMELPTGVHAGTDLEGKFYGPFVDRQTAQAAGTDTTITLNV 3444
SP|P0C6W2|R1AB_BCHK3            CGSVGFNIDYDCVSFCYMHHMELPTGVHAGTDLEGKFYGPFVDRQTAQAAGTDTTITLNV 3438
SP|P0C6W6|R1AB_BCRP3            CGSVGFNIDYDCVSFCYMHHMELPTEVHAGTDLEGKFYGPFVDRQTAQAAGTDTTITLNV 3442
SP|P0C6V9|R1AB_BC279            CGSVGFNIDYDCVSFCYMHHMELPTGVHAGTDLEGKFYGPFVDRQTAQAAGTDTTITLNV 3450
TR|A0A0U1WHI4|A0A0U1WHI4_CVHSA  CGSVGFNIDYDCVSFCYMHHMELPTGVHAGTDLEGKFYGPFVDRQTAQAAGTDTTITLNV 3439
TR|A0A0U1WHG0|A0A0U1WHG0_CVHSA  CGSVGFNIDYDCVSFCYMHHMELPTGVHAGTDLEGKFYGPFVDRQTAQAAGTDTTITLNV 3439
TR|A0A166ZL34|A0A166ZL34_9NIDO  CGSVGFNIDYDCVSFCYMHHMELPTGVHAGTDLEGKFYGPFVDRQTAQAAGTDTAITLNV 3246
TR|R9QTB2|R9QTB2_CVHSA          CGSVGFNIDYDCVSFCYMHHMELPTGVHAGTDLEGKFYGPFVDRQTAQAAGTDTTITLNV 3436
TR|R9QTH2|R9QTH2_CVHSA          CGSVGFNIDYDCVSFCYMHHMELPTGVHAGTDLEGKFYGPFVDRQTAQAAGTDTTITLNV 3445
SP|P0C6U8|R1A_CVHSA             CGSVGFNIDYDCVSFCYMHHMELPTGVHAGTDLEGKFYGPFVDRQTAQAAGTDTTITLNV 3444
TR|Q6JH47|Q6JH47_CVHSA          CGSVGFNIDYDCVSFCYMHHMELPTGVHAGTDLEGKFYGPFVDRQTAQAAGTDTTITLNV 3444
TR|Q692E5|Q692E5_CVHSA          CGSVGFNIDYDCVSFCYMHHMELPTGVHAGTDLEGKFYGPFVDRQTAQAAGTDTTITLNV 3444
SP|P0C6F8|R1A_BCHK3             CGSVGFNIDYDCVSFCYMHHMELPTGVHAGTDLEGKFYGPFVDRQTAQAAGTDTTITLNV 3438
TR|A0A0K1Z0N1|A0A0K1Z0N1_CVHSA  CGSVGFNIDYDCVSFCYMHHMELPTGVHAGTDLEGKFYGPFVDRQTAQAAGTDTTITLNV 3444
SP|P0C6F5|R1A_BC279             CGSVGFNIDYDCVSFCYMHHMELPTGVHAGTDLEGKFYGPFVDRQTAQAAGTDTTITLNV 3450
SP|P0C6T7|R1A_BCRP3             CGSVGFNIDYDCVSFCYMHHMELPTEVHAGTDLEGKFYGPFVDRQTAQAAGTDTTITLNV 3442
                                ************************* *********:******************:**:**

QHN73794                        LAWLYAAVINGDRWFLNRFTTTLNDFNLVAMKYNYEPLTQDHVDILGPLSAQTGIAVLDM 3527
SP|P0C6X7|R1AB_CVHSA            LAWLYAAVINGDRWFLNRFTTTLNDFNLVAMKYNYEPLTQDHVDILGPLSAQTGIAVLDM 3504
TR|Q6UZF5|Q6UZF5_CVHSA          LAWLYAAVINGDRWFLNRFTTTLNDFNLVAMKYNYEPLTQDHVDILGPLSAQTGIAVLDM 3504
TR|Q6UZF1|Q6UZF1_CVHSA          LAWLYAAVINGDRWFLNRFTTTLNDFNLVAMKYNYEPLTQDHVDILGPLSAQTGIAVLDM 3504
TR|Q6JH48|Q6JH48_CVHSA          LAWLYAAVINGDRWFLNRFTTTLNDFNLVAMKYNYEPLTQDHVDILGPLSAQTGIAVLDM 3504
TR|Q692E6|Q692E6_CVHSA          LAWLYAAVINGDRWFLNRFTTTLNDFNLVAMKYNYEPLTQDHVDILGPLSAQTGIAVLDM 3504
TR|A0A0K1YZY7|A0A0K1YZY7_CVHSA  LAWLYAAVINGDRWFLNRFTTTLNDFNLVAMKYNYEPLTQDHVDVLGPLSAQTGIAVLDM 3504
SP|P0C6W2|R1AB_BCHK3            LAWLYAAVINGDRWFLNRFTTTLNDFNLVAMKYNYEPLTQDHVDILGPLSAQTGIAVLDM 3498
SP|P0C6W6|R1AB_BCRP3            LAWLYAAVINGDRWFLNRFTTTLNDFNLVAMKYNYEPLTQDHVDILGPLSAQTGIAVLDM 3502
SP|P0C6V9|R1AB_BC279            LAWLYAAVINGDRWFLNRFTTTLNDFNLVAMKYNYEPLTQDHVDILGPLSAQTGIAVLDM 3510
TR|A0A0U1WHI4|A0A0U1WHI4_CVHSA  LAWLYAAVINGDRWFLNRFTTTLNDFNLVAMKYNYEPLTQDHVDILGPLSAQTGIAVLDM 3499
TR|A0A0U1WHG0|A0A0U1WHG0_CVHSA  LAWLYAAVINGDRWFLNRFTTTLNDFNLVAMKYNYEPLTQDHVDILGPLSAQTGIAVLDM 3499
TR|A0A166ZL34|A0A166ZL34_9NIDO  LAWLYAAVINGDRWFLNRFTTTLNDFNLVAMKYNYEPLTQDHVDILGPLSAQTGIAVLDM 3306
TR|R9QTB2|R9QTB2_CVHSA          LAWLYAAVINGDRWFLNRFTTTLNDFNLVAMKYNYEPLTQDHVDILGPLSAQTGIAVLDM 3496
TR|R9QTH2|R9QTH2_CVHSA          LAWLYAAVINGDRWFLNRFTTTLNDFNLVAMKYNYEPLTQDHVDILGPLSAQTGIAVLDM 3505
SP|P0C6U8|R1A_CVHSA             LAWLYAAVINGDRWFLNRFTTTLNDFNLVAMKYNYEPLTQDHVDILGPLSAQTGIAVLDM 3504
TR|Q6JH47|Q6JH47_CVHSA          LAWLYAAVINGDRWFLNRFTTTLNDFNLVAMKYNYEPLTQDHVDILGPLSAQTGIAVLDM 3504
TR|Q692E5|Q692E5_CVHSA          LAWLYAAVINGDRWFLNRFTTTLNDFNLVAMKYNYEPLTQDHVDILGPLSAQTGIAVLDM 3504
SP|P0C6F8|R1A_BCHK3             LAWLYAAVINGDRWFLNRFTTTLNDFNLVAMKYNYEPLTQDHVDILGPLSAQTGIAVLDM 3498
TR|A0A0K1Z0N1|A0A0K1Z0N1_CVHSA  LAWLYAAVINGDRWFLNRFTTTLNDFNLVAMKYNYEPLTQDHVDVLGPLSAQTGIAVLDM 3504
SP|P0C6F5|R1A_BC279             LAWLYAAVINGDRWFLNRFTTTLNDFNLVAMKYNYEPLTQDHVDILGPLSAQTGIAVLDM 3510
SP|P0C6T7|R1A_BCRP3             LAWLYAAVINGDRWFLNRFTTTLNDFNLVAMKYNYEPLTQDHVDILGPLSAQTGIAVLDM 3502
                                ********************************************:***************
```

```
QHN73794                       CASLKELLQNGMNGRTILGSALLEDEFTPFDVVRQCSGVTFQSAVKRTIKGTHHWLLLTI 3587
SP|P0C6X7|R1AB_CVHSA           CAALKELLQNGMNGRTILGSTILEDEFTPFDVVRQCSGVTFQGKFKKIVKGTHHWMLLTF 3564
TR|Q6UZF5|Q6UZF5_CVHSA         CAALKELLQNGMNGRTILGSTILEDEFTPFDVVRQCSGVTFQGKFKKIVKGTHHWMLLTF 3564
TR|Q6UZF1|Q6UZF1_CVHSA         CAALKELLQNGMNGRTILGSTILEDEFTPFDVVRQCSGVTFQGKFKKIVKGTHHWMLLTF 3564
TR|Q6JH48|Q6JH48_CVHSA         CAALKELLQNGMNGRTILGSTILEDEFTPFDVVRQCSGVTFQGKFKKIVKGTHHWMLLTF 3564
TR|Q692E6|Q692E6_CVHSA         CAALKELLQNGMNGRTILGSTILEDEFTPFDVVRQCSGVTFQGKFKKIVKGTHHWMLLTF 3564
TR|A0A0K1YZY7|A0A0K1YZY7_CVHSA CAALKELLQNGMNGRTILGSTILEDEFTPFDVVRQCSGVTFQGKFKKIVKGTHHWMLLTF 3564
SP|P0C6W2|R1AB_BCHK3           CAALKELLQNGMNGRTILGSTILEDEFTPFDVVRQCSGVTFQGKFKKIVKGTHHWMLLTF 3558
SP|P0C6W6|R1AB_BCRP3           CAALKELLQNGMNGRTILGSTILEDEFTPFDVVRQCSGVTFQGKFKRIVKGTHHWMLLTF 3562
SP|P0C6V9|R1AB_BC279           CAALKELLQNGMNGRTILGSTILEDEFTPFDVVRQCSGVTFQGKFKKIVKGTHHWMLLTF 3570
TR|A0A0U1WHI4|A0A0U1WHI4_CVHSA CAALKELLQNGMNGRTILGSTILEDEFTPFDVVRQCSGVTFQGKFKKIVKGTHHWMLLTF 3559
TR|A0A0U1WHG0|A0A0U1WHG0_CVHSA CAALKELLQNGMNGRTILGSTILEDEFTPFDVVRQCSGVTFQGKFKKIVKGTHHWMLLTF 3559
TR|A0A166ZL34|A0A166ZL34_9NIDO CAALKELLQNGMNGRTILGSTILEDEFTPFDVVRQCSGVTFQGKFKKIVKGTHHWMLLTF 3366
TR|R9QTB2|R9QTB2_CVHSA         CAALKELLQNGMNGRTILGSTILEDEFTPFDVVRQCSGVTFQGKFKKIVKGTHHWMLLTF 3556
TR|R9QTH2|R9QTH2_CVHSA         CAALKELLQNGMNGRTILGSTILEDEFTPFDVVRQCSGVTFQGKFKKIVKGTHHWVLLTF 3565
SP|P0C6U8|R1A_CVHSA            CAALKELLQNGMNGRTILGSTILEDEFTPFDVVRQCSGVTFQGKFKKIVKGTHHWMLLTF 3564
TR|Q6JH47|Q6JH47_CVHSA         CAALKELLQNGMNGRTILGSTILEDEFTPFDVVRQCSGVTFQGKFKKIVKGTHHWMLLTF 3564
TR|Q692E5|Q692E5_CVHSA         CAALKELLQNGMNGRTILGSTILEDEFTPFDVVRQCSGVTFQGKFKKIVKGTHHWMLLTF 3564
SP|P0C6F8|R1A_BCHK3            CAALKELLQNGMNGRTILGSTILEDEFTPFDVVRQCSGVTFQGKFKKIVKGTHHWMLLTF 3558
TR|A0A0K1Z0N1|A0A0K1Z0N1_CVHSA CAALKELLQNGMNGRTILGSTILEDEFTPFDVVRQCSGVTFQGKFKKIVKGTHHWMLLTF 3564
SP|P0C6F5|R1A_BC279            CAALKELLQNGMNGRTILGSTILEDEFTPFDVVRQCSGVTFQGKFKKIVKGTHHWMLLTF 3570
SP|P0C6T7|R1A_BCRP3            CAALKELLQNGMNGRTILGSTILEDEFTPFDVVRQCSGVTFQGKFKRIVKGTHHWMLLTF 3562
                               **:*****************::********************. .*: :******:***:
```
