# Supplementary material for: Structural Genomics of SARS-CoV-2 Indicates Evolutionary Conserved Functional Regions of Viral Proteins
Source: Viruses. 2020 Mar 25;12(4):360. doi: 10.3390/v12040360 (PMC7232164; doi:10.3390/v12040360)
Supplement: Supplementary file 1 [file viruses-12-00360-s001.zip › wORF1AB_wNsp3_domain5_human_PBS_mapped.pdf]

Supplementary File 9. Host-viral protein binding sites mapped on the sequences of wNsp3 protein aligned with the closest homologs found in UniProt.

Host-virus Interaction Protein Binding Sites:

wNsp3_domain5-UBC-4MM3

```
QHN73794                          KPHNSHEGKTFYVLPNDDTLRVEAFEYYHTTDPSFLGRYMSALNHTKKWKYPQVNGLTSI 1667
SP|P0C6X7|R1AB_CVHSA              KPHVNHEGKTFFVLPSDDTLRSEAFEYYHTLDESFLGRYMSALNHTKKWKFPQVGGLTSI 1644
TR|Q6UZF5|Q6UZF5_CVHSA            KPHVNHEGKTFFVLPSDDTLRSEAFEYYHTLDESFLGRYMSALNHTKKWKFPQVGGLTSI 1644
TR|Q6UZF1|Q6UZF1_CVHSA            KPHVNHEGKTFFVLPSDDTLRSEAFEYYHTLDESFLGRYMSALNHTKKWKFPQVGGLTSI 1644
TR|Q6JH48|Q6JH48_CVHSA            KPHVNHEGKTFFVLPSDDTLRSEAFEYYHTLDESFLGRYMSALNHTKKWKFPQVGGLTSI 1644
TR|Q692E6|Q692E6_CVHSA            KPHVNHEGKTFFVLPSDDTLRSEAFEYYHTLDESFLGRYMSALNHTKKWKFPQVGGLTSI 1644
TR|A0A0K1YZY7|A0A0K1YZY7_CVHSA KPHVNHEGKTFYVLPSDDTLRSEAFEYYHTLDESFLGRYMSALNHTKKWKFPQVGGLTSI 1644
SP|P0C6W2|R1AB_BCHK3              KPHVNHEGKTFFVLPSDDTLRSEAFEYYHTIDESFLGRYMSALNHTKKWKFPQVGGLTSI 1638
SP|P0C6W6|R1AB_BCRP3              KPHVNHEGKTFFVLPSDDTLRSEAFEYYHTLDESFLGRYMSALNHTKKWKFPQVGGLTSI 1642
SP|P0C6V9|R1AB_BC279              KPHAKHEGKTFFVLPSDDTLRSEAFEYYHTLDESFLGRYMSALNHTKKWKFPQIGGLTSI 1650
TR|A0A0U1WHI4|A0A0U1WHI4_CVHSA KPHVNHEGKTFFVLPSDDTLRSEAFEYYHTLDESFLGRYMSALSHTKKWKFPQVGGLTSI 1639
TR|A0A0U1WHG0|A0A0U1WHG0_CVHSA KPHVNHEGKTFFVLPSDDTLRSEAFEYYHTLDESFLGRYMSALNHTKKWKFPQVGGLTSI 1639
TR|A0A166ZL34|A0A166ZL34_9NIDO KPHVNHEGKTFFVLPSDDTLRSEAFGYYHTLDESFLGRYMSALNHTKKWKFPQVGGLTSI 1639
TR|R9QTB2|R9QTB2_CVHSA            KPHVNHEGKTFFVLPSDDTLRSEAFEYYHTLDESFLGRYMSALSHTKKWKFPQVGGLTSI 1636
TR|R9QTH2|R9QTH2_CVHSA            KPHVNHEGKTFFVLPSDDTLRSEAFEYYHTLDESFLGRYMSALNHTKKWKFPQVGGLTSI 1645
SP|P0C6U8|R1A_CVHSA               KPHVNHEGKTFFVLPSDDTLRSEAFEYYHTLDESFLGRYMSALNHTKKWKFPQVGGLTSI 1644
TR|Q6JH47|Q6JH47_CVHSA            KPHVNHEGKTFFVLPSDDTLRSEAFEYYHTLDESFLGRYMSALNHTKKWKFPQVGGLTSI 1644
TR|Q692E5|Q692E5_CVHSA            KPHVNHEGKTFFVLPSDDTLRSEAFEYYHTLDESFLGRYMSALNHTKKWKFPQVGGLTSI 1644
SP|P0C6F8|R1A_BCHK3               KPHVNHEGKTFFVLPSDDTLRSEAFEYYHTIDESFLGRYMSALNHTKKWKFPQVGGLTSI 1638
TR|A0A0K1Z0N1|A0A0K1Z0N1_CVHSA KPHVNHEGKTFYVLPSDDTLRSEAFEYYHTLDESFLGRYMSALNHTKKWKFPQVGGLTSI 1644
SP|P0C6F5|R1A_BC279               KPHAKHEGKTFFVLPSDDTLRSEAFEYYHTLDESFLGRYMSALNHTKKWKFPQIGGLTSI 1650
SP|P0C6T7|R1A_BCRP3               KPHVNHEGKTFFVLPSDDTLRSEAFEYYHTLDESFLGRYMSALNHTKKWKFPQVGGLTSI 1642
                                  *** .******:***.***** *** **** * **********.******:**:.*****

QHN73794                          KWADNNCYLATALLTLQQIELKFNPPALQDAYYRARAGEAANFCALILAYCNKTVGELGD 1727
SP|P0C6X7|R1AB_CVHSA              KWADNNCYLSSVLLALQQLEVKFNAPALQEAYYRARAGDAANFCALILAYSNKTVGELGD 1704
TR|Q6UZF5|Q6UZF5_CVHSA            KWADNNCYLSSVLLALQQLEVKFNAPALQEAYYRARAGDAANFCALILAYSNKTVGELGD 1704
TR|Q6UZF1|Q6UZF1_CVHSA            KWADNNCYLSSVLLALQQLEVKFNAPALQEAYYRARAGDAANFCALILAYSNKTVGELGD 1704
TR|Q6JH48|Q6JH48_CVHSA            KWADNNCYLSSVLLALQQLEVKFNAPALQEAYYRARAGDAANFCALILAYSNKTVGELGD 1704
TR|Q692E6|Q692E6_CVHSA            KWADNNCYLSSVLLALQQLEVKFNAPALQEAYYRARAGDAANFCALILAYSNKTVGELGD 1704
TR|A0A0K1YZY7|A0A0K1YZY7_CVHSA KWADNNCYLSSVLLALQQIEVKFNAPALQEAYYRARAGDAANFCALILAYSNKTVGELGD 1704
SP|P0C6W2|R1AB_BCHK3              KWADNNCYLSSVLLALQQVEVKFNAPALQEAYYRARAGDAANFCALILAYSNKTVGELGD 1698
SP|P0C6W6|R1AB_BCRP3              KWADNNCYLSSVLLALQQIEVKFNAPALQEAYYRARAGDAANFCALILAYSNKTVGELGD 1702
SP|P0C6V9|R1AB_BC279              KWADNNCYLSSVLLALQQIEVKFNAPALQEAYYRARAGDAANFCALILAYSNRTVGELGD 1710
TR|A0A0U1WHI4|A0A0U1WHI4_CVHSA KWADNNCYLSSVLLALQQIEVKFNAPALQEAYYRARAGDAANFCALILAYSNKTVGELGD 1699
TR|A0A0U1WHG0|A0A0U1WHG0_CVHSA KWADNNCYLSSVLLALQQIEVKFNAPALQEAYYRARAGEAANFCALILAYSNKTVGELGD 1699
TR|A0A166ZL34|A0A166ZL34_9NIDO KWADNNCYLSSVLLALQQIEVKFNAPALQEAYYRARAGDAANFCALTLAYSNKTVGDLGD 1699
TR|R9QTB2|R9QTB2_CVHSA            KWADNNCYLSSVLLALQQIEVKFNAPALQEAYYRARAGDAANFCALILAYSYKTVGELGD 1696
TR|R9QTH2|R9QTH2_CVHSA            KWADNNCYLSSVLLALQQIEVKFNAPALQEAYYRARAGDAANFCALILAYSNKTVGELGD 1705
SP|P0C6U8|R1A_CVHSA               KWADNNCYLSSVLLALQQLEVKFNAPALQEAYYRARAGDAANFCALILAYSNKTVGELGD 1704
TR|Q6JH47|Q6JH47_CVHSA            KWADNNCYLSSVLLALQQLEVKFNAPALQEAYYRARAGDAANFCALILAYSNKTVGELGD 1704
TR|Q692E5|Q692E5_CVHSA            KWADNNCYLSSVLLALQQLEVKFNAPALQEAYYRARAGDAANFCALILAYSNKTVGELGD 1704
SP|P0C6F8|R1A_BCHK3               KWADNNCYLSSVLLALQQVEVKFNAPALQEAYYRARAGDAANFCALILAYSNKTVGELGD 1698
TR|A0A0K1Z0N1|A0A0K1Z0N1_CVHSA KWADNNCYLSSVLLALQQIEVKFNAPALQEAYYRARAGDAANFCALILAYSNKTVGELGD 1704
SP|P0C6F5|R1A_BC279               KWADNNCYLSSVLLALQQIEVKFNAPALQEAYYRARAGDAANFCALILAYSNRTVGELGD 1710
SP|P0C6T7|R1A_BCRP3               KWADNNCYLSSVLLALQQIEVKFNAPALQEAYYRARAGDAANFCALILAYSNKTVGELGD 1702
                                  *********::.**:***:*:*** ****:********:******* ***. :***:***

QHN73794                          VRETMSYLFQHANLDSCKRVLNVVCKTCGQQQTTLKGVEAVMYMGTLSYEQFKKGVQIPC 1787
SP|P0C6X7|R1AB_CVHSA              VRETMTHLLQHANLESAKRVLNVVCKHCGQKTTTLTGVEAVMYMGTLSYDNLKTGVSIPC 1764
TR|Q6UZF5|Q6UZF5_CVHSA            VRETMTHLLQHANLESAKRVLNVVCKHCGQKTTTLTGVEAVMYMGTLSYDNLKTGVSIPC 1764
TR|Q6UZF1|Q6UZF1_CVHSA            VRETMTHLLQHANLESAKRVLNVVCKHCGQKTTTLTGVEAVMYMGTLSYDNLKTGVSIPC 1764
TR|Q6JH48|Q6JH48_CVHSA            VRETMTHLLQHANLESAKRVLNVVCKHCGQKTTTLTGVEAVMYMGTLSYDNLKTGVSIPC 1764
TR|Q692E6|Q692E6_CVHSA            VRETMTHLLQHANLESAKRVLNVVCKHCGQKTTTLTGVEAVMYMGTLSYDNLKTGVSIPC 1764
TR|A0A0K1YZY7|A0A0K1YZY7_CVHSA VRETMTHLLQHANLESAKRVLNVVCKHCGQKTTTLTGVEAVMYMGTLSYDNLKTGVSIPC 1764
SP|P0C6W2|R1AB_BCHK3              VRETMTHLLQHANLESAKRVLNVVCKHCGQKTTTLKGVEAVMYMGTLSYDELKTGVSIPC 1758
SP|P0C6W6|R1AB_BCRP3              VRETMTHLLQHANLESAKRVLNVVCKHCGQKTTTLTGVEAVMYMGTLSYDNLKMGVSIPC 1762
SP|P0C6V9|R1AB_BC279              VRETMTHLLQHANLESAKRVLNVVCKTCGQKSTTLTGVEAVMYMGTLSYEELKTGVTIPC 1770
TR|A0A0U1WHI4|A0A0U1WHI4_CVHSA VRETMTHLLQHANLEFAKRVLNLVCKHCGQKTTTLTGVEAVMYMGTLSYDELKTGVSIPC 1759
TR|A0A0U1WHG0|A0A0U1WHG0_CVHSA VRETMTHLLQHANLEFAKRVLNLVCKHCGQKTTTLTGVEAVMYMGTLSYDELKTGVSIPC 1759
TR|A0A166ZL34|A0A166ZL34_9NIDO VRETMTHLLQHANLEFAKRVLNLVCKHCGQKTTTLTGVEAVMYMGTLSYDELKTGVSIPC 1759
TR|R9QTB2|R9QTB2_CVHSA            VRETMTHLLQHANLESAKRVLNVVCKHCGQKTTTLTGVEAVMYMGTLSYDELKTGVSIPC 1756
TR|R9QTH2|R9QTH2_CVHSA            VRETMAHLLQHANLESAKRVLNVVCKHCGQKTTTLTGVEAVMYMGTLSYDNLKTGVSVPC 1765
SP|P0C6U8|R1A_CVHSA               VRETMTHLLQHANLESAKRVLNVVCKHCGQKTTTLTGVEAVMYMGTLSYDNLKTGVSIPC 1764
TR|Q6JH47|Q6JH47_CVHSA            VRETMTHLLQHANLESAKRVLNVVCKHCGQKTTTLTGVEAVMYMGTLSYDNLKTGVSIPC 1764
TR|Q692E5|Q692E5_CVHSA            VRETMTHLLQHANLESAKRVLNVVCKHCGQKTTTLTGVEAVMYMGTLSYDNLKTGVSIPC 1764
```

```
SP|P0C6F8|R1A_BCHK3             VRETMTHLLQHANLESAKRVLNVVCKHCGQKTTTLKGVEAVMYMGTLSYDELKTGVSIPC 1758
TR|A0A0K1Z0N1|A0A0K1Z0N1_CVHSA VRETMTHLLQHANLESAKRVLNVVCKHCGQKTTTLTGVEAVMYMGTLSYDNLKTGVSIPC 1764
SP|P0C6F5|R1A_BC279             VRETMTHLLQHANLESAKRVLNVVCKTCGQKSTTLTGVEAVMYMGTLSYEELKTGVTIPC 1770
SP|P0C6T7|R1A_BCRP3             VRETMTHLLQHANLESAKRVLNVVCKHCGQKTTTLTGVEAVMYMGTLSYDNLKMGVSIPC 1762
                                *****::*:*****: .*****:*** ***: ***.*************:::* ** :**
                                _      _              __                _    ___ _
QHN73794                        TCGKQATKYLVQQESPFVMMSAPPAQYELKHGTFTCASEYTGNYQCGHYKHITSKETLYC 1847
SP|P0C6X7|R1AB_CVHSA            VCGRDATQYLVQQESSFVMMSAPPAEYKLQQGTFLCANEYTGNYQCGHYTHITAKETLYR 1824
TR|Q6UZF5|Q6UZF5_CVHSA          VCGRDATQYLVQQESSFVMMSAPPAEYKLQQGTFLCANEYTGNYQCGHYTHITAKETLYR 1824
TR|Q6UZF1|Q6UZF1_CVHSA          VCGRDATQYLVQQESSFVMMSAPPAEYKLQQGTFLCANEYTGNYQCGHYTHITAKETLYR 1824
TR|Q6JH48|Q6JH48_CVHSA          VCGRDATQYLVQQESSFVMMSAPPAEYKLQQGTFLCANEYTGNYQCGHYTHITAKETLYR 1824
TR|Q692E6|Q692E6_CVHSA          VCGRDATQYLVQQESSFVMMSAPPAEYKLQQGTFLCANEYTGNYQCGHYTHITAKETLYR 1824
TR|A0A0K1YZY7|A0A0K1YZY7_CVHSA VCGRDATQYLIQQESSFVMMSAPPAEYKLQQGTFLCANEYTGNYQCGHYTHVTAKETLYR 1824
SP|P0C6W2|R1AB_BCHK3            VCGRNATQYLVQQESSFVMMSAPPAEYKLQQGAFLCANEYTGNYQCGHYTHITAKETLYR 1818
SP|P0C6W6|R1AB_BCRP3            VCGRDATQYLVQQESSFVMMSAPPAEYKLQQGTFLCANEYTGNYQCGHYTHITAKETLYR 1822
SP|P0C6V9|R1AB_BC279            ICGRDATQYLVQQESSFVMMSAPPSEYTLQQGAFLCANEYTGSYQCGHYTHVTVKETLYR 1830
TR|A0A0U1WHI4|A0A0U1WHI4_CVHSA VCGRGATQYLVQQESSFVMMSAPPAEYKLQQGTFLCANEYTGNYQCGHYTHITAKETLYR 1819
TR|A0A0U1WHG0|A0A0U1WHG0_CVHSA VCGRDATQYLVQQESSFVMMSAPPAEYKLQQSTFLCANEYTGNYQCGHYTHITAKETLYR 1819
TR|A0A166ZL34|A0A166ZL34_9NIDO VCGRDATQYLVQQESSFVMMSAPPAEYKLQQGTFLCANEYTGNYQCGHYTHITAKETLYH 1819
TR|R9QTB2|R9QTB2_CVHSA          VCGRNATQYLVQQESSFVMMSAPPAEYKLQQGTFLCANEYTGNYQCGHYTHITAKETLYR 1816
TR|R9QTH2|R9QTH2_CVHSA          VCGRDATQYLVQQESSFVMMSAPPAEYKLQQGTFLCANEYTGNYQCGHYTHITAKETLYR 1825
SP|P0C6U8|R1A_CVHSA             VCGRDATQYLVQQESSFVMMSAPPAEYKLQQGTFLCANEYTGNYQCGHYTHITAKETLYR 1824
TR|Q6JH47|Q6JH47_CVHSA          VCGRDATQYLVQQESSFVMMSAPPAEYKLQQGTFLCANEYTGNYQCGHYTHITAKETLYR 1824
TR|Q692E5|Q692E5_CVHSA          VCGRDATQYLVQQESSFVMMSAPPAEYKLQQGTFLCANEYTGNYQCGHYTHITAKETLYR 1824
SP|P0C6F8|R1A_BCHK3             VCGRNATQYLVQQESSFVMMSAPPAEYKLQQGAFLCANEYTGNYQCGHYTHITAKETLYR 1818
TR|A0A0K1Z0N1|A0A0K1Z0N1_CVHSA VCGRDATQYLIQQESSFVMMSAPPAEYKLQQGTFLCANEYTGNYQCGHYTHVTAKETLYR 1824
SP|P0C6F5|R1A_BC279             ICGRDATQYLVQQESSFVMMSAPPSEYTLQQGAFLCANEYTGSYQCGHYTHVTVKETLYR 1830
SP|P0C6T7|R1A_BCRP3             VCGRDATQYLVQQESSFVMMSAPPAEYKLQQGTFLCANEYTGNYQCGHYTHITAKETLYR 1822
                                 **: **:**:**** ********::* *::.:* **.****.******.*:* *****
                                                _
QHN73794                        IDGALLTKSSEYKGPITDVFYKENSYTTTIKPVTYKLDGVVCTEIDPKLDNYYKKDNSYF 1907
SP|P0C6X7|R1AB_CVHSA            IDGAHLTKMSEYKGPVTDVFYKETSYTTTIKPVSYKLDGVTYTEIEPKLDGYYKKDNAYY 1884
TR|Q6UZF5|Q6UZF5_CVHSA          IDGAHLTKMSEYKGPVTDVFYKETSYTTTIKPVSYKLDGVTYTEIEPKLDGYYKKDNAYY 1884
TR|Q6UZF1|Q6UZF1_CVHSA          IDGAHLTKMSEYKGPVTDVFYKETSYTTTIKPVSYKLDGVTYTEIEPKLDGYYKKDNAYY 1884
TR|Q6JH48|Q6JH48_CVHSA          IDGAHLTKMSEYKGPVTDVFYKETSYTTTIKPVSYKLDGVTYTEIEPKLDGYYKKDNAYY 1884
TR|Q692E6|Q692E6_CVHSA          IDGAHLTKMSEYKGPVTDVFYKETSYTTTIKPVSYKLDGVTYTEIEPKLDGYYKKDNAYY 1884
TR|A0A0K1YZY7|A0A0K1YZY7_CVHSA IDGAHLTKMSEYKGPVTDVFYKETSYTTTIKPVSYKLDGVTYTEIEPKLDGYYKKDNAYY 1884
SP|P0C6W2|R1AB_BCHK3            VDGAHLTKMSEYKGPVTDVFYKETSYTTAIKPVSYKLDGVTYTEIEPKLDGYYKKGNAYY 1878
SP|P0C6W6|R1AB_BCRP3            IDGAHLTKMSEYKGPVTDVFYKETSYTTTIKPVSYKLDGVTYTEIEPKLDGYYKKDNAYY 1882
SP|P0C6V9|R1AB_BC279            IDGAYLTKMSEYKGPVTDVFYKEISYTTTIKPVSYKLDGVIYTEIQPKLDEYYKKDNAYY 1890
TR|A0A0U1WHI4|A0A0U1WHI4_CVHSA IDGAHLTKMSEYKGPVTDVFYKETSYTTTIKPVSYKLDGVTYTEIEPKLDGYYKKDNAYY 1879
TR|A0A0U1WHG0|A0A0U1WHG0_CVHSA IDGAHLTKMSEYKGPVTDVFYKETSYTTTIKPVSYKLDGVTYTEIEPKLDGYYKKDNAYY 1879
TR|A0A166ZL34|A0A166ZL34_9NIDO IDGAHLTKMSEYKGPVTDVFYKETSYTTTIKPVSYKLDGVTYTEIEPKLDGYYKKDNAY- 1878
TR|R9QTB2|R9QTB2_CVHSA          IDGAHLTKMSEYKGPVTDVFYKETSYTTTIKPVSYKLDGVTYTEIEPKLDGYYKKDNAYY 1876
TR|R9QTH2|R9QTH2_CVHSA          IDGAHLTKMSEYKGPVTDVFYKETSYTTTIKPVSYKLDGVTYTEIEPKLDGYYKKDNAYY 1885
SP|P0C6U8|R1A_CVHSA             IDGAHLTKMSEYKGPVTDVFYKETSYTTTIKPVSYKLDGVTYTEIEPKLDGYYKKDNAYY 1884
TR|Q6JH47|Q6JH47_CVHSA          IDGAHLTKMSEYKGPVTDVFYKETSYTTTIKPVSYKLDGVTYTEIEPKLDGYYKKDNAYY 1884
TR|Q692E5|Q692E5_CVHSA          IDGAHLTKMSEYKGPVTDVFYKETSYTTTIKPVSYKLDGVTYTEIEPKLDGYYKKDNAYY 1884
SP|P0C6F8|R1A_BCHK3             VDGAHLTKMSEYKGPVTDVFYKETSYTTAIKPVSYKLDGVTYTEIEPKLDGYYKKGNAYY 1878
TR|A0A0K1Z0N1|A0A0K1Z0N1_CVHSA IDGAHLTKMSEYKGPVTDVFYKETSYTTTIKPVSYKLDGVTYTEIEPKLDGYYKKDNAYY 1884
SP|P0C6F5|R1A_BC279             IDGAYLTKMSEYKGPVTDVFYKEISYTTTIKPVSYKLDGVIYTEIQPKLDEYYKKDNAYY 1890
SP|P0C6T7|R1A_BCRP3             IDGAHLTKMSEYKGPVTDVFYKETSYTTTIKPVSYKLDGVTYTEIEPKLDGYYKKDNAYY 1882
                                :*** *** ******:******* ****:****:******  ***:**** ****.*:*
```
